# Long-term second primary cancer risk in adolescent and young adult (15-39 years) cancer survivors: a population-based study in the Netherlands between 1989 and 2018

**DOI:** 10.1016/j.esmoop.2023.102203

**Published:** 2024-01-02

**Authors:** D.J. van der Meer, W.T.A. van der Graaf, D. van de Wal, H.E. Karim-Kos, O. Husson

**Affiliations:** 1 Department of Medical Oncology, Netherlands Cancer Institute—Antoni van Leeuwenhoek, Amsterdam; 2 Department of Psychosocial Research and Epidemiology, Netherlands Cancer Institute, Amsterdam; 3 Department of Medical Oncology, Erasmus MC Cancer Institute, Erasmus University Medical Center, Rotterdam; 4 Princess Máxima Center for Pediatric Oncology, Utrecht; 5 Department of Research and Development, Netherlands Comprehensive Cancer Organization (IKNL), Utrecht; 6 Department of Surgical Oncology, Erasmus MC Cancer Institute, Erasmus University Medical Center, Rotterdam, The Netherlands

**Keywords:** oncology, adolescents and young adults, cancer survivorship, late effects, second cancer risk, population-based

## Abstract

**Background:**

Few studies have comprehensively investigated the long-term second cancer risk among adolescent and young adult (AYA, aged 15-39 years) cancer survivors. This study investigated the long-term second cancer risk by including the full range of first and second cancer combinations with at least 10 observations in the Netherlands between 1989 and 2018.

**Materials and methods:**

First and second primary cancer data of all 6-month AYA cancer survivors were obtained from the nationwide population-based Netherlands Cancer Registry. Excess cancer risk compared to the general population was assessed with standardized incidence ratio (SIR) and absolute excess risk (AER) statistics up to 25 years after diagnosis. Cumulative incidences were estimated, using death as a competing risk factor. Analyses were carried out with and without applying multiple cancer rules.

**Results:**

The cohort included 99 502 AYA cancer survivors. Male survivors had a 2-fold higher risk of developing any cancer compared to the general population, whereas this was around 1.3-fold in females. AERs were 17.5 and 10.1 per 10 000 person-years for males and females. The long-term excess risk of cancer was significantly higher for most first and second primary cancer combinations, but comparable and lower risk estimates were also observed. Application of the multiple cancer rules resulted in a noticeable risk underestimation in melanoma, testicular, and breast cancer survivors. Risk outcomes remained similar in most cases otherwise. The cumulative incidence of second cancer overall increased over time up to 8.9% in males and 10.3% in females at 25 years’ follow-up. Highest long-term cumulative incidences were observed among lymphoma survivors (13.3% males and 18.9% females).

**Conclusions:**

AYA cancer survivors have a higher cancer risk compared to the general population for most cancers up to 25 years after their initial cancer diagnosis. Additional studies that investigate risk factors for the specific cancer type combinations are needed to develop personalized follow-up strategies.

## Highlights

• Between 1989 and 2018, 1471 (3.7%) male and 2873 (4.8%) female AYAs developed second cancer.
• Male AYA survivors had a 2-fold and females a 1.3-fold higher 25-year cancer risk compared to the general population.
• A higher cancer risk among AYA survivors was found for most first and second primary cancer combinations.
• The cumulative incidence of second cancer overall increased up to 8.9% in males and 10.3% in females at 25-year follow-up.
• Applying the multiple cancer rules resulted in a risk underestimation in melanoma, testicular, and breast cancer survivors.

## Introduction

Adolescents and young adults (AYAs, defined as individuals aged 15-39 years at first cancer diagnosis) form a distinct population that is often overlooked within the oncology care setting.^1^^,^^2^ AYAs present themselves with a distinct spectrum of cancers, varying from cancers frequently found among children (e.g. acute lymphatic leukaemia, Ewing’s sarcoma), to cancers usually found in older adults (e.g. colorectal, lung, and breast carcinomas), but also cancers with the highest incidence at AYA age (e.g. Hodgkin’s lymphoma, melanoma, germ cell tumours, and thyroid carcinomas).^3^

Worldwide, the incidence of cancer at AYA age is increasing for most cancer types, while the survival and mortality are steadily improving.^4^, ^5^, ^6^, ^7^, ^8^, ^9^ Current estimates show that ∼85% of all AYAs are still alive 5 years after their primary cancer diagnosis.^10^ As such, there is a growing population of AYA cancer survivors that is at risk of developing survivorship-related adverse health outcomes later in life, creating new challenges for AYA survivors and their loved ones.^11^ Previous international studies have shown that AYA cancer survivors are at an increased risk of experiencing several cancer and treatment-related (late) adverse health effects, including premature mortality,^12^ cardiovascular^13^, ^14^, ^15^ and endocrine diseases,^16^^,^^17^ and second primary cancers.^11^^,^^18^, ^19^, ^20^, ^21^, ^22^, ^23^, ^24^ AYA survivors in high-income countries are shown to have a 1.2- to 2-fold higher risk of developing any second cancer compared to the general population, resulting on average in 11-23 excess cancers per 10 000 person-years.^18^^,^^20^^,^^23^^,^^24^ Meanwhile, cumulative incidences of second cancer show that between 10% and 17% of AYA survivors will develop a second cancer after 20-35 years of follow-up.^18^^,^^20^, ^21^, ^22^, ^23^, ^24^

These initial findings provide important information. Nevertheless, only a few studies have comprehensively investigated the long-term second cancer risk among AYA survivors across a full range of first and second diagnostic subgroups.^18^^,^^20^, ^21^, ^22^, ^23^, ^24^ Moreover, most previous studies were not AYA-specific or investigated just one or a few specific first primary cancer types.^25^, ^26^, ^27^, ^28^, ^29^, ^30^ As such, there is a general lack of detailed knowledge about the second cancer risk pertaining to a large number of first and second primary cancer types among the vulnerable AYA survivor population. Identification of distinct patient groups that are at a particular high risk of developing a second cancer can further help to guide the development of better survivorship care guidelines aimed at prevention and early detection of second cancers among AYAs, which has been shown to improve relative survival outcomes up to 47%.^31^ Therefore, more detailed second cancer risk studies that describe long-term risk patterns in AYAs are warranted. This study investigated the long-term risk of developing a second malignant cancer overall and by including the full range of first and second cancer combinations with at least 10 observations up to 25 years after diagnosis in the Netherlands between 1989 and 2018.

## Materials and methods

### Data collection

Data of all primary malignancies were obtained from the population-based Netherlands Cancer Registry (NCR), with a national coverage since 1989 and an estimated completeness of at least 95%.^32^ The NCR is based on notification from the nationwide network and registry of histopathology and cytopathology (PALGA) and the national registry of hospital discharges. NCR records contain information about patient, tumour, and primary treatment characteristics collected from medical records by trained registration clerks of the NCR. Malignancies within the NCR are coded based on the International Classification of Diseases for Oncology (ICD-O), using the first edition between 1989 and 1993, the second between 1993 and 2000, and the third edition since 2001.^33^ Information on vital status (i.e. dead, alive, emigrated) is obtained through annual linkage between the NCR and the Nationwide Personal Records Database (BRP, last linkage on 1 February 2020). In the current analyses, the first two primary malignant cancers (ICD-O behaviour /3) per AYA were included. Subsequent primary malignancies were omitted, including 372 third, 34 fourth, and 8 fifth malignancies. AYAs were eligible for inclusion when diagnosed with first cancer at ages 15-39 years in the Netherlands between 1989 and 2018.^2^ The NCR did not provide data about basal-cell skin and lip carcinomas and second primary squamous cell tumours of the skin. Myelodysplastic and myeloproliferative syndromes were available since 2002. Moreover, cancers were included only when they satisfied the international rules for multiple cancers published by the International Agency for Research on Cancer (IARC), which exclude all extensions, recurrences, and metastasis.^33^ To minimize detection and information bias, AYA survivors with a follow-up <6 months after first primary cancer diagnosis were excluded [*n* = 7002 (6.6%) total, including *n* = 318 (0.3%) second cancers].^34^

### Diagnostic classification

Cancers were grouped into 11 diagnostic main groups and further categorized into more detailed diagnostic subgroups according to the topography (anatomic location), morphology, and behaviour based AYA-specific classification scheme developed by Barr and colleagues.^35^^,^^36^ Detailed definitions for each of the diagnostic main groups and subgroups are provided elsewhere.^35^^,^^36^

### Statistical analysis

Expected numbers of second cancer in the general population were calculated by multiplying the accumulated person-time at risk among AYA cancer survivors by the corresponding age- (5-year bands), sex-, calendar year-, and cancer type-specific incidence rates in the general population and then summing the results. Cancer incidence rates for the general background population were provided by the NCR and included annual crude rates per 100 000 person-years for all malignancies diagnosed in the Netherlands between 1989 and 2018. Person-time at risk for each of the strata was obtained by accumulating individual follow-up times (in days) from 6-month survival until the date of second cancer diagnosis, death, loss to follow-up, or 31 December 2018, whichever came first. Standardized incidence ratios (SIRs) and absolute excess risks (AERs) per 10 000 person-years with corresponding exact Poisson distribution-derived 95% confidence intervals (CIs) were calculated from the observed and expected numbers of second cancers, using standard statistical methods.^37^ Second cancer cumulative incidences with 95% CIs were estimated while using death as a competing risk.^38^^,^^39^ Patients (*n* = 7) with identical follow-up times for developing a second cancer and death were included within the competing risk group. All statistical analyses over the entire study period (1989-2018) were carried out separately for males and females. Analyses were further stratified by age at first cancer diagnosis (5-year age bands), follow-up period, and cancer type (first and second cancer). To allow meaningful interpretation, analyses were truncated at 25 years and outcomes were listed only if at least 10 second cancers were observed (all outcomes were listed for analyses by follow-up period). Statistical analyses were carried out with Stata/SE 17.0 (StataCorp LP, College Station, TX). Two-sided *P* values <0.05 were considered statistically significant. Study approval was granted by the Privacy Review Board of the NCR. Data used in this study can be requested from the NCR (study number K20.066, www.iknl.nl).

### Sensitivity analysis

An underestimation of second cancer risk may arise when applying the international rules for multiple cancers in cases with a high frequency of consecutive malignancies that are considered topographically and morphologically identical. Due to this inherent limitation, we repeated all analyses by including all first two malignancies regardless of whether they satisfied the International Association of Cancer Registries (IACR)/IARC rules for multiple cancers. Other research parameters were kept the same.

## Results

### Population and tumour characteristics

A flow chart of the study population selection procedure is presented in Supplementary Figure S1A, available at https://doi.org/10.1016/j.esmoop.2023.102203. Between 1989 and 2018, 99 502 AYAs were diagnosed with a first primary cancer and survived at least 6 months (59.9% females). Cohort characteristics are shown in Table 1 and Supplementary Table S1A, available at https://doi.org/10.1016/j.esmoop.2023.102203. First and second cancer type distributions are displayed in Supplementary Figure S2, available at https://doi.org/10.1016/j.esmoop.2023.102203. Most AYAs had stage I first primary cancer (42.9% males and 45.3% females). Most male AYAs were diagnosed with first primary testicular cancers (29.4%), lymphomas (15.9%), melanomas (14.8%), and gastrointestinal tract carcinomas (7.3%). Females were mostly diagnosed with first primary breast carcinomas (32.8%), melanomas (18.5%), uterine cervix carcinomas (10.5%), and lymphomas (7.7%). The median follow-up time was 8.6 years [interquartile range (IQR) 2.8-16.6 years] for males and 9.1 years (IQR 3.3-17.2 years) for females. During this risk period, 1471 (3.7%) male and 2873 (4.8%) female AYAs developed a second primary cancer. Age at second cancer diagnosis ranged from 17 to 68 years with a median age of 46 years for both sexes. Second gastrointestinal tract carcinomas (21.8%) were most commonly diagnosed among male survivors, whereas this was second primary breast carcinoma (26.9%) among female survivors (Table 1 and Supplementary Figure S2, available at https://doi.org/10.1016/j.esmoop.2023.102203).

**Table 1:** Population, tumour, and treatment characteristics of 6-month AYA (aged 15-39 years) cancer survivors diagnosed with first and second primary malignant cancer in the Netherlands between 1989 and 2018

	First primary cancers		Second primary cancers	
	Males	Females	Males	Females
	*n* (%)	*n* (%)	*n* (%)	*n* (%)
**Total**^a^	39 892 (100)	59 610 (100)	1471 (100)	2873 (100)
**Median age (IQR)**	32.0 (26.0-36.0)	34.0 (29.0-37.0)	46.0 (38.0-52.0)	45.5 (39.0-52.0)
**Age at diagnosis (years)**^b^				
15-19	3219 (8.1)	2581 (4.3)	8 (0.5)	4 (0.1)
20-24	5242 (13.1)	4458 (7.5)	19 (1.3)	14 (0.5)
25-29	7870 (19.7)	8642 (14.5)	64 (4.4)	45 (1.6)
30-34	10 295 (25.8)	16 635 (27.9)	95 (6.5)	165 (5.7)
35-39	13 266 (33.3)	27 294 (45.8)	220 (15.0)	377 (13.1)
40-44	NA	NA	249 (16.9)	604 (21.0)
45-49	NA	NA	287 (19.5)	629 (21.9)
50-54	NA	NA	238 (16.2)	491 (17.1)
55-59	NA	NA	188 (12.8)	361 (12.6)
60-64	NA	NA	90 (6.1)	161 (5.6)
65-68	NA	NA	13 (0.9)	22 (0.8)
**Tumour stage (TNM, FIGO, and Ann Arbor)**				
Stage I	17 114 (42.9)	27 020 (45.3)	469 (31.9)	1114 (38.8)
Stage II	6307 (15.8)	15 707 (26.3)	200 (13.6)	523 (18.2)
Stage III	4665 (11.7)	6013 (10.1)	214 (14.5)	402 (14.0)
Stage IV	3271 (8.2)	3403 (5.7)	292 (19.9)	464 (16.2)
Other/unknown	8535 (21.4)	7467 (12.5)	296 (20.1)	370 (12.9)
**Period of diagnosis**				
1989-1998	12 008 (30.1)	18 972 (31.8)	101 (6.9)	155 (5.4)
1999-2008	13 811 (34.6)	20 679 (34.7)	393 (26.7)	761 (26.5)
2009-2018	14 073 (35.3)	19 959 (33.5)	977 (66.4)	1957 (68.1)
**Median follow-up (IQR)**	8.6 (2.8-16.6)	9.1 (3.3-17.2)	NA	NA
**Follow-up (years)**^c^				
0-4	14 143 (35.5)	20 035 (33.6)	NA	NA
5-9	7790 (19.5)	11 690 (19.6)	NA	NA
10-14	6247 (15.7)	9383 (15.7)	NA	NA
15-19	5019 (12.6)	7747 (13.0)	NA	NA
20-25	4673 (11.7)	7469 (12.5)	NA	NA
≥26	2020 (5.1)	3286 (5.5)	NA	NA
**Cancer types**				
**1. Leukaemia and related disorders**	**2318 (5.8)**	**1951 (3.3)**	**99 (6.7)**	**99 (3.4)**
1.1 Acute lymphoblastic leukaemia	660 (1.7)	407 (0.7)	13 (0.9)	5 (0.2)
1.2 Acute myeloid leukaemia	616 (1.5)	674 (1.1)	38 (2.6)	41 (1.4)
1.3 Chronic myeloid leukaemia	433 (1.1)	264 (0.4)	11 (0.7)	11 (0.4)
1.4 Chronic lymphocytic leukaemia	114 (0.3)	45 (0.1)	8 (0.5)	12 (0.4)
1.5 Polycythaemia vera	74 (0.2)	71 (0.1)	1 (0.1)	1 (0.0)
1.6 Essential thrombocythemia	145 (0.4)	283 (0.5)	6 (0.4)	9 (0.3)
1.7 Primary myelofibrosis	20 (0.1)	19 (0.0)	1 (0.1)	1 (0.0)
1.8 Myelodysplastic syndrome (MDS)	86 (0.2)	100 (0.2)	15 (1.0)	13 (0.5)
1.9 Other and unspecified leukaemia and related disorders	170 (0.4)	88 (0.1)	6 (0.4)	6 (0.2)
**2. Lymphomas**	**6348 (15.9)**	**4614 (7.7)**	**132 (9.0)**	**98 (3.4)**
2.1 Non-Hodgkin’s lymphomas	2764 (6.9)	1693 (2.8)	86 (5.8)	82 (2.9)
2.2 Hodgkin’s lymphoma	3210 (8.0)	2705 (4.5)	30 (2.0)	5 (0.2)
2.3 Myeloma	180 (0.5)	106 (0.2)	7 (0.5)	10 (0.3)
2.4 Cutaneous lymphomas	26 (0.1)	15 (0.0)	1 (0.1)	NA
2.5 Other B-cell and T-cell lymphomas	103 (0.3)	56 (0.1)	5 (0.3)	1 (0.0)
2.6 Other lymphomas, specified and unspecified	65 (0.2)	39 (0.1)	3 (0.2)	NA
**3. CNS and other intracranial and intraspinal neoplasms**	**2556 (6.4)**	**1761 (3.0)**	**41 (2.8)**	**39 (1.4)**
3.1 Astroglial and related neoplasms	2257 (5.7)	1546 (2.6)	36 (2.4)	33 (1.1)
3.2 Medulloblastoma and other invasive embryonal CNS tumours	137 (0.3)	88 (0.1)	NA	1 (0.0)
3.3 Neuroblastomas/ganglioneuromas	3 (0.0)	5 (0.0)	NA	NA
3.4 Neuronal and mixed neuronal-glial neoplasms	9 (0.0)	3 (0.0)	NA	NA
3.5 Meningiomas	15 (0.0)	14 (0.0)	2 (0.1)	NA
3.6 Choroid plexus neoplasms	1 (0.0)	2 (0.0)	NA	NA
3.8 Pituitary neoplasms	4 (0.0)	NA	NA	NA
3.9 Pineal neoplasms	16 (0.0)	17 (0.0)	NA	NA
3.10 Other and unspecified CNS neoplasms	114 (0.3)	86 (0.1)	3 (0.2)	5 (0.2)
**4. Sarcomas**	**2469 (6.2)**	**2229 (3.7)**	**52 (3.5)**	**105 (3.7)**
4.1 Osteosarcoma	333 (0.8)	221 (0.4)	3 (0.2)	11 (0.4)
4.2 Chondrosarcoma	339 (0.8)	339 (0.6)	4 (0.3)	21 (0.7)
4.3 Ewing’s family of tumours	275 (0.7)	177 (0.3)	3 (0.2)	4 (0.1)
4.4 Fibromatous neoplasms	532 (1.3)	574 (1.0)	8 (0.5)	14 (0.5)
4.5 Liposarcoma	208 (0.5)	203 (0.3)	2 (0.1)	9 (0.3)
4.6 Synovial sarcoma	158 (0.4)	126 (0.2)	2 (0.1)	5 (0.2)
4.7 Leiomyosarcoma	144 (0.4)	171 (0.3)	3 (0.2)	10 (0.3)
4.8 Rhabdomyosarcoma	130 (0.3)	74 (0.1)	1 (0.1)	3 (0.1)
4.9 Gastrointestinal stromal tumour, malignant	71 (0.2)	58 (0.1)	4 (0.3)	4 (0.1)
4.10 Spindle cell sarcoma	15 (0.0)	12 (0.0)	2 (0.1)	1 (0.0)
4.11 Epithelioid sarcoma	40 (0.1)	22 (0.0)	NA	1 (0.0)
4.12 Desmoplastic small round cell tumour	11 (0.0)	4 (0.0)	NA	NA
4.13 Chordoma	30 (0.1)	26 (0.0)	1 (0.1)	1 (0.0)
4.14 Giant cell sarcoma	18 (0.0)	12 (0.0)	4 (0.3)	6 (0.2)
4.15 Other soft tissue sarcomas	113 (0.3)	144 (0.2)	12 (0.8)	13 (0.5)
4.16 Other bone tumours	52 (0.1)	66 (0.1)	3 (0.2)	2 (0.1)
**5. Blood and lymphatic vessel tumours**	**650 (1.6)**	**120 (0.2)**	**16 (1.1)**	**11 (0.4)**
**6. Nerve sheath tumours**	**123 (0.3)**	**105 (0.2)**	**5 (0.3)**	**14 (0.5)**
**7. Gonadal and related tumours**	**12 074 (30.3)**	**2249 (3.8)**	**55 (3.7)**	**124 (4.3)**
7.1 Testis	11 716 (29.4)	NA	52 (3.5)	NA
7.2 Ovary	NA	2034 (3.4)	NA	124 (4.3)
7.3 Germ cell and trophoblastic, CNS	108 (0.3)	19 (0.0)	NA	NA
7.4 Germ cell and trophoblastic excluding CNS, ovary, testis	250 (0.6)	189 (0.3)	3 (0.2)	NA
7.5 Non-germ cell specified tumours excluding CNS, ovary, testis	NA	2 (0.0)	NA	NA
7.6 Fibroepithelial including Brenner, excluding breast phyllodes	NA	5 (0.0)	NA	NA
**8. Melanoma, malignant**	**5895 (14.8)**	**11 052 (18.5)**	**133 (9.0)**	**224 (7.8)**
8.1 Superficial spreading/low cumulative sun damage melanoma	3729 (9.3)	7663 (12.9)	94 (6.4)	157 (5.5)
8.2 Nodular melanoma	662 (1.7)	918 (1.5)	10 (0.7)	24 (0.8)
8.3 Other malignant	1504 (3.8)	2471 (4.1)	29 (2.0)	43 (1.5)
**9. Carcinomas**	**7272 (18.2)**	**35 341 (59.3)**	**908 (61.7)**	**2125 (74.0)**
9.1 Thyroid carcinoma	820 (2.1)	2670 (4.5)	38 (2.6)	80 (2.8)
9.2 Other carcinoma of head and neck	1072 (2.7)	782 (1.3)	105 (7.1)	88 (3.1)
9.3 Carcinoma of gastrointestinal tract	2924 (7.3)	3048 (5.1)	320 (21.8)	346 (12.0)
9.3.1 Carcinoma of oesophagus	148 (0.4)	52 (0.1)	38 (2.6)	33 (1.1)
9.3.2 Carcinoma of stomach	384 (1.0)	339 (0.6)	47 (3.2)	31 (1.1)
9.3.3 Carcinoma of small intestine	68 (0.2)	72 (0.1)	14 (1.0)	14 (0.5)
9.3.4 Carcinoma of colon	1256 (3.1)	1550 (2.6)	77 (5.2)	120 (4.2)
9.3.5 Carcinoma of rectum	721 (1.8)	660 (1.1)	53 (3.6)	72 (2.5)
9.3.6 Carcinoma of anus	64 (0.2)	67 (0.1)	18 (1.2)	13 (0.5)
9.3.7 Carcinoma of liver and intrahepatic bile ducts (IBD)	77 (0.2)	79 (0.1)	15 (1.0)	7 (0.2)
9.3.8 Carcinoma of gallbladder and other extrahepatic biliary	72 (0.2)	61 (0.1)	10 (0.7)	8 (0.3)
9.3.9 Carcinoma of pancreas	126 (0.3)	162 (0.3)	45 (3.1)	48 (1.7)
9.3.10 Other carcinoma of gastrointestinal tract	8 (0.0)	6 (0.0)	3 (0.2)	NA
9.4 Carcinoma of lung, bronchus, and trachea	702 (1.8)	910 (1.5)	143 (9.7)	349 (12.1)
9.5 Carcinoma of skin (if collected)	473 (1.2)	541 (0.9)	98 (6.7)	124 (4.3)
9.6 Carcinoma of breast	36 (0.1)	19 572 (32.8)	6 (0.4)	772 (26.9)
9.7 Carcinoma of genital sites excluding ovary and testis	86 (0.2)	6919 (11.6)	78 (5.3)	230 (8.0)
9.7.1 Carcinoma of uterine cervix	NA	6266 (10.5)	NA	84 (2.9)
9.7.2 Corpus uteri	NA	307 (0.5)	NA	100 (3.5)
9.7.3 Carcinoma of vulva and vagina	NA	328 (0.6)	NA	36 (1.3)
9.7.4 Carcinoma of penis	66 (0.2)	NA	5 (0.3)	NA
9.7.5 Carcinoma of prostate	16 (0.0)	NA	72 (4.9)	NA
9.7.6 Other genital	4 (0.0)	18 (0.0)	1 (0.1)	10 (0.3)
9.8 Carcinoma of urinary tract	881 (2.2)	555 (0.9)	95 (6.5)	77 (2.7)
9.8.1 Carcinoma of kidney	593 (1.5)	382 (0.6)	57 (3.9)	39 (1.4)
9.8.2 Carcinoma of bladder	253 (0.6)	155 (0.3)	31 (2.1)	29 (1.0)
9.8.3 Other urinary	35 (0.1)	18 (0.0)	7 (0.5)	9 (0.3)
9.9 Other invasive carcinomas	278 (0.7)	344 (0.6)	25 (1.7)	59 (2.1)
**10. Miscellaneous specified neoplasms**	**102 (0.3)**	**137 (0.2)**	**7 (0.5)**	**11 (0.4)**
**11. Unspecified malignant neoplasms except CNS**	**85 (0.2)**	**51 (0.1)**	**23 (1.6)**	**23 (0.8)**

Cancer types are grouped according to the AYA-specific classification scheme developed by Barr and colleagues (2020).^35^

AYA, adolescent and young adult; CNS, central nervous system; FIGO, Fédération Internationale de Gynécologie et d’Obstétrique; IQR, interquartile range; NA, not applicable; TNM, tumour–node–metastasis.

a Percentages might not add up to 100% due to rounding.

b Age at diagnosis of first and second primary cancer.

c Years of follow-up from 6-month survival until the date of second cancer diagnosis, death, loss to follow-up, or 31 December 2018, whichever came first.

### Overall SIRs and AERs

**Figure 1:**
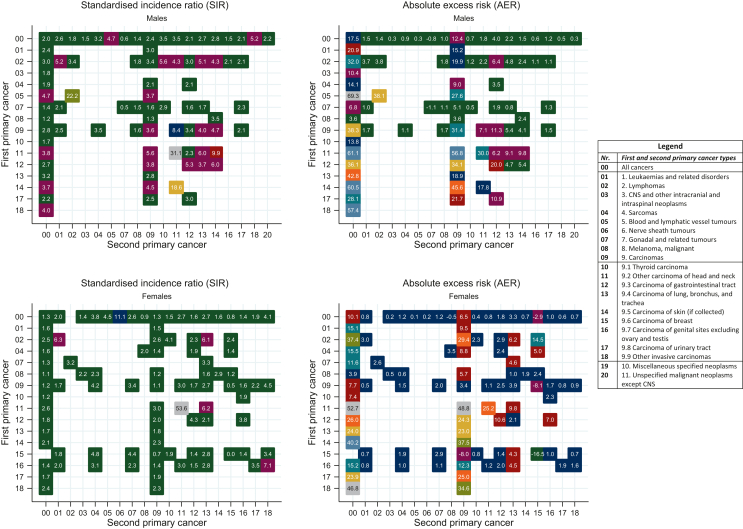
Standardised incidence ratios (SIRs) and absolute excess risks (AERs) of first and second primary malignant cancer combinations diagnosed among 6-month adolescent and young adult (AYA, aged 15-39 years) cancer survivors compared to the general population in the Netherlands between 1989 and 2018. Cancer types were grouped according to the AYA-specific classification scheme developed by Barr and colleagues (2020).^35^ Only cancer combinations with at least *n* = 10 observed second cancers and significant risk estimates are presented. CNS, central nervous system.

The overall risk of developing any second cancer after 25 years of follow-up was 2-fold higher in male survivors and 1.3-fold higher in female survivors compared to the general population. AERs of any second cancer were 17.5 and 10.1 per 10 000 person-years for male and female survivors, respectively (Figure 1, Supplementary Figure S3, available at https://doi.org/10.1016/j.esmoop.2023.102203, and Table 2). The overall higher cancer risk among AYA survivors maintained throughout the entire follow-up duration for all cancers combined (Supplementary Figure S4, available at https://doi.org/10.1016/j.esmoop.2023.102203). A closer look at survivors of specific cancer types showed that only male lymphoma and gastrointestinal tract carcinoma survivors had a higher second cancer risk throughout the entire follow-up period, whereas no such trend was observed among female survivors (Supplementary Table S2A, available at https://doi.org/10.1016/j.esmoop.2023.102203). A higher cancer risk compared to the general population was observed regardless of age at first cancer diagnosis, with SIRs peaking among those aged 15-19 years (3-fold risk for both sexes). AERs were highest among males aged 35-39 years at first cancer diagnosis, whereas this was the case for females aged 15-19 years. Cancer type-specific outcomes showed higher cancer risks among survivors of most of the 11 first primary cancer diagnostic main groups. SIRs in males ranged from 1.2 for melanoma survivors to 4.7 for blood and lymphatic vessel tumour survivors, whereas for the same cancers AERs ranged from 3.6 to 69.3 per 10 000 person-years. In females, SIRs ranged from 1.1 to 2.5 and AERs from 3.9 to 37.4 per 10 000 person-years for melanoma and lymphoma survivors, respectively. SIRs for the carcinoma diagnostic subgroups showed higher cancer risks after most first primary carcinomas among males, ranging from 1.7 to 4.0 for thyroid and other invasive carcinoma survivors, respectively. Meanwhile, female breast carcinoma survivors displayed a cancer risk comparable to the general population [SIR = 0.9 (95% CI 0.9-1.0)], whereas higher cancer risks among female carcinoma survivors were observed otherwise (Table 2).

**Table 2:** SIRs and AERs of any second primary malignant cancer diagnosis after first primary malignant cancer among 6-month AYA (aged 15-39 years) cancer survivors compared to the general population in the Netherlands

First primary cancers	Second primary cancer risk									
	Males					Females				
	Person-years	Obs/exp	SIR (95% CI)	AER per 10 000 person-years (95% CI)	*P* value	Person-years	Obs/exp	SIR (95% CI)	AER per 10 000 person-years (95% CI)	*P* value
**Total**	409 823.4	1431/714	2.0 (1.9-2.1)	17.5 (15.7-19.3)	0.000	636 375.6	2802/2161	1.3 (1.2-1.3)	10.1 (8.5-11.7)	0.000
**Age at diagnosis (years)**^a^										
15-19	33 883.8	54/20	2.7 (2.0-3.5)	10.1 (6.1-14.9)	0.000	28 664.6	76/22	3.4 (2.7-4.2)	18.7 (13.1-25.4)	0.000
20-24	56 864.2	112/48	2.3 (1.9-2.8)	11.2 (7.7-15.2)	0.000	50 657.5	147/68	2.1 (1.8-2.5)	15.5 (11.0-20.6)	0.000
25-29	84 904.9	210/103	2.0 (1.8-2.3)	12.6 (9.4-16.2)	0.000	95 277.8	342/206	1.7 (1.5-1.8)	14.3 (10.6-18.3)	0.000
30-34	107 315.6	380/192	2.0 (1.8-2.2)	17.5 (14.0-21.2)	0.000	179 803.1	723/577	1.3 (1.2-1.3)	8.1 (5.2-11.1)	0.000
35-39	126 854.8	675/351	1.9 (1.8-2.1)	25.5 (21.6-29.7)	0.000	281 972.5	1514/1287	1.2 (1.1-1.2)	8.1 (5.4-10.8)	0.000
**Tumour stage (TNM, FIGO, and Ann Arbor)**										
Stage I	199 269.4	553/361	1.5 (1.4-1.7)	9.7 (7.4-12.1)	0.000	327 333.7	1331/1152	1.2 (1.1-1.2)	5.5 (3.3-7.7)	0.000
Stage II	72 294.1	264/128	2.1 (1.8-2.3)	18.9 (14.6-23.5)	0.000	170 321.4	770/598	1.3 (1.2-1.4)	10.1 (6.9-13.4)	0.000
Stage III	42 215.6	186/67	2.8 (2.4-3.2)	28.2 (22.1-35.0)	0.000	44 945.7	241/137	1.8 (1.5-2.0)	23.2 (16.6-30.4)	0.000
Stage IV	21 923.5	127/38	3.3 (2.8-3.9)	40.5 (30.8-51.5)	0.000	15 758.4	95/42	2.3 (1.8-2.8)	33.6 (22.1-47.0)	0.000
Other/unknown	74 120.8	301/121	2.5 (2.2-2.8)	24.3 (19.8-29.1)	0.000	78 016.4	365/231	1.6 (1.4-1.8)	17.2 (12.5-22.2)	0.000
**Cancer types**										
**1. Leukaemia and related disorders**	**18** **388.6**	**67/28.5**	**2.4 (1.8-3.0)**	**20.9 (12.7-30.8)**	**0.000**	**15** **915.8**	**64/40**	**1.6 (1.2-2.0)**	**15.1 (5.8-26.2)**	**0.001**
1.1 Acute lymphoblastic leukaemia	4910.6	14/5	2.6 (1.4-4.3)	17.4 (4.5-36.7)	0.003	3091.0	10/5	2.0 (1.0-3.7)	16.2 (−0.6 to 43.4)	0.063
1.2 Acute myeloid leukaemia	4529.4	17/7	2.5 (1.5-4.0)	22.7 (7.0-45.2)	0.001	5336.0	27/15	1.8 (1.2-2.7)	22.8 (5.6-45.8)	0.006
1.2.1 Acute promyelocytic leukaemia	NA	NA	NA	NA	NA	1174.9	10/4	2.8 (1.3-5.1)	54.6 (10.3-126)	0.008
1.2.2 Other acute myeloid leukaemia	3758.1	12/6	2.1 (1.1-3.7)	16.9 (1.4-40.7)	0.027	4161.1	17/11	1.5 (0.9-2.4)	13.9 (−3.2 to 38.4)	0.130
1.3 Chronic myeloid leukaemia	3598.6	11/5	2.0 (1.0-3.6)	15.5 (0.2-39.6)	0.046	2583.1	10/7	1.4 (0.7-2.5)	10.6 (−9.5 to 43.1)	0.391
1.4 Chronic lymphocytic leukaemia	1197.7	13/3	4.4 (2.3-7.4)	83.6 (32.9-160.7)	0.000	NA	NA	NA	NA	NA
**2. Lymphomas**	**70** **258.5**	**339/114.1**	**3.0 (2.7-3.3)**	**32.0 (27.0-37.4)**	**0.000**	**52** **629.5**	**326/129.3**	**2.5 (2.3-2.8)**	**37.4 (30.8-44.5)**	**0.000**
2.1 Non-Hodgkin’s lymphomas	27 550.9	132/48	2.7 (2.3-3.2)	30.4 (22.6-39.3)	0.000	17 396.8	101/52	1.9 (1.6-2.3)	28.0 (17.2-40.5)	0.000
2.1.3 Diffuse large B-cell (DLBCL)	11 080.0	60/20	3.0 (2.3-3.8)	36.0 (23.1-51.5)	0.000	6571.1	32/19	1.7 (1.1-2.4)	19.7 (4.3-39.8)	0.008
2.1.5 Anaplastic T-cell and null-cell excluding NK/T-cell	4242.9	18/7	2.4 (1.4-3.9)	25.0 (7.8-49.7)	0.001	2809.3	16/8	2.1 (1.2-3.4)	29.8 (5.4-65.4)	0.011
2.1.6 Follicular	4838.4	29/10	3.0 (2.0-4.2)	39.6 (19.8-65.8)	0.000	3701.0	25/14	1.8 (1.2-2.6)	29.6 (5.8-61.8)	0.010
2.1.9 Other non-Hodgkin’s lymphoma NOS	2025.3	12/4	2.9 (1.5-5.1)	39.0 (10.4-83.2)	0.002	NA	NA	NA	NA	NA
2.2 Hodgkin’s lymphoma	38 953.5	191/58	3.3 (2.9-3.8)	34.2 (27.5-41.7)	0.000	33 078.6	214/69	3.1 (2.7-3.5)	43.8 (35.4-53.1)	0.000
2.2.1 Hodgkin’s NLP	2749.4	11/4	3.0 (1.5-5.4)	26.7 (6.7-58.3)	0.003	NA	NA	NA	NA	NA
2.2.2 Hodgkin’s classic, other	36 204.1	180/54	3.3 (2.9-3.8)	34.8 (27.8-42.6)	0.000	32 320.5	211/68	3.1 (2.7-3.6)	44.4 (35.9-53.8)	0.000
**3. CNS and other intracranial and intraspinal neoplasms**	**17** **903.2**	**42/23.4**	**1.8 (1.3-2.4)**	**10.4 (3.8-18.6)**	**0.001**	**13** **410.8**	**39/30.1**	**1.3 (0.9-1.8)**	**6.6 (−1.8 to 17.3)**	**0.135**
3.1 Astroglial and related neoplasms	15 577.6	39/21	1.9 (1.3-2.6)	11.7 (4.4-20.9)	0.000	11 588.0	34/26	1.3 (0.9-1.8)	6.7 (−2.3 to 18.4)	0.163
3.1.4 Other astrocytoma/astroglial neoplasms	8523.5	20/10	1.9 (1.2-3.0)	11.4 (2.3-24.2)	0.009	6956.7	20/15	1.3 (0.8-2.0)	7.0 (−4.2 to 22.7)	0.262
3.1.4.3 Other astrocytoma/astroglial, invasive	7240.4	18/9	2.0 (1.2-3.2)	12.6 (2.5-27.1)	0.009	6164.4	16/14	1.2 (0.7-1.9)	3.8 (−7.3 to 20.0)	0.597
**4. Sarcomas**	**25** **271.0**	**77/41.4**	**1.9 (1.5-2.3)**	**14.1 (7.7-21.7)**	**0.000**	**24** **366.8**	**105/67.1**	**1.6 (1.3-1.9)**	**15.5 (7.7-24.6)**	**0.000**
4.1 Osteosarcoma	NA	NA	NA	NA	NA	2119.3	11/3	3.4 (1.7-6.1)	36.7 (10.7-77.6)	0.001
4.2 Chondrosarcoma	NA	NA	NA	NA	NA	3697.3	15/10	1.5 (0.8-2.5)	13.5 (−4.3 to 39.9)	0.166
4.4 Fibromatous neoplasms	7712.7	28/15	1.9 (1.3-2.8)	17.4 (5.2-33.5)	0.002	7953.1	24/25	1.0 (0.6-1.5)	−0.7 (−11.6 to 14.0)	1.000
4.4.3 Other fibromatous neoplasms	6330.7	21/12	1.7 (1.1-2.6)	14.0 (1.4-31.5)	0.026	6630.4	19/20	0.9 (0.6-1.5)	−2.2 (−13.6 to 13.9)	0.863
4.5 Liposarcoma	2340.2	10/5	1.9 (0.9-3.4)	19.9 (−2.3 to 55.7)	0.092	2291.3	18/8	2.3 (1.4-3.7)	45.1 (13.1-90.7)	0.002
**5. Blood and lymphatic vessel tumours**	**4761.3**	**42/9**	**4.7 (3.4-6.3)**	**69.3 (44.6-100.3)**	**0.000**	**NA**	**NA**	**NA**	**NA**	**NA**
5.2 Malignant blood and lymphatic vessel tumours, all sites	4761.3	42/9	4.7 (3.4-6.3)	69.3 (44.6-100.3)	0.000	NA	NA	NA	NA	NA
5.2.1 Kaposi sarcoma	4357.2	40/9	4.7 (3.3-6.4)	72.2 (46.0-105.4)	0.000	NA	NA	NA	NA	NA
**7. Gonadal and related tumours**	**140** **853.4**	**324/228.8**	**1.4 (1.3-1.6)**	**6.8 (4.3-9.4)**	**0.000**	**28** **838.4**	**131/97.6**	**1.3 (1.1-1.6)**	**11.6 (4.1-20.0)**	**0.001**
7.1 Testis	137 018.9	297/224	1.3 (1.2-1.5)	5.3 (2.9-7.9)	0.000	NA	NA	NA	NA	NA
7.1.1 Germ cell and trophoblastic	136 746.6	295/224	1.3 (1.2-1.5)	5.2 (2.8-7.8)	0.000	NA	NA	NA	NA	NA
7.1.1.1 Seminoma	63 355.5	155/121	1.3 (1.1-1.5)	5.3 (1.6-9.5)	0.004	NA	NA	NA	NA	NA
7.1.1.2 Embryonal carcinoma	20 486.2	34/29	1.2 (0.8-1.6)	2.4 (−2.7 to 9.0)	0.404	NA	NA	NA	NA	NA
7.1.1.4 Teratoma	22 978.6	48/35	1.4 (1.0-1.8)	5.5 (0.0-12.3)	0.051	NA	NA	NA	NA	NA
7.1.1.5 Mixed germ cell	19 848.5	35/24	1.4 (1.0-2.0)	5.3 (−0.1 to 12.2)	0.053	NA	NA	NA	NA	NA
7.1.1.6 Choriocarcinoma and other trophoblastic	6135.7	13/8	1.6 (0.9-2.8)	8.2 (−1.7 to 23.2)	0.127	NA	NA	NA	NA	NA
7.2 Ovary	NA	NA	NA	NA	NA	26 073.3	120/90	1.3 (1.1-1.6)	11.7 (3.8-20.7)	0.002
7.2.1 Germ cell and trophoblastic	NA	NA	NA	NA	NA	4168.6	16/9	1.8 (1.0-2.9)	16.7 (0.2-40.6)	0.046
7.2.2 Non-germ cell	NA	NA	NA	NA	NA	21 904.7	104/81	1.3 (1.1-1.6)	10.7 (2.0-20.8)	0.013
7.2.2.1 Carcinoma	NA	NA	NA	NA	NA	21 071.5	100/77	1.3 (1.1-1.6)	10.8 (2.0-21.1)	0.014
7.2.2.1.1 Adenocarcinoma	NA	NA	NA	NA	NA	20 651.3	99/76	1.3 (1.1-1.6)	11.2 (2.2-21.6)	0.013
7.2.2.1.1.2 Cystadenocarcinoma	NA	NA	NA	NA	NA	16 654.7	81/60	1.3 (1.1-1.7)	12.3 (2.3-24.2)	0.013
7.4 Germ cell and trophoblastic excluding CNS, ovary, testis	2660.0	22/4	5.7 (3.6-8.7)	68.2 (37.4-110.8)	0.000	2445.3	11/8	1.5 (0.7-2.6)	14.0 (−8.5 to 49.5)	0.288
7.4.1 Germ cell tumours including non-gestational trophoblastic tumours	1839.9	13/3	4.7 (2.5-8.0)	55.6 (22.6-105.8)	0.000	NA	NA	NA	NA	NA
7.4.2 Gestational trophoblastic tumours	NA	NA	NA	NA	NA	2374.7	10/7	1.4 (0.6-2.5)	11.0 (−10.9 to 46.3)	0.423
**8. Melanoma, malignant**	**65** **893.9**	**151/127.6**	**1.2 (1.0-1.4)**	**3.6 (0.0-7.5)**	**0.047**	**134** **464.5**	**479/425.9**	**1.1 (1.0-1.2)**	**3.9 (0.8-7.3)**	**0.012**
8.1 Superficial spreading/low cumulative sun damage melanoma	40 623.2	96/76	1.3 (1.0-1.5)	4.8 (0.3-10.0)	0.034	88 296.6	301/273	1.1 (1.0-1.2)	3.1 (−0.6 to 7.2)	0.104
8.2 Nodular melanoma	NA	NA	NA	NA	NA	11 430.6	49/34	1.4 (1.1-1.9)	12.8 (1.6-26.6)	0.022
8.3 Other malignant	18 193.3	48/37	1.3 (0.9-1.7)	5.8 (−1.1 to 14.4)	0.110	34 737.2	129/118	1.1 (0.9-1.3)	3.1 (−3.0 to 10.1)	0.343
**9. Carcinomas**	**64** **129.3**	**383/137.5**	**2.8 (2.5-3.1)**	**38.3 (32.4-44.6)**	**0.000**	**362** **967.1**	**1638/1359**	**1.2 (1.1-1.3)**	**7.7 (5.5-9.9)**	**0.000**
9.1 Thyroid carcinoma	10 011.7	33/19	1.7 (1.2-2.4)	13.8 (3.5-27.1)	0.005	32 240.4	122/98	1.2 (1.0-1.5)	7.4 (1.0-14.8)	0.022
9.1.1 Medullary	NA	NA	NA	NA	NA	1573.5	10/4	2.3 (1.1-4.3)	36.2 (3.1-89.5)	0.026
9.1.3 Papillary	5611.3	16/11	1.5 (0.8-2.4)	9.3 (−2.9 to 27.1)	0.161	16 857.0	48/49	1.0 (0.7-1.3)	−0.6 (−8.1 to 8.6)	0.953
9.1.4 Follicular	NA	NA	NA	NA	NA	5270.5	25/16	1.5 (1.0-2.3)	16.4 (−0.3 to 39.0)	0.055
9.1.5 Papillary with follicular variant	NA	NA	NA	NA	NA	7884.3	37/26	1.4 (1.0-1.9)	13.5 (−0.4 to 31.2)	0.058
9.2 Other carcinoma of head and neck	10 973.2	91/24	3.8 (3.1-4.7)	61.1 (45.0-80.0)	0.000	8580.6	73/28	2.6 (2.1-3.3)	52.7 (34.3-74.6)	0.000
9.2.1 Nasopharyngeal carcinoma	1452.8	10/2	4.7 (2.2-8.6)	54.1 (18.2-111.8)	0.000	NA	NA	NA	NA	NA
9.2.2 Oral cavity, lip, and pharynx	5682.9	59/13	4.5 (3.4-5.8)	80.6 (55.8-110.7)	0.000	3695.3	41/13	3.2 (2.3-4.4)	76.8 (45.4-116.3)	0.000
9.2.2.1 Oral cavity, lip, and pharynx, squamous	4473.1	55/11	5.2 (3.9-6.7)	99.2 (68.9-136.3)	0.000	2428.0	37/9	4.2 (3.0-5.8)	116.1 (71.0-173.7)	0.000
9.2.3 Salivary gland	NA	NA	NA	NA	NA	2935.3	15/8	1.8 (1.0-2.9)	22.4 (−0.1 to 55.6)	0.051
9.2.3.2 Salivary gland, other malignant	NA	NA	NA	NA	NA	1995.2	12/6	2.0 (1.0-3.4)	29.3 (0.2-74.2)	0.048
9.2.4 Other carcinoma of head and neck	1928.7	19/5	3.6 (2.2-5.7)	71.4 (32.2-126.7)	0.000	1033.1	13/4	3.3 (1.8-5.7)	87.9 (29.1-177.2)	0.000
9.3 Carcinoma of gastrointestinal tract	23 162.1	133/49	2.7 (2.3-3.2)	36.1 (26.7-46.7)	0.000	25 453.2	149/83	1.8 (1.5-2.1)	26.0 (17.0-36.2)	0.000
9.3.2 Carcinoma of stomach	2117.7	11/5	2.2 (1.1-3.9)	28.1 (2.1-69.1)	0.029	1635.3	10/5	1.9 (0.9-3.5)	28.6 (−3.2 to 79.9)	0.090
9.3.4 Carcinoma of colon	12 466.4	73/26	2.8 (2.2-3.5)	37.5 (24.8-52.6)	0.000	15 253.2	85/48	1.8 (1.4-2.2)	24.3 (13.1-37.5)	0.000
9.3.4.1 Appendix	NA	NA	NA	NA	NA	7015.4	15/17	0.9 (0.5-1.4)	−3.0 (−12.4 to 10.9)	0.726
9.3.4.1.1 NET	NA	NA	NA	NA	NA	6476.1	14/15	0.9 (0.5-1.5)	−1.9 (−11.7 to 12.7)	0.879
9.3.4.2 Colon excluding appendix	8820.8	66/21	3.1 (2.4-4.0)	50.9 (33.9-71.3)	0.000	8237.8	70/31	2.3 (1.8-2.9)	47.6 (28.9-70.0)	0.000
9.3.4.2.2 Colon excluding appendix, adenocarcinoma	8604.5	64/21	3.1 (2.4-3.9)	50.3 (33.2-70.9)	0.000	8001.3	68/30	2.3 (1.7-2.9)	47.3 (28.3-70.0)	0.000
9.3.5 Carcinoma of rectum	5748.4	28/13	2.2 (1.5-3.2)	26.9 (10.6-48.6)	0.000	5614.0	30/20	1.5 (1.0-2.1)	17.3 (−0.1 to 40.1)	0.052
9.3.5.2 Rectum, adenocarcinoma	5096.1	26/11	2.3 (1.5-3.4)	28.8 (11.1-52.6)	0.000	4782.1	25/18	1.4 (0.9-2.1)	14.9 (−3.5 to 39.8)	0.128
9.4 Carcinoma of lung, bronchus, and trachea	3364.0	21/7	3.2 (2.0-4.9)	42.8 (19.0-75.8)	0.000	5374.9	32/19	1.7 (1.1-2.4)	24.0 (5.2-48.6)	0.008
9.4.2 Non-small-cell carcinoma	3196.8	17/6	2.7 (1.6-4.3)	33.5 (11.3-65.5)	0.001	5240.1	31/19	1.7 (1.1-2.4)	23.7 (4.7-48.5)	0.010
9.4.2.1 Non-small-cell, adenocarcinoma	NA	NA	NA	NA	NA	1277.5	14/4	3.1 (1.7-5.2)	74.5 (24.8-148.8)	0.000
9.5 Carcinoma of skin (if collected)	5306.4	44/12	3.7 (2.7-5.0)	60.5 (37.8-88.9)	0.000	6179.0	48/23	2.1 (1.5-2.8)	40.2 (19.8-65.5)	0.000
9.6 Carcinoma of breast	NA	NA	NA	NA	NA	194 682.4	716/758	0.9 (0.9-1.0)	−2.2 (−4.8 to 0.6)	0.130
9.6.1 Breast, infiltrating duct	NA	NA	NA	NA	NA	154 044.4	549/588	0.9 (0.9-1.0)	−2.5 (−5.4 to 0.6)	0.114
9.6.2 Breast, adenocarcinoma	NA	NA	NA	NA	NA	5017.5	20/22	0.9 (0.6-1.4)	−3.6 (−19.2 to 18.1)	0.802
9.6.3 Breast, lobular	NA	NA	NA	NA	NA	17 439.9	75/74	1.0 (0.8-1.3)	0.8 (−8.4 to 11.7)	0.902
9.6.5 Breast, medullary	NA	NA	NA	NA	NA	9447.1	32/40	0.8 (0.5-1.1)	−8.6 (−19.3 to 5.4)	0.224
9.6.10 Breast, other	NA	NA	NA	NA	NA	6131.1	24/26	0.9 (0.6-1.4)	−2.9 (−16.9 to 16.2)	0.830
9.7 Carcinoma of genital sites excluding ovary and testis	NA	NA	NA	NA	NA	82 999.0	449/323	1.4 (1.3-1.5)	15.2 (10.3-20.4)	0.000
9.7.1 Carcinoma of uterine cervix	NA	NA	NA	NA	NA	75 551.2	396/292	1.4 (1.2-1.5)	13.8 (8.7-19.2)	0.000
9.7.1.1 Cervix, squamous	NA	NA	NA	NA	NA	53 426.2	269/207	1.3 (1.2-1.5)	11.7 (5.8-18.1)	0.000
9.7.1.2 Cervix, adenosquamous	NA	NA	NA	NA	NA	2521.0	14/10	1.5 (0.8-2.5)	17.8 (−7.4 to 55.4)	0.206
9.7.1.3 Cervix, adenocarcinoma	NA	NA	NA	NA	NA	14 344.5	90/53	1.7 (1.4-2.1)	26.1 (13.8-40.4)	0.000
9.7.1.4 Cervix, other	NA	NA	NA	NA	NA	5259.4	23/23	1.0 (0.6-1.5)	−0.5 (−16.6 to 21.3)	1.000
9.7.2 Corpus uteri	NA	NA	NA	NA	NA	3719.6	18/16	1.1 (0.7-1.7)	4.3 (−15.4 to 32.4)	0.759
9.7.2.1 Corpus uteri, adenocarcinoma	NA	NA	NA	NA	NA	2834.4	15/13	1.2 (0.7-2.0)	8.4 (−14.9 to 42.8)	0.571
9.7.2.1.2 Corpus uteri, other adenocarcinoma	NA	NA	NA	NA	NA	1842.5	14/9	1.5 (0.8-2.5)	24.9 (−9.6 to 76.4)	0.193
9.7.3 Carcinoma of vulva and vagina	NA	NA	NA	NA	NA	3548.1	35/14	2.5 (1.7-3.5)	59.0 (29.0-97.5)	0.000
9.8 Carcinoma of urinary tract	8612.3	45/21	2.2 (1.6-2.9)	28.1 (14.0-45.8)	0.000	5489.5	33/20	1.7 (1.1-2.3)	23.9 (5.2-48.2)	0.009
9.8.1 Carcinoma of kidney	5658.7	27/13	2.0 (1.3-3.0)	24.4 (8.1-46.1)	0.001	3986.0	17/14	1.2 (0.7-1.9)	7.0 (−10.8 to 32.7)	0.522
9.8.1.1 Kidney, adenocarcinoma	5612.6	27/13	2.1 (1.4-3.0)	24.7 (8.3-46.6)	0.001	3986.0	17/14	1.2 (0.7-1.9)	7.0 (−10.8 to 32.7)	0.522
9.8.1.1.1 Kidney, renal cell	5218.1	23/12	1.9 (1.2-2.8)	20.6 (4.4-42.6)	0.008	3648.1	16/13	1.2 (0.7-2.0)	7.8 (−11.0 to 35.2)	0.499
9.8.2 Carcinoma of bladder	2558.8	17/6	2.6 (1.5-4.2)	41.2 (13.5-81.1)	0.001	1268.5	13/5	2.8 (1.5-4.7)	65.5 (17.6-138.3)	0.002
9.8.2.1 Urinary bladder, transitional cell carcinoma	2341.9	13/6	2.1 (1.1-3.7)	29.6 (3.7-69.1)	0.019	995.8	11/4	3.1 (1.5-5.5)	74.4 (19.1-161.6)	0.002
9.9 Other invasive carcinomas	1434.8	11/3	4.0 (2.0-7.1)	57.4 (19.0-117.9)	0.000	1968.2	16/7	2.4 (1.3-3.8)	46.8 (12.0-97.5)	0.004

Cancer types are grouped according to the AYA-specific classification scheme developed by Barr and colleagues (2020).^35^ Cancer combinations with less than *n* = 10 observed second cancers were excluded from the analyses.

AERs, absolute excess risks; AYA, adolescent and young adult; CNS, central nervous system; Exp, expected number of second cancers; FIGO, Fédération Internationale de Gynécologie et d'Obstétrique; NA, not applicable; NET, neuroendocrine tumour; NK, natural killer; NLP, nodular lymphocyte predominant; NOS, not otherwise specified; Obs, observed number of second cancers; TNM, tumour–node–metastasis.

a Age at diagnosis of first primary cancer.

### Second cancer type-specific SIRs and AERs

A higher cancer risk compared to the general population was observed for most second cancer types when grouping all first cancer survivors together. In general, male survivors had a lower second testicular cancer risk [SIR = 0.6 (95% CI 0.5-0.8)], whereas a lower risk for second melanomas [SIR = 0.9 (95% CI 0.8-1.0)] and breast carcinomas [SIR = 0.8 (95% CI 0.7-0.9)] was found among female survivors (Supplementary Table S3A, available at https://doi.org/10.1016/j.esmoop.2023.102203). SIRs and AERs for a distinct range of first and second cancer combinations are displayed in Figure 1 (significant outcomes only) and Supplementary Tables S4A-S20A, available at https://doi.org/10.1016/j.esmoop.2023.102203. Second primary carcinomas were observed after nearly all first primary cancer types and most often included the gastrointestinal and lung carcinoma diagnostic subgroups. Second skin (males) and breast carcinomas (females) occurred after five of eight first primary cancer types (Figure 1 and Supplementary Tables S4A-S20A, available at https://doi.org/10.1016/j.esmoop.2023.102203). Male gonadal and related tumour survivors had a lower second primary testicular cancer risk [SIR = 0.4 (95% CI 0.2-0.8)], whereas female breast carcinoma survivors had a lower risk of developing another breast carcinoma compared to the general population [SIR = 0.0 (95% CI 0.0-0.1)]. Comparable cancer risks between AYA cancer survivors and the general population were observed for several first and second primary cancer combinations, including (non-Hodgkin’s) lymphoma risk among (breast) carcinoma and melanoma survivors (both sexes) and male gonadal and related tumour survivors. In females, comparable breast cancer risk was observed in first primary leukaemia [SIR = 0.9 (95% CI 0.5-1.4)], gonadal and related tumour [SIR = 0.9 (95% CI 0.6-1.2)], and central nervous system and other intracranial and intraspinal neoplasm survivors [SIR = 1.0 (95% CI 0.5-1.7)]. Likewise, breast cancer risk was comparable after most carcinoma diagnostic subgroups (Supplementary Tables S4A-S20A, available at https://doi.org/10.1016/j.esmoop.2023.102203).

In males, AERs were the highest for second primary (non-Hodgkin’s) lymphomas among blood and lymphatic vessel tumour survivors [AER = 38.1 (95% CI 22.2-60.5)], who also had the highest AERs for developing second primary carcinomas, together with first primary carcinoma survivors [AER = 31.4 (95% CI 26.4-36.8)]. More distinctly, highest AERs after first primary carcinoma were observed for second primary carcinomas of the skin, gastrointestinal tract, other head and neck and lung, bronchus, and trachea. Highest AERs in females were observed for second primary carcinomas after first primary lymphoma [AER = 29.4 (95% CI 23.7-35.7)] and leukaemia [AER = 9.5 (95% CI 1.9-19.1)]. In female lymphoma survivors, this mainly included carcinomas of the breast, thyroid, gastrointestinal tract, other head and lung, bronchus, and trachea (Supplementary Tables S4A-S20A, available at https://doi.org/10.1016/j.esmoop.2023.102203).

### Cumulative incidence

**Figure 2:**
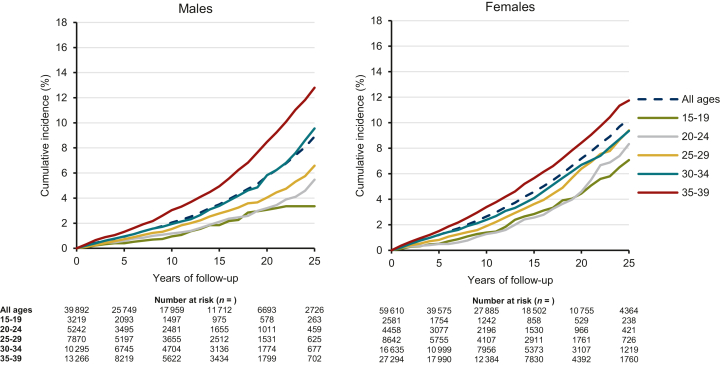
Cumulative incidence of any second primary cancer diagnosed up to 25 years after first primary cancer among 6-month adolescent and young adult (AYA, aged 15-39 years) cancer survivors in the Netherlands between 1989 and 2018. Outcomes are presented for males and females overall and by age group at first primary cancer diagnosis. Death of any cause was included as a competing risk event.

From 6 months after the date of first primary cancer diagnosis, the overall 5-year cumulative incidence of second cancer was 0.9% for male and 1.2% for female survivors and increased to 8.9% for male and 10.3% for female survivors at 25 years of follow-up (Figure 2 and Table 3). Age-specific cumulative incidences at 25 years of follow-up ranged from 3.4% among male and 7.1% among female survivors aged 15-19 years to 12.8% and 11.7% among male and female survivors aged 35-39 years, respectively (Figure 2 and Table 3). Five-year cumulative incidences of any second cancer remained below 8% for males and below 12% for females. At 25 years of follow-up, the cumulative incidence of second cancer was overall the highest for male lymphoma survivors (13.3%), followed by carcinoma survivors (11.5%), with the highest point estimates observed after first primary skin and other head and neck carcinomas. Among females, the highest 25-year cumulative incidence was observed among first primary lymphoma survivors (18.9%). Among female carcinoma survivors, highest cumulative incidences were observed after skin and other head and neck carcinomas (Table 3, and Supplementary Figures S5 and S6, available at https://doi.org/10.1016/j.esmoop.2023.102203).

**Table 3:** Cumulative incidence of any second primary cancer up to 25 years after first primary cancer among 6-month AYA (aged 15-39 years) cancer survivors in the Netherlands between 1989 and 2018

	Cumulative incidence (95% CI)							
	Males				Females			
	5	10	20	25	5	10	20	25
**Total**	0.9 (0.8-1.0)	2.1 (1.9-2.2)	5.8 (5.4-6.1)	8.9 (8.4-9.5)	1.2 (1.1-1.3)	2.7 (2.5-2.8)	7.2 (6.9-7.5)	10.3 (9.8-10.7)
**Age at diagnosis (years)**^a^								
15-19	0.4 (0.2-0.7)	1.0 (0.6-1.4)	3.1 (2.3-4.1)	3.4 (2.5-4.4)	0.5 (0.3-0.9)	1.4 (0.9-2.0)	4.4 (3.4-5.7)	7.1 (5.4-9.1)
20-24	0.6 (0.4-0.8)	1.2 (0.9-1.5)	3.2 (2.5-4.0)	5.5 (4.3-6.8)	0.5 (0.3-0.7)	1.3 (0.9-1.7)	4.6 (3.7-5.6)	8.3 (6.9-9.9)
25-29	0.7 (0.5-0.9)	1.5 (1.2-1.9)	4.0 (3.4-4.7)	6.6 (5.6-7.7)	0.8 (0.6-1.0)	1.9 (1.6-2.3)	6.4 (5.7-7.2)	9.4 (8.3-10.6)
30-34	0.9 (0.8-1.2)	1.9 (1.7-2.3)	5.8 (5.2-6.5)	9.6 (8.4-10.7)	1.2 (1.1-1.4)	2.4 (2.1-2.7)	6.7 (6.2-7.3)	9.4 (8.6-10.2)
35-39	1.3 (1.1-1.5)	3.0 (2.7-3.4)	8.5 (7.8-9.2)	12.8 (11.7-14.0)	1.5 (1.4-1.7)	3.4 (3.2-3.6)	8.4 (8.0-8.9)	11.7 (11.1-12.4)
**Tumour stage (TNM, FIGO, and Ann Arbor)**								
Stage I	0.7 (0.6-0.9)	1.8 (1.6-2.0)	5.5 (5.0-6.1)	9.0 (8.1-9.9)	1.3 (1.1-1.4)	2.9 (2.7-3.1)	7.6 (7.2-8.1)	10.8 (10.1-11.4)
Stage II	1.1 (0.8-1.4)	2.3 (1.9-2.7)	6.3 (5.4-7.2)	10.0 (8.6-11.4)	1.3 (1.1-1.5)	2.7 (2.4-2.9)	7.3 (6.7-7.9)	10.4 (9.6-11.3)
Stage III	1.2 (0.9-1.6)	2.3 (1.8-2.8)	7.2 (6.1-8.4)	10.6 (8.7-12.6)	1.2 (0.9-1.5)	2.3 (1.9-2.8)	7.8 (6.7-8.9)	10.7 (9.1-12.4)
Stage IV	1.3 (1.0-1.8)	2.6 (2.0-3.2)	5.7 (4.7-6.9)	7.6 (6.2-9.1)	1.0 (0.7-1.4)	1.9 (1.4-2.5)	4.5 (3.5-5.6)	7.8 (6.0-9.8)
Other/unknown	0.9 (0.7-1.2)	2.1 (1.8-2.5)	5.0 (4.4-5.6)	7.6 (6.6-8.6)	1.1 (0.9-1.4)	2.5 (2.1-2.9)	6.6 (5.9-7.4)	9.7 (8.6-10.8)
**Cancer types**								
**1. Leukaemia and related disorders**	**0.6 (0.4-1.1)**	**1.9 (1.3-2.6)**	**5.1 (3.8-6.7)**	**8.7 (6.2-11.7)**	**1.3 (0.9-2.0)**	**2.4 (1.7-3.3)**	**5.4 (3.9-7.2)**	**9.6 (6.7-13.0)**
1.1 Acute lymphoblastic leukaemia	0.7 (0.2-1.7)	1.4 (0.6-2.7)	4.3 (2.3-7.1)	4.3 (2.3-7.1)	0.8 (0.2-2.2)	1.2 (0.4-3.1)	4.3 (1.9-8.4)	6.8 (3.2-12.2)
1.2 Acute myeloid leukaemia	0.4 (0.1-1.2)	1.7 (0.8-3.3)	4.4 (2.4-7.4)	6.5 (3.5-10.9)	1.7 (0.9-3.0)	2.5 (1.5-4.1)	4.9 (3.0-7.6)	9.4 (5.6-14.5)
1.2.1 Acute promyelocytic leukaemia	NA	NA	NA	NA	2.2 (0.4-6.8)	4.8 (1.5-11.0)	10.4 (3.7-21.3)	31.2 (13.8-50.5)
1.2.2 Other acute myeloid leukaemia	0.4 (0.1-1.4)	1.4 (0.6-3.0)	3.4 (1.6-6.2)	4.6 (2.1-8.7)	1.7 (0.8-3.0)	2.2 (1.1-3.7)	3.9 (2.2-6.5)	4.7 (2.5-7.7)
1.3 Chronic myeloid leukaemia	1.1 (0.4-2.7)	1.7 (0.7-3.6)	5.2 (2.2-10.1)	10.4 (3.0-23.3)	0.8 (0.2-2.8)	3.1 (1.3-6.3)	4.8 (2.2-9.0)	10.5 (4.0-20.7)
1.4 Chronic lymphocytic leukaemia	1.8 (0.4-5.8)	6.7 (2.7-13.1)	12.0 (5.7-20.9)	25.3 (11.7-41.5)	NA	NA	NA	NA
**2. Lymphomas**	**1.0 (0.7-1.3)**	**2.4 (2.0-2.9)**	**8.6 (7.6-9.7)**	**13.3 (11.7-14.9)**	**0.9 (0.7-1.3)**	**2.8 (2.3-3.4)**	**11.7 (10.3-13.1)**	**18.9 (16.7-21.2)**
2.1 Non-Hodgkin’s lymphomas	1.0 (0.7-1.5)	2.4 (1.8-3.2)	9.0 (7.4-10.8)	12.2 (9.9-14.7)	1.2 (0.7-1.8)	3.3 (2.4-4.4)	9.1 (7.2-11.3)	16.7 (13.1-20.7)
2.1.3 Diffuse large B-cell (DLBCL)	1.3 (0.7-2.2)	2.9 (1.9-4.2)	9.5 (7.0-12.4)	13.8 (10.0-18.2)	0.8 (0.3-1.8)	3.0 (1.7-4.8)	8.3 (5.5-12.0)	13.3 (8.3-19.5)
2.1.5 Anaplastic T-cell and null-cell excluding NK/T-cell	0.2 (0.0-1.3)	1.3 (0.4-3.2)	9.4 (5.4-14.7)	10.9 (6.2-16.9)	1.2 (0.3-3.2)	2.2 (0.8-4.9)	8.1 (4.0-14.0)	19.3 (10.3-30.3)
2.1.6 Follicular	0.8 (0.2-2.1)	2.8 (1.4-4.9)	11.0 (7.2-15.8)	15.3 (9.7-22.1)	1.5 (0.5-3.5)	3.1 (1.5-5.8)	9.8 (6.0-14.7)	18.8 (11.1-28.1)
2.1.9 Other non-Hodgkin’s lymphoma NOS	0.6 (0.1-3.1)	3.5 (1.3-7.5)	9.2 (4.8-15.3)	10.5 (5.7-17.1)	NA	NA	NA	NA
2.2 Hodgkin’s lymphoma	1.0 (0.7-1.4)	2.5 (2.0-3.2)	8.6 (7.3-10.1)	15.0 (12.7-17.4)	0.8 (0.5-1.2)	2.6 (2.0-3.4)	13.6 (11.7-15.7)	21.0 (18.1-24.0)
2.2.1 Hodgkin’s NLP	NA	1.3 (0.2-4.2)	10.3 (4.9-18.1)	16.8 (6.0-32.5)	NA	NA	NA	NA
2.2.2 Hodgkin’s classic, other	1.1 (0.7-1.5)	2.6 (2.0-3.4)	8.5 (7.1-10.1)	14.9 (12.6-17.4)	0.8 (0.5-1.2)	2.6 (2.0-3.4)	13.6 (11.7-15.7)	20.8 (18.0-23.9)
**3. CNS and other intracranial and intraspinal neoplasms**	**0.5 (0.3-0.8)**	**1.0 (0.6-1.5)**	**2.2 (1.5-3.0)**	**3.2 (2.2-4.4)**	**0.6 (0.3-1.1)**	**1.1 (0.6-1.7)**	**3.7 (2.6-5.1)**	**4.4 (3.1-6.0)**
3.1 Astroglial and related neoplasms	0.5 (0.3-0.9)	1.1 (0.7-1.7)	2.4 (1.6-3.3)	3.1 (2.1-4.3)	0.7 (0.4-1.3)	1.2 (0.7-1.9)	3.6 (2.4-5.0)	4.1 (2.8-5.8)
3.1.4 Other astrocytoma/astroglial neoplasms	0.4 (0.2-1.0)	1.2 (0.7-2.0)	1.9 (1.1-2.9)	2.7 (1.6-4.3)	0.6 (0.2-1.4)	1.1 (0.5-2.0)	3.6 (2.2-5.5)	4.1 (2.5-6.4)
3.1.4.3 Other astrocytoma/astroglial, invasive	0.5 (0.2-1.1)	1.2 (0.6-2.0)	2.0 (1.2-3.1)	2.6 (1.5-4.2)	0.5 (0.2-1.3)	1.0 (0.4-2.0)	3.1 (1.8-5.1)	3.7 (2.1-6.1)
**4. Sarcomas**	**1.0 (0.6-1.4)**	**1.7 (1.2-2.3)**	**4.1 (3.1-5.2)**	**6.9 (5.2-8.8)**	**0.9 (0.5-1.4)**	**2.9 (2.2-3.7)**	**7.2 (5.8-8.8)**	**9.6 (7.7-11.7)**
4.1 Osteosarcoma	NA	NA	NA	NA	1.0 (0.2-3.2)	3.4 (1.4-7.0)	7.5 (3.6-13.3)	10.6 (4.6-19.4)
4.2 Chondrosarcoma	NA	NA	NA	NA	1.3 (0.4-3.1)	4.5 (2.3-7.7)	9.4 (4.8-15.7)	9.4 (4.8-15.7)
4.4 Fibromatous neoplasms	1.2 (0.5-2.5)	2.4 (1.3-4.1)	5.4 (3.3-8.1)	11.0 (7.1-15.9)	0.6 (0.2-1.6)	2.4 (1.3-4.1)	6.4 (4.0-9.6)	8.1 (5.1-11.8)
4.4.3 Other fibromatous neoplasms	1.0 (0.3-2.4)	2.5 (1.2-4.5)	4.7 (2.6-7.6)	11.1 (6.6-17.0)	0.2 (0.0-1.2)	2.4 (1.2-4.4)	6.5 (3.8-10.2)	8.0 (4.8-12.2)
4.5 Liposarcoma	1.1 (0.2-3.6)	1.8 (0.5-4.7)	5.8 (2.5-11.2)	10.1 (4.4-18.7)	1.7 (0.5-4.5)	5.9 (2.9-10.4)	13.0 (7.8-19.5)	15.8 (8.9-24.4)
**5. Blood and lymphatic vessel tumours**	**3.2 (2.1-4.9)**	**5.1 (3.5-7.2)**	**8.6 (6.2-11.6)**	**10.9 (7.1-15.7)**	**NA**	**NA**	**NA**	**NA**
5.2 Malignant blood and lymphatic vessel tumours, all sites	3.2 (2.1-4.9)	5.1 (3.5-7.2)	8.6 (6.2-11.6)	10.9 (7.1-15.7)	NA	NA	NA	NA
5.2.1 Kaposi sarcoma	3.4 (2.1-5.1)	5.3 (3.6-7.5)	9.0 (6.4-12.2)	11.6 (7.4-16.8)	NA	NA	NA	NA
**7. Gonadal and related tumours**	**0.5 (0.4-0.7)**	**1.4 (1.2-1.7)**	**4.7 (4.2-5.4)**	**7.8 (6.9-8.9)**	**0.8 (0.5-1.3)**	**2.1 (1.5-2.8)**	**6.9 (5.7-8.3)**	**9.7 (8.0-11.5)**
7.1 Testis	0.5 (0.4-0.7)	1.2 (1.0-1.5)	4.5 (3.9-5.2)	7.6 (6.6-8.7)	NA	NA	NA	NA
7.1.1 Germ cell and trophoblastic	0.5 (0.4-0.7)	1.2 (1.0-1.5)	4.5 (3.9-5.1)	7.6 (6.6-8.7)	NA	NA	NA	NA
7.1.1.1 Seminoma	0.6 (0.4-0.8)	1.4 (1.1-1.8)	5.1 (4.2-6.1)	8.6 (7.1-10.4)	NA	NA	NA	NA
7.1.1.2 Embryonal carcinoma	0.4 (0.2-0.9)	0.9 (0.5-1.5)	3.5 (2.2-5.1)	6.4 (4.2-9.1)	NA	NA	NA	NA
7.1.1.4 Teratoma	0.5 (0.2-1.0)	1.2 (0.7-2.0)	3.3 (2.3-4.6)	6.5 (4.7-8.7)	NA	NA	NA	NA
7.1.1.5 Mixed germ cell	0.4 (0.2-0.8)	0.9 (0.5-1.5)	5.0 (3.3-7.2)	7.3 (4.2-11.5)	NA	NA	NA	NA
7.1.1.6 Choriocarcinoma and other trophoblastic	0.2 (0.0-1.3)	1.0 (0.3-2.4)	4.9 (2.5-8.5)	5.7 (3.0-9.8)	NA	NA	NA	NA
7.2 Ovary	NA	NA	NA	NA	0.8 (0.5-1.3)	2.1 (1.5-2.8)	6.7 (5.5-8.1)	9.7 (7.9-11.7)
7.2.1 Germ cell and trophoblastic	NA	NA	NA	NA	0.9 (0.3-2.4)	1.8 (0.7-3.9)	6.6 (3.4-11.2)	9.2 (4.8-15.2)
7.2.2 Non-germ cell	NA	NA	NA	NA	0.8 (0.5-1.4)	2.1 (1.5-2.9)	6.6 (5.3-8.1)	9.6 (7.7-11.7)
7.2.2.1 Carcinoma	NA	NA	NA	NA	0.8 (0.5-1.4)	2.2 (1.5-3.0)	6.7 (5.4-8.2)	9.4 (7.6-11.5)
7.2.2.1.1 Adenocarcinoma	NA	NA	NA	NA	0.9 (0.5-1.5)	2.2 (1.5-3.1)	6.8 (5.4-8.3)	9.6 (7.7-11.7)
7.2.2.1.1.2 Cystadenocarcinoma	NA	NA	NA	NA	0.8 (0.4-1.5)	2.0 (1.3-3.0)	7.1 (5.5-8.8)	9.8 (7.7-12.2)
7.4 Germ cell and trophoblastic excluding CNS, ovary, testis	1.8 (0.6-4.2)	6.5 (3.6-10.5)	11.6 (7.1-17.4)	16.4 (10.0-24.1)	0.5 (0.1-2.8)	2.5 (0.8-5.9)	11.0 (5.6-18.4)	11.0 (5.6-18.4)
7.4.1 Germ cell tumours including non-gestational Trophoblastic tumours	1.4 (0.3-4.5)	5.3 (2.4-10.1)	9.5 (4.6-16.5)	16.5 (8.5-26.9)	NA	NA	NA	NA
7.4.2 Gestational Trophoblastic tumours	NA	NA	NA	NA	0.6 (0.1-2.8)	2.6 (0.9-6.1)	10.2 (5.0-17.6)	10.2 (5.0-17.6)
**8. Melanoma, malignant**	**0.7 (0.5-1.0)**	**1.6 (1.3-2.0)**	**4.0 (3.3-4.9)**	**6.1 (5.0-7.3)**	**1.1 (0.9-1.3)**	**2.5 (2.2-2.9)**	**7.1 (6.4-7.8)**	**9.3 (8.3-10.2)**
8.1 Superficial spreading/low cumulative sun damage melanoma	0.8 (0.5-1.1)	1.7 (1.3-2.3)	4.9 (3.9-6.2)	6.6 (5.2-8.4)	1.0 (0.8-1.3)	2.4 (2.0-2.8)	7.6 (6.7-8.6)	9.3 (8.1-10.6)
8.2 Nodular melanoma	NA	NA	NA	NA	1.6 (0.9-2.6)	3.5 (2.4-5.0)	6.7 (4.9-9.0)	9.5 (6.6-12.9)
8.3 Other malignant	0.7 (0.4-1.3)	1.7 (1.1-2.5)	3.4 (2.4-4.7)	6.2 (4.4-8.5)	1.1 (0.8-1.6)	2.6 (2.0-3.3)	6.1 (5.0-7.4)	8.8 (7.2-10.5)
**9. Carcinomas**	**1.8 (1.5-2.1)**	**3.4 (3.0-3.9)**	**8.0 (7.1-8.9)**	**11.5 (10.2-12.8)**	**1.3 (1.2-1.5)**	**2.8 (2.6-3.0)**	**6.9 (6.6-7.3)**	**9.9 (9.4-10.5)**
9.1 Thyroid carcinoma	0.8 (0.3-1.7)	2.1 (1.2-3.5)	6.8 (4.4-9.8)	10.4 (7.0-14.7)	0.8 (0.5-1.2)	2.3 (1.7-3.1)	7.5 (6.0-9.1)	12.4 (10.0-15.0)
9.1.1 Medullary	NA	NA	NA	NA	1.0 (0.1-4.8)	2.2 (0.4-7.1)	10.5 (4.5-19.5)	19.5 (9.4-32.4)
9.1.3 Papillary	1.0 (0.3-2.3)	1.6 (0.7-3.4)	5.6 (2.8-9.6)	10.7 (5.8-17.4)	0.5 (0.2-1.0)	2.0 (1.3-3.0)	6.3 (4.4-8.7)	9.6 (6.6-13.3)
9.1.4 Follicular	NA	NA	NA	NA	1.5 (0.6-3.3)	3.6 (1.9-6.2)	8.9 (5.6-13.2)	13.6 (8.4-20.0)
9.1.5 Papillary with follicular variant	NA	NA	NA	NA	0.9 (0.4-2.1)	2.6 (1.4-4.3)	8.7 (5.9-12.3)	13.6 (9.4-18.6)
9.2 Other carcinoma of head and neck	2.8 (1.9-3.9)	5.0 (3.7-6.5)	13.1 (10.3-16.1)	17.4 (13.7-21.5)	3.7 (2.5-5.3)	5.8 (4.2-7.8)	13.0 (10.0-16.3)	16.1 (12.2-20.3)
9.2.1 Nasopharyngeal carcinoma	1.3 (0.3-4.4)	3.8 (1.4-8.2)	15.2 (6.6-27.0)	15.2 (6.6-27.0)	NA	NA	NA	NA
9.2.2 Oral cavity, lip, and pharynx	4.2 (2.8-6.2)	6.8 (4.8-9.3)	14.3 (10.6-18.4)	19.2 (14.2-24.8)	4.9 (2.9-7.6)	7.9 (5.2-11.2)	16.5 (11.7-22.0)	19.5 (13.5-26.3)
9.2.2.1 Oral cavity, lip, and pharynx, squamous	4.8 (3.1-7.1)	7.9 (5.6-10.8)	15.8 (11.6-20.5)	21.8 (15.9-28.2)	6.2 (3.5-9.8)	10.3 (6.6-14.8)	20.6 (14.4-27.5)	24.4 (16.7-32.9)
9.2.3 Salivary gland	NA	NA	NA	NA	2.7 (1.1-5.6)	3.9 (1.8-7.3)	8.4 (4.5-13.8)	10.8 (5.4-18.2)
9.2.3.2 Salivary gland, other malignant	NA	NA	NA	NA	3.4 (1.3-7.3)	5.1 (2.2-9.7)	10.1 (5.1-17.0)	10.1 (5.1-17.0)
9.2.4 Other carcinoma of head and neck	1.8 (0.5-4.8)	4.5 (2.0-8.7)	15.5 (9.2-23.3)	19.9 (11.9-29.4)	5.5 (2.0-11.4)	6.9 (2.8-13.5)	16.0 (8.3-26.0)	19.5 (10.0-31.3)
9.3 Carcinoma of gastrointestinal tract	1.8 (1.3-2.3)	3.2 (2.6-4.0)	6.5 (5.3-7.9)	9.5 (7.8-11.5)	1.7 (1.3-2.3)	2.8 (2.2-3.6)	7.3 (6.1-8.7)	10.4 (8.6-12.4)
9.3.2 Carcinoma of stomach	1.1 (0.4-2.8)	1.9 (0.8-4.0)	3.3 (1.4-6.4)	5.4 (2.5-10.0)	1.6 (0.6-3.6)	2.1 (0.9-4.4)	4.3 (2.0-8.0)	4.3 (2.0-8.0)
9.3.4 Carcinoma of colon	2.4 (1.6-3.4)	4.1 (3.0-5.5)	7.3 (5.5-9.4)	12.2 (9.2-15.7)	1.4 (0.8-2.1)	2.7 (1.9-3.8)	7.7 (6.0-9.7)	11.9 (9.2-14.9)
9.3.4.1 Appendix	NA	NA	NA	NA	NA	0.3 (0.0-1.7)	2.2 (0.9-4.5)	7.8 (4.1-13.0)
9.3.4.1.1 NET	NA	NA	NA	NA	NA	0.4 (0.0-1.9)	2.0 (0.7-4.4)	8.1 (4.1-13.7)
9.3.4.2 Colon excluding appendix	3.0 (2.0-4.3)	4.8 (3.4-6.5)	8.3 (6.1-10.8)	15.2 (11.2-19.8)	2.1 (1.3-3.2)	4.0 (2.7-5.5)	11.1 (8.5-14.1)	14.5 (11.0-18.5)
9.3.4.2.2 Colon excluding appendix, adenocarcinoma	3.0 (2.0-4.3)	4.8 (3.4-6.5)	8.4 (6.2-11.0)	15.1 (11.0-19.7)	2.0 (1.3-3.1)	3.9 (2.7-5.5)	11.0 (8.4-14.0)	14.5 (10.9-18.5)
9.3.5 Carcinoma of rectum	1.2 (0.6-2.3)	2.7 (1.6-4.3)	6.8 (4.4-10.0)	7.6 (4.9-11.2)	1.8 (1.0-3.2)	3.0 (1.7-4.7)	7.3 (4.7-10.5)	10.7 (6.6-16.0)
9.3.5.2 Rectum, adenocarcinoma	1.3 (0.6-2.6)	2.9 (1.7-4.7)	6.5 (4.1-9.7)	7.4 (4.6-10.9)	1.9 (1.0-3.3)	2.9 (1.7-4.7)	6.2 (3.9-9.3)	9.7 (5.7-15.0)
9.4 Carcinoma of lung, bronchus, and trachea	1.0 (0.4-2.0)	1.8 (0.9-3.3)	4.8 (2.8-7.6)	7.4 (4.3-11.7)	1.6 (0.9-2.7)	2.7 (1.7-4.0)	4.6 (3.0-6.6)	6.0 (3.9-8.6)
9.4.2 Non-small-cell carcinoma	0.7 (0.2-1.7)	1.5 (0.7-3.0)	4.3 (2.4-7.1)	7.4 (3.9-12.3)	1.7 (0.9-2.8)	2.8 (1.8-4.3)	4.9 (3.2-7.2)	6.5 (4.2-9.4)
9.4.2.1 Non-small-cell, adenocarcinoma	NA	NA	NA	NA	2.3 (1.1-4.3)	3.4 (1.8-5.8)	4.1 (2.2-7.1)	5.5 (2.7-9.7)
9.5 Carcinoma of skin (if collected)	3.8 (2.3-5.9)	7.2 (4.9-10.2)	14.2 (10.0-19.2)	17.8 (12.6-23.8)	3.0 (1.7-4.9)	7.1 (4.9-10.0)	13.4 (9.5-18.0)	19.4 (13.4-26.3)
9.6 Carcinoma of breast	NA	NA	NA	NA	1.1 (1.0-1.3)	2.4 (2.1-2.6)	5.6 (5.1-6.0)	7.8 (7.1-8.5)
9.6.1 Breast, infiltrating duct	NA	NA	NA	NA	1.2 (1.0-1.3)	2.4 (2.1-2.6)	5.6 (5.1-6.2)	7.6 (6.9-8.4)
9.6.2 Breast, adenocarcinoma	NA	NA	NA	NA	0.7 (0.4-1.2)	2.3 (1.6-3.2)	6.1 (4.7-7.7)	8.0 (6.2-10.0)
9.6.3 Breast, lobular	NA	NA	NA	NA	1.0 (0.5-1.8)	2.3 (1.4-3.5)	4.8 (3.2-6.8)	6.0 (4.0-8.4)
9.6.5 Breast, medullary	NA	NA	NA	NA	1.7 (0.7-3.3)	3.1 (1.7-5.2)	5.4 (3.2-8.3)	9.2 (5.6-13.8)
9.6.10 Breast, other	NA	NA	NA	NA	0.5 (0.1-1.8)	1.9 (0.9-3.7)	4.2 (2.4-6.7)	7.6 (4.6-11.4)
9.7 Carcinoma of genital sites excluding ovary and testis	NA	NA	NA	NA	1.5 (1.2-1.8)	3.3 (2.9-3.8)	9.1 (8.2-10.1)	13.3 (12.0-14.7)
9.7.1 Carcinoma of uterine cervix	NA	NA	NA	NA	1.5 (1.2-1.8)	3.1 (2.6-3.6)	8.7 (7.8-9.7)	13.1 (11.8-14.6)
9.7.1.1 Cervix, squamous	NA	NA	NA	NA	1.4 (1.1-1.8)	3.2 (2.7-3.8)	8.4 (7.4-9.6)	12.6 (10.9-14.3)
9.7.1.2 Cervix, adenosquamous	NA	NA	NA	NA	NA	3.7 (1.5-7.5)	8.3 (4.3-13.9)	13.4 (7.1-21.9)
9.7.1.3 Cervix, adenocarcinoma	NA	NA	NA	NA	2.0 (1.3-3.0)	3.0 (2.1-4.2)	11.4 (9.0-14.2)	16.5 (13.1-20.3)
9.7.1.4 Cervix, other	NA	NA	NA	NA	1.4 (0.5-3.3)	1.4 (0.5-3.3)	5.5 (3.2-8.9)	10.2 (6.4-15.1)
9.7.2 Corpus uteri	NA	NA	NA	NA	1.1 (0.3-2.9)	3.8 (1.8-6.8)	8.6 (5.0-13.4)	12.0 (6.7-18.9)
9.7.2.1 Corpus uteri, adenocarcinoma	NA	NA	NA	NA	1.4 (0.4-3.7)	5.0 (2.4-8.9)	10.0 (5.5-16.0)	12.5 (6.5-20.6)
9.7.2.1.2 Corpus uteri, other adenocarcinoma	NA	NA	NA	NA	1.8 (0.4-5.8)	7.6 (3.5-13.6)	13.4 (7.5-21.0)	15.7 (8.7-24.7)
9.7.3 Carcinoma of vulva and vagina	NA	NA	NA	NA	3.0 (1.5-5.4)	7.9 (5.0-11.7)	17.4 (12.2-23.4)	18.4 (12.9-24.7)
9.8 Carcinoma of urinary tract	1.2 (0.6-2.2)	2.9 (1.8-4.3)	8.0 (5.6-10.9)	12.1 (8.5-16.3)	1.3 (0.6-2.6)	3.5 (2.0-5.5)	9.9 (6.6-14.0)	14.1 (9.2-19.9)
9.8.1 Carcinoma of kidney	1.7 (0.8-3.0)	3.2 (1.8-5.1)	8.3 (5.2-12.3)	10.5 (6.5-15.7)	1.1 (0.4-2.7)	3.6 (1.8-6.4)	7.5 (4.1-12.2)	10.7 (5.7-17.3)
9.8.1.1 Kidney, adenocarcinoma	1.7 (0.8-3.1)	3.2 (1.8-5.1)	8.3 (5.2-12.4)	10.6 (6.5-15.8)	1.1 (0.4-2.7)	3.6 (1.8-6.4)	7.5 (4.1-12.2)	10.7 (5.7-17.3)
9.8.1.1.1 Kidney, renal cell	1.5 (0.6-2.9)	2.8 (1.5-4.8)	8.3 (5.1-12.5)	9.6 (5.7-14.7)	1.2 (0.4-3.0)	3.6 (1.7-6.5)	7.8 (4.2-12.8)	11.1 (5.9-18.2)
9.8.2 Carcinoma of bladder	0.4 (0.0-2.2)	2.4 (0.9-5.3)	7.3 (3.9-12.0)	14.5 (8.2-22.5)	1.3 (0.3-4.3)	3.0 (1.0-6.9)	11.8 (6.1-19.4)	18.2 (9.0-30.0)
9.8.2.1 Urinary bladder, transitional cell carcinoma	NA	2.4 (0.8-5.5)	6.5 (3.1-11.5)	13.3 (6.9-21.8)	1.8 (0.3-5.7)	3.9 (1.3-9.0)	14.3 (7.1-23.9)	17.2 (8.7-28.0)
9.9 Other invasive carcinomas	0.8 (0.2-2.5)	3.3 (1.4-6.3)	4.0 (1.9-7.5)	10.7 (4.8-19.4)	2.8 (1.4-5.0)	4.3 (2.4-7.0)	6.3 (3.6-9.9)	6.3 (3.6-9.9)

Cancer types were grouped according to the AYA-specific classification scheme developed by Barr and colleagues (2020).^35^ Death of any cause was included as a competing risk event.

AYA, adolescent and young adult; CNS, central nervous system; FIGO, Fédération Internationale de Gynécologie et d'Obstétrique; NA, not applicable; NET, neuroendocrine tumour; NK, natural killer; NLP, nodular lymphocyte predominant; NOS, not otherwise specified; TNM, tumour–node–metastasis.

a Age at diagnosis of first primary cancer.

### Sensitivity analysis

A flow chart of the study population selection procedure for the sensitivity analysis is presented in Supplementary Figure S1B, available at https://doi.org/10.1016/j.esmoop.2023.102203. Outcomes of the sensitivity analyses are presented in Supplementary Figure S7 and Tables S1B-S22, available at https://doi.org/10.1016/j.esmoop.2023.102203. SIRs, AERs, and cumulative incidences from the sensitivity analyses were generally higher compared to those that were obtained after applying the IACR/IARC rules for multiple cancers, with overall SIRs now indicating a 3-fold higher second cancer risk among male and a 2-fold higher risk among female survivors. AERs were, respectively, 32.5 and 39.5 per 10 000 person-years for male and female survivors, indicating a two times higher number of excess cancers in the sensitivity analyses in males and four times in females. Albeit still higher, outcomes by diagnostic group were more consistent between both analyses settings. However, SIR estimates obtained from the sensitivity analyses were noticeably two times higher among male testicular cancer, melanoma, and urinary tract carcinoma survivors. Testicular cancer survivors displayed a 10-fold higher risk of developing a second primary testicular cancer compared to the general population, whereas second melanoma risk was 13 times higher in male melanoma survivors. A noticeable 27 times higher risk of developing second kidney cancer was observed among male urinary tract carcinoma survivors (mostly kidney). SIRs also doubled for female melanoma survivors. Among female breast carcinoma survivors, a three times higher risk compared to the general population was observed in the sensitivity analyses, whereas risk was comparable in the main analyses. The risk of developing another breast cancer was four times higher in female breast cancer survivors compared to the general population, whereas female melanoma survivors had an 8-fold higher second melanoma risk. Higher risk throughout the entire follow-up duration was now also observed for male melanoma and gonadal and related tumour survivors. In female survivors this was the case for head and neck, genital site, and breast carcinomas (Supplementary Tables S1B-S22, available at https://doi.org/10.1016/j.esmoop.2023.102203).

## Discussion

This study comprehensively investigated the long-term second primary cancer risk in 6-month AYA (15-39 years at first diagnosis) cancer survivors for both sexes in the Netherlands between 1989 and 2018. Male survivors overall had a 2-fold higher risk of developing any cancer at 25-year follow-up compared to the general population, whereas this was around 1.3-fold in females, resulting in 17.5 (males) and 10.1 (females) excess cancers per 10 000 person-years. Higher long-term cancer risks compared to the general population were observed for most first and second primary cancer combinations. The cumulative incidence of second cancer steadily increased during the 25-year follow-up period to 8.9% in male and 10.3% in female survivors. Nevertheless, there were some noticeable risk differences depending on whether the IACR/IARC rules for multiple cancers were applied.

Cancer risk literature among AYA survivors is slowly increasing in recent years.^18^^,^^20^, ^21^, ^22^, ^23^, ^24^ Nevertheless, direct comparison of outcomes between available studies is difficult due to the use of different age ranges (e.g. 12-24 and 15-39 years), classification systems (e.g. Birch^40^ and Barr^35^), and applied primary cancer, multiple tumour, and latency period definitions. This is compounded by the use of different diagnostic periods, follow-up times, and general population definitions, which influences SIR and AER estimates by affecting the expected number calculation.

In line with previous findings, SIRs and AERs in this study showed an overall higher cancer risk among AYA survivors compared to the general population.^18^, ^19^, ^20^, ^21^^,^^23^^,^^24^^,^^41^ Cumulative incidences reported by previous studies indicated that around 10-17% of AYA survivors developed a subsequent cancer after 20-35 years of follow-up.^18^^,^^20^, ^21^, ^22^, ^23^, ^24^ Second cancer cumulative incidences in this study increased with time, but varied considerably depending on the first primary cancer type, which is supported by previous findings.^20^, ^21^, ^22^ As in other studies, the highest long-term cumulative incidences were found among both male and female (Hodgkin’s) lymphoma survivors.^22^ Previous studies all reported higher cancer risks among AYA survivors for most first and second primary cancer combinations.^18^, ^19^, ^20^, ^21^, ^22^, ^23^, ^24^ Still, contradicting observations have been described and likely relate to the previously described methodological and cohort differences. For some specific combinations, this likely also relates to smaller sample sizes, which can inflate the resulting excess risk estimates and it is therefore generally advised to carefully interpret outcomes that are based on small sample sizes.

Despite falling outside the scope of most AYA papers, the literature suggests several intrinsic and extrinsic factors that (in composite) might explain the higher cancer risk among AYA cancer survivors.^42^^,^^43^ Depending on first cancer type, Chao and colleagues observed different second cancer risk factors.^21^ For instance, higher risk of solid malignancies among AYA survivors was independently associated with white race/ethnicity, receipt of radiotherapy, female sex, and older age and advanced stage at first cancer diagnosis.^21^ Female sex was only associated with increased risk when second breast cancers were included, whereas an inverted association was observed otherwise.^21^ Elsewhere, female sex was found to be a risk factor among teenage and young adult Hodgkin’s lymphoma survivors,^44^ and a protective factor among AYA melanoma^45^ and thyroid cancer survivors.^46^ As stated by Chao et al., the observed risk factor variation between first cancer types might hint at different pathogenic mechanisms, but additional research is needed to ascertain this.^21^ Nevertheless, current findings do emphasize the importance of risk stratification by first primary cancer type.

Higher risks of subsequent cancers have also been related to (combined) receipt of radiotherapy and chemotherapy,^21^^,^^23^^,^^44^^,^^47^ in several large case-control studies (not AYA exclusive), for a large number of second primary cancer types, including breast, stomach, small intestine, liver, pancreas, lung, kidney, and bladder cancers.^27^^,^^48^, ^49^, ^50^ Higher cancer risk after radiotherapy and chemotherapy exposure in Hodgkin’s lymphoma survivors is also well-documented.^26^^,^^27^^,^^51^ Radiotherapy volumes and doses have become smaller over time,^52^^,^^53^ but the introduction of more modern therapies does not appear to have resulted in a lower risk of second malignancies.^26^^,^^27^^,^^54^ In line with previous studies, AYA lymphoma survivors in this study had a higher risk of developing most second cancers and overall had the highest SIRs and AERs for all second cancers combined.^18^^,^^20^^,^^22^, ^23^, ^24^^,^^55^ Considering the disease prevalence, most previous studies included a young survivor population (aged ≤50 years), making it likely that the increased risk of second cancer among AYA lymphoma survivors is to some extent also treatment-induced. Nevertheless, investigation of risk estimates by first cancer treatment fell outside the scope of this and most other AYA-centric studies, warranting the need for more tumour-centric studies incorporating treatment exposure among AYA survivors.

Changes in radiotherapy and chemotherapy dose over time have resulted in lower breast cancer rates among childhood cancer survivors.^56^ Our main analyses showed female AYA survivors to have a noticeable lower second breast cancer risk, especially among first breast cancer survivors. However, this was not supported by the results from the sensitivity analyses, showing a 3-fold higher breast cancer risk among female AYA survivors compared to the general population. In the sensitivity analyses, a higher risk for developing another breast cancer was also found among AYA breast cancer survivors, which is in line with the substantial higher same site breast cancer risk that has been reported by previous studies.^21^^,^^24^ The differences in breast cancer risk between our main and sensitivity analyses most likely relate to the IACR/IARC rules for multiple cancers, which were applied in the main analyses and exclude all consecutive malignancies with identical topography and morphology. This might also explain why male gonadal and related tumour survivors in the main analyses had a lower second testicular cancer risk compared to the general population, whereas outcomes from the sensitivity analyses indicated a marked 10-fold higher risk. Similar high melanoma risk outcomes were observed among melanoma survivors (both sexes), whereas this risk combination was not available when the IACR/IARC rules were applied. Still, the outcomes of the sensitivity analyses are likely biased and it should be noted that other studies have reported a 5-38 times higher risk of developing second testicular cancer (different laterality) among AYA testicular cancer survivors whilst applying multiple cancer rules, including the IACR/IARC rules.^21^^,^^24^

Genetic predisposition (syndromes) have also been suggested by an accumulating body of evidence, especially among younger cancer patients.^57^, ^58^, ^59^ Among others, this includes *BRCA* and the well-known *TP53* germline mutation-related Li–Fraumeni syndrome, which increases the risk of several AYA-specific cancers.^59^, ^60^, ^61^ *BRCA1* and *BRCA2* mutation carriers typically have increased breast, ovarian, uterine, and pancreatic cancer risk.^59^^,^^62^ Breast cancer survivors in this study also had a higher risk of developing second ovarian and pancreatic cancers, which might hint at a shared genetic predisposition pattern.

Modifiable lifestyle factors likely also play an important role in second cancer development. Smoking is a well-known risk factor for developing cancer in general and has been shown to increase the risk of second lung and other tobacco-related cancers (e.g. oesophagus, colorectal, stomach, and haematological malignancies) in cancer survivors of all ages.^63^, ^64^, ^65^, ^66^ Previous findings show that AYA survivors (especially females) more often smoke compared to a similar population without cancer (33% versus 22%) and that ever (those who had regularly smoked before or at diagnosis) and current smokers and drinkers at the time of first primary cancer diagnosis in general had a higher second cancer risk compared to those who never smoked or consumed alcohol regularly during their lifetime.^63^^,^^67^^,^^68^

There is also evidence that second cancers are biologically different compared to first primaries of the same type.^29^^,^^46^^,^^69^ As such, better survivorship care guidelines and targeted interventions aimed at prevention and early detection of second cancers among AYAs are desperately needed, especially among radiotherapy recipients. Less invasive radiotherapy and chemotherapy regimens have resulted in lower cancer rates among childhood cancer survivors.^56^ Safer treatments that minimize toxic effects may also lower the long-term subsequent cancer risk among AYA survivors and should be explored considering that AYAs have many life years remaining. Results from this and related studies could help conceptualise such guidelines for AYA cancer survivors, which was also suggested elsewhere.^21^ Nevertheless, more studies investigating (modifiable) risk factors for the specific cancer type combinations among AYA survivors are required to best inform and further guide the development of age-appropriate survivorship care guidelines, as detailed information about second cancer risk factors among AYAs remains scarce, especially about treatment dose/exposure and modifiable factors like obesity, smoking, and physical activity.^43^

This study has several strengths, including the generalizability of outcomes to the entire Netherlands population by using data from the population-based NCR, which contains complete records of all malignant cancers in the Netherlands since 1989 and has a near 100% coverage.^32^ Use of these data also minimizes selection bias, which is another major strength. Furthermore, we included all first and second cancer types based on observed numbers rather than making a prior selection, as was done in most previous studies. Outcomes in this study were also presented for males and females separately, resulting in a detailed risk overview in general and for distinct diagnostic subgroups. Adding to this, we reported both SIRs and AERs, whereas previous studies mostly reported SIRs. Self-contained interpretation of relative measures like the SIR is difficult, as they can easily inflate in a low number setting, providing no guidance to health services.

This study also has several limitations. Firstly, cancers with a non-malignant behaviour were not taken into consideration, but their existence and treatment may have affected the second cancer risk profile within our cohort. Furthermore, we included AYA 6-month survivors, whereas other studies usually adopted a 5-year latency period to not misclassify recurrences and metastases as new primaries. Albeit considerably shorter, a recent study into the most appropriate period to define synchronous cancers showed 4 months to be sufficient.^34^ As confirmed by our sensitivity analyses, application of the IACR/IARC rules resulted in a noticeable second cancer risk underestimation in melanoma, testicular, and breast cancer survivors, all of which had a high frequency of developing subsequent malignancies with identical topography and morphology [*n* = 125 (20.2%) testicular, *n* = 155 (25.1%) melanoma, and *n* = 278 (45.0%) breast cancers]. Our general background population likely amplified this effect by including all malignancies, including those that were excluded from the survivor population based on the IACR/IARC rules. Although risk outcomes remained similar in most cases otherwise, researchers should carefully consider how to best define their survivor and background populations and in all cases provide a clear description of this process when investigating second cancer risk. Lastly, despite being available in the NCR, treatment fell outside the scope of this study due to the large number of detailed combinations of diagnostic subgroups. Considering that treatment can vary greatly per cancer type, we believe that it is better to address this topic in more tumour-centric studies.

In conclusion, this study demonstrated that AYA cancer survivors in the Netherlands have a higher cancer risk compared to the general population for most first and second primary malignant cancer combinations up to 25 years after their initial diagnosis between 1989 and 2018. Considerable risk variation was observed for both sexes and between cancer types, but interpretation requires some caution due to low numbers and underestimation of risks caused by the IACR/IARC rules. Nevertheless, these findings highlight the need for personalized follow-up strategies. Additional studies that investigate risk factors for the specific cancer type combinations are needed to best inform and develop such tailored survivorship care guidelines for AYA cancer survivors.
