# Supplementary material for: Long-term second primary cancer risk in adolescent and young adult (15-39 years) cancer survivors: a population-based study in the Netherlands between 1989 and 2018
Source: ESMO Open. 2024 Jan 2;9(1):102203. doi: 10.1016/j.esmoop.2023.102203 (PMC10837779; doi:10.1016/j.esmoop.2023.102203)
Supplement: Supplementary data [file mmc1.docx]

**Supplementary Materials:**

**Long-term Second Primary Cancer Risk in Adolescent and Young Adult (15−39 years) Cancer Survivors: a population-based study in the Netherlands between 1989 and 2018**

Daniël J. van der Meer, Winette T.A. van der Graaf, Deborah van de Wal, Henrike E. Karim-Kos and Olga Husson

**Included (N=99,502 first & N=4,344 second cancers)**

- AYA six-month cancer survivors included for analyses

**Included (N=106,504 first & N=4,669 second cancers)**

- AYA cancer survivors aged 15-39 years at the time of first cancer diagnosis
- Survivors with identical follow-up for second cancer and death included as death (n=7)

**Included (N=107,686 first & N=4,758 second cancers)**

- AYA cancer survivors with cancers that satisfied the IARC rules for multiple cancers

**Selected from the NCR (N=107,820 first & N=7,643 second cancers)**

- AYA cancer survivors diagnosed in the Netherlands between 1989-2018
- No prior childhood cancers (Age <15 years)
- Both sexes (Males & females)
- Any topography
- Any morphology
- Malignant cancers only (Behaviour /3)
- All tumour stages

**Excluded**

- Cancers that did not satisfy the IARC rules for multiple cancers
  - N=134 first cancers
  - N=2,885 second cancers

**Excluded**

- AYAs not aged 15-39 years at first cancer diagnosis*
  - N=1,182 first cancers
  - N=89 second cancers

**Excluded**

- AYA survivors with follow-up <six months after first cancer
  - N=7,002 first cancers
  - N=318 second cancers

* Numbering of sequential malignancies after removing all cancers with non-malignant behaviour from the database resulted in first primary cancers that were diagnosed outside the 15-39 years AYA age range. These cases were removed from the analyses.

**Supplementary Figure S1A.** Study population flow chart of adolescent and young adult (AYA) cancer survivors diagnosed with first and second primary malignant cancer in the Netherlands between 1989-2018. Data were obtained from the population-based Netherlands Cancer Registry (NCR). This Figure presents the case selection procedure for the main analysis, which included only cancers that satisfied the international rules for multiple cancers published by the International agency for research on cancer (IARC).

**Selected from the NCR (N=107,820 first & N=7,643 second cancers)**

- AYA cancer survivors diagnosed in the Netherlands between 1989-2018
- No prior childhood cancers (Age <15 years)
- Both sexes (Males & females)
- Any topography
- Any morphology
- Malignant cancers only (Behaviour /3)
- All tumour stages

**Included (N=99,144 first & N=6,694 second cancers)**

- AYA six-month cancer survivors included for analyses

**Included (N=106,631 first & N=7,525 second cancers)**

- AYA cancer survivors aged 15-39 years at the time of first cancer diagnosis
- Survivors with identical follow-up for second cancer and death included as death (n=7)

**Excluded**

- AYAs not aged 15-39 years at first cancer diagnosis*
  - N=1,189 first cancers
  - N=118 second cancers

**Excluded**

- AYA survivors with follow-up <six months after first cancer
  - N=7,487 first cancers
  - N=824 second cancers

* Numbering of sequential malignancies after removing all cancers with non-malignant behaviour from the database resulted in first primary cancers that were diagnosed outside the 15-39 years AYA age range. These cases were removed from the analyses.

**Supplementary Figure S1B.** Study population flow chart of adolescent and young adult (AYA) cancer survivors diagnosed with first and second primary malignant cancer in the Netherlands between 1989-2018. Data were obtained from the population-based Netherlands Cancer Registry (NCR). This Figure presents the case selection procedure for the sensitivity analysis.

**Supplementary Figure S2.** Relative frequencies (%) of first and second primary malignant cancer types among adolescents and young adults (AYA) diagnosed at ages 15-39 years in the Netherlands between 1989-2018. Cancer types were grouped according to the AYA-specific classification scheme developed by Barr and colleagues (2020).

**Supplementary Figure S3.** Overall standardised incidence ratios (SIRs) and absolute excess risks (AERs) of any second primary malignancy diagnosed after first primary cancer among six-month adolescent and young adult (AYA, aged 15-39 years) cancer survivors compared to the general population in the Netherlands between 1989-2018. Cancer types were grouped according to the AYA-specific classification scheme developed by Barr and colleagues (2020). Cancer combinations with less than n=10 observed second cancers were excluded from the analyses.

**Supplementary Figure S4.** Standardised incidence ratios (SIRs) and absolute excess risks (AERs) of any second primary malignant cancer diagnosis after first primary malignant cancer among six-month adolescent and young adult (AYA, aged 15-39 years) cancer survivors compared to the general population in the Netherlands by follow-up period. First and second cancers are presented combined to obtain overall risk results.


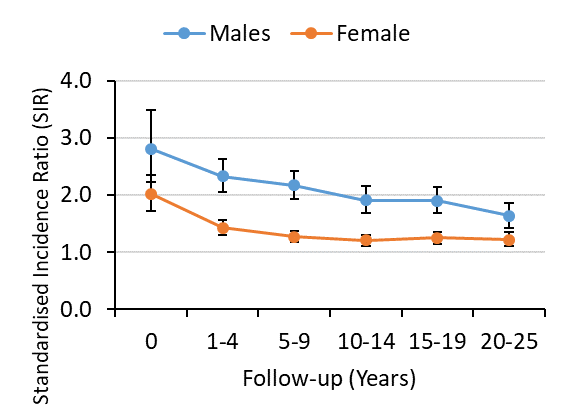

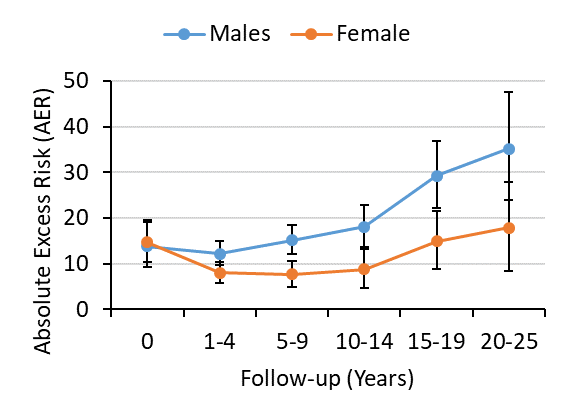


**Supplementary Figure S5.** Cumulative incidence of any second primary malignancy up-to 25 years after first primary cancer among six-month adolescent and young adult (AYA, aged 15-39 years) cancer survivors in the Netherlands between 1989-2018. Outcomes are presented by first primary cancer type. Cancer types were grouped according to the AYA-specific classification scheme developed by Barr and colleagues (2020). Death of any cause was included as competing risk event.


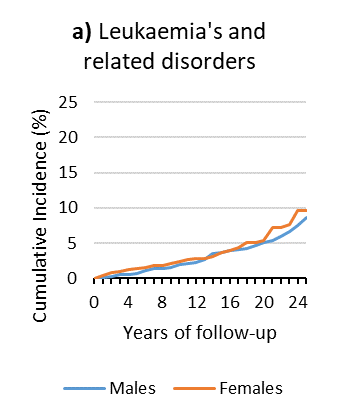

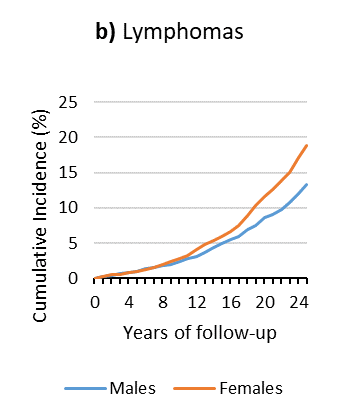

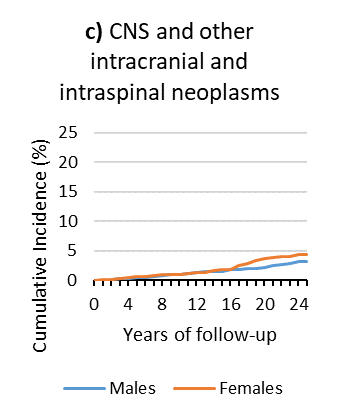

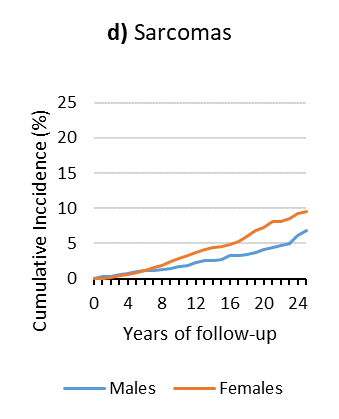

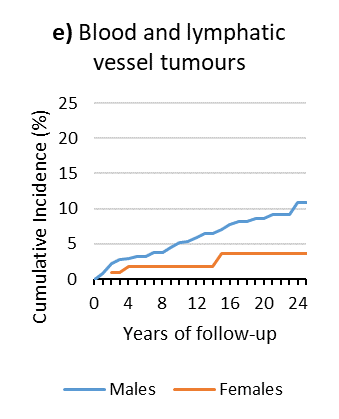

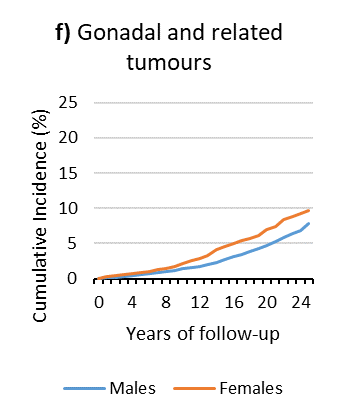

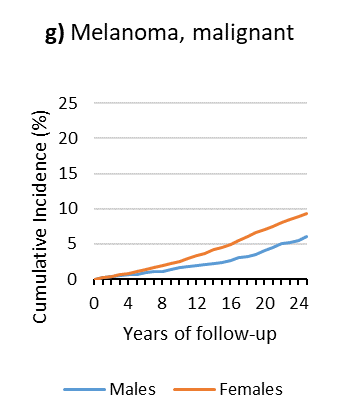

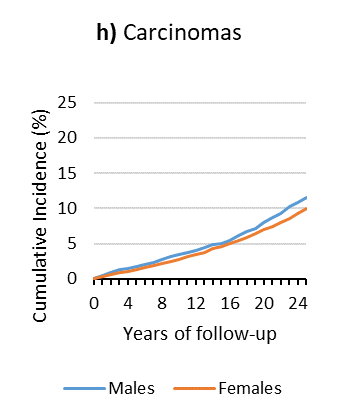


**Supplementary Figure S6.** Cumulative incidence of any second primary malignancy up-to 25 years after first primary cancer among six-month adolescent and young adult (AYA, aged 15-39 years) cancer survivors in the Netherlands between 1989-2018. Outcomes are presented by first primary carcinoma subtype. Cancer types were grouped according to the AYA-specific classification scheme developed by Barr and colleagues (2020). Death of any cause was included as competing risk event.


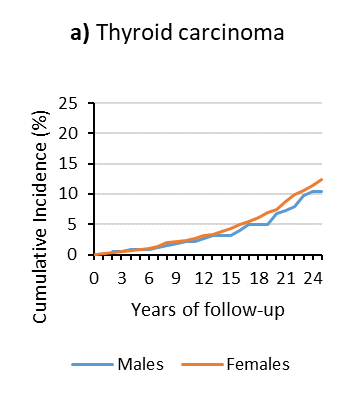

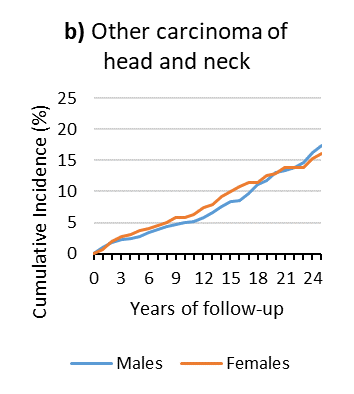

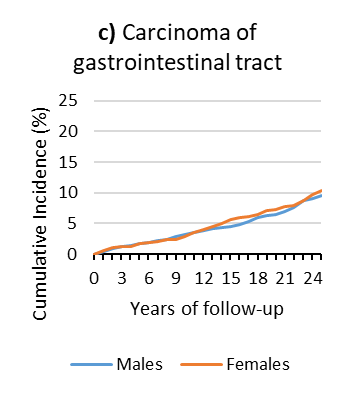

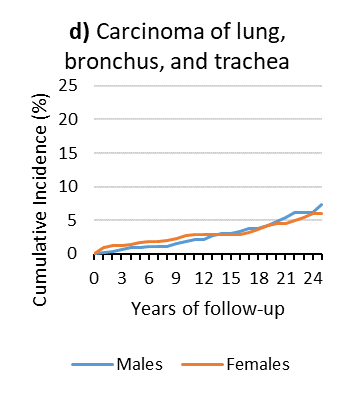

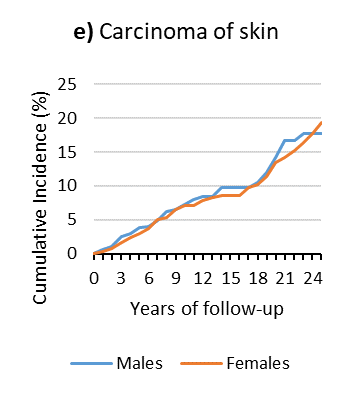

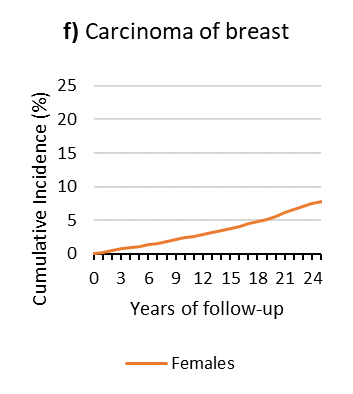

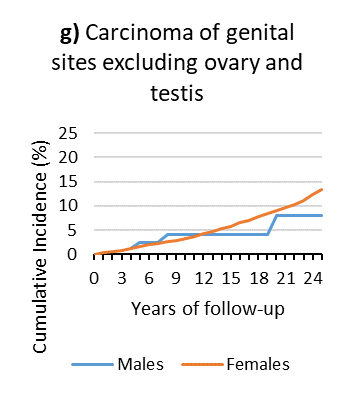

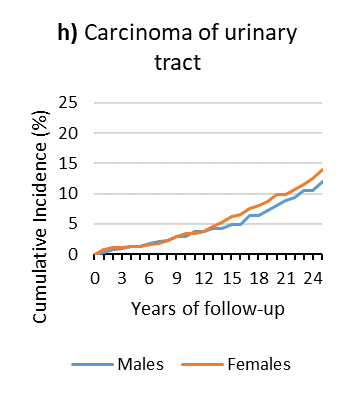

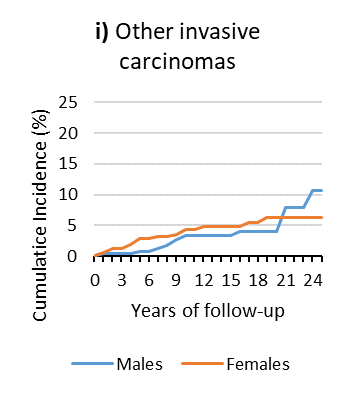

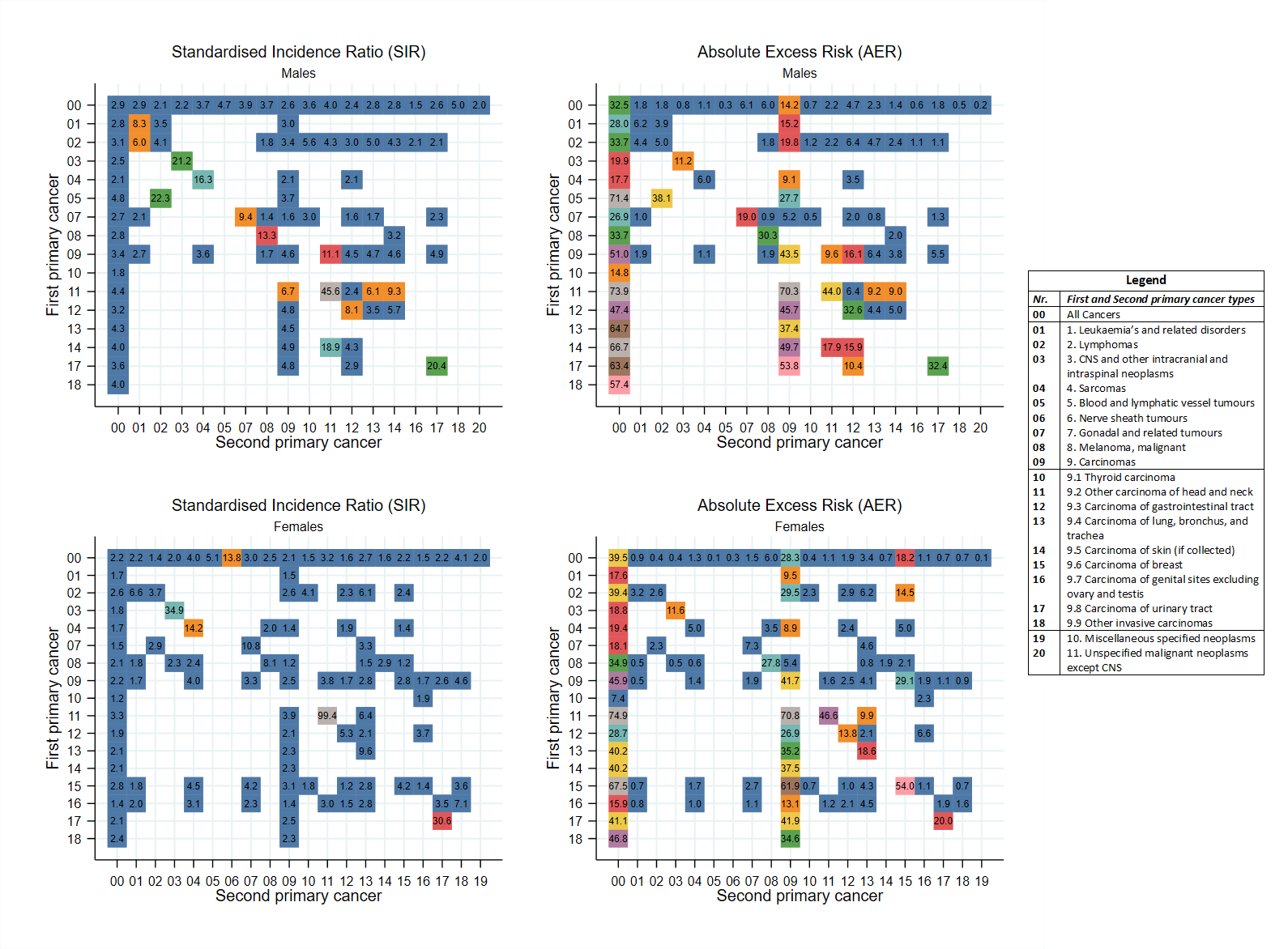


**Supplementary Figure S7.** Standardised incidence ratios (SIRs) and absolute excess risks (AERs) of first and second primary malignant cancer combinations diagnosed among six-month adolescent and young adult (AYA, aged 15-39 years) cancer survivors compared to the general population in the Netherlands between 1989-2018. Cancer types were grouped according to the AYA-specific classification scheme developed by Barr and colleagues (2020). Only cancer combinations with at least n=10 observed second cancers and significant excess risk estimates are presented. This Table presents outcomes from the sensitivity analysis.

**Supplementary Table S1A.** Population, tumour and treatment characteristics of six-month adolescent and young adult (AYA, aged 15-39 years) cancer survivors diagnosed with first and second primary malignant cancer in the Netherlands between 1989-2018. Cancer types were grouped according to the AYA-specific classification scheme developed by Barr and colleagues (2020). This Table presents outcomes from the main analysis.

|  | **First primary cancers** | | **Second primary cancers** | |
| --- | --- | --- | --- | --- |
|  | **Males** | **Females** | **Males** | **Females** |
|  | **n= (%)** | **n= (%)** | **n= (%)** | **n= (%)** |
| **Total^a^** | **39,892 (100)** | **59,610 (100)** | **1,471 (100)** | **2,873 (100)** |
| **Cancer types** |  |  |  |  |
| **1. Leukaemia’s and related disorders** | **2,318 (5.8)** | **1,951 (3.3)** | **99 (6.7)** | **99 (3.4)** |
| 1.1 Acute lymphoblastic leukaemia | 660 (1.7) | 407 (0.7) | 13 (0.9) | 5 (0.2) |
| 1.2 Acute myeloid leukaemia | 616 (1.5) | 674 (1.1) | 38 (2.6) | 41 (1.4) |
| 1.2.1 Acute promyelocytic leukaemia | 73 (0.2) | 103 (0.2) | 2 (0.1) | 1 (0.0) |
| 1.2.2 Other acute myeloid leukaemia | 543 (1.4) | 571 (1.0) | 36 (2.4) | 40 (1.4) |
| 1.3 Chronic myeloid leukaemia | 433 (1.1) | 264 (0.4) | 11 (0.7) | 11 (0.4) |
| 1.4 Chronic lymphocytic leukaemia | 114 (0.3) | 45 (0.1) | 8 (0.5) | 12 (0.4) |
| 1.5 Polycythaemia vera | 74 (0.2) | 71 (0.1) | 1 (0.1) | 1 (0.0) |
| 1.6 Essential thrombocythemia | 145 (0.4) | 283 (0.5) | 6 (0.4) | 9 (0.3) |
| 1.7 Primary myelofibrosis | 20 (0.1) | 19 (0.0) | 1 (0.1) | 1 (0.0) |
| 1.8 Myelodysplastic syndrome (MDS) | 86 (0.2) | 100 (0.2) | 15 (1.0) | 13 (0.5) |
| 1.9 Other and unspecified leukaemia’s and related disorders | 170 (0.4) | 88 (0.1) | 6 (0.4) | 6 (0.2) |
| 1.9.1 Hairy cell leukaemia | 82 (0.2) | 13 (0.0) | 3 (0.2) | 2 (0.1) |
| 1.9.2 Other lymphocytic/lymphoblastic leukaemia | 13 (0.0) | 13 (0.0) | 1 (0.1) | 2 (0.1) |
| 1.9.3 Other myeloid leukaemia | 17 (0.0) | 12 (0.0) | 1 (0.1) | 1 (0.0) |
| 1.9.4 Leukaemia’s of mixed phenotype | 12 (0.0) | 12 (0.0) | NA | NA |
| 1.9.5 Mast cell diseases | 13 (0.0) | 12 (0.0) | NA | 1 (0.0) |
| 1.9.6 Other | 33 (0.1) | 26 (0.0) | 1 (0.1) | NA |
| **2. Lymphomas** | **6,348 (15.9)** | **4,614 (7.7)** | **132 (9.0)** | **98 (3.4)** |
| 2.1 Non-Hodgkin lymphomas | 2,764 (6.9) | 1,693 (2.8) | 86 (5.8) | 82 (2.9) |
| 2.1.1 Lymphoblastic | 166 (0.4) | 40 (0.1) | 1 (0.1) | 2 (0.1) |
| 2.1.2 Burkitt | 172 (0.4) | 57 (0.1) | 3 (0.2) | NA |
| 2.1.3 Diffuse large B-cell (DLBCL) | 1,099 (2.8) | 649 (1.1) | 50 (3.4) | 35 (1.2) |
| 2.1.4 Primary mediastinal large B-cell excluded from DLBCL | 115 (0.3) | 140 (0.2) | 2 (0.1) | 1 (0.0) |
| 2.1.5 Anaplastic T-cell and null-cell excluding NK/T-cell | 428 (1.1) | 273 (0.5) | 8 (0.5) | 10 (0.3) |
| 2.1.6 Follicular | 417 (1.0) | 289 (0.5) | 9 (0.6) | 21 (0.7) |
| 2.1.7 NK/T-cell (excluded from anaplastic T-cell) | 14 (0.0) | 8 (0.0) | 1 (0.1) | NA |
| 2.1.8 MALT (Mucosa-associated lymphoid tissue) | 179 (0.4) | 131 (0.2) | 3 (0.2) | 11 (0.4) |
| 2.1.9 Other non-Hodgkin lymphoma NOS | 174 (0.4) | 106 (0.2) | 9 (0.6) | 2 (0.1) |
| 2.2 Hodgkin lymphoma | 3,210 (8.0) | 2,705 (4.5) | 30 (2.0) | 5 (0.2) |
| 2.2.1 Hodgkin NLP | 249 (0.6) | 76 (0.1) | 3 (0.2) | NA |
| 2.2.2 Hodgkin classic, other | 2,961 (7.4) | 2,629 (4.4) | 27 (1.8) | 5 (0.2) |
| 2.3 Myeloma | 180 (0.5) | 106 (0.2) | 7 (0.5) | 10 (0.3) |
| 2.4 Cutaneous lymphomas | 26 (0.1) | 15 (0.0) | 1 (0.1) | NA |
| 2.5 Other B-cell and T-cell lymphomas | 103 (0.3) | 56 (0.1) | 5 (0.3) | 1 (0.0) |
| 2.6 Other lymphomas, specified and unspecified | 65 (0.2) | 39 (0.1) | 3 (0.2) | NA |
| 2.6.1 Histiocytic and dendritic cell neoplasms | 45 (0.1) | 27 (0.0) | NA | NA |
| 2.6.2 Lymphoma NOS | 20 (0.1) | 12 (0.0) | 3 (0.2) | NA |
| **3. CNS and other intracranial and intraspinal neoplasms** | **2,556 (6.4)** | **1,761 (3.0)** | **41 (2.8)** | **39 (1.4)** |
| 3.1 Astroglial and related neoplasms | 2,257 (5.7) | 1,546 (2.6) | 36 (2.4) | 33 (1.1) |
| 3.1.1 Oligodendriogliomas | 511 (1.3) | 339 (0.6) | 3 (0.2) | 6 (0.2) |
| 3.1.1.2 Oligodendrioglioma, invasive | 511 (1.3) | 339 (0.6) | 3 (0.2) | 6 (0.2) |
| 3.1.2 Glioblastomas/gliofibromas | 346 (0.9) | 197 (0.3) | 16 (1.1) | 15 (0.5) |
| 3.1.2.2 Glioblastoma, invasive | 346 (0.9) | 197 (0.3) | 16 (1.1) | 15 (0.5) |
| 3.1.3 Ependymomas | 169 (0.4) | 136 (0.2) | 2 (0.1) | 2 (0.1) |
| 3.1.3.2 Ependymoma, invasive | 169 (0.4) | 136 (0.2) | 2 (0.1) | 2 (0.1) |
| 3.1.4 Other astrocytoma/astroglial neoplasms | 1,231 (3.1) | 874 (1.5) | 15 (1.0) | 10 (0.3) |
| 3.1.4.1 Pilocytic astrocytoma | 70 (0.2) | 43 (0.1) | NA | NA |
| 3.1.4.3 Other astrocytoma/astroglial, invasive | 1,161 (2.9) | 831 (1.4) | 15 (1.0) | 10 (0.3) |
| 3.2 Medulloblastoma and other invasive embryonal CNS tumours | 137 (0.3) | 88 (0.1) | NA | 1 (0.0) |
| 3.3 Neuroblastomas/ganglioneuromas | 3 (0.0) | 5 (0.0) | NA | NA |
| 3.3.2 Neuroblastoma/ganglioneuroblastoma, invasive | 3 (0.0) | 5 (0.0) | NA | NA |
| 3.4 Neuronal and mixed neuronal-glial neoplasms | 9 (0.0) | 3 (0.0) | NA | NA |
| 3.4.2 Neuronal and mixed neuronal-glial, invasive | 9 (0.0) | 3 (0.0) | NA | NA |
| 3.5 Meningioma’s | 15 (0.0) | 14 (0.0) | 2 (0.1) | NA |
| 3.5.2 Meningioma, invasive | 15 (0.0) | 14 (0.0) | 2 (0.1) | NA |
| 3.6 Choroid plexus neoplasms | 1 (0.0) | 2 (0.0) | NA | NA |
| 3.6.2 Choroid plexus, invasive | 1 (0.0) | 2 (0.0) | NA | NA |
| 3.8 Pituitary neoplasms | 4 (0.0) | NA | NA | NA |
| 3.8.2 Pituitary, invasive | 4 (0.0) | NA | NA | NA |
| 3.9 Pineal neoplasms | 16 (0.0) | 17 (0.0) | NA | NA |
| 3.9.2 Pineal, invasive | 16 (0.0) | 17 (0.0) | NA | NA |
| 3.10 Other and unspecified CNS neoplasms | 114 (0.3) | 86 (0.1) | 3 (0.2) | 5 (0.2) |
| 3.10.2 Other and unspecified CNS, invasive | 114 (0.3) | 86 (0.1) | 3 (0.2) | 5 (0.2) |
| **4. Sarcomas** | **2,469 (6.2)** | **2,229 (3.7)** | **52 (3.5)** | **105 (3.7)** |
| 4.1 Osteosarcoma | 333 (0.8) | 221 (0.4) | 3 (0.2) | 11 (0.4) |
| 4.2 Chondrosarcoma | 339 (0.8) | 339 (0.6) | 4 (0.3) | 21 (0.7) |
| 4.3 Ewing family of tumours | 275 (0.7) | 177 (0.3) | 3 (0.2) | 4 (0.1) |
| 4.3.1 Bone | 193 (0.5) | 104 (0.2) | 2 (0.1) | 1 (0.0) |
| 4.3.2 Soft tissue | 82 (0.2) | 73 (0.1) | 1 (0.1) | 3 (0.1) |
| 4.4 Fibromatous neoplasms | 532 (1.3) | 574 (1.0) | 8 (0.5) | 14 (0.5) |
| 4.4.1 Myxofibrosarcoma | 34 (0.1) | 39 (0.1) | 2 (0.1) | 6 (0.2) |
| 4.4.2 Malignant fibrous histiocytoma | 69 (0.2) | 50 (0.1) | 1 (0.1) | 2 (0.1) |
| 4.4.3 Other fibromatous neoplasms | 429 (1.1) | 485 (0.8) | 5 (0.3) | 6 (0.2) |
| 4.5 Liposarcoma | 208 (0.5) | 203 (0.3) | 2 (0.1) | 9 (0.3) |
| 4.6 Synovial sarcoma | 158 (0.4) | 126 (0.2) | 2 (0.1) | 5 (0.2) |
| 4.7 Leiomyosarcoma | 144 (0.4) | 171 (0.3) | 3 (0.2) | 10 (0.3) |
| 4.8 Rhabdomyosarcoma | 130 (0.3) | 74 (0.1) | 1 (0.1) | 3 (0.1) |
| 4.9 Gastrointestinal stromal tumour, malignant | 71 (0.2) | 58 (0.1) | 4 (0.3) | 4 (0.1) |
| 4.10 Spindle cell sarcoma | 15 (0.0) | 12 (0.0) | 2 (0.1) | 1 (0.0) |
| 4.11 Epithelioid sarcoma | 40 (0.1) | 22 (0.0) | NA | 1 (0.0) |
| 4.12 Desmoplastic small round cell tumour | 11 (0.0) | 4 (0.0) | NA | NA |
| 4.13 Chordoma | 30 (0.1) | 26 (0.0) | 1 (0.1) | 1 (0.0) |
| 4.14 Giant cell sarcoma | 18 (0.0) | 12 (0.0) | 4 (0.3) | 6 (0.2) |
| 4.15 Other soft tissue sarcomas | 113 (0.3) | 144 (0.2) | 12 (0.8) | 13 (0.5) |
| 4.16 Other bone tumours | 52 (0.1) | 66 (0.1) | 3 (0.2) | 2 (0.1) |
| **5. Blood and lymphatic vessel tumours** | **650 (1.6)** | **120 (0.2)** | **16 (1.1)** | **11 (0.4)** |
| 5.2 Malignant blood and lymphatic vessel tumours, all sites | 650 (1.6) | 120 (0.2) | 16 (1.1) | 11 (0.4) |
| 5.2.1 Kaposi sarcoma | 591 (1.5) | 44 (0.1) | 6 (0.4) | NA |
| 5.2.2 Other | 59 (0.1) | 76 (0.1) | 10 (0.7) | 11 (0.4) |
| **6. Nerve sheath tumours** | **123 (0.3)** | **105 (0.2)** | **5 (0.3)** | **14 (0.5)** |
| 6.2 Malignant | 123 (0.3) | 105 (0.2) | 5 (0.3) | 14 (0.5) |
| 6.2.1 MPNST (Malignant peripheral nerve sheath tumour) | 90 (0.2) | 77 (0.1) | 5 (0.3) | 14 (0.5) |
| 6.2.1.1 CNS | 2 (0.0) | 3 (0.0) | NA | NA |
| 6.2.1.2 Peripheral | 88 (0.2) | 74 (0.1) | 5 (0.3) | 14 (0.5) |
| 6.2.2 Other | 33 (0.1) | 28 (0.0) | NA | NA |
| **7. Gonadal and related tumours** | **12,074 (30.3)** | **2,249 (3.8)** | **55 (3.7)** | **124 (4.3)** |
| 7.1 Testis | 11,716 (29.4) | NA | 52 (3.5) | NA |
| 7.1.1 Germ cell and trophoblastic | 11,684 (29.3) | NA | 52 (3.5) | NA |
| 7.1.1.1 Seminoma | 5,444 (13.6) | NA | 33 (2.2) | NA |
| 7.1.1.2 Embryonal carcinoma | 1,690 (4.2) | NA | 2 (0.1) | NA |
| 7.1.1.3 Endodermal sinus (yolk sac tumour) | 223 (0.6) | NA | NA | NA |
| 7.1.1.4 Teratoma | 1,468 (3.7) | NA | 3 (0.2) | NA |
| 7.1.1.5 Mixed germ cell | 2,277 (5.7) | NA | 14 (1.0) | NA |
| 7.1.1.6 Choriocarcinoma and other trophoblastic | 452 (1.1) | NA | NA | NA |
| 7.1.1.7 Other | 130 (0.3) | NA | NA | NA |
| 7.1.2 Non-germ cell | 32 (0.1) | NA | NA | NA |
| 7.1.2.1 Carcinoma | 6 (0.0) | NA | NA | NA |
| 7.1.2.2 Sex cord | 26 (0.1) | NA | NA | NA |
| 7.2 Ovary | NA | 2,034 (3.4) | NA | 124 (4.3) |
| 7.2.1 Germ cell and trophoblastic | NA | 357 (0.6) | NA | 3 (0.1) |
| 7.2.1.1 Teratoma | NA | 139 (0.2) | NA | 2 (0.1) |
| 7.2.1.2 Dysgerminoma | NA | 110 (0.2) | NA | NA |
| 7.2.1.3 Yolk sac | NA | 61 (0.1) | NA | NA |
| 7.2.1.4 Mixed germ cell | NA | 26 (0.0) | NA | 1 (0.0) |
| 7.2.1.5 Other germ cell and trophoblastic | NA | 21 (0.0) | NA | NA |
| 7.2.2 Non-germ cell | NA | 1,677 (2.8) | NA | 121 (4.2) |
| 7.2.2.1 Carcinoma | NA | 1,618 (2.7) | NA | 120 (4.2) |
| 7.2.2.1.1 Adenocarcinoma | NA | 1,565 (2.6) | NA | 120 (4.2) |
| 7.2.2.1.1.1 Clear cell adenocarcinoma | NA | 52 (0.1) | NA | 3 (0.1) |
| 7.2.2.1.1.2 Cystadenocarcinoma | NA | 1,140 (1.9) | NA | 79 (2.7) |
| 7.2.2.1.1.4 Mucinous adenocarcinoma | NA | 173 (0.3) | NA | 9 (0.3) |
| 7.2.2.1.1.5 Endometrioid | NA | 96 (0.2) | NA | 13 (0.5) |
| 7.2.2.1.1.6 Other adenocarcinoma | NA | 104 (0.2) | NA | 16 (0.6) |
| 7.2.2.1.2 Other carcinoma | NA | 53 (0.1) | NA | NA |
| 7.2.2.2 Sex cord and other specialized gonadal | NA | 59 (0.1) | NA | 1 (0.0) |
| 7.3 Germ cell and trophoblastic, CNS | 108 (0.3) | 19 (0.0) | NA | NA |
| 7.4 Germ cell and trophoblastic excluding CNS, ovary, testis | 250 (0.6) | 189 (0.3) | 3 (0.2) | NA |
| 7.4.1 Germ cell tumours including non-gestational Trophoblastic tumours | 168 (0.4) | 5 (0.0) | 3 (0.2) | NA |
| 7.4.2 Gestational Trophoblastic tumours | 82 (0.2) | 184 (0.3) | NA | NA |
| 7.5 Non-germ cell specified tumours excluding CNS, ovary, testis | NA | 2 (0.0) | NA | NA |
| 7.6 Fibroepithelial including Brenner, excluding breast phyllodes | NA | 5 (0.0) | NA | NA |
| **8. Melanoma, malignant** | **5,895 (14.8)** | **11,052 (18.5)** | **133 (9.0)** | **224 (7.8)** |
| 8.1 Superficial spreading/low cumulative sun damage melanoma | 3,729 (9.3) | 7,663 (12.9) | 94 (6.4) | 157 (5.5) |
| 8.2 Nodular melanoma | 662 (1.7) | 918 (1.5) | 10 (0.7) | 24 (0.8) |
| 8.3 Other malignant | 1,504 (3.8) | 2,471 (4.1) | 29 (2.0) | 43 (1.5) |
| **9. Carcinomas** | **7,272 (18.2)** | **35,341 (59.3)** | **908 (61.7)** | **2,125 (74.0)** |
| 9.1 Thyroid carcinoma | 820 (2.1) | 2,670 (4.5) | 38 (2.6) | 80 (2.8) |
| 9.1.1 Medullary | 95 (0.2) | 111 (0.2) | 2 (0.1) | 2 (0.1) |
| 9.1.2 Hurthle cell carcinoma | 15 (0.0) | 28 (0.0) | NA | 4 (0.1) |
| 9.1.3 Papillary | 476 (1.2) | 1,571 (2.6) | 28 (1.9) | 54 (1.9) |
| 9.1.4 Follicular | 86 (0.2) | 374 (0.6) | 2 (0.1) | 7 (0.2) |
| 9.1.5 Papillary with follicular variant | 138 (0.3) | 565 (0.9) | 6 (0.4) | 12 (0.4) |
| 9.1.6 Other | 10 (0.0) | 21 (0.0) | NA | 1 (0.0) |
| 9.2 Other carcinoma of head and neck | 1,072 (2.7) | 782 (1.3) | 105 (7.1) | 88 (3.1) |
| 9.2.1 Nasopharyngeal carcinoma | 167 (0.4) | 94 (0.2) | 3 (0.2) | 2 (0.1) |
| 9.2.1.1 Nasopharyngeal carcinoma, squamous | 75 (0.2) | 46 (0.1) | 2 (0.1) | 2 (0.1) |
| 9.2.1.2 Nasopharyngeal carcinoma, other | 92 (0.2) | 48 (0.1) | 1 (0.1) | NA |
| 9.2.2 Oral cavity, lip, and pharynx | 573 (1.4) | 348 (0.6) | 82 (5.6) | 59 (2.1) |
| 9.2.2.1 Oral cavity, lip, and pharynx, squamous | 485 (1.2) | 247 (0.4) | 79 (5.4) | 56 (1.9) |
| 9.2.2.2 Oral cavity, lip, and pharynx, mucoepidermoid | 52 (0.1) | 58 (0.1) | 2 (0.1) | 2 (0.1) |
| 9.2.2.3 Oral cavity, lip, and pharynx, other | 36 (0.1) | 43 (0.1) | 1 (0.1) | 1 (0.0) |
| 9.2.3 Salivary gland | 160 (0.4) | 237 (0.4) | 6 (0.4) | 8 (0.3) |
| 9.2.3.1 Salivary gland, acinar | 54 (0.1) | 75 (0.1) | 1 (0.1) | 2 (0.1) |
| 9.2.3.2 Salivary gland, other malignant | 106 (0.3) | 162 (0.3) | 5 (0.3) | 6 (0.2) |
| 9.2.4 Other carcinoma of head and neck | 172 (0.4) | 103 (0.2) | 14 (1.0) | 19 (0.7) |
| 9.3 Carcinoma of gastrointestinal tract | 2,924 (7.3) | 3,048 (5.1) | 320 (21.8) | 346 (12.0) |
| 9.3.1 Carcinoma of oesophagus | 148 (0.4) | 52 (0.1) | 38 (2.6) | 33 (1.1) |
| 9.3.2 Carcinoma of stomach | 384 (1.0) | 339 (0.6) | 47 (3.2) | 31 (1.1) |
| 9.3.2.1 Stomach, neuroendocrine | 17 (0.0) | 21 (0.0) | 1 (0.1) | 1 (0.0) |
| 9.3.2.1.1 Neuroendocrine tumour (NET) | 15 (0.0) | 20 (0.0) | 1 (0.1) | 1 (0.0) |
| 9.3.2.1.2 Neuroendocrine carcinoma (NEC) | 2 (0.0) | 1 (0.0) | NA | NA |
| 9.3.2.2 Stomach, signet ring | 118 (0.3) | 154 (0.3) | 12 (0.8) | 6 (0.2) |
| 9.3.2.3 Stomach, other adenocarcinoma | 246 (0.6) | 161 (0.3) | 33 (2.2) | 24 (0.8) |
| 9.3.2.4 Stomach, other invasive | 3 (0.0) | 3 (0.0) | 1 (0.1) | NA |
| 9.3.3 Carcinoma of small intestine | 68 (0.2) | 72 (0.1) | 14 (1.0) | 14 (0.5) |
| 9.3.3.1 Small intestine, neuroendocrine | 30 (0.1) | 38 (0.1) | 4 (0.3) | 7 (0.2) |
| 9.3.3.1.1 NET | 30 (0.1) | 37 (0.1) | 4 (0.3) | 7 (0.2) |
| 9.3.3.1.2 NEC | NA | 1 (0.0) | NA | NA |
| 9.3.3.2 Small intestine, other | 38 (0.1) | 34 (0.1) | 10 (0.7) | 7 (0.2) |
| 9.3.4 Carcinoma of colon | 1,256 (3.1) | 1,550 (2.6) | 77 (5.2) | 120 (4.2) |
| 9.3.4.1 Appendix | 309 (0.8) | 572 (1.0) | 4 (0.3) | 7 (0.2) |
| 9.3.4.1.1 NET | 281 (0.7) | 518 (0.9) | 3 (0.2) | 6 (0.2) |
| 9.3.4.1.3 other | 28 (0.1) | 54 (0.1) | 1 (0.1) | 1 (0.0) |
| 9.3.4.2 Colon excluding appendix | 947 (2.4) | 978 (1.6) | 73 (5.0) | 113 (3.9) |
| 9.3.4.2.1 Colon excluding appendix, neuroendocrine | 18 (0.0) | 19 (0.0) | 1 (0.1) | 2 (0.1) |
| 9.3.4.2.1.1 NET | 13 (0.0) | 17 (0.0) | 1 (0.1) | 1 (0.0) |
| 9.3.4.2.1.2 NEC | 5 (0.0) | 2 (0.0) | NA | 1 (0.0) |
| 9.3.4.2.2 Colon excluding appendix, adenocarcinoma | 925 (2.3) | 955 (1.6) | 72 (4.9) | 111 (3.9) |
| 9.3.4.2.3 Colon excluding appendix, other | 4 (0.0) | 4 (0.0) | NA | NA |
| 9.3.5 Carcinoma of rectum | 721 (1.8) | 660 (1.1) | 53 (3.6) | 72 (2.5) |
| 9.3.5.1 Rectum, neuroendocrine | 61 (0.2) | 81 (0.1) | 2 (0.1) | 2 (0.1) |
| 9.3.5.1.1 NET | 61 (0.2) | 77 (0.1) | 2 (0.1) | 1 (0.0) |
| 9.3.5.1.2 NEC | NA | 4 (0.0) | NA | 1 (0.0) |
| 9.3.5.2 Rectum, adenocarcinoma | 658 (1.6) | 575 (1.0) | 50 (3.4) | 70 (2.4) |
| 9.3.5.3 Rectum, other | 2 (0.0) | 4 (0.0) | 1 (0.1) | NA |
| 9.3.6 Carcinoma of anus | 64 (0.2) | 67 (0.1) | 18 (1.2) | 13 (0.5) |
| 9.3.6.1 Anus, squamous | 51 (0.1) | 58 (0.1) | 17 (1.2) | 13 (0.5) |
| 9.3.6.2 Anus, other | 13 (0.0) | 9 (0.0) | 1 (0.1) | NA |
| 9.3.7 Carcinoma of liver and intrahepatic bile ducts (IBD) | 77 (0.2) | 79 (0.1) | 15 (1.0) | 7 (0.2) |
| 9.3.7.1 Liver and IBD, cholangiocarcinoma | 7 (0.0) | 15 (0.0) | 2 (0.1) | 1 (0.0) |
| 9.3.7.2 Liver and IBD, hepatocellular carcinoma | 62 (0.2) | 60 (0.1) | 9 (0.6) | 4 (0.1) |
| 9.3.7.3 Liver and IBD, other | 8 (0.0) | 4 (0.0) | 4 (0.3) | 2 (0.1) |
| 9.3.8 Carcinoma of gallbladder and other extrahepatic biliary | 72 (0.2) | 61 (0.1) | 10 (0.7) | 8 (0.3) |
| 9.3.9 Carcinoma of pancreas | 126 (0.3) | 162 (0.3) | 45 (3.1) | 48 (1.7) |
| 9.3.9.1 Pancreas, neuroendocrine | 57 (0.1) | 81 (0.1) | 6 (0.4) | 13 (0.5) |
| 9.3.9.1.1 NET | 38 (0.1) | 59 (0.1) | 5 (0.3) | 13 (0.5) |
| 9.3.9.1.2 NEC | 6 (0.0) | 2 (0.0) | 1 (0.1) | NA |
| 9.3.9.1.3 Neuroendocrine, other | 13 (0.0) | 20 (0.0) | NA | NA |
| 9.3.9.2 Pancreas, adenocarcinoma | 66 (0.2) | 78 (0.1) | 38 (2.6) | 33 (1.1) |
| 9.3.9.3 Pancreas, other | 3 (0.0) | 3 (0.0) | 1 (0.1) | 2 (0.1) |
| 9.3.10 Other carcinoma of gastrointestinal tract | 8 (0.0) | 6 (0.0) | 3 (0.2) | NA |
| 9.4 Carcinoma of lung, bronchus, and trachea | 702 (1.8) | 910 (1.5) | 143 (9.7) | 349 (12.1) |
| 9.4.1 Small cell carcinoma, NEC | 79 (0.2) | 81 (0.1) | 19 (1.3) | 41 (1.4) |
| 9.4.2 Non-small cell carcinoma | 623 (1.6) | 829 (1.4) | 124 (8.4) | 308 (10.7) |
| 9.4.2.1 Non-small cell, adenocarcinoma | 257 (0.6) | 371 (0.6) | 67 (4.6) | 202 (7.0) |
| 9.4.2.2 Non-small cell, neuroendocrine | 159 (0.4) | 254 (0.4) | 2 (0.1) | 12 (0.4) |
| 9.4.2.2.1 Non-small cell NET | 142 (0.4) | 238 (0.4) | 1 (0.1) | 4 (0.1) |
| 9.4.2.2.2 Non-small cell NEC | 17 (0.0) | 16 (0.0) | 1 (0.1) | 8 (0.3) |
| 9.4.2.3 Non-small cell, other | 207 (0.5) | 204 (0.3) | 55 (3.7) | 94 (3.3) |
| 9.5 Carcinoma of skin (if collected) | 473 (1.2) | 541 (0.9) | 98 (6.7) | 124 (4.3) |
| 9.6 Carcinoma of breast | 36 (0.1) | 19,572 (32.8) | 6 (0.4) | 772 (26.9) |
| 9.6.1 Breast, infiltrating duct | 28 (0.1) | 16,066 (27.0) | 5 (0.3) | 646 (22.5) |
| 9.6.2 Breast, adenocarcinoma | 6 (0.0) | 1,511 (2.5) | 1 (0.1) | 48 (1.7) |
| 9.6.3 Breast, lobular | NA | 890 (1.5) | NA | 53 (1.8) |
| 9.6.4 Breast, phyllodes | NA | 65 (0.1) | NA | 1 (0.0) |
| 9.6.5 Breast, medullary | NA | 450 (0.8) | NA | 9 (0.3) |
| 9.6.6 Breast, Paget | 1 (0.0) | 98 (0.2) | NA | 4 (0.1) |
| 9.6.7 Breast, ductal | NA | 36 (0.1) | NA | 2 (0.1) |
| 9.6.8 Breast, metaplastic | NA | 72 (0.1) | NA | 2 (0.1) |
| 9.6.9 Breast, inflammatory | NA | 3 (0.0) | NA | NA |
| 9.6.10 Breast, other | 1 (0.0) | 381 (0.6) | NA | 7 (0.2) |
| 9.7 Carcinoma of genital sites excluding ovary and testis | 86 (0.2) | 6,919 (11.6) | 78 (5.3) | 230 (8.0) |
| 9.7.1 Carcinoma of uterine cervix | NA | 6,266 (10.5) | NA | 84 (2.9) |
| 9.7.1.1 Cervix, squamous | NA | 4,517 (7.6) | NA | 57 (2.0) |
| 9.7.1.2 Cervix, adenosquamous | NA | 219 (0.4) | NA | 2 (0.1) |
| 9.7.1.3 Cervix, adenocarcinoma | NA | 1,223 (2.1) | NA | 24 (0.8) |
| 9.7.1.4 Cervix, other | NA | 307 (0.5) | NA | 1 (0.0) |
| 9.7.2 Corpus uteri | NA | 307 (0.5) | NA | 100 (3.5) |
| 9.7.2.1 Corpus uteri, adenocarcinoma | NA | 243 (0.4) | NA | 94 (3.3) |
| 9.7.2.1.1 Corpus uteri, endometrioid | NA | 130 (0.2) | NA | 79 (2.7) |
| 9.7.2.1.2 Corpus uteri, other adenocarcinoma | NA | 113 (0.2) | NA | 15 (0.5) |
| 9.7.2.2 Corpus uteri, other | NA | 64 (0.1) | NA | 6 (0.2) |
| 9.7.3 Carcinoma of vulva and vagina | NA | 328 (0.6) | NA | 36 (1.3) |
| 9.7.4 Carcinoma of penis | 66 (0.2) | NA | 5 (0.3) | NA |
| 9.7.5 Carcinoma of prostate | 16 (0.0) | NA | 72 (4.9) | NA |
| 9.7.6 Other genital | 4 (0.0) | 18 (0.0) | 1 (0.1) | 10 (0.3) |
| 9.8 Carcinoma of urinary tract | 881 (2.2) | 555 (0.9) | 95 (6.5) | 77 (2.7) |
| 9.8.1 Carcinoma of kidney | 593 (1.5) | 382 (0.6) | 57 (3.9) | 39 (1.4) |
| 9.8.1.1 Kidney, adenocarcinoma | 589 (1.5) | 382 (0.6) | 57 (3.9) | 38 (1.3) |
| 9.8.1.1.1 Kidney, renal cell | 534 (1.3) | 340 (0.6) | 45 (3.1) | 36 (1.3) |
| 9.8.1.1.2 Kidney, other adenocarcinoma | 55 (0.1) | 42 (0.1) | 12 (0.8) | 2 (0.1) |
| 9.8.1.2 Kidney, other | 4 (0.0) | NA | NA | 1 (0.0) |
| 9.8.2 Carcinoma of bladder | 253 (0.6) | 155 (0.3) | 31 (2.1) | 29 (1.0) |
| 9.8.2.1 Urinary bladder, transitional cell carcinoma | 219 (0.5) | 116 (0.2) | 28 (1.9) | 23 (0.8) |
| 9.8.2.2 Urinary bladder, other carcinoma | 34 (0.1) | 39 (0.1) | 3 (0.2) | 6 (0.2) |
| 9.8.3 Other urinary | 35 (0.1) | 18 (0.0) | 7 (0.5) | 9 (0.3) |
| 9.9 Other invasive carcinomas | 278 (0.7) | 344 (0.6) | 25 (1.7) | 59 (2.1) |
| 9.9.1 Adrenocortical carcinoma | 22 (0.1) | 46 (0.1) | 2 (0.1) | 1 (0.0) |
| 9.9.2 Unknown primary | 177 (0.4) | 201 (0.3) | 21 (1.4) | 46 (1.6) |
| 9.9.3 Thymic carcinoma | 53 (0.1) | 41 (0.1) | 2 (0.1) | 1 (0.0) |
| 9.9.4 Carcinoma of other and ill-defined sites | 26 (0.1) | 56 (0.1) | NA | 11 (0.4) |
| **10. Miscellaneous specified neoplasms** | **102 (0.3)** | **137 (0.2)** | **7 (0.5)** | **11 (0.4)** |
| 10.1 Other paediatric and embryonal tumours | 35 (0.1) | 51 (0.1) | 1 (0.1) | 1 (0.0) |
| 10.1.1 Wilms tumour | 8 (0.0) | 11 (0.0) | NA | NA |
| 10.1.2 Olfactory and non-CNS neuroblastoma | 21 (0.1) | 30 (0.1) | NA | 1 (0.0) |
| 10.1.3 Other embryonal non-CNS tumours | 6 (0.0) | 10 (0.0) | 1 (0.1) | NA |
| 10.2 Other specified tumours | 67 (0.2) | 86 (0.1) | 6 (0.4) | 10 (0.3) |
| 10.2.1 Paraganglioma, non-CNS | 32 (0.1) | 22 (0.0) | 1 (0.1) | NA |
| 10.2.2 Other specified neoplasms | 35 (0.1) | 64 (0.1) | 5 (0.3) | 10 (0.3) |
| **11. Unspecified malignant neoplasms except CNS** | **85 (0.2)** | **51 (0.1)** | **23 (1.6)** | **23 (0.8)** |

Abbreviation: NOS=Not otherwise specified, CNS=Central Nervous System, NA=Not Applicable, NA=Not Applicable.

^a^ Percentages might not add-up to 100% due to rounding.

**Supplementary Table S1B.** Population, tumour and treatment characteristics of six-month adolescent and young adult (AYA, aged 15-39 years) cancer survivors diagnosed with first and second primary malignant cancer in the Netherlands between 1989-2018. Cancer types were grouped according to the AYA-specific classification scheme developed by Barr and colleagues (2020). This Table presents outcomes from the sensitivity analysis.

|  | **First primary cancers** | | | **Second primary cancers** | |
| --- | --- | --- | --- | --- | --- |
|  | **Males** | **Females** | | **Males** | **Females** |
|  | **n= (%)** | **n= (%)** | | **n= (%)** | **n= (%)** |
| **Total^a^** | 39,774 (100) | 59,370 (100) | | 2,060 (100) | 4,634 (100) |
| **Median age (IQR)** | 31.0 (26.0-36.0) | | 34.0 (29.0-37.0) | 43.0 (36.0-51.0) | 45.0 (39.0-51.0) |
| **Age at diagnosis (years)^b^** |  |  | |  |  |
| 15−19 | 3,219 (8.1) | 2,578 (4.3) | | 12 (0.6) | 4 (0.1) |
| 20−24 | 5,226 (13.1) | 4,436 (7.5) | | 47 (2.3) | 43 (0.9) |
| 25−29 | 7,845 (19.7) | 8,615 (14.5) | | 143 (6.9) | 112 (2.4) |
| 30−34 | 10,260 (25.8) | 16,562 (27.9) | | 212 (10.3) | 352 (7.6) |
| 35−39 | 13,224 (33.2) | 27,179 (45.8) | | 360 (17.5) | 737 (15.9) |
| 40−44 | NA | NA | | 367 (17.8) | 1,048 (22.6) |
| 45−49 | NA | NA | | 331 (16.1) | 958 (20.7) |
| 50−54 | NA | NA | | 279 (13.5) | 687 (14.8) |
| 55−59 | NA | NA | | 201 (9.8) | 452 (9.8) |
| 60−64 | NA | NA | | 95 (4.6) | 216 (4.7) |
| 65−68 | NA | NA | | 13 (0.6) | 25 (0.5) |
| **Tumour stage (TNM, Figo and Ann arbor)** |  |  | |  |  |
| stage I | 17,048 (42.9) | 26,925 (45.4) | | 920 (44.7) | 2,128 (45.9) |
| stage II | 6,283 (15.8) | 15,647 (26.4) | | 253 (12.3) | 961 (20.7) |
| stage III | 4,641 (11.7) | 5,964 (10.0) | | 245 (11.9) | 526 (11.4) |
| stage IV | 3,264 (8.2) | 3,369 (5.7) | | 307 (14.9) | 599 (12.9) |
| Other/unknown | 8,538 (21.5) | 7,465 (12.6) | | 335 (16.3) | 420 (9.1) |
| **Period of diagnosis** |  |  | |  |  |
| 1989-1998 | 11,999 (30.2) | 18,952 (31.9) | | 149 (7.2) | 420 (9.1) |
| 1999-2008 | 13,772 (34.6) | 20,577 (34.7) | | 604 (29.3) | 1,367 (29.5) |
| 2009-2018 | 14,003 (35.2) | 19,841 (33.4) | | 1,307 (63.4) | 2,847 (61.4) |
| **Years of follow-up (years)^c^** |  |  | |  |  |
| 0-4 | 14,290 (35.9) | 20,374 (34.3) | | NA | NA |
| 5-9 | 7,827 (19.7) | 11,833 (19.9) | | NA | NA |
| 10-14 | 6,183 (15.5) | 9,332 (15.7) | | NA | NA |
| 15-19 | 4,931 (12.4) | 7,546 (12.7) | | NA | NA |
| 20-25 | 4,578 (11.5) | 7,162 (12.1) | | NA | NA |
| ≥26 | 1,965 (4.9) | 3,123 (5.3) | | NA | NA |
| **Cancer types** |  |  | |  |  |
| **1. Leukaemia’s and related disorders** | 2,322 (5.8) | 1,954 (3.3) | | 111 (5.4) | 102 (2.2) |
| 1.1 Acute lymphoblastic leukaemia | 660 (1.7) | 407 (0.7) | | 14 (0.7) | 5 (0.1) |
| 1.2 Acute myeloid leukaemia | 621 (1.6) | 677 (1.1) | | 43 (2.1) | 42 (0.9) |
| 1.2.1 Acute promyelocytic leukaemia | 73 (0.2) | 103 (0.2) | | 2 (0.1) | 1 (0.0) |
| 1.2.2 Other acute myeloid leukaemia | 548 (1.4) | 574 (1.0) | | 41 (2.0) | 41 (0.9) |
| 1.3 Chronic myeloid leukaemia | 433 (1.1) | 264 (0.4) | | 12 (0.6) | 11 (0.2) |
| 1.4 Chronic lymphocytic leukaemia | 114 (0.3) | 45 (0.1) | | 10 (0.5) | 12 (0.3) |
| 1.5 Polycythaemia vera | 75 (0.2) | 71 (0.1) | | 1 (0.0) | 1 (0.0) |
| 1.6 Essential thrombocythemia | 145 (0.4) | 284 (0.5) | | 6 (0.3) | 9 (0.2) |
| 1.7 Primary myelofibrosis | 20 (0.1) | 19 (0.0) | | 1 (0.0) | 1 (0.0) |
| 1.8 Myelodysplastic syndrome (MDS) | 84 (0.2) | 98 (0.2) | | 17 (0.8) | 15 (0.3) |
| 1.9 Other and unspecified leukaemia’s and related disorders | 170 (0.4) | 89 (0.1) | | 7 (0.3) | 6 (0.1) |
| 1.9.1 Hairy cell leukaemia | 82 (0.2) | 13 (0.0) | | 4 (0.2) | 1 (0.0) |
| 1.9.2 Other lymphocytic/lymphoblastic leukaemia | 13 (0.0) | 13 (0.0) | | 1 (0.0) | 2 (0.0) |
| 1.9.3 Other myeloid leukaemia | 17 (0.0) | 12 (0.0) | | 1 (0.0) | 1 (0.0) |
| 1.9.4 Leukaemia’s of mixed phenotype | 12 (0.0) | 13 (0.0) | | NA | NA |
| 1.9.5 Mast cell diseases | 13 (0.0) | 12 (0.0) | | NA | 2 (0.0) |
| 1.9.6 Other | 33 (0.1) | 26 (0.0) | | 1 (0.0) | NA |
| **2. Lymphomas** | 6,348 (16.0) | 4,613 (7.8) | | 146 (7.1) | 106 (2.3) |
| 2.1 Non-Hodgkin lymphomas | 2,763 (6.9) | 1,693 (2.9) | | 97 (4.7) | 89 (1.9) |
| 2.1.1 Lymphoblastic | 166 (0.4) | 40 (0.1) | | 1 (0.0) | 2 (0.0) |
| 2.1.2 Burkitt | 172 (0.4) | 57 (0.1) | | 3 (0.1) | NA |
| 2.1.3 Diffuse large B-cell (DLBCL) | 1,097 (2.8) | 649 (1.1) | | 57 (2.8) | 35 (0.8) |
| 2.1.4 Primary mediastinal large B-cell excluded from DLBCL | 115 (0.3) | 140 (0.2) | | 2 (0.1) | 1 (0.0) |
| 2.1.5 Anaplastic T-cell and null-cell excluding NK/T-cell | 429 (1.1) | 273 (0.5) | | 8 (0.4) | 10 (0.2) |
| 2.1.6 Follicular | 416 (1.0) | 289 (0.5) | | 9 (0.4) | 24 (0.5) |
| 2.1.7 NK/T-cell (excluded from anaplastic T-cell) | 14 (0.0) | 8 (0.0) | | 1 (0.0) | NA |
| 2.1.8 MALT (Mucosa-associated lymphoid tissue) | 179 (0.5) | 131 (0.2) | | 3 (0.1) | 13 (0.3) |
| 2.1.9 Other non-Hodgkin lymphoma NOS | 175 (0.4) | 106 (0.2) | | 13 (0.6) | 4 (0.1) |
| 2.2 Hodgkin lymphoma | 3,211 (8.1) | 2,705 (4.6) | | 31 (1.5) | 6 (0.1) |
| 2.2.1 Hodgkin NLP | 249 (0.6) | 76 (0.1) | | 4 (0.2) | 1 (0.0) |
| 2.2.2 Hodgkin classic, other | 2,962 (7.4) | 2,629 (4.4) | | 27 (1.3) | 5 (0.1) |
| 2.3 Myeloma | 180 (0.5) | 106 (0.2) | | 7 (0.3) | 10 (0.2) |
| 2.4 Cutaneous lymphomas | 26 (0.1) | 14 (0.0) | | 1 (0.0) | NA |
| 2.5 Other B-cell and T-cell lymphomas | 103 (0.3) | 56 (0.1) | | 6 (0.3) | 1 (0.0) |
| 2.6 Other lymphomas, specified and unspecified | 65 (0.2) | 39 (0.1) | | 4 (0.2) | NA |
| 2.6.1 Histiocytic and dendritic cell neoplasms | 45 (0.1) | 27 (0.0) | | NA | NA |
| 2.6.2 Lymphoma NOS | 20 (0.1) | 12 (0.0) | | 4 (0.2) | NA |
| **3. CNS and other intracranial and intraspinal neoplasms** | 2,556 (6.4) | 1,759 (3.0) | | 58 (2.8) | 54 (1.2) |
| 3.1 Astroglial and related neoplasms | 2,257 (5.7) | 1,544 (2.6) | | 53 (2.6) | 47 (1.0) |
| 3.1.1 Oligodendriogliomas | 511 (1.3) | 338 (0.6) | | 6 (0.3) | 12 (0.3) |
| 3.1.1.2 Oligodendrioglioma, invasive | 511 (1.3) | 338 (0.6) | | 6 (0.3) | 12 (0.3) |
| 3.1.2 Glioblastomas/gliofibromas | 346 (0.9) | 197 (0.3) | | 25 (1.2) | 18 (0.4) |
| 3.1.2.2 Glioblastoma, invasive | 346 (0.9) | 197 (0.3) | | 25 (1.2) | 18 (0.4) |
| 3.1.3 Ependymomas | 169 (0.4) | 136 (0.2) | | 2 (0.1) | 2 (0.0) |
| 3.1.3.2 Ependymoma, invasive | 169 (0.4) | 136 (0.2) | | 2 (0.1) | 2 (0.0) |
| 3.1.4 Other astrocytoma/astroglial neoplasms | 1,231 (3.1) | 873 (1.5) | | 20 (1.0) | 15 (0.3) |
| 3.1.4.1 Pilocytic astrocytoma | 70 (0.2) | 43 (0.1) | | NA | NA |
| 3.1.4.3 Other astrocytoma/astroglial, invasive | 1,161 (2.9) | 830 (1.4) | | 20 (1.0) | 15 (0.3) |
| 3.2 Medulloblastoma and other invasive embryonal CNS tumours | 137 (0.3) | 88 (0.1) | | NA | 2 (0.0) |
| 3.3 Neuroblastomas/ganglioneuromas | 3 (0.0) | 5 (0.0) | | NA | NA |
| 3.3.2 Neuroblastoma/ganglioneuroblastoma, invasive | 3 (0.0) | 5 (0.0) | | NA | NA |
| 3.4 Neuronal and mixed neuronal-glial neoplasms | 9 (0.0) | 3 (0.0) | | NA | NA |
| 3.4.2 Neuronal and mixed neuronal-glial, invasive | 9 (0.0) | 3 (0.0) | | NA | NA |
| 3.5 Meningiomas | 15 (0.0) | 14 (0.0) | | 2 (0.1) | NA |
| 3.5.2 Meningioma, invasive | 15 (0.0) | 14 (0.0) | | 2 (0.1) | NA |
| 3.6 Choroid plexus neoplasms | 1 (0.0) | 2 (0.0) | | NA | NA |
| 3.6.2 Choroid plexus, invasive | 1 (0.0) | 2 (0.0) | | NA | NA |
| 3.8 Pituitary neoplasms | 4 (0.0) | NA | | NA | NA |
| 3.8.2 Pituitary, invasive | 4 (0.0) | NA | | NA | NA |
| 3.9 Pineal neoplasms | 16 (0.0) | 17 (0.0) | | NA | NA |
| 3.9.2 Pineal, invasive | 16 (0.0) | 17 (0.0) | | NA | NA |
| 3.10 Other and unspecified CNS neoplasms | 114 (0.3) | 86 (0.1) | | 3 (0.1) | 5 (0.1) |
| 3.10.2 Other and unspecified CNS, invasive | 114 (0.3) | 86 (0.1) | | 3 (0.1) | 5 (0.1) |
| **4. Sarcomas** | 2,470 (6.2) | 2,229 (3.8) | | 60 (2.9) | 110 (2.4) |
| 4.1 Osteosarcoma | 333 (0.8) | 221 (0.4) | | 4 (0.2) | 9 (0.2) |
| 4.2 Chondrosarcoma | 339 (0.9) | 339 (0.6) | | 8 (0.4) | 26 (0.6) |
| 4.3 Ewing family of tumours | 275 (0.7) | 177 (0.3) | | 3 (0.1) | 4 (0.1) |
| 4.3.1 Bone | 193 (0.5) | 104 (0.2) | | 2 (0.1) | 1 (0.0) |
| 4.3.2 Soft tissue | 82 (0.2) | 73 (0.1) | | 1 (0.0) | 3 (0.1) |
| 4.4 Fibromatous neoplasms | 532 (1.3) | 574 (1.0) | | 9 (0.4) | 15 (0.3) |
| 4.4.1 Myxofibrosarcoma | 34 (0.1) | 39 (0.1) | | 2 (0.1) | 6 (0.1) |
| 4.4.2 Malignant fibrous histiocytomac | 69 (0.2) | 50 (0.1) | | 1 (0.0) | 2 (0.0) |
| 4.4.3 Other fibromatous neoplasms | 429 (1.1) | 485 (0.8) | | 6 (0.3) | 7 (0.2) |
| 4.5 Liposarcoma | 208 (0.5) | 203 (0.3) | | 2 (0.1) | 9 (0.2) |
| 4.6 Synovial sarcoma | 158 (0.4) | 126 (0.2) | | 2 (0.1) | 5 (0.1) |
| 4.7 Leiomyosarcoma | 144 (0.4) | 171 (0.3) | | 3 (0.1) | 10 (0.2) |
| 4.8 Rhabdomyosarcoma | 130 (0.3) | 74 (0.1) | | 1 (0.0) | 3 (0.1) |
| 4.9 Gastrointestinal stromal tumour, malignant | 71 (0.2) | 58 (0.1) | | 4 (0.2) | 4 (0.1) |
| 4.10 Spindle cell sarcoma | 15 (0.0) | 12 (0.0) | | 3 (0.1) | 2 (0.0) |
| 4.11 Epithelioid sarcoma | 40 (0.1) | 22 (0.0) | | NA | 1 (0.0) |
| 4.12 Desmoplastic small round cell tumour | 11 (0.0) | 4 (0.0) | | NA | NA |
| 4.13 Chordoma | 30 (0.1) | 26 (0.0) | | 1 (0.0) | 1 (0.0) |
| 4.14 Giant cell sarcoma | 19 (0.0) | 12 (0.0) | | 5 (0.2) | 5 (0.1) |
| 4.15 Other soft tissue sarcomas | 113 (0.3) | 144 (0.2) | | 12 (0.6) | 13 (0.3) |
| 4.16 Other bone tumours | 52 (0.1) | 66 (0.1) | | 3 (0.1) | 3 (0.1) |
| **5. Blood and lymphatic vessel tumours** | 650 (1.6) | 120 (0.2) | | 16 (0.8) | 12 (0.3) |
| 5.2 Malignant blood and lymphatic vessel tumours, all sites | 650 (1.6) | 120 (0.2) | | 16 (0.8) | 12 (0.3) |
| 5.2.1 Kaposi sarcoma | 591 (1.5) | 44 (0.1) | | 6 (0.3) | NA |
| 5.2.2 Other | 59 (0.1) | 76 (0.1) | | 10 (0.5) | 12 (0.3) |
| **6. Nerve sheath tumours** | 123 (0.3) | 105 (0.2) | | 5 (0.2) | 17 (0.4) |
| 6.2 Malignant | 123 (0.3) | 105 (0.2) | | 5 (0.2) | 17 (0.4) |
| 6.2.1 MPNST (Malignant peripheral nerve sheath tumour) | 90 (0.2) | 77 (0.1) | | 5 (0.2) | 17 (0.4) |
| 6.2.1.1 CNS | 2 (0.0) | 3 (0.0) | | NA | NA |
| 6.2.1.2 Peripheral | 88 (0.2) | 74 (0.1) | | 5 (0.2) | 17 (0.4) |
| 6.2.2 Other | 33 (0.1) | 28 (0.0) | | NA | NA |
| **7. Gonadal and related tumours** | 12,006 (30.2) | 2,250 (3.8) | | 332 (16.1) | 139 (3.0) |
| 7.1 Testis | 11,649 (29.3) | NA | | 329 (16.0) | NA |
| 7.1.1 Germ cell and trophoblastic | 11,617 (29.2) | NA | | 329 (16.0) | NA |
| 7.1.1.1 Seminoma | 5,404 (13.6) | NA | | 218 (10.6) | NA |
| 7.1.1.2 Embryonal carcinoma | 1,681 (4.2) | NA | | 29 (1.4) | NA |
| 7.1.1.3 Endodermal sinus (yolk sac tumour) | 224 (0.6) | NA | | NA | NA |
| 7.1.1.4 Teratoma | 1,464 (3.7) | NA | | 13 (0.6) | NA |
| 7.1.1.5 Mixed germ cell | 2,264 (5.7) | NA | | 63 (3.1) | NA |
| 7.1.1.6 Choriocarcinoma and other trophoblastic | 453 (1.1) | NA | | 4 (0.2) | NA |
| 7.1.1.7 Other | 127 (0.3) | NA | | 2 (0.1) | NA |
| 7.1.2 Non-germ cell | 32 (0.1) | NA | | NA | NA |
| 7.1.2.1 Carcinoma | 6 (0.0) | NA | | NA | NA |
| 7.1.2.2 Sex cord | 26 (0.1) | NA | | NA | NA |
| 7.2 Ovary | NA | 2,035 (3.4) | | NA | 139 (3.0) |
| 7.2.1 Germ cell and trophoblastic | NA | 357 (0.6) | | NA | 5 (0.1) |
| 7.2.1.1 Teratoma | NA | 139 (0.2) | | NA | 3 (0.1) |
| 7.2.1.2 Dysgerminoma | NA | 110 (0.2) | | NA | 1 (0.0) |
| 7.2.1.3 Yolk sac | NA | 61 (0.1) | | NA | NA |
| 7.2.1.4 Mixed germ cell | NA | 26 (0.0) | | NA | 1 (0.0) |
| 7.2.1.5 Other germ cell and trophoblastic | NA | 21 (0.0) | | NA | NA |
| 7.2.2 Non-germ cell | NA | 1,678 (2.8) | | NA | 134 (2.9) |
| 7.2.2.1 Carcinoma | NA | 1,619 (2.7) | | NA | 133 (2.9) |
| 7.2.2.1.1 Adenocarcinoma | NA | 1,566 (2.6) | | NA | 133 (2.9) |
| 7.2.2.1.1.1 Clear cell adenocarcinoma | NA | 52 (0.1) | | NA | 4 (0.1) |
| 7.2.2.1.1.2 Cystadenocarcinoma | NA | 1,141 (1.9) | | NA | 88 (1.9) |
| 7.2.2.1.1.4 Mucinous adenocarcinoma | NA | 173 (0.3) | | NA | 11 (0.2) |
| 7.2.2.1.1.5 Endometrioid | NA | 96 (0.2) | | NA | 14 (0.3) |
| 7.2.2.1.1.6 Other adenocarcinoma | NA | 104 (0.2) | | NA | 16 (0.3) |
| 7.2.2.1.2 Other carcinoma | NA | 53 (0.1) | | NA | NA |
| 7.2.2.2 Sex cord and other specialized gonadal | NA | 59 (0.1) | | NA | 1 (0.0) |
| 7.3 Germ cell and trophoblastic, CNS | 107 (0.3) | 19 (0.0) | | NA | NA |
| 7.4 Germ cell and trophoblastic excluding CNS, ovary, testis | 250 (0.6) | 189 (0.3) | | 3 (0.1) | NA |
| 7.4.1 Germ cell tumours including non-gestational Trophoblastic tumours | 168 (0.4) | 5 (0.0) | | 3 (0.1) | NA |
| 7.4.2 Gestational Trophoblastic tumours | 82 (0.2) | 184 (0.3) | | NA | NA |
| 7.5 Non-germ cell specified tumours excluding CNS, ovary, testis | NA | 2 (0.0) | | NA | NA |
| 7.6 Fibroepithelial including Brenner, excluding breast phyllodes | NA | 5 (0.0) | | NA | NA |
| **8. Melanoma, malignant** | 5,871 (14.8) | 10,970 (18.5) | | 336 (16.3) | 629 (13.6) |
| 8.1 Superficial spreading/low cumulative sun damage melanoma | 3,717 (9.3) | 7,611 (12.8) | | 255 (12.4) | 486 (10.5) |
| 8.2 Nodular melanoma | 654 (1.6) | 909 (1.5) | | 31 (1.5) | 42 (0.9) |
| 8.3 Other malignant | 1,500 (3.8) | 2,450 (4.1) | | 50 (2.4) | 101 (2.2) |
| **9. Carcinomas** | 7,242 (18.2) | 35,184 (59.3) | | 968 (47.0) | 3427 (74.0) |
| 9.1 Thyroid carcinoma | 820 (2.1) | 2,670 (4.5) | | 39 (1.9) | 76 (1.6) |
| 9.1.1 Medullary | 95 (0.2) | 111 (0.2) | | 2 (0.1) | 2 (0.0) |
| 9.1.2 Hurthle cell carcinoma | 15 (0.0) | 28 (0.0) | | NA | 3 (0.1) |
| 9.1.3 Papillary | 476 (1.2) | 1,571 (2.6) | | 29 (1.4) | 51 (1.1) |
| 9.1.4 Follicular | 86 (0.2) | 374 (0.6) | | 2 (0.1) | 7 (0.2) |
| 9.1.5 Papillary with follicular variant | 138 (0.3) | 565 (1.0) | | 6 (0.3) | 12 (0.3) |
| 9.1.6 Other | 10 (0.0) | 21 (0.0) | | NA | 1 (0.0) |
| 9.2 Other carcinoma of head and neck | 1,072 (2.7) | 781 (1.3) | | 119 (5.8) | 102 (2.2) |
| 9.2.1 Nasopharyngeal carcinoma | 167 (0.4) | 94 (0.2) | | 3 (0.1) | 4 (0.1) |
| 9.2.1.1 Nasopharyngeal carcinoma, squamous | 75 (0.2) | 46 (0.1) | | 2 (0.1) | 4 (0.1) |
| 9.2.1.2 Nasopharyngeal carcinoma, other | 92 (0.2) | 48 (0.1) | | 1 (0.0) | NA |
| 9.2.2 Oral cavity, lip, and pharynx | 572 (1.4) | 347 (0.6) | | 93 (4.5) | 69 (1.5) |
| 9.2.2.1 Oral cavity, lip, and pharynx, squamous | 484 (1.2) | 246 (0.4) | | 90 (4.4) | 67 (1.4) |
| 9.2.2.2 Oral cavity, lip, and pharynx, mucoepidermoid | 52 (0.1) | 58 (0.1) | | 2 (0.1) | 1 (0.0) |
| 9.2.2.3 Oral cavity, lip, and pharynx, other | 36 (0.1) | 43 (0.1) | | 1 (0.0) | 1 (0.0) |
| 9.2.3 Salivary gland | 160 (0.4) | 237 (0.4) | | 6 (0.3) | 9 (0.2) |
| 9.2.3.1 Salivary gland, acinar | 54 (0.1) | 75 (0.1) | | 1 (0.0) | 2 (0.0) |
| 9.2.3.2 Salivary gland, other malignant | 106 (0.3) | 162 (0.3) | | 5 (0.2) | 7 (0.2) |
| 9.2.4 Other carcinoma of head and neck | 173 (0.4) | 103 (0.2) | | 17 (0.8) | 20 (0.4) |
| 9.3 Carcinoma of gastrointestinal tract | 2,909 (7.3) | 3,038 (5.1) | | 345 (16.7) | 338 (7.3) |
| 9.3.1 Carcinoma of oesophagus | 148 (0.4) | 52 (0.1) | | 37 (1.8) | 29 (0.6) |
| 9.3.2 Carcinoma of stomach | 384 (1.0) | 339 (0.6) | | 45 (2.2) | 29 (0.6) |
| 9.3.2.1 Stomach, neuroendocrine | 17 (0.0) | 21 (0.0) | | 1 (0.0) | 1 (0.0) |
| 9.3.2.1.1 Neuroendocrine tumour (NET) | 15 (0.0) | 20 (0.0) | | 1 (0.0) | 1 (0.0) |
| 9.3.2.1.2 Neuroendocrine carcinoma (NEC) | 2 (0.0) | 1 (0.0) | | NA | NA |
| 9.3.2.2 Stomach, signet ring | 118 (0.3) | 154 (0.3) | | 11 (0.5) | 6 (0.1) |
| 9.3.2.3 Stomach, other adenocarcinoma | 246 (0.6) | 161 (0.3) | | 32 (1.6) | 22 (0.5) |
| 9.3.2.4 Stomach, other invasive | 3 (0.0) | 3 (0.0) | | 1 (0.0) | NA |
| 9.3.3 Carcinoma of small intestine | 68 (0.2) | 72 (0.1) | | 14 (0.7) | 13 (0.3) |
| 9.3.3.1 Small intestine, neuroendocrine | 30 (0.1) | 38 (0.1) | | 4 (0.2) | 7 (0.2) |
| 9.3.3.1.1 NET | 30 (0.1) | 37 (0.1) | | 4 (0.2) | 7 (0.2) |
| 9.3.3.1.2 NEC | NA | 1 (0.0) | | NA | NA |
| 9.3.3.2 Small intestine, other | 38 (0.1) | 34 (0.1) | | 10 (0.5) | 6 (0.1) |
| 9.3.4 Carcinoma of colon | 1,241 (3.1) | 1,540 (2.6) | | 105 (5.1) | 129 (2.8) |
| 9.3.4.1 Appendix | 309 (0.8) | 571 (1.0) | | 4 (0.2) | 7 (0.2) |
| 9.3.4.1.1 NET | 281 (0.7) | 518 (0.9) | | 3 (0.1) | 6 (0.1) |
| 9.3.4.1.3 other | 28 (0.1) | 53 (0.1) | | 1 (0.0) | 1 (0.0) |
| 9.3.4.2 Colon excluding appendix | 932 (2.3) | 969 (1.6) | | 101 (4.9) | 122 (2.6) |
| 9.3.4.2.1 Colon excluding appendix, neuroendocrine | 18 (0.0) | 19 (0.0) | | 1 (0.0) | 2 (0.0) |
| 9.3.4.2.1.1 NET | 13 (0.0) | 17 (0.0) | | 1 (0.0) | 1 (0.0) |
| 9.3.4.2.1.2 NEC | 5 (0.0) | 2 (0.0) | | NA | 1 (0.0) |
| 9.3.4.2.2 Colon excluding appendix, adenocarcinoma | 910 (2.3) | 946 (1.6) | | 100 (4.9) | 120 (2.6) |
| 9.3.4.2.3 Colon excluding appendix, other | 4 (0.0) | 4 (0.0) | | NA | NA |
| 9.3.5 Carcinoma of rectum | 720 (1.8) | 660 (1.1) | | 53 (2.6) | 65 (1.4) |
| 9.3.5.1 Rectum, neuroendocrine | 61 (0.2) | 81 (0.1) | | 2 (0.1) | 2 (0.0) |
| 9.3.5.1.1 NET | 61 (0.2) | 77 (0.1) | | 2 (0.1) | 1 (0.0) |
| 9.3.5.1.2 NEC | NA | 4 (0.0) | | NA | 1 (0.0) |
| 9.3.5.2 Rectum, adenocarcinoma | 657 (1.7) | 575 (1.0) | | 50 (2.4) | 63 (1.4) |
| 9.3.5.3 Rectum, other | 2 (0.0) | 4 (0.0) | | 1 (0.0) | NA |
| 9.3.6 Carcinoma of anus | 64 (0.2) | 67 (0.1) | | 18 (0.9) | 13 (0.3) |
| 9.3.6.1 Anus, squamous | 51 (0.1) | 58 (0.1) | | 17 (0.8) | 13 (0.3) |
| 9.3.6.2 Anus, other | 13 (0.0) | 9 (0.0) | | 1 (0.0) | NA |
| 9.3.7 Carcinoma of liver and intrahepatic bile ducts (IBD) | 77 (0.2) | 79 (0.1) | | 15 (0.7) | 7 (0.2) |
| 9.3.7.1 Liver and IBD, cholangiocarcinoma | 7 (0.0) | 15 (0.0) | | 2 (0.1) | 1 (0.0) |
| 9.3.7.2 Liver and IBD, hepatocellular carcinoma | 62 (0.2) | 60 (0.1) | | 9 (0.4) | 4 (0.1) |
| 9.3.7.3 Liver and IBD, other | 8 (0.0) | 4 (0.0) | | 4 (0.2) | 2 (0.0) |
| 9.3.8 Carcinoma of gallbladder and other extrahepatic biliary | 72 (0.2) | 61 (0.1) | | 10 (0.5) | 8 (0.2) |
| 9.3.9 Carcinoma of pancreas | 127 (0.3) | 162 (0.3) | | 45 (2.2) | 45 (1.0) |
| 9.3.9.1 Pancreas, neuroendocrine | 58 (0.1) | 81 (0.1) | | 6 (0.3) | 12 (0.3) |
| 9.3.9.1.1 NET | 38 (0.1) | 59 (0.1) | | 5 (0.2) | 12 (0.3) |
| 9.3.9.1.2 NEC | 6 (0.0) | 2 (0.0) | | 1 (0.0) | NA |
| 9.3.9.1.3 Neuroendocrine, other | 14 (0.0) | 20 (0.0) | | NA | NA |
| 9.3.9.2 Pancreas, adenocarcinoma | 66 (0.2) | 78 (0.1) | | 38 (1.8) | 31 (0.7) |
| 9.3.9.3 Pancreas, other | 3 (0.0) | 3 (0.0) | | 1 (0.0) | 2 (0.0) |
| 9.3.10 Other carcinoma of gastrointestinal tract | 8 (0.0) | 6 (0.0) | | 3 (0.1) | NA |
| 9.4 Carcinoma of lung, bronchus, and trachea | 701 (1.8) | 908 (1.5) | | 146 (7.1) | 345 (7.4) |
| 9.4.1 Small cell carcinoma, NEC | 79 (0.2) | 81 (0.1) | | 22 (1.1) | 39 (0.8) |
| 9.4.2 Non-small cell carcinoma | 622 (1.6) | 827 (1.4) | | 124 (6.0) | 306 (6.6) |
| 9.4.2.1 Non-small cell, adenocarcinoma | 256 (0.6) | 370 (0.6) | | 64 (3.1) | 199 (4.3) |
| 9.4.2.2 Non-small cell, neuroendocrine | 159 (0.4) | 253 (0.4) | | 3 (0.1) | 13 (0.3) |
| 9.4.2.2.1 Non-small cell NET | 142 (0.4) | 237 (0.4) | | 2 (0.1) | 4 (0.1) |
| 9.4.2.2.2 Non-small cell NEC | 17 (0.0) | 16 (0.0) | | 1 (0.0) | 9 (0.2) |
| 9.4.2.3 Non-small cell, other | 207 (0.5) | 204 (0.3) | | 57 (2.8) | 94 (2.0) |
| 9.5 Carcinoma of skin (if collected) | 474 (1.2) | 541 (0.9) | | 93 (4.5) | 121 (2.6) |
| 9.6 Carcinoma of breast | 36 (0.1) | 19,435 (32.7) | | 6 (0.3) | 2,071 (44.7) |
| 9.6.1 Breast, infiltrating duct | 28 (0.1) | 15,962 (26.9) | | 5 (0.2) | 1,708 (36.9) |
| 9.6.2 Breast, adenocarcinoma | 6 (0.0) | 1,500 (2.5) | | 1 (0.0) | 147 (3.2) |
| 9.6.3 Breast, lobular | NA | 878 (1.5) | | NA | 152 (3.3) |
| 9.6.4 Breast, phyllodes | NA | 65 (0.1) | | NA | 1 (0.0) |
| 9.6.5 Breast, medullary | NA | 445 (0.7) | | NA | 27 (0.6) |
| 9.6.6 Breast, Paget | 1 (0.0) | 96 (0.2) | | NA | 6 (0.1) |
| 9.6.7 Breast, ductal | NA | 36 (0.1) | | NA | 2 (0.0) |
| 9.6.8 Breast, metaplastic | NA | 71 (0.1) | | NA | 11 (0.2) |
| 9.6.9 Breast, inflammatory | NA | 3 (0.0) | | NA | NA |
| 9.6.10 Breast, other | 1 (0.0) | 379 (0.6) | | NA | 17 (0.4) |
| 9.7 Carcinoma of genital sites excluding ovary and testis | 86 (0.2) | 6,919 (11.7) | | 77 (3.7) | 231 (5.0) |
| 9.7.1 Carcinoma of uterine cervix | NA | 6,266 (10.6) | | NA | 86 (1.9) |
| 9.7.1.1 Cervix, squamous | NA | 4,517 (7.6) | | NA | 59 (1.3) |
| 9.7.1.2 Cervix, adenosquamous | NA | 219 (0.4) | | NA | 2 (0.0) |
| 9.7.1.3 Cervix, adenocarcinoma | NA | 1,223 (2.1) | | NA | 24 (0.5) |
| 9.7.1.4 Cervix, other | NA | 307 (0.5) | | NA | 1 (0.0) |
| 9.7.2 Corpus uteri | NA | 307 (0.5) | | NA | 96 (2.1) |
| 9.7.2.1 Corpus uteri, adenocarcinoma | NA | 243 (0.4) | | NA | 90 (1.9) |
| 9.7.2.1.1 Corpus uteri, endometrioid | NA | 130 (0.2) | | NA | 76 (1.6) |
| 9.7.2.1.2 Corpus uteri, other adenocarcinoma | NA | 113 (0.2) | | NA | 14 (0.3) |
| 9.7.2.2 Corpus uteri, other | NA | 64 (0.1) | | NA | 6 (0.1) |
| 9.7.3 Carcinoma of vulva and vagina | NA | 328 (0.6) | | NA | 40 (0.9) |
| 9.7.4 Carcinoma of penis | 66 (0.2) | NA | | 5 (0.2) | NA |
| 9.7.5 Carcinoma of prostate | 16 (0.0) | NA | | 71 (3.4) | NA |
| 9.7.6 Other genital | 4 (0.0) | 18 (0.0) | | 1 (0.0) | 9 (0.2) |
| 9.8 Carcinoma of urinary tract | 866 (2.2) | 548 (0.9) | | 119 (5.8) | 85 (1.8) |
| 9.8.1 Carcinoma of kidney | 579 (1.5) | 375 (0.6) | | 78 (3.8) | 45 (1.0) |
| 9.8.1.1 Kidney, adenocarcinoma | 575 (1.4) | 375 (0.6) | | 78 (3.8) | 44 (0.9) |
| 9.8.1.1.1 Kidney, renal cell | 524 (1.3) | 334 (0.6) | | 64 (3.1) | 42 (0.9) |
| 9.8.1.1.2 Kidney, other adenocarcinoma | 51 (0.1) | 41 (0.1) | | 14 (0.7) | 2 (0.0) |
| 9.8.1.2 Kidney, other | 4 (0.0) | NA | | NA | 1 (0.0) |
| 9.8.2 Carcinoma of bladder | 252 (0.6) | 155 (0.3) | | 32 (1.6) | 31 (0.7) |
| 9.8.2.1 Urinary bladder, transitional cell carcinoma | 218 (0.5) | 116 (0.2) | | 28 (1.4) | 25 (0.5) |
| 9.8.2.2 Urinary bladder, other carcinoma | 34 (0.1) | 39 (0.1) | | 4 (0.2) | 6 (0.1) |
| 9.8.3 Other urinary | 35 (0.1) | 18 (0.0) | | 9 (0.4) | 9 (0.2) |
| 9.9 Other invasive carcinomas | 278 (0.7) | 344 (0.6) | | 24 (1.2) | 58 (1.3) |
| 9.9.1 Adrenocortical carcinoma | 22 (0.1) | 46 (0.1) | | 2 (0.1) | 1 (0.0) |
| 9.9.2 Unknown primary | 177 (0.4) | 201 (0.3) | | 20 (1.0) | 45 (1.0) |
| 9.9.3 Thymic carcinoma | 53 (0.1) | 41 (0.1) | | 2 (0.1) | 1 (0.0) |
| 9.9.4 Carcinoma of other and ill-defined sites | 26 (0.1) | 56 (0.1) | | NA | 11 (0.2) |
| **10. Miscellaneous specified neoplasms** | 103 (0.3) | 137 (0.2) | | 7 (0.3) | 11 (0.2) |
| 10.1 Other paediatric and embryonal tumours | 35 (0.1) | 51 (0.1) | | 1 (0.0) | 1 (0.0) |
| 10.1.1 Wilms tumour | 8 (0.0) | 11 (0.0) | | NA | (0.0) |
| 10.1.2 Olfactory and non-CNS neuroblastoma | 21 (0.1) | 30 (0.1) | | NA | 1 (0.0) |
| 10.1.3 Other embryonal non-CNS tumours | 6 (0.0) | 10 (0.0) | | 1 (0.0) | NA |
| 10.2 Other specified tumours | 68 (0.2) | 86 (0.1) | | 6 (0.3) | 10 (0.2) |
| 10.2.1 Paraganglioma, non-CNS | 32 (0.1) | 22 (0.0) | | 1 (0.0) | 1 (0.0) |
| 10.2.2 Other specified neoplasms | 36 (0.1) | 64 (0.1) | | 5 (0.2) | 9 (0.2) |
| **11. Unspecified malignant neoplasms except CNS** | 83 (0.2) | 49 (0.1) | | 21 (1.0) | 27 (0.6) |

Abbreviation: TNM=Tumour, Node, Metastasis, Figo=Fédération Internationale de Gynécologie et d'Obstétrique, IQR=Interquartile range, NOS=Not otherwise specified, CNS=Central Nervous System, NA=Not Applicable, NA=Not Applicable.

^a^ Percentages might not add-up to 100% due to rounding.

^b^ Age at diagnosis of first and second primary cancer.

^c^ Years of follow-up from six month survival until the date of second cancer diagnosis, death, loss to follow-up, or December 31, 2018, whichever came first.

**Supplementary Table S2A.** Standardised incidence ratios (SIRs) and absolute excess risks (AERs) of any second primary malignant cancer diagnosis after first primary malignant cancer among six-month adolescent and young adult (AYA, aged 15-39 years) cancer survivors compared to the general population in the Netherlands by follow-up years. Cancer types were grouped according to the AYA-specific classification scheme developed by Barr and colleagues (2020). First and second cancer combinations with less than n=10 observed second cancers were excluded from the analyses. This Table presents outcomes from the main analysis.

|  | **Follow-up (years)** | **1-4** | | | | **5-9** | | | | **10-14** | | | | **15-19** | | | | **20-25** | | | |
| --- | --- | --- | --- | --- | --- | --- | --- | --- | --- | --- | --- | --- | --- | --- | --- | --- | --- | --- | --- | --- | --- |
|  |  | **Obs/Exp** | **SIR (95%CI)** | **AER (95%CI)** | **P-value** | **Obs/Exp** | **SIR  (95%CI)** | **AER  (95%CI)** | **P-value** | **Obs/Exp** | **SIR (95%CI)** | **AER (95%CI)** | **P-value** | **Obs/Exp** | **SIR  (95%CI)** | **AER  (95%CI)** | **P-value** | **Obs/Exp** | **SIR  (95%CI)** | **AER  (95%CI)** | **P-value** |
| **Males** | **Total** | **255/110** | **2.3  (2.0, 2.6)** | **12.2  (9.6, 15.0)** | **0.000** | **304/140** | **2.2  (1.9, 2.4)** | **15.1  (12.1, 18.5)** | **0.000** | **279/146** | **1.9  (1.7, 2.2)** | **18.1 (13.8, 22.8)** | **0.000** | **281/148** | **1.9  (1.7, 2.1)** | **29.2 (22.2, 36.9)** | **0.000** | **232/142** | **1.6  (1.4, 1.9)** | **35.2  (23.9, 47.6)** | **0.000** |
|  | **Age at diagnosis (years)^a^** |  |  |  |  |  |  |  |  |  |  |  |  |  |  |  |  |  |  |  |  |
|  | 15-19 | 9/3 | 2.9 (1.3, 5.5) | 6.1  (1.0, 14.6) | 0.010 | 12/5 | 2.5  (1.3, 4.4) | 8.1  (1.6, 18.1) | 0.008 | 14/5 | 3.0  (1.7, 5.1) | 15.3  (5.0, 30.8) | 0.001 | 13/4 | 3.6  (1.9, 6.1) | 24.1  (8.4, 47.8) | 0.000 | 3/3 | 1.0  (0.2, 2.8) | -0.3  (-10.6, 24.5) | 1.000 |
|  | 20-24 | 22/9 | 2.5  (1.5, 3.7) | 8.0  (3.0, 15.0) | 0.000 | 21/11 | 1.9  (1.2, 2.9) | 6.8  (1.4, 14.3) | 0.009 | 23/10 | 2.4  (1.5, 3.6) | 13.0  (4.8, 24.2) | 0.000 | 18/8 | 2.1  (1.3, 3.4) | 14.5  (3.4, 30.4) | 0.006 | 22/8 | 2.6  (1.6, 4.0) | 33.6  (13.4, 61.4) | 0.000 |
|  | 25-29 | 41/18 | 2.3  (1.6, 3.1) | 9.5  (4.7, 15.5) | 0.000 | 43/20 | 2.1  (1.5, 2.8) | 10.3  (4.9, 17.0) | 0.000 | 47/19 | 2.4  (1.8, 3.2) | 18  (9.9, 28.2) | 0.000 | 33/20 | 1.6  (1.1, 2.3) | 12.8  (2.6, 26) | 0.010 | 37/20 | 1.9  (1.3, 2.6) | 29.3  (10.7, 53.0) | 0.001 |
|  | 30-34 | 66/29 | 2.3  (1.7, 2.9) | 11.8  (7.0, 17.6) | 0.000 | 70/36 | 1.9  (1.5, 2.5) | 12.0  (6.5, 18.5) | 0.000 | 72/39 | 1.9  (1.5, 2.3) | 17.0  (9.0, 26.6) | 0.000 | 78/40 | 2.0  (1.6, 2.5) | 31.7  (18.2, 47.7) | 0.000 | 73/41 | 1.8  (1.4, 2.3) | 48.6  (24.8, 76.8) | 0.000 |
|  | 35-39 | 117/50 | 2.3  (1.9, 2.8) | 17.4  (12.1, 23.5) | 0.000 | 158/68 | 2.3  (2.0, 2.7) | 26.2  (19.3, 33.9) | 0.000 | 123/73 | 1.7  (1.4, 2.0) | 22.1  (12.8, 32.7) | 0.000 | 139/76 | 1.8  (1.5, 2.2) | 49  (31.7, 68.6) | 0.000 | 97/70 | 1.4  (1.1, 1.7) | 40.4  (12.8, 72.5) | 0.003 |
|  | **Tumour stage (TNM, Figo and Ann arbor)** |  |  |  |  |  |  |  |  |  |  |  |  |  |  |  |  |  |  |  |  |
|  | stage I | 82/54 | 1.5  (1.2, 1.9) | 4.8  (1.9, 8.3) | 0.001 | 118/71 | 1.7  (1.4, 2.0) | 8.8  (5.0, 13.1) | 0.000 | 115/74 | 1.6  (1.3, 1.9) | 11.3  (5.8, 17.6) | 0.000 | 112/75 | 1.5  (1.2, 1.8) | 16.4  (7.5, 26.5) | 0.000 | 99/73 | 1.4  (1.1, 1.7) | 20.7  (6.0, 37.7) | 0.004 |
|  | stage II | 49/18 | 2.7  (2.0, 3.5) | 15.0  (8.7, 22.7) | 0.000 | 54/24 | 2.2  (1.7, 2.9) | 15.5  (8.4, 24.1) | 0.000 | 49/27 | 1.8  (1.4, 2.4) | 16.3  (6.9, 27.9) | 0.000 | 56/28 | 2.0  (1.5, 2.6) | 32.6  (16.7, 52.0) | 0.000 | 44/25 | 1.7  (1.3, 2.3) | 41.2  (14.6, 74.5) | 0.001 |
|  | stage III | 39/12 | 3.4  (2.4, 4.6) | 21.3  (12.6, 32.5) | 0.000 | 33/14 | 2.3  (1.6, 3.3) | 16.7  (7.6, 28.6) | 0.000 | 35/14 | 2.4  (1.7, 3.4) | 27.4  (13.4, 45.5) | 0.000 | 44/13 | 3.4  (2.4, 4.5) | 72.9  (44.5, 108.4) | 0.000 | 21/11 | 2.0  (1.2, 3.0) | 52.0  (12.2, 107.3) | 0.006 |
|  | stage IV | 27/6 | 4.7  (3.1, 6.9) | 33.8  (19.2, 53.2) | 0.000 | 27/7 | 4.1  (2.7, 5.9) | 39.2  (21.5, 62.8) | 0.000 | 22/7 | 3.3  (2.0, 4.9) | 43.0  (19.8, 74.9) | 0.000 | 20/7 | 2.7  (1.7, 4.2) | 53.4  (20.7, 99.3) | 0.000 | 19/10 | 1.9  (1.1, 3.0) | 47.9  (7.8, 104.5) | 0.014 |
|  | Other/unknown | 58/20 | 3.0  (2.2, 3.8) | 17.2  (10.9, 24.8) | 0.000 | 72/24 | 3.0  (2.3, 3.8) | 25.1  (16.9, 34.8) | 0.000 | 58/24 | 2.4  (1.8, 3.1) | 26.9  (15.8, 40.4) | 0.000 | 49/24 | 2.0  (1.5, 2.7) | 31.3  (15.1, 51.3) | 0.000 | 49/23 | 2.1  (1.6, 2.8) | 56.9  (28.7, 91.8) | 0.000 |
|  | **Cancer type** |  |  |  |  |  |  |  |  |  |  |  |  |  |  |  |  |  |  |  |  |
|  | **1. Leukaemia’s and related disorder** | **10/5** | **2.0  (0.9, 3.6)** | **8.4  (-0.5, 22.8)** | **0.070** | **19/6** | **3.1  (1.9, 4.8)** | **26.1  (10.7, 47.8)** | **0.000** | **16/6** | **2.7  (1.5, 4.4)** | **33.1  (10.6, 65.9)** | **0.001** | **8/5** | **1.5  (0.6, 3.0)** | **16.1  (-11.2, 62.7)** | **0.339** | **11/5** | **2.4  (1.2, 4.3)** | **75.0  (11.0, 175.9)** | **0.014** |
|  | **2. Lymphomas** | **42/17** | **2.5  (1.8, 3.3)** | **12.6  (6.7, 20.0)** | **0.000** | **65/23** | **2.9  (2.2, 3.7)** | **22.7  (14.7, 32.2)** | **0.000** | **82/24** | **3.4  (2.7, 4.2)** | **44.3  (31.4, 59.3)** | **0.000** | **75/24** | **3.2  (2.5, 4.0)** | **63.5  (43.7, 87)** | **0.000** | **61/22** | **2.7  (2.1, 3.5)** | **84.5  (53.1, 122.5)** | **0.000** |
|  | **3. CNS and other intracranial and intraspinal neoplasms** | **11/5** | **2.1  (1.0, 3.7)** | **8.9  (0.3, 22.5)** | **0.041** | **10/5** | **1.9  (0.9, 3.5)** | **10.6  (-1.0, 29.2)** | **0.083** | **8/4** | **1.9  (0.8, 3.7)** | **15.1  (-3.4, 46.6)** | **0.139** | **7/4** | **1.9  (0.7, 3.8)** | **22.2  (-6.5, 73.0)** | **0.174** | **6/3** | **1.9  (0.7, 4.1)** | **36.3  (-12.8, 127.7)** | **0.209** |
|  | **4. Sarcomas** | **16/6** | **2.8  (1.6, 4.5)** | **14.6  (4.8, 28.8)** | **0.001** | **13/8** | **1.7  (0.9, 2.9)** | **8.3  (-1.1, 22.6)** | **0.096** | **15/8** | **1.8  (1.0, 3.0)** | **14.9  (0.4, 36.3)** | **0.042** | **12/9** | **1.4  (0.7, 2.4)** | **10.8  (-8.3, 40.3)** | **0.344** | **15/10** | **1.6  (0.9, 2.6)** | **28.4  (-6.1, 79.4)** | **0.126** |
|  | **5. Blood and lymphatic vessel tumours** | **15/1** | **10.5  (5.9, 17.3)** | **95.1  (48.8, 163.4)** | **0.000** | **9/2** | **4.9  (2.3, 9.4)** | **57.9  (18.5, 123.2)** | **0.000** | **7/2** | **3.6  (1.5, 7.5)** | **61.8  (10.7, 152.3)** | **0.008** | **4/2** | **2.0  (0.5, 5.2)** | **40.2  (-17.9, 165.1)** | **0.281** | **2/1** | **1.4  (0.2, 5.1)** | **27.3  (-54.6, 270.7)** | **0.827** |
|  | **6. Nerve sheath tumours** | **0/0** | **0.0  (0.0, 14.9)** | **-8.6  (-8.6, 118.9)** | **0.781** | **2/0** | **5.9  (0.7, 21.1)** | **60.6  (-3.6, 251.7)** | **0.093** | **0/0** | **0.0  (0.0, 9.5)** | **-20.0  (-20.0, 169.6)** | **0.678** | **1/0** | **2.2  (0.1, 12.0)** | **44.3  (-36.1, 421.4)** | **0.741** | **0/0** | **0.0  (0.0, 7.8)** | **-57.7  (-57.7, 391.7)** | **0.623** |
|  | **7. Gonadal and related tumours** | **45/35** | **1.3  (0.9, 1.7)** | **2.4  (-0.6, 6.2)** | **0.127** | **66/45** | **1.5  (1.1, 1.8)** | **5.4  (1.5, 10.2)** | **0.005** | **67/47** | **1.4  (1.1, 1.8)** | **7.7  (1.9, 14.6)** | **0.007** | **73/47** | **1.5  (1.2, 1.9)** | **15.9  (6.1, 27.6)** | **0.001** | **61/45** | **1.4  (1.0, 1.7)** | **18.0  (1.8, 37.6)** | **0.027** |
|  | **8. Melanoma, malignant** | **29/19** | **1.5  (1.0, 2.2)** | **5.1  (0.1, 11.7)** | **0.046** | **36/25** | **1.4  (1.0, 2.0)** | **6.2  (0.1, 14.1)** | **0.047** | **24/26** | **0.9  (0.6, 1.3)** | **-2.1  (-9.2, 7.7)** | **0.723** | **31/27** | **1.2  (0.8, 1.6)** | **5.7  (-7.8, 23.4)** | **0.464** | **22/25** | **0.9  (0.5, 1.3)** | **-7.8  (-27.9, 19.8)** | **0.609** |
|  | **9. Carcinomas** | **86/20** | **4.3  (3.5, 5.3)** | **35.1  (26.0, 45.9)** | **0.000** | **84/25** | **3.3  (2.6, 4.1)** | **35.6  (25.3, 47.7)** | **0.000** | **59/27** | **2.2  (1.6, 2.8)** | **28.2  (15.7, 43.5)** | **0.000** | **70/29** | **2.4  (1.9, 3.0)** | **57.2  (35.5, 83.3)** | **0.000** | **54/30** | **1.8  (1.4, 2.4)** | **58.1  (25.7, 97.8)** | **0.000** |
|  | 9.1 Thyroid carcinoma | 6/3 | 2.3  (0.8, 4.9) | 12.0  (-1.6, 37.3) | 0.104 | 7/4 | 2.0  (0.8, 4.1) | 13.3  (-2.7, 41.6) | 0.134 | 4/4 | 1.0  (0.3, 2.7) | 1.0  (-14.9, 35.1) | 1.000 | 10/4 | 2.4  (1.1, 4.4) | 47.8  (5.1, 116.7) | 0.021 | 6/4 | 1.4  (0.5, 3.0) | 21.5  (-29.5, 116.2) | 0.560 |
|  | 9.2 Other carcinoma of head and neck | 17/3 | 5.2  (3.0, 8.3) | 43.7  (21.1, 76.3) | 0.000 | 18/5 | 4.0  (2.4, 6.3) | 45.7  (20.8, 81.1) | 0.000 | 19/5 | 3.8  (2.3, 5.9) | 68.6  (31.5, 120.9) | 0.000 | 17/5 | 3.3  (1.9, 5.3) | 99.8  (40.1, 185.7) | 0.000 | 9/5 | 1.8  (0.8, 3.3) | 57.8  (-15.1, 178.5) | 0.153 |
|  | 9.3 Carcinoma of gastrointestinal tract | 35/7 | 4.8  (3.3, 6.6) | 40.0  (24.7, 59.8) | 0.000 | 28/9 | 3.2  (2.1, 4.6) | 33.8  (17.2, 55.7) | 0.000 | 17/9 | 1.8  (1.0, 2.9) | 19.3  (1.1, 45.5) | 0.035 | 21/11 | 2.0  (1.2, 3.1) | 41  (9.7, 84.3) | 0.006 | 21/11 | 1.9  (1.2, 2.9) | 62.7  (11.9, 133.3) | 0.011 |
|  | 9.4 Carcinoma of lung, bronchus, and trachea | 5/1 | 4.6  (1.5, 10.7) | 37.9  (5.2, 102.5) | 0.010 | 4/1 | 3.2  (0.9, 8.1) | 33.5  (-2.1, 109.8) | 0.079 | 4/1 | 3.0  (0.8, 7.8) | 50.5  (-4.3, 168.2) | 0.090 | 4/1 | 3.1  (0.8, 7.8) | 84.3  (-6.9, 279.9) | 0.088 | 3/1 | 2.5  (0.5, 7.3) | 108.5  (-34.7, 455.5) | 0.239 |
|  | 9.5 Carcinoma of skin (if collected) | 13/2 | 7.5  (4.0, 12.8) | 71.9  (33.1, 130.9) | 0.000 | 11/2 | 4.8  (2.4, 8.5) | 61.1  (22.4, 122.1) | 0.000 | 6/2 | 2.4  (0.9, 5.3) | 36.8  (-2.8, 110.4) | 0.081 | 6/3 | 2.4  (0.9, 5.1) | 59.4  (-5.7, 180.5) | 0.089 | 5/2 | 2.1  (0.7, 4.8) | 80.3  (-25.1, 288.3) | 0.199 |
|  | 9.6 Carcinoma of breast | 0/0 | 0.0  (0.0, 29) | -10.7  (-10.7, 299) | 0.881 | 0/0 | 0.0  (0.0, 25.5) | -15.7  (-15.7, 384.2) | 0.865 | 0/0 | 0.0  (0.0, 22) | -25.8  (-25.8, 541) | 0.846 | 1/0 | 8.3  (0.2, 46.1) | 277.7  (-30.2, 1722.0) | 0.228 | 0/0 | 0.0  (0.0, 38.1) | -60.1  (-60.1, 2231.4) | 0.908 |
|  | 9.7 Carcinoma of genital sites excluding ovary and testis | 2/0 | 6.0  (0.7, 21.7) | 60.0  (-3.3, 247.9) | 0.089 | 1/0 | 2.3  (0.1, 12.8) | 22.9  (-16.6, 208.4) | 0.705 | 0/0 | 0.0  (0.0, 8.7) | -28.6  (-28.6, 221.1) | 0.655 | 1/1 | 1.9  (0.0, 10.5) | 45.5  (-49.0, 488.6) | 0.824 | 0/0 | 0.0  (0.0, 8.9) | -87.2  (-87.2, 688) | 0.660 |
|  | 9.8 Carcinoma of urinary tract | 7/3 | 2.4  (1.0, 5.0) | 16.3  (-0.4, 45.7) | 0.058 | 10/4 | 2.6  (1.2, 4.8) | 27.1  (4.2, 64.1) | 0.013 | 9/4 | 2.2  (1.0, 4.2) | 32.5  (0.3, 85.9) | 0.047 | 9/5 | 2.0  (0.9, 3.8) | 47.5  (-4.2, 133.1) | 0.082 | 7/5 | 1.5  (0.6, 3.1) | 41.3  (-33.9, 174.5) | 0.391 |
|  | 9.9 Other invasive carcinomas | 1/0 | 2.1  (0.1, 11.9) | 11.8  (-9.8, 112.8) | 0.747 | 5/1 | 9.4  (3.1, 22.0) | 124.1  (30.3, 309.3) | 0.000 | 0/1 | 0.0  (0.0, 6.6) | -23.7  (-23.7, 133) | 0.573 | 1/1 | 1.6  (0.0, 9.0) | 28.2  (-43.5, 364.5) | 0.921 | 3/0 | 7.1  (1.5, 20.6) | 502.0  (37.8, 1626.1) | 0.019 |
|  | **10. Miscellaneous specified neoplasms** | **0/0** | **0.0  (0.0, 20.4)** | **-8.5  (-8.5, 164.7)** | **0.835** | **0/0** | **0.0  (0.0, 16.7)** | **-12.3  (-12.3, 192.8)** | **0.801** | **0/0** | **0.0  (0.0, 14.7)** | **-19.6  (-19.6, 269.1)** | **0.778** | **0/0** | **0.0  (0.0, 12.5)** | **-34.4  (-34.4, 397.4)** | **0.745** | **0/0** | **0.0  (0.0, 16.3)** | **-58.5  (-58.5, 893.1)** | **0.797** |
|  | **11. Unspecified malignant neoplasms except CNS** | **1/0** | **6.4  (0.2, 35.8)** | **45.3  (-7.0, 290.6)** | **0.288** | **0/0** | **0.0  (0.0, 21.7)** | **-11.1  (-11.1, 230.1)** | **0.843** | **1/0** | **6.9  (0.2, 38.3)** | **89.0  (-12.5, 565.0)** | **0.271** | **0/0** | **0.0  (0.0, 35.3)** | **-22.4  (-22.4, 769.5)** | **0.901** | **0/0** | **0.0  (0.0, 52.5)** | **-53.5  (-53.5, 2758.4)** | **0.932** |
| **Females** | **Total** | **490/343** | **1.4  (1.3, 1.6)** | **8.0  (5.7, 10.4)** | **0.000** | **608/479** | **1.3  (1.2, 1.4)** | **7.7  (4.8, 10.7)** | **0.000** | **594/493** | **1.2  (1.1, 1.3)** | **8.8  (4.7, 13.1)** | **0.000** | **541/433** | **1.3  (1.1, 1.4)** | **15.0  (8.8, 21.5)** | **0.000** | **404/331** | **1.2  (1.1, 1.3)** | **17.9  (8.5, 28.0)** | **0.000** |
|  | **Age at diagnosis (years)^a^** |  |  |  |  |  |  |  |  |  |  |  |  |  |  |  |  |  |  |  |  |
|  | 15-19 | 9/2 | 4.1  (1.9, 7.9) | 8.5  (2.4, 18.6) | 0.001 | 16/4 | 4.3  (2.5, 7.0) | 16.4  (7.3, 29.8) | 0.000 | 18/5 | 3.7  (2.2, 5.8) | 25.4  (11.2, 45.6) | 0.000 | 14/6 | 2.5  (1.4, 4.2) | 24.3  (6.0, 51.7) | 0.004 | 16/6 | 2.9  (1.7, 4.7) | 49.6  (17.2, 96.9) | 0.000 |
|  | 20-24 | 11/8 | 1.5  (0.7, 2.6) | 2.5  (-1.4, 8.6) | 0.278 | 23/13 | 1.8  (1.2, 2.8) | 8.0  (1.6, 16.8) | 0.010 | 29/15 | 1.9  (1.3, 2.8) | 15.1  (4.7, 28.8) | 0.002 | 31/16 | 1.9  (1.3, 2.8) | 24.3  (8.3, 45.3) | 0.001 | 44/16 | 2.8 ( 2.0, 3.7) | 73.3  (41.9, 112.7) | 0.000 |
|  | 25-29 | 47/27 | 1.7  (1.3, 2.3) | 7.4  (2.8, 13.2) | 0.001 | 64/40 | 1.6  (1.2, 2.1) | 9.9  (3.9, 17.2) | 0.000 | 76/45 | 1.7  (1.3, 2.1) | 18.1  (8.8, 29.1) | 0.000 | 84/48 | 1.8  (1.4, 2.2) | 31.0  (16.5, 48.0) | 0.000 | 54/41 | 1.3  (1.0, 1.7) | 19.3  (-0.4, 43.4) | 0.055 |
|  | 30-34 | 146/86 | 1.7  (1.4, 2.0) | 11.8  (7.3, 16.8) | 0.000 | 137/119 | 1.1  (1.0, 1.4) | 3.7  (-0.9, 9.1) | 0.123 | 151/134 | 1.1  (1.0, 1.3) | 5.1  (-1.9, 12.9) | 0.163 | 156/124 | 1.3  (1.1, 1.5) | 15.3  (4.1, 27.9) | 0.006 | 96/94 | 1.0  (0.8, 1.3) | 2.1  (-13.6, 20.3) | 0.831 |
|  | 35-39 | 277/221 | 1.3  (1.1, 1.4) | 6.7  (2.9, 10.8) | 0.000 | 368/304 | 1.2  (1.1, 1.3) | 8.5 (3.6, 13.7) | 0.000 | 320/294 | 1.1  (1.0, 1.2) | 5.2  (-1.6, 12.6) | 0.141 | 256/239 | 1.1  (0.9, 1.2) | 5.5  (-4.6, 16.5) | 0.299 | 194/175 | 1.1  (1.0, 1.3) | 11.5  (-4.5, 29.2) | 0.166 |
|  | **Tumour stage (TNM, Figo and Ann arbor)** |  |  |  |  |  |  |  |  |  |  |  |  |  |  |  |  |  |  |  |  |
|  | stage I | 236/172 | 1.4  (1.2, 1.6) | 7.0  (3.8, 10.5) | 0.000 | 311/251 | 1.2  (1.1, 1.4) | 6.9  (3.0, 11.1) | 0.000 | 289/264 | 1.1  (1.0, 1.2) | 4.2  (-1.1, 10.0) | 0.128 | 240/238 | 1.0  (0.9, 1.1) | 0.5  (-7.0, 8.8) | 0.916 | 188/189 | 1.0  (0.9, 1.1) | -0.3  (-11.6, 12.3) | 1.000 |
|  | stage II | 136/98 | 1.4  (1.2, 1.6) | 7.6  (3.2, 12.6) | 0.000 | 157/136 | 1.2  (1.0, 1.4) | 4.7  (-0.5, 10.6) | 0.081 | 156/139 | 1.1  (1.0, 1.3) | 5.4  (-2.2, 14.0) | 0.169 | 163/117 | 1.4  (1.2, 1.6) | 24.3  (11.6, 38.6) | 0.000 | 112/85 | 1.3  (1.1, 1.6) | 26.2  (6.7, 48.5) | 0.007 |
|  | stage III | 41/30 | 1.4  (1.0, 1.9) | 7.1  (-0.4, 16.6) | 0.064 | 45/35 | 1.3  (0.9, 1.7) | 8.7  (-1.6, 21.5) | 0.105 | 58/29 | 2.0  (1.5, 2.6) | 42.1  (21.7, 67.0) | 0.000 | 47/21 | 2.3  (1.7, 3.0) | 75.4  (39.9, 119.6) | 0.000 | 27/14 | 1.9  (1.3, 2.8) | 72.1  (20.4, 141.0) | 0.003 |
|  | stage IV | 20/9 | 2.1  (1.3, 3.3) | 19.5  (5.2, 39.6) | 0.004 | 19/9 | 2.1  (1.2, 3.2) | 27.0  (6.0, 56.5) | 0.007 | 18/8 | 2.2  (1.3, 3.4) | 44.9  (10.9, 93.2) | 0.005 | 13/7 | 2.0  (1.1, 3.4) | 53.5  (3.2, 129.9) | 0.033 | 16/5 | 3.3  (1.9, 5.3) | 168.6  (64.8, 319.8) | 0.000 |
|  | Other/unknown | 57/33 | 1.7  (1.3, 2.2) | 11.0  (4.5, 18.8) | 0.000 | 76/48 | 1.6  (1.2, 2.0) | 13.7  (5.7, 23.2) | 0.000 | 73/53 | 1.4  (1.1, 1.7) | 14.2  (3.2, 27.2) | 0.009 | 78/50 | 1.5  (1.2, 1.9) | 28.1  (11.4, 47.8) | 0.000 | 61/38 | 1.6  (1.2, 2.1) | 42.8  (16.3, 74.9) | 0.001 |
|  | **Cancer type** |  |  |  |  |  |  |  |  |  |  |  |  |  |  |  |  |  |  |  |  |
|  | **1. Leukaemia’s and related disorders** | **17/8** | **2.2  (1.3, 3.6)** | **18.3  (4.4, 38.3)** | **0.005** | **13/10** | **1.3  (0.7, 2.2)** | **6.5  (-7.3, 27.6)** | **0.442** | **9/9** | **1.0  (0.5, 1.9)** | **-0.2  (-18.8, 30.5)** | **1.000** | **8/7** | **1.2  (0.5, 2.4)** | **10.0  (-23.0, 66.4)** | **0.691** | **10/5** | **2.2  (1.1, 4.0)** | **82.7  (3.8, 209.8)** | **0.036** |
|  | **2. Lymphomas** | **25/19** | **1.3  (0.9, 2.0)** | **4.2  (-1.8, 12.2)** | **0.201** | **59/28** | **2.1  (1.6, 2.7)** | **22.1  (12.1, 34.3)** | **0.000** | **76/30** | **2.5  (2.0, 3.1)** | **46.5  (30.2, 66.0)** | **0.000** | **88/28** | **3.2  (2.6, 3.9)** | **97.2  (69.2, 130.1)** | **0.000** | **64/20** | **3.1  (2.4, 4.0)** | **131.4  (87.0, 184.9)** | **0.000** |
|  | **3. CNS and other intracranial and intraspinal neoplasms** | **7/6** | **1.1  (0.4, 2.2)** | **1.3  (-7.8, 17.4)** | **0.923** | **6/7** | **0.8  (0.3, 1.8)** | **-3.5  (-14.6, 17.2)** | **0.844** | **7/6** | **1.1  (0.5, 2.3)** | **4.0  (-17.1, 41.4)** | **0.855** | **13/5** | **2.7  (1.4, 4.6)** | **71.8  (18.6, 152.6)** | **0.003** | **3/4** | **0.8  (0.2, 2.3)** | **-11.1  (-47.2, 76.3)** | **0.975** |
|  | **4. Sarcomas** | **16/10** | **1.6  (0.9, 2.6)** | **8.9  (-1.2, 23.4)** | **0.094** | **32/14** | **2.2  (1.5, 3.1)** | **27.4  (11.6, 47.9)** | **0.000** | **21/15** | **1.4  (0.8, 2.1)** | **12.7  (-5.3, 37.7)** | **0.196** | **21/14** | **1.5  (0.9, 2.3)** | **24.6  (-3.2, 63.3)** | **0.092** | **13/11** | **1.2  (0.6, 2.0)** | **10.8  (-25.0, 65.2)** | **0.658** |
|  | **5. Blood and lymphatic vessel tumours** | **2/0** | **4.0  (0.5, 14.6)** | **47.1  (-7.9, 210.5)** | **0.178** | **0/1** | **0.0  (0.0, 5.3)** | **-24.9  (-24.9, 106.8)** | **0.498** | **1/1** | **1.3  (0.0, 7.4)** | **12.7  (-37.9, 250.3)** | **1.000** | **0/1** | **0.0  (0.0, 7.0)** | **-53.6  (-53.6, 322.5)** | **0.591** | **0/1** | **0.0  (0.0, 7.0)** | **-75.5  (-75.5, 450.9)** | **0.589** |
|  | **6. Nerve sheath tumours** | **2/0** | **7.1  (0.9, 25.5)** | **79.2  (-1.9, 320.0)** | **0.066** | **0/0** | **0.0  (0.0, 8.7)** | **-21.5  (-21.5, 165.8)** | **0.655** | **0/1** | **0.0  (0.0, 6.7)** | **-35.6  (-35.6, 204.6)** | **0.579** | **1/0** | **2.1  (0.1, 11.8)** | **61.9  (-52.8, 599.5)** | **0.755** | **0/0** | **0.0  (0.0, 11.7)** | **-71.0  (-71.0, 759.4)** | **0.730** |
|  | **7. Gonadal and related tumours** | **10/12** | **0.9  (0.4, 1.6)** | **-2.2  (-9.6, 9.7)** | **0.792** | **23/18** | **1.3  (0.8, 1.9)** | **7.1  (-4.8, 23.4)** | **0.285** | **38/22** | **1.7  (1.2, 2.3)** | **27.0  (7.7, 51.6)** | **0.003** | **29/24** | **1.2 (0.8, 1.7)** | **10.5  (-11.4, 39.3)** | **0.401** | **24/19** | **1.3  (0.8, 1.9)** | **21.9  (-13.1, 69.5)** | **0.259** |
|  | **8. Melanoma, malignant** | **89/65** | **1.4  (1.1, 1.7)** | **6.2  (1.6, 11.7)** | **0.007** | **112/96** | **1.2  (1.0, 1.4)** | **4.3  (-1.1, 10.6)** | **0.125** | **114/99** | **1.1  (0.9, 1.4)** | **5.9  (-2.0, 15.0)** | **0.154** | **92/85** | **1.1  (0.9, 1.3)** | **4.3  (-7.1, 17.8)** | **0.493** | **50/65** | **0.8  (0.6, 1.0)** | **-17.6  (-32.3, 0.7)** | **0.059** |
|  | **9. Carcinomas** | **320/222** | **1.4  (1.3, 1.6)** | **9.2  (6.0, 12.7)** | **0.000** | **360/303** | **1.2  (1.1, 1.3)** | **6.0  (2.2, 10.1)** | **0.002** | **326/308** | **1.1  (0.9, 1.2)** | **2.9  (-2.5, 8.7)** | **0.308** | **286/267** | **1.1  (0.9, 1.2)** | **4.6  (-3.4, 13.2)** | **0.271** | **238/205** | **1.2  (1.0, 1.3)** | **14.3  (1.7, 28.3)** | **0.026** |
|  | 9.1 Thyroid carcinoma | 13/15 | 0.9  (0.5, 1.5) | -1.9  (-8.6, 8.3) | 0.785 | 28/21 | 1.3  (0.9, 1.9) | 7.9  (-3.0, 22.5) | 0.180 | 25/23 | 1.1  (0.7, 1.6) | 3.7  (-10.9, 23.5) | 0.690 | 26/20 | 1.3  (0.8, 1.9) | 15.2  (-8.5, 47) | 0.245 | 25/16 | 1.6  (1.0, 2.3) | 41.3  (0.9, 95.9) | 0.044 |
|  | 9.2 Other carcinoma of head and neck | 22/4 | 5.1  (3.2, 7.7) | 72.9  (39.1, 119.5) | 0.000 | 12/6 | 1.9  (1.0, 3.4) | 25.2  (0.0, 64.1) | 0.050 | 17/6 | 2.7  (1.6, 4.3) | 68.0  (22.6, 133.2) | 0.001 | 9/6 | 1.6  (0.7, 3.1) | 34.9  (-14.8, 117) | 0.223 | 8/4 | 1.8  (0.8, 3.6) | 65.0  (-15.5, 202.5) | 0.146 |
|  | 9.3 Carcinoma of gastrointestinal tract | 31/13 | 2.3  (1.6, 3.3) | 23.6  (10.3, 41.1) | 0.000 | 22/18 | 1.2  (0.8, 1.9) | 6.9  (-6.2, 25.0) | 0.357 | 41/18 | 2.3  (1.6, 3.1) | 53.1  (26.4, 86.8) | 0.000 | 18/17 | 1.1  (0.6, 1.7) | 4.4  (-20.2, 39.5) | 0.812 | 21/13 | 1.6  (1.0, 2.4) | 44.4  (-0.8, 107) | 0.055 |
|  | 9.4 Carcinoma of lung, bronchus, and trachea | 6/3 | 2.0  (0.7, 4.4) | 20.4  (-5.0, 67.5) | 0.157 | 7/4 | 1.8  (0.7, 3.7) | 24.3  (-8.2, 81.7) | 0.195 | 1/4 | 0.2  (0.0, 1.3) | -34.9  (-45.5, 15.2) | 0.158 | 6/4 | 1.5  (0.6, 3.3) | 33.8  (-27.5, 147.7) | 0.400 | 4/3 | 1.3  (0.3, 3.3) | 23.8  (-54.3, 191.4) | 0.757 |
|  | 9.5 Carcinoma of skin (if collected) | 12/4 | 3.2  (1.7, 5.6) | 46.7  (13.9, 97.5) | 0.001 | 15/5 | 2.9  (1.6, 4.7) | 59.5  (19.3, 118.7) | 0.001 | 4/5 | 0.7  (0.2, 1.9) | -12.0  (-37.7, 43.1) | 0.760 | 8/5 | 1.7  (0.7, 3.3) | 43.3  (-18.3, 148.6) | 0.228 | 7/3 | 2.2  (0.9, 4.6) | 101.3  (-9.4, 297.5) | 0.086 |
|  | 9.6 Carcinoma of breast | 155/135 | 1.1  (1.0, 1.3) | 3.3  (-0.6, 7.7) | 0.099 | 174/179 | 1.0  (0.8, 1.1) | -0.9  (-5.7, 4.5) | 0.758 | 137/172 | 0.8  (0.7, 0.9) | -10.5  (-17.1, -3.0) | 0.007 | 114/139 | 0.8  (0.7, 1.0) | -12.8  (-23.0, -1.0) | 0.033 | 93/101 | 0.9  (0.7, 1.1) | -7.6  (-24.5, 12.2) | 0.459 |
|  | 9.7 Carcinoma of genital sites excluding ovary and testis | 71/43 | 1.6  (1.3, 2.1) | 12.8  (5.6, 21.3) | 0.000 | 90/64 | 1.4  (1.1, 1.7) | 12.2  (3.8, 22.0) | 0.003 | 93/73 | 1.3  (1.0, 1.6) | 12.7  (1.3, 26.0) | 0.028 | 96/72 | 1.3  (1.1, 1.6) | 21.6  (5.1, 40.8) | 0.008 | 76/60 | 1.3  (1.0, 1.6) | 23.3  (-0.4, 51.5) | 0.054 |
|  | 9.8 Carcinoma of urinary tract | 3/3 | 1.0  (0.2, 2.8) | -0.7  (-15.9, 36.1) | 1.000 | 8/4 | 1.8  (0.8, 3.6) | 25.4  (-6.4, 79.7) | 0.153 | 7/4 | 1.6  (0.6, 3.2) | 26.1  (-16.8, 102.2) | 0.326 | 7/4 | 1.8  (0.7, 3.6) | 47.1  (-18.5, 163.2) | 0.220 | 4/3 | 1.3  (0.3, 3.2) | 22.3  (-54.8, 187.5) | 0.777 |
|  | 9.9 Other invasive carcinomas | 7/1 | 5.6  (2.3, 11.6) | 93.1  (25.4, 213.1) | 0.001 | 4/2 | 2.6  (0.7, 6.6) | 50.6  (-9.6, 179.8) | 0.145 | 1/2 | 0.7  (0.0, 3.7) | -16.0  (-46.7, 127.6) | 1.000 | 2/1 | 1.6  (0.2, 5.9) | 41.3  (-52.5, 320.1) | 0.693 | 0/1 | 0.0  (0.0, 4.3) | -86.2  (-86.2, 284) | 0.423 |
|  | **10. Miscellaneous specified neoplasms** | **2/1** | **3.1  (0.4, 11.3)** | **34.0  (-10.0, 164.8)** | **0.271** | **1/1** | **1.1  (0.0, 6.2)** | **2.7  (-25.2, 133.9)** | **1.000** | **0/1** | **0.0  (0.0, 3.2)** | **-40.6  (-40.6, 89.6)** | **0.317** | **3/1** | **2.5  (0.5, 7.2)** | **83.9  (-27.9, 354.6)** | **0.246** | **2/1** | **2.0  (0.2, 7.3)** | **79.4  (-59.7, 493)** | **0.526** |
|  | **11. Unspecified malignant neoplasms except CNS** | **0/0** | **0.0  (0.0, 16.8)** | **-19.1  (-19.1, 301.8)** | **0.803** | **2/0** | **6.6  (0.8, 23.7)** | **156.5  (-5.8, 638.9)** | **0.076** | **2/0** | **6.3  (0.8, 22.7)** | **205.4  (-9.2, 843.4)** | **0.082** | **0/0** | **0.0  (0.0, 13.1)** | **-55.6  (-55.6, 673.5)** | **0.755** | **0/0** | **0.0  (0.0, 11.5)** | **-81.0  (-81.0, 847.2)** | **0.725** |

Abbreviation: TNM=Tumour, Node, Metastasis, Figo=Fédération Internationale de Gynécologie et d'Obstétrique, CNS=Central Nervous System.

^a^ Age at diagnosis of first and second primary cancer.

**Supplementary Table S2B.** Standardised incidence ratios (SIRs) and absolute excess risks (AERs) of any second primary malignant cancer diagnosis after first primary malignant cancer among six-month adolescent and young adult (AYA, aged 15-39 years) cancer survivors compared to the general population in the Netherlands by follow-up years. Cancer types were grouped according to the AYA-specific classification scheme developed by Barr and colleagues (2020). First and second cancer combinations with less than n=10 observed second cancers were excluded from the analyses. This Table presents outcomes from the sensitivity analysis.

|  | **Follow-up (years)** | **1-4** | | | | **5-9** | | | | **10-14** | | | | **15-19** | | | | **20-25** | | | |
| --- | --- | --- | --- | --- | --- | --- | --- | --- | --- | --- | --- | --- | --- | --- | --- | --- | --- | --- | --- | --- | --- |
|  |  | **Obs/Exp** | **SIR (95%CI)** | **AER (95%CI)** | **P-value** | **Obs/Exp** | **SIR  (95%CI)** | **AER  (95%CI)** | **P-value** | **Obs/Exp** | **SIR (95%CI)** | **AER (95%CI)** | **P-value** | **Obs/Exp** | **SIR  (95%CI)** | **AER  (95%CI)** | **P-value** | **Obs/Exp** | **SIR  (95%CI)** | **AER  (95%CI)** | **P-value** |
| **Males** | **Total** | 441/109 | 4.1  (3.7, 4.5) | 28.0  (24.6, 31.7) | 0.000 | 484/138 | 3.5 (3.2, 3.8) | 32.3 (28.4, 36.6) | 0.000 | 361/143 | 2.5 (2.3, 2.8) | 30.1 (25.1, 35.5) | 0.000 | 331/145 | 2.3 (2.0, 2.5) | 41.8 (34.0, 50.2) | 0.000 | 256/139 | 1.8 (1.6, 2.1) | 47.0 (34.8, 60.4) | 0.000 |
|  | **Age at diagnosis (years)^a^** |  |  |  |  |  |  |  |  |  |  |  |  |  |  |  |  |  |  |  |  |
|  | 15-19 | 19/3 | 6.1  (3.7, 9.6) | 16.6  (8.7, 27.8) | 0.000 | 27/5 | 5.7 (3.8, 8.3) | 25.2 (14.8, 39.1) | 0.000 | 20/5 | 4.4 (2.7, 6.8) | 25.6 (12.7, 43.6) | 0.000 | 15/4 | 4.2 (2.3, 6.9) | 30.0 (12.7, 55.7) | 0.000 | 3/3 | 1.0 (0.2, 2.9) | -0.2 (-10.6, 25.0) | 1.000 |
|  | 20-24 | 60/9 | 6.8  (5.2, 8.7) | 31.8 (23.0, 42.5) | 0.000 | 51/11 | 4.8 (3.5, 6.3) | 27.8 (18.8, 38.9) | 0.000 | 35/9 | 3.7 (2.6, 5.2) | 25.5 (14.9, 39.1) | 0.000 | 28/8 | 3.4 (2.3, 4.9) | 30.7 (16.1, 50.2) | 0.000 | 27/8 | 3.3 (2.2, 4.8) | 47.5 (24.3, 78.5) | 0.000 |
|  | 25-29 | 86/18 | 4.8  (3.8, 5.9) | 28.4  (21.2, 36.8) | 0.000 | 84/20 | 4.2 (3.3, 5.2) | 29.4 (21.6, 38.6) | 0.000 | 67/19 | 3.5 (2.7, 4.5) | 32.2 (22.1, 44.3) | 0.000 | 41/20 | 2.1 (1.5, 2.8) | 21.8 (10.0, 36.6) | 0.000 | 43/19 | 2.2 (1.6, 3.0) | 41.6 (20.9, 67.6) | 0.000 |
|  | 30-34 | 113/29 | 3.9  (3.2, 4.7) | 27.2  (20.8, 34.6) | 0.000 | 116/36 | 3.3 (2.7, 3.9) | 28.9 (21.6, 37.2) | 0.000 | 87/38 | 2.3 (1.8, 2.8) | 25.5 (16.5, 36.1) | 0.000 | 90/39 | 2.3 (1.9, 2.9) | 43.1 (28.3, 60.5) | 0.000 | 80/40 | 2.0 (1.6, 2.5) | 61.7 (36.2, 91.8) | 0.000 |
|  | 35-39 | 163/50 | 3.3  (2.8, 3.8) | 29.7  (23.4, 36.8) | 0.000 | 206/67 | 3.1 (2.7, 3.5) | 40.8 (32.8, 49.7) | 0.000 | 152/72 | 2.1 (1.8, 2.5) | 36.0 (25.5, 47.8) | 0.000 | 157/75 | 2.1 (1.8, 2.5) | 65.4 (46.6, 86.5) | 0.000 | 103/68 | 1.5 (1.2, 1.8) | 53.4 (24.2, 87.2) | 0.000 |
|  | **Tumour stage (TNM, Figo and Ann arbor)** |  |  |  |  |  |  |  |  |  |  |  |  |  |  |  |  |  |  |  |  |
|  | stage I | 218/54 | 4.1  (3.5, 4.6) | 28.9  (24.0, 34.3) | 0.000 | 239/70 | 3.4 (3.0, 3.9) | 32.2 (26.6, 38.3) | 0.000 | 168/72 | 2.3 (2.0, 2.7) | 27.0 (20.1, 34.7) | 0.000 | 139/73 | 1.9 (1.6, 2.2) | 30.1 (19.9, 41.7) | 0.000 | 114/71 | 1.6 (1.3, 1.9) | 34.9 (18.7, 53.6) | 0.000 |
|  | stage II | 72/18 | 3.9  (3.1, 5.0) | 26.5  (18.8, 35.8) | 0.000 | 78/24 | 3.2 (2.6, 4.0) | 28.5 (19.9, 38.8) | 0.000 | 69/26 | 2.6 (2.0, 3.3) | 32.0 (20.5, 45.7) | 0.000 | 65/27 | 2.4 (1.8, 3.1) | 45.1 (27.4, 66.4) | 0.000 | 50/24 | 2.1 (1.5, 2.7) | 58.8 (29.2, 95.3) | 0.000 |
|  | stage III | 51/11 | 4.5  (3.3, 5.9) | 31.0  (20.8, 43.6) | 0.000 | 47/14 | 3.4 (2.5, 4.5) | 29.7 (18.5, 43.6) | 0.000 | 39/14 | 2.8 (2.0, 3.8) | 33.5 (18.4, 52.7) | 0.000 | 51/13 | 4.0 (3.0, 5.2) | 91.2 (60.1, 129.6) | 0.000 | 22/10 | 2.1 (1.3, 3.2) | 59.3 (17.7, 116.5) | 0.002 |
|  | stage IV | 31/6 | 5.4  (3.7, 7.7) | 40.1  (24.3, 60.7) | 0.000 | 35/7 | 5.3 (3.7, 7.4) | 54.9 (34.4, 81.3) | 0.000 | 23/7 | 3.4 (2.2, 5.2) | 46.4 (22.5, 79.2) | 0.000 | 20/7 | 2.8 (1.7, 4.3) | 54.2 (21.1, 100.5) | 0.000 | 19/10 | 1.9 (1.2, 3.0) | 49.0 (8.4, 106.3) | 0.013 |
|  | Other/unknown | 69/20 | 3.5  (2.7, 4.5) | 22.1  (15.3, 30.3) | 0.000 | 85/24 | 3.5 (2.8, 4.4) | 32.0 (23.0, 42.5) | 0.000 | 62/24 | 2.6 (2.0, 3.3) | 30.2 (18.7, 44.1) | 0.000 | 56/24 | 2.3 (1.7, 3.0) | 40.4 (23.0, 61.7) | 0.000 | 51/23 | 2.2 (1.6, 2.9) | 62.4 (33.3, 98.1) | 0.000 |
|  | **Cancer type** |  |  |  |  |  |  |  |  |  |  |  |  |  |  |  |  |  |  |  |  |
|  | **1. Leukaemia’s and related disorders** | **11/5** | **2.2  (1.1, 3.9)** | **10.1  (0.7, 25.0)** | **0.031** | **22/6** | **3.6 (2.2, 5.4)** | **32.3 (15.5, 55.4)** | **0.000** | **17/6** | **2.9 (1.7, 4.6)** | **36.4 (13.0, 69.9)** | **0.000** | **10/5** | **1.9 (0.9, 3.5)** | **28.1 (-3.1, 78.5)** | **0.090** | **11/5** | **2.4 (1.2, 4.3)** | **75.0 (11.0, 175.9)** | **0.014** |
|  | **2. Lymphomas** | **46/17** | **2.7 (2.0, 3.6)** | **14.6 (8.4, 22.4)** | **0.000** | **68/23** | **3.0 (2.3, 3.8)** | **24.3 (16.2, 34.1)** | **0.000** | **83/24** | **3.5 (2.8, 4.3)** | **45.1 (32.2, 60.3)** | **0.000** | **77/24** | **3.3 (2.6, 4.1)** | **66.2 (46.0, 90.0)** | **0.000** | **61/22** | **2.7 (2.1, 3.5)** | **85.1 (53.6, 123.2)** | **0.000** |
|  | **3. CNS and other intracranial and intraspinal neoplasms** | **16/5** | **3.0 (1.7, 4.9)** | **16.8 (6.0, 32.4)** | **0.000** | **18/5** | **3.4 (2.0, 5.4)** | **28.5 (12.2, 51.9)** | **0.000** | **9/4** | **2.1 (1.0, 4.0)** | **19.3 (-0.6, 52.2)** | **0.061** | **8/4** | **2.1 (0.9, 4.2)** | **29.1 (-2.1, 82.3)** | **0.076** | **7/3** | **2.2 (0.9, 4.5)** | **49.6 (-4.8, 146.0)** | **0.087** |
|  | **4. Sarcomas** | **19/6** | **3.3 (2.0, 5.2)** | **18.9 (8.1, 34.1)** | **0.000** | **15/8** | **2.0 (1.1, 3.2)** | **11.5 (1.2, 26.7)** | **0.023** | **15/8** | **1.8 (1.0, 3.0)** | **15.0 (0.4, 36.5)** | **0.042** | **13/9** | **1.5 (0.8, 2.5)** | **14.1 (-5.9, 44.6)** | **0.210** | **15/10** | **1.6 (0.9, 2.6)** | **28.5 (-6.1, 79.5)** | **0.126** |
|  | **5. Blood and lymphatic vessel tumours** | **15/1** | **10.5 (5.9, 17.3)** | **95.1 (48.8, 163.4)** | **0.000** | **9/2** | **4.9 (2.3, 9.4)** | **57.9 (18.5, 123.2)** | **0.000** | **8/2** | **4.1 (1.8, 8.2)** | **74.2 (18.6, 169.0)** | **0.002** | **4/2** | **2.0 (0.5, 5.2)** | **40.6 (-18.0, 166.0)** | **0.280** | **2/1** | **1.4 (0.2, 5.1)** | **27.3 (-54.6, 270.7)** | **0.827** |
|  | **6. Nerve sheath tumours** | **0/0** | **0.0 (0.0, 14.9)** | **-8.6 (-8.6, 118.9)** | **0.781** | **2/0** | **5.9 (0.7, 21.1)** | **60.6 (-3.6, 251.7)** | **0.093** | **0/0** | **0.0 (0.0, 9.5)** | **-20.0 (-20.0, 169.6)** | **0.678** | **1/0** | **2.2 (0.1, 12.0)** | **44.3 (-36.1, 421.4)** | **0.741** | **0/0** | **0.0 (0.0, 7.8)** | **-57.7 (-57.7, 391.7)** | **0.623** |
|  | **7. Gonadal and related tumours** | **148/35** | **4.3 (3.6, 5.0)** | **28.5 (22.7, 35.0)** | **0.000** | **167/44** | **3.8 (3.2, 4.4)** | **33.1 (26.5, 40.4)** | **0.000** | **97/46** | **2.1 (1.7, 2.6)** | **20.3 (13.0, 28.7)** | **0.000** | **83/46** | **1.8 (1.4, 2.2)** | **23.7 (12.8, 36.5)** | **0.000** | **66/44** | **1.5 (1.2, 1.9)** | **25.6 (8.2, 46.4)** | **0.002** |
|  | **8. Melanoma, malignant** | **78/19** | **4.1 (3.2, 5.1)** | **31.2 (22.5, 41.4)** | **0.000** | **80/25** | **3.3 (2.6, 4.1)** | **32.1 (22.5, 43.5)** | **0.000** | **61/26** | **2.4 (1.8, 3.0)** | **30.3 (18.0, 45.2)** | **0.000** | **53/26** | **2.1 (1.5, 2.7)** | **38.6 (19.8, 61.7)** | **0.000** | **35/24** | **1.5 (1.0, 2.0)** | **28.5 (1.1, 63.7)** | **0.040** |
|  | **9. Carcinomas** | **107/20** | **5.4 (4.4, 6.5)** | **46.7 (36.4, 58.6)** | **0.000** | **103/25** | **4.1 (3.4, 5.0)** | **47.8 (36.2, 61.3)** | **0.000** | **70/27** | **2.6 (2.0, 3.3)** | **39.1 (25.1, 55.8)** | **0.000** | **82/29** | **2.8 (2.3, 3.5)** | **76.6 (52.5, 105.1)** | **0.000** | **59/29** | **2.0 (1.6, 2.6)** | **74.6 (39.6, 117.1)** | **0.000** |
|  | 9.1 Thyroid carcinoma | 6/3 | 2.3 (0.8, 4.9) | 12.0 (-1.6, 37.3) | 0.104 | 7/4 | 2.0 (0.8, 4.1) | 13.3 (-2.7, 41.6) | 0.134 | 4/4 | 1.0 (0.3, 2.7) | 1.0 (-14.9, 35.1) | 1.000 | 10/4 | 2.4 (1.1, 4.4) | 47.8 (5.1, 116.7) | 0.021 | 6/4 | 1.4 (0.5, 3.0) | 21.5 (-29.5, 116.2) | 0.560 |
|  | 9.2 Other carcinoma of head and neck | 19/3 | 5.8 (3.5, 9.0) | 50.0 (25.9, 83.9) | 0.000 | 22/5 | 4.9 (3.1, 7.4) | 59.5 (31.6, 97.9) | 0.000 | 24/5 | 4.8 (3.1, 7.2) | 94.3 (51.6, 152.2) | 0.000 | 21/5 | 4.2 (2.6, 6.4) | 138.1 (69.1, 233.9) | 0.000 | 9/5 | 1.8 (0.8, 3.5) | 63.1 (-12.7, 188.4) | 0.127 |
|  | 9.3 Carcinoma of gastrointestinal tract | 42/7 | 5.8 (4.2, 7.8) | 50.6 (33.5, 72.1) | 0.000 | 35/9 | 4.0 (2.8, 5.6) | 46.8 (27.9, 71.1) | 0.000 | 19/9 | 2.0 (1.2, 3.2) | 25.2 (5.5, 53.0) | 0.007 | 25/10 | 2.4 (1.6, 3.6) | 58.4 (23.4, 105.7) | 0.000 | 24/11 | 2.2 (1.4, 3.3) | 86.4 (30.0, 162.8) | 0.001 |
|  | 9.4 Carcinoma of lung, bronchus, and trachea | 8/1 | 7.4 (3.2, 14.6) | 67.4 (23.1, 143.0) | 0.000 | 4/1 | 3.2 (0.9, 8.1) | 33.8 (-2.1, 110.7) | 0.078 | 5/1 | 3.8 (1.2, 8.9) | 70.7 (6.0, 198.3) | 0.022 | 5/1 | 3.9 (1.3, 9.1) | 119.3 (10.8, 333.8) | 0.021 | 4/1 | 3.6 (1.0, 9.2) | 182.7 (-1.7, 578.3) | 0.054 |
|  | 9.5 Carcinoma of skin (if collected) | 13/2 | 7.5 (4.0, 12.8) | 71.8 (33.1, 130.7) | 0.000 | 11/2 | 4.8 (2.4, 8.5) | 61.1 (22.4, 122.1) | 0.000 | 7/2 | 2.8 (1.1, 5.8) | 47.4 (3.6, 124.9) | 0.027 | 7/2 | 2.8 (1.1, 5.8) | 78.4 (5.7, 207.4) | 0.028 | 5/2 | 2.2 (0.7, 5.0) | 86.7 (-22.5, 302.3) | 0.172 |
|  | 9.6 Carcinoma of breast | 0/0 | 0.0 (0.0, 29.0) | -10.7 (-10.7, 299.0) | 0.881 | 0/0 | 0.0 (0.0, 25.5) | -15.7 (-15.7, 384.2) | 0.865 | 0/0 | 0.0 (0.0, 22.0) | -25.8 (-25.8, 541.0) | 0.846 | 1/0 | 8.3 (0.2, 46.1) | 277.7 (-30.2, 1722.0) | 0.228 | 0/0 | 0.0 (0.0, 38.1) | -60.1 (-60.1, 2231.4) | 0.908 |
|  | 9.7 Carcinoma of genital sites excluding ovary and testis | 2/0 | 6.0 (0.7, 21.7) | 60.0 (-3.3, 247.9) | 0.089 | 1/0 | 2.3 (0.1, 12.8) | 22.9 (-16.6, 208.4) | 0.705 | 0/0 | 0.0 (0.0, 8.7) | -28.6 (-28.6, 221.1) | 0.655 | 1/1 | 1.9 (0.0, 10.5) | 45.5 (-49.0, 488.6) | 0.824 | 0/0 | 0.0 (0.0, 8.9) | -87.2 (-87.2, 688.0) | 0.660 |
|  | 9.8 Carcinoma of urinary tract | 16/3 | 5.7 (3.3, 9.2) | 54.1 (26.0, 95.1) | 0.000 | 18/4 | 4.9 (2.9, 7.7) | 66.2 (32.3, 114.6) | 0.000 | 11/4 | 2.8 (1.4, 5.1) | 50.1 (11.4, 111.0) | 0.004 | 11/4 | 2.6 (1.3, 4.6) | 75.4 (13.5, 172.9) | 0.009 | 8/4 | 1.8 (0.8, 3.5) | 67.5 (-19.3, 215.6) | 0.167 |
|  | 9.9 Other invasive carcinomas | 1/0 | 2.1 (0.1, 11.9) | 11.8 (-9.8, 112.8) | 0.747 | 5/1 | 9.4 (3.1, 22.0) | 124.1 (30.3, 309.3) | 0.000 | 0/1 | 0.0 (0.0, 6.6) | -23.7 (-23.7, 133.0) | 0.573 | 1/1 | 1.6 (0.0, 9.0) | 28.2 (-43.5, 364.5) | 0.921 | 3/0 | 7.1 (1.5, 20.6) | 502.0 (37.8, 1626.1) | 0.019 |
|  | **10. Miscellaneous specified neoplasms** | **0/0** | **0.0 (0.0, 20.0)** | **-8.5 (-8.5, 161.5)** | **0.831** | **0/0** | **0.0 (0.0, 16.2)** | **-12.4 (-12.4, 187.2)** | **0.796** | **0/0** | **0.0 (0.0, 14.1)** | **-19.7 (-19.7, 258.1)** | **0.769** | **0/0** | **0.0 (0.0, 11.8)** | **-34.5 (-34.5, 373.4)** | **0.732** | **0/0** | **0.0 (0.0, 14.7)** | **-59.2 (-59.2, 808.5)** | **0.778** |
|  | **11. Unspecified malignant neoplasms except CNS** | **1/0** | **6.5 (0.2, 36)** | **45.6 (-7.0, 292)** | **0.287** | **0/0** | **0.0 (0.0, 21.7)** | **-11.1 (-11.1, 230.1)** | **0.843** | **1/0** | **6.9 (0.2, 38.3)** | **89.0 (-12.5, 565.0)** | **0.271** | **0/0** | **0.0 (0.0, 35.3)** | **-22.4 (-22.4, 769.5)** | **0.901** | **0/0** | **0.0 (0.0, 52.5)** | **-53.5 (-53.5, 2758.4)** | **0.932** |
| **Females** | **Total** | 1,006/339 | 3.0 (2.8, 3.2) | 36.7 (33.3, 40.2) | 0.000 | 1,056/469 | 2.3 (2.1, 2.4) | 35.7 (31.9, 39.7) | 0.000 | 896/476 | 1.9 (1.8, 2.0) | 37.7 (32.5, 43.2) | 0.000 | 744/414 | 1.8 (1.7, 1.9) | 47.4 (39.9, 55.4) | 0.000 | 527/314 | 1.7 (1.5, 1.8) | 54.6 (43.4, 66.7) | 0.000 |
|  | **Age at diagnosis (years)^a^** |  |  |  |  |  |  |  |  |  |  |  |  |  |  |  |  |  |  |  |  |
|  | 15-19 | 16/2 | 7.4 (4.2, 12.0) | 17.3 (8.7, 29.8) | 0.000 | 22/4 | 6.0 (3.8, 9.1) | 24.7 (13.6, 39.9) | 0.000 | 21/5 | 4.3 (2.7, 6.6) | 31.5 (15.9, 53.2) | 0.000 | 16/6 | 2.9 (1.6, 4.7) | 30.4 (10.5, 59.5) | 0.000 | 17/5 | 3.1 (1.8, 5.0) | 55.3 (21.3, 104.2) | 0.000 |
|  | 20-24 | 40/7 | 5.4 (3.8, 7.3) | 23.4 (15.2, 33.7) | 0.000 | 40/12 | 3.2 (2.3, 4.4) | 21.5 (12.6, 32.8) | 0.000 | 43/15 | 2.9 (2.1, 3.9) | 31.3 (18.2, 47.8) | 0.000 | 36/16 | 2.3 (1.6, 3.2) | 34.0 (16.1, 56.9) | 0.000 | 49/16 | 3.2 (2.3, 4.2) | 89.8 (55.6, 132.1) | 0.000 |
|  | 25-29 | 112/27 | 4.2 (3.4, 5.0) | 32.3 (24.8, 40.9) | 0.000 | 126/39 | 3.2 (2.7, 3.9) | 36.3 (27.5, 46.3) | 0.000 | 109/43 | 2.5 (2.1, 3.0) | 39.1 (27.5, 52.5) | 0.000 | 106/46 | 2.3 (1.9, 2.8) | 53.3 (36.2, 72.9) | 0.000 | 70/39 | 1.8 (1.4, 2.3) | 47.3 (23.7, 75.5) | 0.000 |
|  | 30-34 | 292/85 | 3.5 (3.1, 3.9) | 41.0 (34.6, 48.0) | 0.000 | 268/117 | 2.3 (2.0, 2.6) | 32.9 (26.1, 40.3) | 0.000 | 234/130 | 1.8 (1.6, 2.0) | 32.4 (23.4, 42.4) | 0.000 | 211/119 | 1.8 (1.5, 2.0) | 45.8 (32.1, 60.9) | 0.000 | 123/89 | 1.4 (1.1, 1.6) | 30.8 (12.0, 52.3) | 0.001 |
|  | 35-39 | 546/218 | 2.5 (2.3, 2.7) | 39.5 (34.1, 45.3) | 0.000 | 600/297 | 2.0 (1.9, 2.2) | 40.9 (34.5, 47.6) | 0.000 | 489/284 | 1.7 (1.6, 1.9) | 42.6 (33.8, 52.0) | 0.000 | 375/228 | 1.6 (1.5, 1.8) | 51.1 (38.2, 64.9) | 0.000 | 268/165 | 1.6 (1.4, 1.8) | 66.1 (46.2, 88.0) | 0.000 |
|  | **Tumour stage (TNM, Figo and Ann arbor)** |  |  |  |  |  |  |  |  |  |  |  |  |  |  |  |  |  |  |  |  |
|  | stage I | 439/170 | 2.6 (2.3, 2.8) | 29.7 (25.2, 34.4) | 0.000 | 543/246 | 2.2 (2.0, 2.4) | 34.8 (29.5, 40.4) | 0.000 | 460/255 | 1.8 (1.6, 2.0) | 34.9 (27.9, 42.4) | 0.000 | 346/228 | 1.5 (1.4, 1.7) | 31.4 (21.9, 41.6) | 0.000 | 252/179 | 1.4 (1.2, 1.6) | 33.2 (19.4, 48.4) | 0.000 |
|  | stage II | 343/97 | 3.6 (3.2, 3.9) | 49.9 (42.7, 57.6) | 0.000 | 344/132 | 2.6 (2.3, 2.9) | 48.5 (40.4, 57.2) | 0.000 | 266/132 | 2.0 (1.8, 2.3) | 45.3 (34.8, 56.8) | 0.000 | 241/109 | 2.2 (1.9, 2.5) | 74.0 (57.5, 92.2) | 0.000 | 164/79 | 2.1 (1.8, 2.4) | 90.2 (64.7, 118.9) | 0.000 |
|  | stage III | 116/29 | 3.9 (3.3, 4.7) | 57.0 (43.7, 72.2) | 0.000 | 59/34 | 1.7 (1.3, 2.3) | 21.7 (9.6, 36.4) | 0.000 | 71/28 | 2.5 (2.0, 3.2) | 63.9 (40.6, 91.8) | 0.000 | 55/20 | 2.8 (2.1, 3.6) | 102.4 (62.8, 150.9) | 0.000 | 31/14 | 2.3 (1.5, 3.2) | 99.5 (42.3, 174.5) | 0.000 |
|  | stage IV | 25/9 | 2.7 (1.7, 4.0) | 29.5 (13.0, 51.7) | 0.000 | 22/9 | 2.4 (1.5, 3.6) | 35.8 (12.9, 67.3) | 0.000 | 19/8 | 2.3 (1.4, 3.6) | 50.3 (15.0, 100.2) | 0.002 | 15/6 | 2.3 (1.3, 3.8) | 71.6 (16.3, 153.1) | 0.006 | 17/5 | 3.5 (2.1, 5.6) | 185.4 (77.4, 341.0) | 0.000 |
|  | Other/unknown | 83/33 | 2.5 (2.0, 3.1) | 23.2 (15.3, 32.5) | 0.000 | 88/48 | 1.8 (1.5, 2.3) | 20.0 (11.3, 30.2) | 0.000 | 80/52 | 1.5 (1.2, 1.9) | 19.5 (7.9, 33.2) | 0.000 | 87/50 | 1.7 (1.4, 2.1) | 38.2 (20.4, 59.2) | 0.000 | 63/37 | 1.7 (1.3, 2.2) | 48.7 (21.3, 81.8) | 0.000 |
|  | **Cancer type** |  |  |  |  |  |  |  |  |  |  |  |  |  |  |  |  |  |  |  |  |
|  | **1. Leukaemia’s and related disorders** | **19/8** | **2.5 (1.5, 3.9)** | **22.2 (7.4, 43.0)** | **0.001** | **13/10** | **1.3 (0.7, 2.2)** | **6.5 (-7.4, 27.5)** | **0.445** | **9/9** | **1.0 (0.5, 1.9)** | **-0.2 (-18.8, 30.5)** | **1.000** | **8/7** | **1.2 (0.5, 2.4)** | **10.0 (-23.0, 66.4)** | **0.691** | **10/5** | **2.2 (1.1, 4.0)** | **82.7 (3.8, 209.8)** | **0.036** |
|  | **2. Lymphomas** | **25/19** | **1.3 (0.9, 2.0)** | **4.2 (-1.8, 12.2)** | **0.200** | **64/28** | **2.3 (1.8, 2.9)** | **25.7 (15.3, 38.3)** | **0.000** | **77/30** | **2.6 (2.0, 3.2)** | **47.7 (31.1, 67.3)** | **0.000** | **91/27** | **3.3 (2.7, 4.1)** | **102.5 (73.9, 136.0)** | **0.000** | **65/20** | **3.2 (2.5, 4.1)** | **135 (90.1, 189.1)** | **0.000** |
|  | **3. CNS and other intracranial and intraspinal neoplasms** | **12/6** | **1.9 (1.0, 3.3)** | **12.2 (-0.4, 31.8)** | **0.061** | **10/7** | **1.4 (0.7, 2.6)** | **8.5 (-6.9, 33.3)** | **0.367** | **9/6** | **1.5 (0.7, 2.8)** | **14.4 (-10.4, 55.4)** | **0.341** | **16/5** | **3.3 (1.9, 5.4)** | **98.5 (38.3, 186.2)** | **0.000** | **3/4** | **0.8 (0.2, 2.4)** | **-10.1 (-46.6, 78.4)** | **1.000** |
|  | **4. Sarcomas** | **20/10** | **2.0 (1.2, 3.1)** | **14.8 (3.4, 30.7)** | **0.006** | **35/14** | **2.4 (1.7, 3.4)** | **32.3 (15.6, 53.7)** | **0.000** | **21/15** | **1.4 (0.8, 2.1)** | **12.9 (-5.2, 37.9)** | **0.193** | **22/14** | **1.6 (1.0, 2.4)** | **28.4 (-0.3, 67.9)** | **0.053** | **13/11** | **1.2 (0.6, 2.0)** | **11.4 (-24.7, 66.2)** | **0.639** |
|  | **5. Blood and lymphatic vessel tumours** | **2/0** | **4.1 (0.5, 14.9)** | **48.0 (-7.7, 213.5)** | **0.172** | **0/1** | **0.0 (0.0, 5.4)** | **-24.8 (-24.8, 108.5)** | **0.503** | **1/1** | **1.3 (0.0, 7.4)** | **12.8 (-37.9, 250.4)** | **1.000** | **0/1** | **0.0 (0.0, 7.0)** | **-53.6 (-53.6, 322.6)** | **0.591** | **1/0** | **2.0 (0.1, 11.3)** | **78.1 (-72.5, 784.4)** | **0.780** |
|  | **6. Nerve sheath tumours** | **4/0** | **14.2 (3.9, 36.4)** | **174.7 (38.0, 467.9)** | **0.000** | **0/0** | **0.0 (0.0, 8.7)** | **-21.5 (-21.5, 166.4)** | **0.656** | **0/1** | **0.0 (0.0, 6.7)** | **-35.6 (-35.6, 204.6)** | **0.579** | **1/0** | **2.1 (0.1, 11.8)** | **61.9 (-52.8, 599.5)** | **0.755** | **0/0** | **0.0 (0.0, 11.7)** | **-71 (-71, 759.4)** | **0.730** |
|  | **7. Gonadal and related tumours** | **20/12** | **1.7 (1.1, 2.7)** | **12.1 (1.0, 27.6)** | **0.029** | **25/18** | **1.4 (0.9, 2.1)** | **10.2 (-2.4, 27.1)** | **0.128** | **38/22** | **1.7 (1.2, 2.3)** | **27.4 (8.0, 52.2)** | **0.003** | **30/24** | **1.2 (0.8, 1.8)** | **13.3 (-9.2, 42.8)** | **0.285** | **26/18** | **1.4 (0.9, 2.1)** | **31.3 (-5.8, 81)** | **0.109** |
|  | **8. Melanoma, malignant** | **200/64** | **3.1 (2.7, 3.6)** | **36.6 (29.4, 44.6)** | **0.000** | **216/93** | **2.3 (2.0, 2.6)** | **34.6 (26.7, 43.3)** | **0.000** | **174/95** | **1.8 (1.6, 2.1)** | **32.4 (22.2, 43.9)** | **0.000** | **133/81** | **1.6 (1.4, 1.9)** | **35.0 (20.4, 51.6)** | **0.000** | **75/62** | **1.2 (1.0, 1.5)** | **16 (-3.4, 39)** | **0.113** |
|  | **9. Carcinomas** | **700/219** | **3.2 (3.0, 3.4)** | **45.6 (40.8, 50.7)** | **0.000** | **690/296** | **2.3 (2.2, 2.5)** | **42.5 (37.1, 48.3)** | **0.000** | **565/295** | **1.9 (1.8, 2.1)** | **43.7 (36.3, 51.5)** | **0.000** | **439/253** | **1.7 (1.6, 1.9)** | **48.3 (37.9, 59.4)** | **0.000** | **332/192** | **1.7 (1.5, 1.9)** | **64.4 (48.4, 81.7)** | **0.000** |
|  | 9.1 Thyroid carcinoma | 13/15 | 0.9 (0.5, 1.5) | -1.9 (-8.6, 8.3) | 0.785 | 28/21 | 1.3 (0.9, 1.9) | 7.9 (-3.0, 22.5) | 0.180 | 25/23 | 1.1 (0.7, 1.6) | 3.7 (-10.9, 23.5) | 0.690 | 26/20 | 1.3 (0.8, 1.9) | 15.2 (-8.5, 47.0) | 0.245 | 25/16 | 1.6 (1.0, 2.3) | 41.3 (0.9, 95.9) | 0.044 |
|  | 9.2 Other carcinoma of head and neck | 23/4 | 5.4 (3.4, 8.0) | 77.3 (42.5, 124.8) | 0.000 | 15/6 | 2.4 (1.4, 4.0) | 38.6 (9.7, 81.1) | 0.004 | 23/6 | 3.7 (2.3, 5.5) | 108.3 (53.8, 182.8) | 0.000 | 13/5 | 2.4 (1.3, 4.1) | 79.3 (15.9, 175.6) | 0.008 | 12/4 | 2.9 (1.5, 5.0) | 142.4 (36.8, 305.6) | 0.003 |
|  | 9.3 Carcinoma of gastrointestinal tract | 36/13 | 2.7 (1.9, 3.7) | 30.6 (16.0, 49.3) | 0.000 | 21/18 | 1.2 (0.7, 1.8) | 5.5 (-7.3, 23.4) | 0.470 | 41/18 | 2.3 (1.6, 3.1) | 53.5 (26.7, 87.4) | 0.000 | 20/17 | 1.2 (0.7, 1.9) | 11.3 (-14.9, 48.0) | 0.471 | 21/13 | 1.6 (1.0, 2.5) | 45.2 (-0.2, 108.2) | 0.051 |
|  | 9.4 Carcinoma of lung, bronchus, and trachea | 8/3 | 2.8 (1.2, 5.4) | 34.2 (3.7, 86.5) | 0.020 | 10/4 | 2.6 (1.3, 4.9) | 48.8 (8.0, 114.8) | 0.011 | 3/4 | 0.7 (0.2, 2.1) | -12.1 (-38.6, 52.1) | 0.833 | 6/4 | 1.6 (0.6, 3.4) | 36.2 (-26.3, 152.3) | 0.370 | 5/3 | 1.7 (0.5, 3.9) | 54.1 (-38.5, 237.0) | 0.378 |
|  | 9.5 Carcinoma of skin (if collected) | 12/4 | 3.2 (1.7, 5.6) | 46.7 (13.9, 97.5) | 0.001 | 15/5 | 2.9 (1.6, 4.7) | 59.5 (19.3, 118.7) | 0.001 | 4/5 | 0.7 (0.2, 1.9) | -12.0 (-37.7, 43.1) | 0.760 | 8/5 | 1.7 (0.7, 3.3) | 43.3 (-18.3, 148.6) | 0.228 | 7/3 | 2.2 (0.9, 4.6) | 101.3 (-9.4, 297.5) | 0.086 |
|  | 9.6 Carcinoma of breast | 519/132 | 3.9 (3.6, 4.3) | 65.1 (57.7, 72.9) | 0.000 | 497/172 | 2.9 (2.6, 3.2) | 65.3 (56.7, 74.5) | 0.000 | 366/160 | 2.3 (2.1, 2.5) | 66.7 (54.8, 79.4) | 0.000 | 261/125 | 2.1 (1.8, 2.4) | 77.3 (59.8, 96.4) | 0.000 | 181/89 | 2.0 (1.8, 2.4) | 99.7 (72.3, 130.4) | 0.000 |
|  | 9.7 Carcinoma of genital sites excluding ovary and testis | 73/43 | 1.7 (1.3, 2.1) | 13.7 (6.4, 22.3) | 0.000 | 92/64 | 1.4 (1.2, 1.8) | 13.1 (4.7, 23.0) | 0.001 | 94/73 | 1.3 (1.0, 1.6) | 13.3 (1.9, 26.7) | 0.021 | 96/72 | 1.3 (1.1, 1.6) | 21.7 (5.2, 40.9) | 0.008 | 77/60 | 1.3 (1.0, 1.6) | 24.9 (1.0, 53.2) | 0.041 |
|  | 9.8 Carcinoma of urinary tract | 9/3 | 3.0 (1.4, 5.6) | 38.9 (7.0, 91.6) | 0.008 | 8/4 | 1.9 (0.8, 3.7) | 27.4 (-5.7, 83.8) | 0.132 | 8/4 | 1.9 (0.8, 3.7) | 40.0 (-8.8, 123.3) | 0.138 | 7/4 | 1.8 (0.7, 3.8) | 51.7 (-16.8, 173.3) | 0.190 | 4/3 | 1.3 (0.4, 3.4) | 26.0 (-54.0, 197.6) | 0.730 |
|  | 9.9 Other invasive carcinomas | 7/1 | 5.6 (2.3, 11.6) | 93.1 (25.4, 213.1) | 0.001 | 4/2 | 2.6 (0.7, 6.6) | 50.6 (-9.6, 179.8) | 0.145 | 1/2 | 0.7 (0.0, 3.7) | -16.0 (-46.7, 127.6) | 1.000 | 2/1 | 1.6 (0.2, 5.9) | 41.3 (-52.5, 320.1) | 0.693 | 0/1 | 0.0 (0.0, 4.3) | -86.2 (-86.2, 284.0) | 0.423 |
|  | **10. Miscellaneous specified neoplasms** | **2/1** | **3.1 (0.4, 11.3)** | **34.1 (-9.9, 164.8)** | **0.270** | **1/1** | **1.1 (0.0, 6.2)** | **2.8 (-25.2, 134.0)** | **1.000** | **0/1** | **0.0 (0.0, 3.2)** | **-40.5 (-40.5, 89.7)** | **0.317** | **4/1** | **3.4 (0.9, 8.6)** | **133.3 (-4.8, 429.4)** | **0.066** | **2/1** | **2.1 (0.3, 7.8)** | **88.9 (-57.2, 523.0)** | **0.478** |
|  | **11. Unspecified malignant neoplasms except CNS** | **2/0** | **10.1 (1.2, 36.4)** | **171 (4.1, 667.0)** | **0.035** | **2/0** | **6.9 (0.8, 24.9)** | **167.1 (-4.7, 678.0)** | **0.070** | **2/0** | **6.7 (0.8, 24.1)** | **221.0 (-7.5, 900.5)** | **0.074** | **0/0** | **0.0 (0.0, 14.5)** | **-55.8 (-55.8, 753.3)** | **0.776** | **0/0** | **0.0 (0.0, 13.4)** | **-81.8 (-81.8, 1,011.4)** | **0.759** |

Abbreviation: TNM=Tumour, Node, Metastasis, Figo=Fédération Internationale de Gynécologie et d'Obstétrique, CNS=Central Nervous System.

^a^ Age at diagnosis of first and second primary cancer.

**Supplementary Table S3A.** Standardised incidence ratios (SIRs) and absolute excess risks (AERs) of second primary cancer diagnosis after any first primary cancer among six-month adolescent and young adult (AYA, aged 15-39 years) cancer survivors compared to the general population in the Netherlands. Second primary cancer types are grouped according to the AYA-specific classification scheme developed by Barr and colleagues (2020). Cancer combinations with less than n=10 observed second cancers were excluded from the analyses. This Table presents outcomes from the main analysis.

| **Second primary cancers** | **Cancer risk after any first cancer** | | | | | | | |
| --- | --- | --- | --- | --- | --- | --- | --- | --- |
|  | **Males** | | | | **Females** | | | |
|  | **Obs/exp** | **SIR (95%CI)** | **AER per 10,000 person-years (95%CI)** | **P-value** | **Obs/exp** | **SIR (95%CI)** | **AER per 10,000 person-years (95%CI)** | **P-value** |
| **Total** | **1,431/714** | **2.0 (1.9, 2.1)** | **17.5 (15.7, 19.3)** | **0.000** | **2,802/2,161** | **1.3 (1.2, 1.3)** | **10.1 (8.5, 11.7)** | **0.000** |
| **Cancer types** |  |  |  |  |  |  |  |  |
| **1. Leukaemia’s and related disorders** | **98/38** | **2.6 (2.1, 3.1)** | **1.5 (1.0, 2.0)** | **0.000** | **97/47** | **2.0. (1.7, 2.5)** | **0.8 (0.5, 1.1)** | **0.000** |
| 1.1 Acute lymphoblastic leukaemia | 13/3 | 4.1 (2.2, 7.0) | 0.2 (0.1, 0.5) | 0.000 | NA | NA | NA | NA |
| 1.2 Acute myeloid leukaemia | 37/8 | 4.5 (3.2, 6.2) | 0.7 (0.4, 1.0) | 0.000 | 41/12 | 3.5 (2.5, 4.7) | 0.5 (0.3, 0.7) | 0.000 |
| 1.2.2 Other acute myeloid leukaemia | 35/7 | 4.9 (3.4, 6.8) | 0.7 (0.4, 1.0) | 0.000 | 40/10 | 3.8 (2.7, 5.2) | 0.5 (0.3, 0.7) | 0.000 |
| 1.3 Chronic myeloid leukaemia | 11/4 | 2.5 (1.2, 4.4) | 0.2 (0.0, 0.4) | 0.012 | 11/5 | 2.1 (1.1, 3.8) | 0.1 (0.0, 0.2) | 0.034 |
| 1.4 Chronic lymphocytic leukaemia | NA | NA | NA | NA | 10/6 | 1.8 (0.8, 3.3) | 0.1 (0.0, 0.2) | 0.123 |
| 1.8 Myelodysplastic syndrome (MDS) | 15/3 | 5.9 (3.3, 9.7) | 0.3 (0.1, 0.5) | 0.000 | 13/4 | 3.6 (1.9, 6.2) | 0.1 (0.1, 0.3) | 0.000 |
| **2. Lymphomas** | **127/69** | **1.8 (1.5, 2.2)** | **1.4 (0.9, 2.0)** | **0.000** | **93/75** | **1.2 (1.0, 1.5)** | **0.3 (0.0, 0.6)** | **0.054** |
| 2.1 Non-Hodgkin lymphomas | 83/40 | 2.1 (1.7, 2.6) | 1.0 (0.6, 1.5) | 0.000 | 79/47 | 1.7 (1.3, 2.1) | 0.5 (0.2, 0.8) | 0.000 |
| 2.1.3 Diffuse large B-cell (DLBCL) | 49/17 | 2.9 (2.1, 3.8) | 0.8 (0.5, 1.2) | 0.000 | 35/18 | 2.0 (1.4, 2.8) | 0.3 (0.1, 0.5) | 0.000 |
| 2.1.5 Anaplastic T-cell and null-cell excluding NK/T-cell | NA | NA | NA | NA | 10/6 | 1.7 (0.8, 3.1) | 0.1 (0.0, 0.2) | 0.159 |
| 2.1.6 Follicular | NA | NA | NA | NA | 19/11 | 1.7 (1.0, 2.6) | 0.1 (0.0, 0.3) | 0.048 |
| 2.1.8 MALT (Mucosa-associated lymphoid tissue) | NA | NA | NA | NA | 10/6 | 1.8 (0.8, 3.2) | 0.1 (0.0, 0.2) | 0.129 |
| 2.2 Hodgkin lymphoma | 30/17 | 1.8 (1.2, 2.6) | 0.3 (0.1, 0.6) | 0.004 | NA | NA | NA | NA |
| 2.2.2 Hodgkin classic, other | 27/14 | 1.9 (1.3, 2.8) | 0.3 (0.1, 0.6) | 0.003 | NA | NA | NA | NA |
| 2.3 Myeloma | NA | NA | NA | NA | 10/11 | 0.9 (0.4, 1.6) | 0.0 (-0.1, 0.1) | 0.884 |
| **3. CNS and other intracranial and intraspinal neoplasms** | **41/27** | **1.5 (1.1, 2.1)** | **0.3 (0.1, 0.7)** | **0.013** | **39/27** | **1.4 (1.0, 2.0)** | **0.2 (0.0, 0.4)** | **0.037** |
| 3.1 Astroglial and related neoplasms | 36/24 | 1.5 (1.1, 2.1) | 0.3 (0.0, 0.6) | 0.025 | 33/25 | 1.3 (0.9, 1.9) | 0.1 (0.0, 0.3) | 0.126 |
| 3.1.2 Glioblastomas/gliofibromas | 16/12 | 1.3 (0.7, 2.1) | 0.1 (-0.1, 0.3) | 0.378 | 15/11 | 1.3 (0.8, 2.2) | 0.1 (0.0, 0.2) | 0.312 |
| 3.1.2.2 Glioblastoma, invasive | 16/12 | 1.3 (0.7, 2.1) | 0.1 (-0.1, 0.3) | 0.378 | 15/11 | 1.3 (0.8, 2.2) | 0.1 (0.0, 0.2) | 0.312 |
| 3.1.4 Other astrocytoma/astroglial neoplasms | 15/6 | 2.5 (1.4, 4.1) | 0.2 (0.1, 0.5) | 0.003 | 10/7 | 1.4 (0.6, 2.5) | 0.0 (0.0, 0.2) | 0.426 |
| 3.1.4.3 Other astrocytoma/astroglial, invasive | 15/6 | 2.5 (1.4, 4.1) | 0.2 (0.1, 0.5) | 0.003 | 10/7 | 1.4 (0.6, 2.5) | 0.0 (0.0, 0.2) | 0.423 |
| **4. Sarcomas** | **52/16** | **3.2 (2.4, 4.2)** | **0.9 (0.5, 1.3)** | **0.000** | **105/28** | **3.8 (3.1, 4.6)** | **1.2 (0.9, 1.6)** | **0.000** |
| 4.1 Osteosarcoma | NA | NA | NA | NA | 11/1 | 10.1 (5.1, 18.1) | 0.2 (0.1, 0.3) | 0.000 |
| 4.2 Chondrosarcoma | NA | NA | NA | NA | 21/2 | 8.7 (5.4, 13.3) | 0.3 (0.2, 0.5) | 0.000 |
| 4.4 Fibromatous neoplasms | NA | NA | NA | NA | 14/6 | 2.4 (1.3, 4.1) | 0.1 (0.0, 0.3) | 0.005 |
| 4.7 Leiomyosarcoma | NA | NA | NA | NA | 10/4 | 2.2 (1.1, 4.1) | 0.1 (0.0, 0.2) | 0.032 |
| 4.15 Other soft tissue sarcomas | 12/1 | 12.9 (6.7, 22.6) | 0.3 (0.1, 0.5) | 0.000 | 13/2 | 5.7 (3.1, 9.8) | 0.2 (0.1, 0.3) | 0.000 |
| **5. Blood and lymphatic vessel tumours** | **16/3** | **4.7 (2.7, 7.6)** | **0.3 (0.1, 0.6)** | **0.000** | **10/2** | **4.5 (2.2, 8.3)** | **0.1 (0.0, 0.3)** | **0.000** |
| 5.2 Malignant blood and lymphatic vessel tumours, all sites | 16/3 | 4.7 (2.7, 7.6) | 0.3 (0.1, 0.6) | 0.000 | 10/2 | 4.5 (2.2, 8.3) | 0.1 (0.0, 0.3) | 0.000 |
| 5.2.2 Other | 10/1 | 12.3 (5.9, 22.6) | 0.2 (0.1, 0.4) | 0.000 | 10/2 | 6.5 (3.1, 12.0) | 0.1 (0.1, 0.3) | 0.000 |
| **6. Nerve sheath tumours** | **NA** | **NA** | **NA** | **NA** | **14/1** | **11.1 (6.1, 18.7)** | **0.2 (0.1, 0.3)** | **0.000** |
| 6.2 Malignant | NA | NA | NA | NA | 14/1 | 11.1 (6.1, 18.7) | 0.2 (0.1, 0.3) | 0.000 |
| 6.2.1 MPNST (Malignant peripheral nerve sheath tumour) | NA | NA | NA | NA | 14/1 | 11.4 (6.2, 19.1) | 0.2 (0.1, 0.3) | 0.000 |
| 6.2.1.2 Peripheral | NA | NA | NA | NA | 14/1 | 11.5 (6.3, 19.3) | 0.2 (0.1, 0.3) | 0.000 |
| **7. Gonadal and related tumours** | **55/87** | **0.6 (0.5, 0.8)** | **-0.8 (-1.1, -0.4)** | **0.000** | **123/47** | **2.6 (2.2, 3.1)** | **1.2 (0.9, 1.6)** | **0.000** |
| 7.1 Testis | 52/85 | 0.6 (0.5, 0.8) | -0.8 (-1.1, -0.4) | 0.000 | NA | NA | NA | NA |
| 7.1.1 Germ cell and trophoblastic | 52/84 | 0.6 (0.5, 0.8) | -0.8 (-1.1, -0.4) | 0.000 | NA | NA | NA | NA |
| 7.1.1.1 Seminoma | 33/54 | 0.6 (0.4, 0.9) | -0.5 (-0.8, -0.2) | 0.003 | NA | NA | NA | NA |
| 7.1.1.5 Mixed germ cell | 14/18 | 0.8 (0.4, 1.3) | -0.1 (-0.2, 0.1) | 0.444 | NA | NA | NA | NA |
| 7.2 Ovary | NA | NA | NA | NA | 123/46 | 2.7 (2.2, 3.2) | 1.2 (0.9, 1.6) | 0.000 |
| 7.2.2 Non-germ cell | NA | NA | NA | NA | 120/43 | 2.8 (2.3, 3.3) | 1.2 (0.9, 1.6) | 0.000 |
| 7.2.2.1 Carcinoma | NA | NA | NA | NA | 119/41 | 2.9 (2.4, 3.5) | 1.2 (0.9, 1.6) | 0.000 |
| 7.2.2.1.1 Adenocarcinoma | NA | NA | NA | NA | 119/40 | 3.0 (2.5, 3.6) | 1.2 (0.9, 1.6) | 0.000 |
| 7.2.2.1.1.2 Cystadenocarcinoma | NA | NA | NA | NA | 79/22 | 3.7 (2.9, 4.5) | 0.9 (0.6, 1.2) | 0.000 |
| 7.2.2.1.1.5 Endometrioid | NA | NA | NA | NA | 13/6 | 2.2 (1.2, 3.8) | 0.1 (0.0, 0.3) | 0.015 |
| 7.2.2.1.1.6 Other adenocarcinoma | NA | NA | NA | NA | 16/3 | 5.5 (3.2, 9.0) | 0.2 (0.1, 0.4) | 0.000 |
| **8. Melanoma, malignant** | **132/92** | **1.4 (1.2, 1.7)** | **1.0 (0.5, 1.6)** | **0.000** | **223/257** | **0.9 (0.8, 1.0)** | **-0.5 (-1.0, -0.04)** | **0.033** |
| 8.1 Superficial spreading/low cumulative sun damage melanoma | 93/71 | 1.3 (1.1, 1.6) | 0.5 (0.1, 1.0) | 0.016 | 157/210 | 0.7 (0.6, 0.9) | -0.8 (-1.2, -0.4) | 0.000 |
| 8.2 Nodular melanoma | 10/6 | 1.6 (0.8, 3.0) | 0.1 (0.0, 0.3) | 0.189 | 24/14 | 1.8 (1.1, 2.6) | 0.2 (0.0, 0.3) | 0.013 |
| 8.3 Other malignant | 29/14 | 2.1 (1.4, 2.9) | 0.4 (0.1, 0.7) | 0.001 | 42/34 | 1.3 (0.9, 1.7) | 0.1 (-0.1, 0.4) | 0.176 |
| **9. Carcinomas** | **877/369** | **2.4 (2.2, 2.5)** | **12.4 (11.0, 13.9)** | **0.000** | **2,067/1,652** | **1.3 (1.2, 1.3)** | **6.5 (5.1, 7.9)** | **0.000** |
| 9.1 Thyroid carcinoma | 38/11 | 3.5 (2.5, 4.8) | 0.7 (0.4, 1.0) | 0.000 | 80/53 | 1.5 (1.2, 1.9) | 0.4 (0.2, 0.7) | 0.001 |
| 9.1.3 Papillary | 28/7 | 4.0 (2.7, 5.8) | 0.5 (0.3, 0.8) | 0.000 | 54/39 | 1.4 (1.0, 1.8) | 0.2 (0.0, 0.5) | 0.031 |
| 9.1.5 Papillary with follicular variant | NA | NA | NA | NA | 12/7 | 1.8 (0.9, 3.2) | 0.1 (0.0, 0.2) | 0.076 |
| 9.2 Other carcinoma of head and neck | 103/30 | 3.5 (2.8, 4.2) | 1.8 (1.3, 2.3) | 0.000 | 86/32 | 2.7 (2.1, 3.3) | 0.8 (0.6, 1.2) | 0.000 |
| 9.2.2 Oral cavity, lip, and pharynx | 80/19 | 4.1 (3.3, 5.1) | 1.5 (1.1, 2.0) | 0.000 | 57/21 | 2.7 (2.0, 3.5) | 0.6 (0.3, 0.8) | 0.000 |
| 9.2.2.1 Oral cavity, lip, and pharynx, squamous | 77/19 | 4.1 (3.3, 5.2) | 1.4 (1.0, 1.9) | 0.000 | 54/19 | 2.9 (2.1, 3.7) | 0.6 (0.3, 0.8) | 0.000 |
| 9.2.4 Other carcinoma of head and neck | 14/7 | 2.1 (1.1, 3.5) | 0.2 (0.0, 0.4) | 0.019 | 19/6 | 3.0 (1.8, 4.7) | 0.2 (0.1, 0.4) | 0.000 |
| 9.3 Carcinoma of gastrointestinal tract | 306/143 | 2.1 (1.9, 2.4) | 4.0 (3.2, 4.9) | 0.000 | 330/212 | 1.6 (1.4, 1.7) | 1.8 (1.3, 2.4) | 0.000 |
| 9.3.1 Carcinoma of oesophagus | 34/16 | 2.1 (1.5, 3.0) | 0.4 (0.2, 0.8) | 0.000 | 31/8 | 4.0 (2.7, 5.7) | 0.4 (0.2, 0.6) | 0.000 |
| 9.3.2 Carcinoma of stomach | 45/13 | 3.6 (2.6, 4.8) | 0.8 (0.5, 1.2) | 0.000 | 31/15 | 2.1 (1.4, 3.0) | 0.3 (0.1, 0.5) | 0.000 |
| 9.3.2.2 Stomach, signet ring | 12/2 | 6.9 (3.5, 12.0) | 0.3 (0.1, 0.5) | 0.000 | NA | NA | NA | NA |
| 9.3.2.3 Stomach, other adenocarcinoma | 31/10 | 3.2 (2.1, 4.5) | 0.5 (0.3, 0.8) | 0.000 | 24/11 | 2.2 (1.4, 3.3) | 0.2 (0.1, 0.4) | 0.001 |
| 9.3.3 Carcinoma of small intestine | 13/4 | 3.2 (1.7, 5.4) | 0.2 (0.1, 0.4) | 0.001 | 14/5 | 2.9 (1.6, 4.8) | 0.1 (0.0, 0.3) | 0.001 |
| 9.3.3.2 Small intestine, other | 10/1 | 7.1 (3.4, 13.1) | 0.2 (0.1, 0.4) | 0.000 | NA | NA | NA | NA |
| 9.3.4 Carcinoma of colon | 74/52 | 1.4 (1.1, 1.8) | 0.5 (0.1, 1.0) | 0.006 | 112/97 | 1.1 (0.9, 1.4) | 0.2 (-0.1, 0.6) | 0.158 |
| 9.3.4.2 Colon excluding appendix | 70/48 | 1.5 (1.1, 1.9) | 0.5 (0.2, 1.0) | 0.003 | 105/87 | 1.2 (1.0, 1.5) | 0.3 (0.0, 0.6) | 0.070 |
| 9.3.4.2.2 Colon excluding appendix, adenocarcinoma | 69/47 | 1.5 (1.1, 1.9) | 0.5 (0.2, 1.0) | 0.003 | 103/86 | 1.2 (1.0, 1.5) | 0.3 (0.0, 0.6) | 0.073 |
| 9.3.5 Carcinoma of rectum | 52/37 | 1.4 (1.1, 1.8) | 0.4 (0.0, 0.8) | 0.022 | 69/49 | 1.4 (1.1, 1.8) | 0.3 (0.1, 0.6) | 0.007 |
| 9.3.5.2 Rectum, adenocarcinoma | 49/34 | 1.4 (1.1, 1.9) | 0.4 (0.0, 0.7) | 0.023 | 67/47 | 1.4 (1.1, 1.8) | 0.3 (0.1, 0.6) | 0.006 |
| 9.3.6 Carcinoma of anus | 18/3 | 6.7 (4.0, 10.6) | 0.4 (0.2, 0.6) | 0.000 | 13/6 | 2.3 (1.2, 4.0) | 0.1 (0.0, 0.3) | 0.009 |
| 9.3.6.1 Anus, squamous | 17/3 | 6.4 (3.7, 10.3) | 0.4 (0.2, 0.6) | 0.000 | 13/5 | 2.4 (1.3, 4.1) | 0.1 (0.0, 0.3) | 0.008 |
| 9.3.7 Carcinoma of liver and intrahepatic bile ducts (IBD) | 15/4 | 3.5 (2.0, 5.8) | 0.3 (0.1, 0.5) | 0.000 | NA | NA | NA | NA |
| 9.3.8 Carcinoma of gallbladder and other extrahepatic biliary | 10/3 | 3.5 (1.7, 6.5) | 0.2 (0.0, 0.4) | 0.001 | NA | NA | NA | NA |
| 9.3.9 Carcinoma of pancreas | 42/11 | 3.8 (2.7, 5.1) | 0.8 (0.5, 1.1) | 0.000 | 46/21 | 2.2 (1.6, 3.0) | 0.4 (0.2, 0.6) | 0.000 |
| 9.3.9.1 Pancreas, neuroendocrine | NA | NA | NA | NA | 13/4 | 3.3 (1.8, 5.7) | 0.1 (0.0, 0.3) | 0.000 |
| 9.3.9.1.1 NET | NA | NA | NA | NA | 13/3 | 4.4 (2.3, 7.5) | 0.2 (0.1, 0.3) | 0.000 |
| 9.3.9.2 Pancreas, adenocarcinoma | 35/9 | 3.8 (2.7, 5.3) | 0.6 (0.4, 1.0) | 0.000 | 31/16 | 1.9 (1.3, 2.7) | 0.2 (0.1, 0.4) | 0.002 |
| 9.4 Carcinoma of lung, bronchus, and trachea | 141/53 | 2.7 (2.2, 3.2) | 2.2 (1.6, 2.8) | 0.000 | 337/126 | 2.7 (2.4, 3.0) | 3.3 (2.8, 3.9) | 0.000 |
| 9.4.1 Small cell carcinoma, NEC | 19/6 | 3.4 (2.1, 5.3) | 0.3 (0.1, 0.6) | 0.000 | 40/16 | 2.5 (1.8, 3.3) | 0.4 (0.2, 0.6) | 0.000 |
| 9.4.2 Non-small cell carcinoma | 122/47 | 2.6 (2.1, 3.1) | 1.8 (1.3, 2.4) | 0.000 | 297/110 | 2.7 (2.4, 3.0) | 2.9 (2.4, 3.5) | 0.000 |
| 9.4.2.1 Non-small cell, adenocarcinoma | 65/30 | 2.2 (1.7, 2.8) | 0.9 (0.5, 1.3) | 0.000 | 195/80 | 2.4 (2.1, 2.8) | 1.8 (1.4, 2.3) | 0.000 |
| 9.4.2.2 Non-small cell, neuroendocrine | NA | NA | NA | NA | 12/7 | 1.6 (0.8, 2.8) | 0.1 (0.0, 0.2) | 0.154 |
| 9.4.2.3 Non-small cell, other | 55/15 | 3.7 (2.8, 4.9) | 1.0 (0.7, 1.4) | 0.000 | 90/23 | 3.9 (3.2, 4.8) | 1.1 (0.8, 1.4) | 0.000 |
| 9.5 Carcinoma of skin (if collected) | 95/33 | 2.8 (2.3, 3.5) | 1.5 (1.1, 2.0) | 0.000 | 116/73 | 1.6 (1.3, 1.9) | 0.7 (0.4, 1.0) | 0.000 |
| 9.6 Carcinoma of breast | NA | NA | NA | NA | 756/940 | 0.8 (0.7, 0.9) | -2.9 (-3.7, -2.0) | 0.000 |
| 9.6.1 Breast, infiltrating duct | NA | NA | NA | NA | 633/792 | 0.8 (0.7, 0.9) | -2.5 (-3.3, -1.7) | 0.000 |
| 9.6.2 Breast, adenocarcinoma | NA | NA | NA | NA | 48/33 | 1.5 (1.1, 1.9) | 0.2 (0.0, 0.5) | 0.015 |
| 9.6.3 Breast, lobular | NA | NA | NA | NA | 50/91 | 0.5 (0.4, 0.7) | -0.6 (-0.9, -0.4) | 0.000 |
| 9.7 Carcinoma of genital sites excluding ovary and testis | 70/47 | 1.5 (1.2, 1.9) | 0.6 (0.2, 1.0) | 0.002 | 227/161 | 1.4 (1.2, 1.6) | 1.0 (0.6, 1.5) | 0.000 |
| 9.7.1 Carcinoma of uterine cervix | NA | NA | NA | NA | 84/104 | 0.8 (0.6, 1.0) | -0.3 (-0.6, 0.0) | 0.049 |
| 9.7.1.1 Cervix, squamous | NA | NA | NA | NA | 57/76 | 0.8 (0.6, 1.0) | -0.3 (-0.5, 0.0) | 0.029 |
| 9.7.1.3 Cervix, adenocarcinoma | NA | NA | NA | NA | 24/23 | 1.1 (0.7, 1.6) | 0.0 (-0.1, 0.2) | 0.835 |
| 9.7.2 Corpus uteri | NA | NA | NA | NA | 98/41 | 2.4 (1.9, 2.9) | 0.9 (0.6, 1.2) | 0.000 |
| 9.7.2.1 Corpus uteri, adenocarcinoma | NA | NA | NA | NA | 92/39 | 2.4 (1.9, 2.9) | 0.8 (0.6, 1.2) | 0.000 |
| 9.7.2.1.1 Corpus uteri, endometrioid | NA | NA | NA | NA | 78/33 | 2.3 (1.8, 2.9) | 0.7 (0.4, 1.0) | 0.000 |
| 9.7.2.1.2 Corpus uteri, other adenocarcinoma | NA | NA | NA | NA | 14/5 | 2.6 (1.4, 4.4) | 0.1 (0.0, 0.3) | 0.002 |
| 9.7.3 Carcinoma of vulva and vagina | NA | NA | NA | NA | 35/15 | 2.4 (1.7, 3.3) | 0.3 (0.2, 0.5) | 0.000 |
| 9.7.5 Carcinoma of prostate | 64/44 | 1.5 (1.1, 1.9) | 0.5 (0.1, 0.9) | 0.006 | NA | NA | NA | NA |
| 9.7.6 Other genital | NA | NA | NA | NA | 10/2 | 6.1 (2.9, 11.2) | 0.1 (0.0, 0.3) | 0.000 |
| 9.8 Carcinoma of urinary tract | 93/45 | 2.1 (1.7, 2.5) | 1.2 (0.7, 1.7) | 0.000 | 77/40 | 1.9 (1.5, 2.4) | 0.6 (0.3, 0.9) | 0.000 |
| 9.8.1 Carcinoma of kidney | 56/29 | 1.9 (1.5, 2.5) | 0.7 (0.3, 1.1) | 0.000 | 39/26 | 1.5 (1.1, 2.0) | 0.2 (0.0, 0.4) | 0.022 |
| 9.8.1.1 Kidney, adenocarcinoma | 56/29 | 1.9 (1.5, 2.5) | 0.7 (0.3, 1.1) | 0.000 | 38/26 | 1.5 (1.0, 2.0) | 0.2 (0.0, 0.4) | 0.033 |
| 9.8.1.1.1 Kidney, renal cell | 44/24 | 1.8 (1.3, 2.5) | 0.5 (0.2, 0.9) | 0.000 | 36/22 | 1.6 (1.1, 2.2) | 0.2 (0.0, 0.4) | 0.010 |
| 9.8.1.1.2 Kidney, other adenocarcinoma | 12/5 | 2.4 (1.3, 4.3) | 0.2 (0.0, 0.4) | 0.010 | NA | NA | NA | NA |
| 9.8.2 Carcinoma of bladder | 30/14 | 2.2 (1.5, 3.1) | 0.4 (0.2, 0.7) | 0.000 | 29/12 | 2.4 (1.6, 3.5) | 0.3 (0.1, 0.5) | 0.000 |
| 9.8.2.1 Urinary bladder, transitional cell carcinoma | 27/13 | 2.2 (1.4, 3.1) | 0.4 (0.1, 0.7) | 0.001 | 23/10 | 2.3 (1.4, 3.4) | 0.2 (0.1, 0.4) | 0.001 |
| 9.9 Other invasive carcinomas | 25/5 | 5.2 (3.3, 7.6) | 0.5 (0.3, 0.8) | 0.000 | 58/14 | 4.1 (3.1, 5.3) | 0.7 (0.5, 1.0) | 0.000 |
| 9.9.2 Unknown primary | 21/4 | 5.5 (3.4, 8.5) | 0.4 (0.2, 0.7) | 0.000 | 46/9 | 5.1 (3.8, 6.9) | 0.6 (0.4, 0.8) | 0.000 |
| 9.9.4 Carcinoma of other and ill-defined sites | NA | NA | NA | NA | 10/3 | 3.8 (1.8, 7.0) | 0.1 (0.0, 0.2) | 0.001 |
| **10. Miscellaneous specified neoplasms** | **NA** | **NA** | **NA** | **NA** | **11/6** | **2.0 (1.0, 3.5)** | **0.1 (0.0, 0.2)** | **0.056** |
| 10.2 Other specified tumours | NA | NA | NA | NA | 10/5 | 1.9 (0.9, 3.5) | 0.1 (0.0, 0.2) | 0.079 |
| 10.2.2 Other specified neoplasms | NA | NA | NA | NA | 10/4 | 2.3 (1.1, 4.2) | 0.1 (0.0, 0.2) | 0.028 |
| **11. Unspecified malignant neoplasms except CNS** | **21/10** | **2.2 (1.4, 3.4)** | **0.3 (0.1, 0.6)** | **0.002** | **20/17** | **1.1 (0.7, 1.8)** | **0.0 (-0.1, 0.2)** | **0.595** |

Abbreviation: CNS=Central Nervous System, NET=Neuroendocrine tumour, NA=Not Applicable.

**Supplementary Table S3B.** Standardised incidence ratios (SIRs) and absolute excess risks (AERs) of second primary cancer diagnosis after any first primary cancer among six-month adolescent and young adult (AYA, aged 15-39 years) cancer survivors compared to the general population in the Netherlands. Second primary cancer types are grouped according to the AYA-specific classification scheme developed by Barr and colleagues (2020). Cancer combinations with less than n=10 observed second cancers were excluded from the analyses. This Table presents outcomes from the sensitivity analysis.

| **Second primary cancers** | **Cancer risk after any first cancer** | | | | | | | |
| --- | --- | --- | --- | --- | --- | --- | --- | --- |
|  | **Males** | | | | **Females** | | | |
|  | **Obs/exp** | **SIR (95%CI)** | **AER per 10,000 person-years (95%CI)** | **P-value** | **Obs/exp** | **SIR (95%CI)** | **AER per 10,000 person-years (95%CI)** | **P-value** |
| **Total** | 2,018/702 | 2.9 (2.7, 3.0) | 32.5 (30.4, 34.8) | 0.000 | 4,549/2,093 | 2.2 (2.1, 2.2) | 39.5 (37.3, 41.6) | 0.000 |
| **Cancer types** |  |  |  |  |  |  |  |  |
| **1. Leukaemia’s and related disorders** | 110/38 | 2.9 (2.4, 3.5) | 1.8 (1.3, 2.3) | 0.000 | 100/46 | 2.2 (1.8, 2.6) | 0.9 (0.6, 1.2) | 0.000 |
| 1.1 Acute lymphoblastic leukaemia | 14/3 | 4.5 (2.4, 7.5) | 0.3 (0.1, 0.5) | 0.000 | NA | NA | NA | NA |
| 1.2 Acute myeloid leukaemia | 42/8 | 5.2 (3.8, 7.1) | 0.8 (0.6, 1.2) | 0.000 | 42/11 | 3.7 (2.6, 4.9) | 0.5 (0.3, 0.7) | 0.000 |
| 1.2.2 Other acute myeloid leukaemia | 40/7 | 5.7 (4.1, 7.8) | 0.8 (0.5, 1.2) | 0.000 | 41/10 | 4.0 (2.9, 5.5) | 0.5 (0.3, 0.7) | 0.000 |
| 1.3 Chronic myeloid leukaemia | 12/4 | 2.7 (1.4, 4.8) | 0.2 (0.0, 0.4) | 0.004 | 11/5 | 2.2 (1.1, 3.9) | 0.1 (0.0, 0.2) | 0.029 |
| 1.4 Chronic lymphocytic leukaemia | 10/6 | 1.7 (0.8, 3.2) | 0.1 (0.0, 0.3) | 0.139 | 10/5 | 1.8 (0.9, 3.4) | 0.1 (0.0, 0.2) | 0.103 |
| 1.8 Myelodysplastic syndrome (MDS) | 17/3 | 6.8 (4.0, 10.9) | 0.4 (0.2, 0.6) | 0.000 | 15/3 | 4.3 (2.4, 7.1) | 0.2 (0.1, 0.3) | 0.000 |
| **2. Lymphomas** | 141/68 | 2.1 (1.7, 2.5) | 1.8 (1.3, 2.4) | 0.000 | 101/73 | 1.4 (1.1, 1.7) | 0.4 (0.1, 0.8) | 0.002 |
| 2.1 Non-Hodgkin lymphomas | 94/39 | 2.4 (1.9, 2.9) | 1.4 (0.9, 1.9) | 0.000 | 86/46 | 1.9 (1.5, 2.3) | 0.6 (0.4, 1.0) | 0.000 |
| 2.1.3 Diffuse large B-cell (DLBCL) | 56/17 | 3.3 (2.5, 4.3) | 1.0 (0.6, 1.4) | 0.000 | 35/17 | 2.0 (1.4, 2.8) | 0.3 (0.1, 0.5) | 0.000 |
| 2.1.5 Anaplastic T-cell and null-cell excluding NK/T-cell | NA | NA | NA | NA | 10/6 | 1.7 (0.8, 3.2) | 0.1 (0.0, 0.2) | 0.136 |
| 2.1.6 Follicular | NA | NA | NA | NA | 22/11 | 2.0 (1.3, 3.0) | 0.2 (0.0, 0.4) | 0.005 |
| 2.1.8 MALT (Mucosa-associated lymphoid tissue) | NA | NA | NA | NA | 12/6 | 2.2 (1.1, 3.8) | 0.1 (0.0, 0.2) | 0.022 |
| 2.1.9 Other non-Hodgkin lymphoma NOS | 13/3 | 4.5 (2.4, 7.6) | 0.2 (0.1, 0.5) | 0.000 | NA | NA | NA | NA |
| 2.2 Hodgkin lymphoma | 31/16 | 1.9 (1.3, 2.7) | 0.4 (0.1, 0.7) | 0.002 | NA | NA | NA | NA |
| 2.2.2 Hodgkin classic, other | 27/14 | 1.9 (1.3, 2.8) | 0.3 (0.1, 0.6) | 0.003 | NA | NA | NA | NA |
| **3. CNS and other intracranial and intraspinal neoplasms** | 58/26 | 2.2 (1.7, 2.8) | 0.8 (0.4, 1.2) | 0.000 | 53/26 | 2.0 (1.5, 2.6) | 0.4 (0.2, 0.7) | 0.000 |
| 3.1 Astroglial and related neoplasms | 53/24 | 2.3 (1.7, 2.9) | 0.7 (0.4, 1.1) | 0.000 | 46/24 | 1.9 (1.4, 2.6) | 0.4 (0.2, 0.6) | 0.000 |
| 3.1.1 Oligodendriogliomas | NA | NA | NA | NA | 12/5 | 2.7 (1.4, 4.6) | 0.1 (0.0, 0.3) | 0.005 |
| 3.1.1.2 Oligodendrioglioma, invasive | NA | NA | NA | NA | 12/5 | 2.7 (1.4, 4.6) | 0.1 (0.0, 0.3) | 0.005 |
| 3.1.2 Glioblastomas/gliofibromas | 25/12 | 2.0 (1.3, 3.0) | 0.3 (0.1, 0.6) | 0.002 | 17/11 | 1.6 (0.9, 2.5) | 0.1 (0.0, 0.3) | 0.095 |
| 3.1.2.2 Glioblastoma, invasive | 25/12 | 2.0 (1.3, 3.0) | 0.3 (0.1, 0.6) | 0.002 | 17/11 | 1.6 (0.9, 2.5) | 0.1 (0.0, 0.3) | 0.095 |
| 3.1.4 Other astrocytoma/astroglial neoplasms | 20/6 | 3.4 (2.1, 5.2) | 0.3 (0.2, 0.6) | 0.000 | 15/7 | 2.1 (1.2, 3.4) | 0.1 (0.0, 0.3) | 0.016 |
| 3.1.4.3 Other astrocytoma/astroglial, invasive | 20/6 | 3.4 (2.1, 5.2) | 0.3 (0.2, 0.6) | 0.000 | 15/7 | 2.1 (1.2, 3.4) | 0.1 (0.0, 0.3) | 0.016 |
| **4. Sarcomas** | 60/16 | 3.7 (2.8, 4.8) | 1.1 (0.7, 1.5) | 0.000 | 110/27 | 4.0 (3.3, 4.9) | 1.3 (1.0, 1.7) | 0.000 |
| 4.2 Chondrosarcoma | NA | NA | NA | NA | 26/2 | 10.5 (6.9, 15.5) | 0.4 (0.2, 0.6) | 0.000 |
| 4.4 Fibromatous neoplasms | NA | NA | NA | NA | 15/6 | 2.7 (1.5, 4.4) | 0.2 (0.0, 0.3) | 0.002 |
| 4.7 Leiomyosarcoma | NA | NA | NA | NA | 10/4 | 2.3 (1.1, 4.3) | 0.1 (0.0, 0.2) | 0.027 |
| 4.15 Other soft tissue sarcomas | 12/1 | 13.1 (6.8, 22.9) | 0.3 (0.1, 0.5) | 0.000 | 13/2 | 5.9 (3.1, 10.1) | 0.2 (0.1, 0.3) | 0.000 |
| **5. Blood and lymphatic vessel tumours** | 16/3 | 4.7 (2.7, 7.7) | 0.3 (0.1, 0.6) | 0.000 | 11/2 | 5.1 (2.5, 9.1) | 0.1 (0.1, 0.3) | 0.000 |
| 5.2 Malignant blood and lymphatic vessel tumours, all sites | 16/3 | 4.7 (2.7, 7.7) | 0.3 (0.1, 0.6) | 0.000 | 11/2 | 5.1 (2.5, 9.1) | 0.1 (0.1, 0.3) | 0.000 |
| 5.2.2 Other | 10/1 | 12.5 (6.0, 22.9) | 0.2 (0.1, 0.4) | 0.000 | 11/1 | 7.4 (3.7, 13.2) | 0.2 (0.1, 0.3) | 0.000 |
| **6. Nerve sheath tumours** | NA | NA | NA | NA | 17/1 | 13.8 (8.1, 22.2) | 0.3 (0.1, 0.4) | 0.000 |
| 6.2 Malignant | NA | NA | NA | NA | 17/1 | 13.8 (8.1, 22.2) | 0.3 (0.1, 0.4) | 0.000 |
| 6.2.1 MPNST (Malignant peripheral nerve sheath tumour) | NA | NA | NA | NA | 17/1 | 14.2 (8.3, 22.7) | 0.3 (0.1, 0.4) | 0.000 |
| 6.2.1.2 Peripheral | NA | NA | NA | NA | 17/1 | 14.3 (8.3, 22.9) | 0.3 (0.1, 0.4) | 0.000 |
| **7. Gonadal and related tumours** | 332/85 | 3.9 (3.5, 4.3) | 6.1 (5.2, 7.0) | 0.000 | 138/46 | 3.0 (2.5, 3.6) | 1.5 (1.1, 1.9) | 0.000 |
| 7.1 Testis | 329/83 | 3.9 (3.5, 4.4) | 6.1 (5.2, 7.0) | 0.000 | NA | NA | NA | NA |
| 7.1.1 Germ cell and trophoblastic | 329/83 | 4.0 (3.5, 4.4) | 6.1 (5.2, 7.0) | 0.000 | NA | NA | NA | NA |
| 7.1.1.1 Seminoma | 218/53 | 4.1 (3.6, 4.7) | 4.1 (3.4, 4.8) | 0.000 | NA | NA | NA | NA |
| 7.1.1.2 Embryonal carcinoma | 29/7 | 4.3 (2.9, 6.2) | 0.6 (0.3, 0.9) | 0.000 | NA | NA | NA | NA |
| 7.1.1.4 Teratoma | 13/3 | 4.0 (2.1, 6.8) | 0.2 (0.1, 0.5) | 0.000 | NA | NA | NA | NA |
| 7.1.1.5 Mixed germ cell | 63/18 | 3.6 (2.8, 4.6) | 1.1 (0.8, 1.6) | 0.000 | NA | NA | NA | NA |
| 7.2 Ovary | NA | NA | NA | NA | 138/44 | 3.1 (2.6, 3.7) | 1.5 (1.2, 1.9) | 0.000 |
| 7.2.2 Non-germ cell | NA | NA | NA | NA | 133/42 | 3.2 (2.7, 3.8) | 1.5 (1.1, 1.9) | 0.000 |
| 7.2.2.1 Carcinoma | NA | NA | NA | NA | 132/40 | 3.3 (2.8, 4.0) | 1.5 (1.1, 1.9) | 0.000 |
| 7.2.2.1.1 Adenocarcinoma | NA | NA | NA | NA | 132/39 | 3.4 (2.9, 4.0) | 1.5 (1.2, 1.9) | 0.000 |
| 7.2.2.1.1.2 Cystadenocarcinoma | NA | NA | NA | NA | 88/21 | 4.2 (3.4, 5.2) | 1.1 (0.8, 1.4) | 0.000 |
| 7.2.2.1.1.4 Mucinous adenocarcinoma | NA | NA | NA | NA | 11/5 | 2.3 (1.2, 4.1) | 0.1 (0.0, 0.2) | 0.020 |
| 7.2.2.1.1.5 Endometrioid | NA | NA | NA | NA | 14/6 | 2.5 (1.3, 4.1) | 0.1 (0.0, 0.3) | 0.005 |
| 7.2.2.1.1.6 Other adenocarcinoma | NA | NA | NA | NA | 16/3 | 5.7 (3.2, 9.2) | 0.2 (0.1, 0.4) | 0.000 |
| **8. Melanoma, malignant** | 334/90 | 3.7 (3.3, 4.1) | 6.0 (5.2, 7.0) | 0.000 | 624/250 | 2.5 (2.3, 2.7) | 6.0 (5.2, 6.8) | 0.000 |
| 8.1 Superficial spreading/low cumulative sun damage melanoma | 253/70 | 3.6 (3.2, 4.1) | 4.5 (3.8, 5.3) | 0.000 | 482/204 | 2.4 (2.2, 2.6) | 4.5 (3.8, 5.2) | 0.000 |
| 8.2 Nodular melanoma | 31/6 | 5.1 (3.5, 7.2) | 0.6 (0.4, 0.9) | 0.000 | 42/13 | 3.2 (2.3, 4.3) | 0.5 (0.3, 0.7) | 0.000 |
| 8.3 Other malignant | 50/14 | 3.6 (2.7, 4.7) | 0.9 (0.6, 1.3) | 0.000 | 100/33 | 3.1 (2.5, 3.7) | 1.1 (0.8, 1.4) | 0.000 |
| **9. Carcinomas** | 936/362 | 2.6 (2.4, 2.8) | 14.2 (12.7, 15.7) | 0.000 | 3,360/1,599 | 2.1 (2.0, 2.2) | 28.3 (26.5, 30.1) | 0.000 |
| 9.1 Thyroid carcinoma | 39/11 | 3.6 (2.6, 5.0) | 0.7 (0.4, 1.1) | 0.000 | 76/52 | 1.5 (1.2, 1.8) | 0.4 (0.1, 0.7) | 0.002 |
| 9.1.3 Papillary | 29/7 | 4.2 (2.8, 6.0) | 0.5 (0.3, 0.9) | 0.000 | 51/38 | 1.3 (1.0, 1.8) | 0.2 (0.0, 0.5) | 0.056 |
| 9.1.5 Papillary with follicular variant | NA | NA | NA | NA | 12/6 | 1.9 (1.0, 3.2) | 0.1 (0.0, 0.2) | 0.067 |
| 9.2 Other carcinoma of head and neck | 117/29 | 4.0 (3.3, 4.8) | 2.2 (1.7, 2.7) | 0.000 | 100/31 | 3.2 (2.6, 3.9) | 1.1 (0.8, 1.5) | 0.000 |
| 9.2.2 Oral cavity, lip, and pharynx | 91/19 | 4.8 (3.8, 5.9) | 1.8 (1.3, 2.3) | 0.000 | 67/21 | 3.3 (2.5, 4.1) | 0.7 (0.5, 1.0) | 0.000 |
| 9.2.2.1 Oral cavity, lip, and pharynx, squamous | 88/18 | 4.8 (3.8, 5.9) | 1.7 (1.3, 2.2) | 0.000 | 65/18 | 3.6 (2.7, 4.5) | 0.8 (0.5, 1.0) | 0.000 |
| 9.2.4 Other carcinoma of head and neck | 17/7 | 2.6 (1.5, 4.1) | 0.3 (0.1, 0.5) | 0.001 | 20/6 | 3.3 (2.0, 5.1) | 0.2 (0.1, 0.4) | 0.000 |
| 9.3 Carcinoma of gastrointestinal tract | 331/141 | 2.4 (2.1, 2.6) | 4.7 (3.8, 5.6) | 0.000 | 321/205 | 1.6 (1.4, 1.8) | 1.9 (1.3, 2.5) | 0.000 |
| 9.3.1 Carcinoma of oesophagus | 33/16 | 2.1 (1.5, 3.0) | 0.4 (0.2, 0.8) | 0.000 | 27/7 | 3.6 (2.4, 5.3) | 0.3 (0.2, 0.5) | 0.000 |
| 9.3.2 Carcinoma of stomach | 43/12 | 3.5 (2.5, 4.7) | 0.8 (0.5, 1.1) | 0.000 | 29/14 | 2.0 (1.4, 2.9) | 0.2 (0.1, 0.4) | 0.001 |
| 9.3.2.2 Stomach, signet ring | 11/2 | 6.4 (3.2, 11.4) | 0.2 (0.1, 0.4) | 0.000 | NA | NA | NA | NA |
| 9.3.2.3 Stomach, other adenocarcinoma | 30/10 | 3.1 (2.1, 4.4) | 0.5 (0.3, 0.8) | 0.000 | 22/10 | 2.1 (1.3, 3.2) | 0.2 (0.1, 0.4) | 0.002 |
| 9.3.3 Carcinoma of small intestine | 13/4 | 3.2 (1.7, 5.5) | 0.2 (0.1, 0.5) | 0.001 | 13/5 | 2.8 (1.5, 4.7) | 0.1 (0.0, 0.3) | 0.002 |
| 9.3.3.2 Small intestine, other | 10/1 | 7.2 (3.5, 13.3) | 0.2 (0.1, 0.4) | 0.000 | NA | NA | NA | NA |
| 9.3.4 Carcinoma of colon | 102/51 | 2.0 (1.6, 2.4) | 1.3 (0.8, 1.8) | 0.000 | 120/94 | 1.3 (1.1, 1.5) | 0.4 (0.1, 0.8) | 0.010 |
| 9.3.4.2 Colon excluding appendix | 98/47 | 2.1 (1.7, 2.5) | 1.3 (0.8, 1.8) | 0.000 | 113/84 | 1.3 (1.1, 1.6) | 0.5 (0.1, 0.8) | 0.003 |
| 9.3.4.2.2 Colon excluding appendix, adenocarcinoma | 97/46 | 2.1 (1.7, 2.6) | 1.3 (0.8, 1.8) | 0.000 | 111/82 | 1.4 (1.1, 1.6) | 0.5 (0.1, 0.8) | 0.003 |
| 9.3.5 Carcinoma of rectum | 52/36 | 1.4 (1.1, 1.9) | 0.4 (0.1, 0.8) | 0.016 | 62/47 | 1.3 (1.0, 1.7) | 0.2 (0.0, 0.5) | 0.039 |
| 9.3.5.2 Rectum, adenocarcinoma | 49/34 | 1.4 (1.1, 1.9) | 0.4 (0.1, 0.8) | 0.017 | 60/45 | 1.3 (1.0, 1.7) | 0.2 (0.0, 0.5) | 0.035 |
| 9.3.6 Carcinoma of anus | 18/3 | 6.8 (4.0, 10.8) | 0.4 (0.2, 0.6) | 0.000 | 13/5 | 2.4 (1.3, 4.2) | 0.1 (0.0, 0.3) | 0.007 |
| 9.3.6.1 Anus, squamous | 17/3 | 6.5 (3.8, 10.5) | 0.4 (0.2, 0.6) | 0.000 | 13/5 | 2.5 (1.3, 4.2) | 0.1 (0.0, 0.3) | 0.006 |
| 9.3.7 Carcinoma of liver and intrahepatic bile ducts (IBD) | 15/4 | 3.6 (2.0, 5.9) | 0.3 (0.1, 0.5) | 0.000 | NA | NA | NA | NA |
| 9.3.8 Carcinoma of gallbladder and other extrahepatic biliary | 10/3 | 3.6 (1.7, 6.6) | 0.2 (0.0, 0.4) | 0.001 | NA | NA | NA | NA |
| 9.3.9 Carcinoma of pancreas | 42/11 | 3.8 (2.8, 5.2) | 0.8 (0.5, 1.1) | 0.000 | 43/20 | 2.2 (1.6, 2.9) | 0.4 (0.2, 0.6) | 0.000 |
| 9.3.9.1 Pancreas, neuroendocrine | NA | NA | NA | NA | 12/4 | 3.2 (1.7, 5.6) | 0.1 (0.0, 0.3) | 0.001 |
| 9.3.9.1.1 NET | NA | NA | NA | NA | 12/3 | 4.2 (2.2, 7.4) | 0.1 (0.1, 0.3) | 0.000 |
| 9.3.9.2 Pancreas, adenocarcinoma | 35/9 | 3.9 (2.7, 5.4) | 0.6 (0.4, 1.0) | 0.000 | 29/16 | 1.8 (1.2, 2.6) | 0.2 (0.1, 0.4) | 0.004 |
| 9.4 Carcinoma of lung, bronchus, and trachea | 143/52 | 2.8 (2.3, 3.2) | 2.3 (1.7, 2.9) | 0.000 | 333/122 | 2.7 (2.5, 3.0) | 3.4 (2.8, 4.0) | 0.000 |
| 9.4.1 Small cell carcinoma, NEC | 22/5 | 4.0 (2.5, 6.1) | 0.4 (0.2, 0.7) | 0.000 | 38/16 | 2.4 (1.7, 3.3) | 0.4 (0.2, 0.6) | 0.000 |
| 9.4.2 Non-small cell carcinoma | 121/46 | 2.6 (2.2, 3.1) | 1.8 (1.3, 2.4) | 0.000 | 295/106 | 2.8 (2.5, 3.1) | 3.0 (2.5, 3.6) | 0.000 |
| 9.4.2.1 Non-small cell, adenocarcinoma | 62/29 | 2.1 (1.6, 2.7) | 0.8 (0.5, 1.2) | 0.000 | 192/77 | 2.5 (2.2, 2.9) | 1.9 (1.4, 2.3) | 0.000 |
| 9.4.2.2 Non-small cell, neuroendocrine | NA | NA | NA | NA | 13/7 | 1.8 (1.0, 3.1) | 0.1 (0.0, 0.2) | 0.066 |
| 9.4.2.3 Non-small cell, other | 56/15 | 3.9 (2.9, 5.0) | 1.0 (0.7, 1.4) | 0.000 | 90/22 | 4.1 (3.3, 5.0) | 1.1 (0.8, 1.4) | 0.000 |
| 9.5 Carcinoma of skin (if collected) | 90/33 | 2.8 (2.2, 3.4) | 1.4 (1.0, 1.9) | 0.000 | 113/70 | 1.6 (1.3, 1.9) | 0.7 (0.4, 1.1) | 0.000 |
| 9.6 Carcinoma of breast | NA | NA | NA | NA | 2,047/911 | 2.2 (2.1, 2.3) | 18.2 (16.8, 19.7) | 0.000 |
| 9.6.1 Breast, infiltrating duct | NA | NA | NA | NA | 1,689/768 | 2.2 (2.1, 2.3) | 14.8 (13.5, 16.1) | 0.000 |
| 9.6.2 Breast, adenocarcinoma | NA | NA | NA | NA | 147/32 | 4.6 (3.9, 5.4) | 1.8 (1.5, 2.3) | 0.000 |
| 9.6.3 Breast, lobular | NA | NA | NA | NA | 147/88 | 1.7 (1.4, 2.0) | 0.9 (0.6, 1.4) | 0.000 |
| 9.6.5 Breast, medullary | NA | NA | NA | NA | 27/8 | 3.4 (2.2, 5.0) | 0.3 (0.2, 0.5) | 0.000 |
| 9.6.8 Breast, metaplastic | NA | NA | NA | NA | 11/5 | 2.0 (1.0, 3.6) | 0.1 (0.0, 0.2) | 0.047 |
| 9.6.10 Breast, other | NA | NA | NA | NA | 17/2 | 11.3 (6.6, 18.1) | 0.2 (0.1, 0.4) | 0.000 |
| 9.7 Carcinoma of genital sites excluding ovary and testis | 69/46 | 1.5 (1.2, 1.9) | 0.6 (0.2, 1.0) | 0.002 | 228/157 | 1.5 (1.3, 1.7) | 1.1 (0.7, 1.7) | 0.000 |
| 9.7.1 Carcinoma of uterine cervix | NA | NA | NA | NA | 86/102 | 0.8 (0.7, 1.0) | -0.2 (-0.5, 0.1) | 0.131 |
| 9.7.1.1 Cervix, squamous | NA | NA | NA | NA | 59/74 | 0.8 (0.6, 1.0) | -0.2 (-0.5, 0.0) | 0.085 |
| 9.7.1.3 Cervix, adenocarcinoma | NA | NA | NA | NA | 24/22 | 1.1 (0.7, 1.6) | 0.0 (-0.1, 0.2) | 0.742 |
| 9.7.2 Corpus uteri | NA | NA | NA | NA | 94/39 | 2.4 (1.9, 2.9) | 0.9 (0.6, 1.2) | 0.000 |
| 9.7.2.1 Corpus uteri, adenocarcinoma | NA | NA | NA | NA | 88/37 | 2.4 (1.9, 2.9) | 0.8 (0.5, 1.1) | 0.000 |
| 9.7.2.1.1 Corpus uteri, endometrioid | NA | NA | NA | NA | 75/32 | 2.3 (1.8, 2.9) | 0.7 (0.4, 1.0) | 0.000 |
| 9.7.2.1.2 Corpus uteri, other adenocarcinoma | NA | NA | NA | NA | 13/5 | 2.6 (1.4, 4.4) | 0.1 (0.0, 0.3) | 0.005 |
| 9.7.3 Carcinoma of vulva and vagina | NA | NA | NA | NA | 39/14 | 2.7 (2.0, 3.8) | 0.4 (0.2, 0.6) | 0.000 |
| 9.7.5 Carcinoma of prostate | 63/43 | 1.5 (1.1, 1.9) | 0.5 (0.1, 0.9) | 0.005 | NA | NA | NA | NA |
| 9.8 Carcinoma of urinary tract | 117/44 | 2.6 (2.2, 3.2) | 1.8 (1.3, 2.4) | 0.000 | 85/38 | 2.2 (1.8, 2.7) | 0.7 (0.5, 1.1) | 0.000 |
| 9.8.1 Carcinoma of kidney | 77/29 | 2.7 (2.1, 3.4) | 1.2 (0.8, 1.7) | 0.000 | 45/25 | 1.8 (1.3, 2.4) | 0.3 (0.1, 0.6) | 0.000 |
| 9.8.1.1 Kidney, adenocarcinoma | 77/28 | 2.7 (2.1, 3.4) | 1.2 (0.8, 1.7) | 0.000 | 44/25 | 1.7 (1.3, 2.3) | 0.3 (0.1, 0.5) | 0.001 |
| 9.8.1.1.1 Kidney, renal cell | 63/24 | 2.7 (2.0, 3.4) | 1.0 (0.6, 1.4) | 0.000 | 42/22 | 1.9 (1.4, 2.6) | 0.3 (0.1, 0.6) | 0.000 |
| 9.8.1.1.2 Kidney, other adenocarcinoma | 14/5 | 2.9 (1.6, 4.9) | 0.2 (0.1, 0.5) | 0.001 | NA | NA | NA | NA |
| 9.8.2 Carcinoma of bladder | 31/13 | 2.3 (1.6, 3.3) | 0.4 (0.2, 0.8) | 0.000 | 31/11 | 2.7 (1.8, 3.8) | 0.3 (0.2, 0.5) | 0.000 |
| 9.8.2.1 Urinary bladder, transitional cell carcinoma | 27/12 | 2.2 (1.4, 3.2) | 0.4 (0.1, 0.7) | 0.000 | 25/10 | 2.5 (1.6, 3.8) | 0.2 (0.1, 0.4) | 0.000 |
| 9.9 Other invasive carcinomas | 24/5 | 5.0 (3.2, 7.4) | 0.5 (0.3, 0.8) | 0.000 | 57/14 | 4.1 (3.1, 5.4) | 0.7 (0.5, 1.0) | 0.000 |
| 9.9.2 Unknown primary | 20/4 | 5.3 (3.3, 8.2) | 0.4 (0.2, 0.7) | 0.000 | 45/9 | 5.2 (3.8, 6.9) | 0.6 (0.4, 0.8) | 0.000 |
| 9.9.4 Carcinoma of other and ill-defined sites | NA | NA | NA | NA | 10/3 | 3.9 (1.9, 7.3) | 0.1 (0.0, 0.3) | 0.001 |
| **10. Miscellaneous specified neoplasms** | NA | NA | NA | NA | 11/5 | 2.0 (1.0, 3.6) | 0.1 (0.0, 0.2) | 0.045 |
| 10.2 Other specified tumours | NA | NA | NA | NA | 10/5 | 2.0 (1.0, 3.7) | 0.1 (0.0, 0.2) | 0.064 |
| **11. Unspecified malignant neoplasms except CNS** | 19/9 | 2.0 (1.2, 3.2) | 0.2 (0.1, 0.5) | 0.007 | 24/17 | 1.4 (0.9, 2.2) | 0.1 (0.0, 0.3) | 0.103 |

Abbreviation: CNS=Central Nervous System, NET=Neuroendocrine tumour, NA=Not Applicable.

**Supplementary Table S4A.** Standardised incidence ratios (SIRs) and absolute excess risks (AERs) of second primary leukaemia’s after each distinct first primary cancer type among six-month adolescent and young adult (AYA, aged 15-39 years) cancer survivors compared to the general population in the Netherlands. Second primary cancer types are grouped according to the AYA-specific classification scheme developed by Barr and colleagues (2020). First and second cancer combinations with less than n=10 observed second cancers were excluded from the analyses. This Table presents outcomes from the main analysis.

|  | **Second primary cancers** | **1. Leukaemia’s and related disorders** | | | **1.2 Acute myeloid leukaemia** | | | **1.2.2 Other acute myeloid leukaemia** | | |
| --- | --- | --- | --- | --- | --- | --- | --- | --- | --- | --- |
|  | **First primary cancers** | **Obs/exp** | **SIR (95%CI)** | **AER per 10,000 person-years (95%CI)** | **Obs/exp** | **SIR (95%CI)** | **AER per 10,000 person-years (95%CI)** | **Obs/exp** | **SIR (95%CI)** | **AER per 10,000 person-years (95%CI)** |
| **Males** | **2. Lymphomas** | **32/6** | **5.2 (3.6, 7.3)** | **3.7 (2.2, 5.6)** | **15/1** | **11.3 (6.4, 18.7)** | **1.9 (1.0, 3.3)** | **14/11** | **12.2 (6.7, 20.4)** | **1.8 (0.9, 3.2)** |
|  | 2.1 Non-Hodgkin lymphomas | 12/3 | 4.6 (2.4, 8.1) | 3.4 (1.3, 6.7) | NA | NA | NA | NA | NA | NA |
|  | 2.2 Hodgkin lymphoma | 20/3 | 6.3 (3.9, 9.8) | 4.3 (2.3, 7.1) | NA | NA | NA | NA | NA | NA |
|  | 2.2.2 Hodgkin classic, other | 17/3 | 5.8 (3.4, 9.2) | 3.9 (1.9, 6.7) | NA | NA | NA | NA | NA | NA |
|  | **7. Gonadal and related tumours** | **26/12** | **2.1 (1.4, 3.1)** | **1.0 (0.3, 1.8)** | **11/3** | **4.1 (2.0, 7.3)** | **0.6 (0.2, 1.2)** | **10/2** | **4.3 (2.1, 7.9)** | **0.5 (0.2, 1.1)** |
|  | 7.1 Testis | 25/12 | 2.1 (1.3, 3.0) | 0.9 (0.3, 1.8) | 10/3 | 3.8 (1.8, 7.0) | 0.5 (0.2, 1.2) | NA | NA | NA |
|  | 7.1.1 Germ cell and trophoblastic | 25/12 | 2.1 (1.3, 3.0) | 0.9 (0.3, 1.8) | 10/3 | 3.8 (1.8, 7.0) | 0.5 (0.2, 1.2) | NA | NA | NA |
|  | 7.1.1.1 Seminoma | 10/6 | 1.5 (0.7, 2.8) | 0.6 (-0.3, 1.9) | NA | NA | NA | NA | NA | NA |
|  | **9. Carcinomas** | **18/7** | **2.5 (1.5, 4.0)** | **1.7 (0.6, 3.3)** | NA | NA | NA | NA | NA | NA |
| **Females** | **2. Lymphomas** | **19/3** | **6.3 (3.8, 9.8)** | **3.0. (1.6, 5.1)** | **13/1** | **16.7 (8.9, 28.6)** | **2.3 (1.2, 4.1)** | **13/1** | **19.5 (10.4, 33.4)** | **2.3 (1.2, 4.1)** |
|  | 2.1 Non-Hodgkin lymphomas | 14/1 | 12.0 (6.6, 20.1) | 7.4 (3.7, 12.8) | 10/0 | 33.9 (16.3, 62.4) | 5.6 (2.6, 10.4) | 10/0 | 38.8 (18.6, 71.4) | 5.6 (2.6, 10.4) |
|  | **8. Melanoma, malignant** | **16/9** | **1.7 (1.0, 2.8)** | **0.5 (0.0, 1.2)** | NA | NA | NA | NA | NA | NA |
|  | **9. Carcinomas** | **49/29** | **1.7 (1.2, 2.2)** | **0.5 (0.2, 1.0)** | **19/7** | **2.6 (1.6, 4.1)** | **0.3 (0.1, 0.6)** | **19/6** | **2.9 (1.8, 4.6)** | **0.3 (0.1, 0.6)** |
|  | 9.6 Carcinoma of breast | 30/16 | 1.8 (1.2, 2.6) | 0.7 (0.2, 1.4) | 15/4 | 3.7 (2.1, 6.2) | 0.6 (0.2, 1.1) | 15/4 | 4.2 (2.3, 6.9) | 0.6 (0.2, 1.1) |
|  | 9.6.1 Breast, infiltrating duct | 23/13 | 1.8 (1.2, 2.7) | 0.7 (0.1, 1.4) | 14/3 | 4.5 (2.4, 7.5) | 0.7 (0.3, 1.3) | 14/3 | 5.0 (2.7, 8.4) | 0.7 (0.3, 1.3) |
|  | 9.7 Carcinoma of genital sites excluding ovary and testis | 14/7 | 2.0 (1.1, 3.4) | 0.8 (0.1, 2.0) | NA | NA | NA | NA | NA | NA |
|  | 9.7.1 Carcinoma of uterine cervix | 12/6 | 1.9 (1.0, 3.3) | 0.8 (0.0, 1.9) | NA | NA | NA | NA | NA | NA |
|  | 9.7.1.1 Cervix, squamous | 10/4 | 2.2 (1.1, 4.1) | 1.0 (0.1, 2.6) | NA | NA | NA | NA | NA | NA |

Abbreviation: NA=Not Applicable.

**Supplementary Table S4B.** Standardised incidence ratios (SIRs) and absolute excess risks (AERs) of second primary leukaemia’s after each distinct first primary cancer type among six-month adolescent and young adult (AYA, aged 15-39 years) cancer survivors compared to the general population in the Netherlands. Second primary cancer types are grouped according to the AYA-specific classification scheme developed by Barr and colleagues (2020). First and second cancer combinations with less than n=10 observed second cancers were excluded from the analyses. This Table presents outcomes from the sensitivity analysis.

|  | **Second primary cancers** | **1. Leukaemia’s and related disorders** | | | **1.2 Acute myeloid leukaemia** | | | **1.2.2 Other acute myeloid leukaemia** | | |
| --- | --- | --- | --- | --- | --- | --- | --- | --- | --- | --- |
|  | **First primary cancers** | **Obs/exp** | **SIR (95%CI)** | **AER per 10,000 person-years (95%CI)** | **Obs/exp** | **SIR (95%CI)** | **AER per 10,000 person-years (95%CI)** | **Obs/exp** | **SIR (95%CI)** | **AER per 10,000 person-years (95%CI)** |
| **Males** | **1. Leukaemia’s and related disorders** | 13/2 | 8.3 (4.4, 14.2) | 6.2 (2.9, 11.3) | NA | NA | NA | NA | NA | NA |
|  | **2. Lymphomas** | 37/6 | 6.0 (4.2, 8.3) | 4.4 (2.8, 6.4) | 15/1 | 11.4 (6.4, 18.8) | 1.9 (1.0, 3.3) | 14/1 | 12.2 (6.7, 20.5) | 1.8 (0.9, 3.2) |
|  | 2.1 Non-Hodgkin lymphomas | 15/3 | 5.8 (3.2, 9.6) | 4.5 (2.1, 8.1) | NA | NA | NA | NA | NA | NA |
|  | 2.2 Hodgkin lymphoma | 21/3 | 6.6 (4.1, 10.2) | 4.6 (2.5, 7.4) | NA | NA | NA | NA | NA | NA |
|  | 2.2.2 Hodgkin classic, other | 18/3 | 6.1 (3.6, 9.6) | 4.2 (2.1, 7.0) | NA | NA | NA | NA | NA | NA |
|  | **7. Gonadal and related tumours** | 26/12 | 2.1 (1.4, 3.1) | 1.0 (0.4, 1.9) | 11/3 | 4.2 (2.1, 7.5) | 0.6 (0.2, 1.2) | 10/2 | 4.4 (2.1, 8.1) | 0.6 (0.2, 1.2) |
|  | 7.1 Testis | 25/12 | 2.1 (1.4, 3.1) | 1.0 (0.3, 1.9) | 10/3 | 3.9 (1.9, 7.2) | 0.6 (0.2, 1.2) | NA | NA | NA |
|  | 7.1.1 Germ cell and trophoblastic | 25/12 | 2.1 (1.4, 3.1) | 1.0 (0.3, 1.9) | 10/3 | 3.9 (1.9, 7.2) | 0.6 (0.2, 1.2) | NA | NA | NA |
|  | 7.1.1.1 Seminoma | 10/6 | 1.6 (0.8, 2.9) | 0.6 (-0.2, 2.0) | NA | NA | NA | NA | NA | NA |
|  | **9. Carcinomas** | 19/7 | 2.7 (1.6, 4.3) | 1.9 (0.7, 3.6) | NA | NA | NA | NA | NA | NA |
| **Females** | **2. Lymphomas** | 20/3 | 6.6 (4.1, 10.3) | 3.2 (1.8, 5.3) | 13/1 | 16.8 (8.9, 28.7) | 2.3 (1.2, 4.1) | 13/1 | 19.5 (10.4, 33.4) | 2.3 (1.2, 4.1) |
|  | 2.1 Non-Hodgkin lymphomas | 14/1 | 12.0 (6.6, 20.2) | 7.4 (3.7, 12.9) | 10/0 | 34.0 (16.3, 62.5) | 5.6 (2.6, 10.4) | 10/0 | 38.9 (18.7, 71.6) | 5.6 (2.6, 10.4) |
|  | **8. Melanoma, malignant** | 16/9 | 1.8 (1.0, 2.9) | 0.5 (0.0, 1.3) | NA | NA | NA | NA | NA | NA |
|  | **9. Carcinomas** | 47/28 | 1.7 (1.2, 2.2) | 0.5 (0.2, 1.0) | 18/7 | 2.6 (1.5, 4.1) | 0.3 (0.1, 0.6) | 18/6 | 2.9 (1.7, 4.6) | 0.3 (0.1, 0.6) |
|  | 9.6 Carcinoma of breast | 28/15 | 1.8 (1.2, 2.6) | 0.7 (0.2, 1.4) | 14/4 | 3.7 (2.0, 6.2) | 0.5 (0.2, 1.1) | 14/3 | 4.1 (2.3, 6.9) | 0.6 (0.2, 1.1) |
|  | 9.6.1 Breast, infiltrating duct | 22/12 | 1.8 (1.2, 2.8) | 0.7 (0.1, 1.5) | 13/3 | 4.4 (2.3, 7.5) | 0.7 (0.3, 1.3) | 13/3 | 4.9 (2.6, 8.4) | 0.7 (0.3, 1.3) |
|  | 9.7 Carcinoma of genital sites excluding ovary and testis | 14/7 | 2.0 (1.1, 3.4) | 0.8 (0.1, 2.0) | NA | NA | NA | NA | NA | NA |
|  | 9.7.1 Carcinoma of uterine cervix | 12/6 | 1.9 (1.0, 3.3) | 0.8 (0.0, 1.9) | NA | NA | NA | NA | NA | NA |
|  | 9.7.1.1 Cervix, squamous | 10/4 | 2.2 (1.1, 4.1) | 1.0 (0.1, 2.6) | NA | NA | NA | NA | NA | NA |

Abbreviation: NA=Not Applicable.

**Supplementary Table S5A.** Standardised incidence ratios (SIRs) and absolute excess risks (AERs) of second primary lymphomas after each distinct first primary cancer type among six-month adolescent and young adult (AYA, aged 15-39 years) cancer survivors compared to the general population in the Netherlands. Second primary cancer types are grouped according to the AYA-specific classification scheme developed by Barr and colleagues (2020). First and second cancer combinations with less than n=10 observed second cancers were excluded from the analyses. This Table presents outcomes from the main analysis.

|  | **Second primary cancers** | **2. Lymphomas** | | | **2.1 Non-Hodgkin lymphomas** | | | **2.1.3 Diffuse large B-cell (DLBCL)** | | | **2.1.6 Follicular** | | | **2.2 Hodgkin lymphoma** | | | **2.2.2 Hodgkin classic, other** | | |
| --- | --- | --- | --- | --- | --- | --- | --- | --- | --- | --- | --- | --- | --- | --- | --- | --- | --- | --- | --- |
|  | **First primary cancers** | **Obs/exp** | **SIR (95%CI)** | **AER per 10,000 person-years (95%CI)** | **Obs/exp** | **SIR (95%CI)** | **AER per 10,000 person-years (95%CI)** | **Obs/exp** | **SIR (95%CI)** | **AER per 10,000 person-years (95%CI)** | **Obs/exp** | **SIR (95%CI)** | **AER per 10,000 person-years (95%CI)** | **Obs/exp** | **SIR (95%CI)** | **AER per 10,000 person-years (95%CI)** | **Obs/exp** | **SIR (95%CI)** | **AER per 10,000 person-years (95%CI)** |
| **Males** | **2. Lymphomas** | **38/11** | **3.4 (2.4, 4.6)** | **3.8 (2.2, 5.8)** | **23/6** | **3.6 (2.3, 5.4)** | **2.4 (1.2, 4.0)** | **13/3** | **4.7 (2.5, 8.0)** | **1.5 (0.6, 2.8)** | **NA** | **NA** | **NA** | **14/3** | **4.9 (2.7, 8.2)** | **1.6 (0.7, 2.9)** | **13/2** | **5.3 (2.8, 9.1)** | **1.5 (0.6, 2.8)** |
|  | 2.1 Non-Hodgkin lymphomas | 15/5 | 3.2 (1.8, 5.3) | 3.8 (1.4, 7.3) | NA | NA | NA | NA | NA | NA | NA | NA | NA | 14/1 | 12.6 (6.9, 21.2) | 4.7 (2.4, 8.1) | 13/1 | 13.8 (7.3, 23.5) | 4.4 (2.2, 7.7) |
|  | 2.2 Hodgkin lymphoma | 22/6 | 3.7 (2.3, 5.7) | 4.1 (2.0, 7.0) | 21/3 | 6.4 (3.9, 9.7) | 4.5 (2.5, 7.4) | 13/1 | 9.2 (4.9, 15.7) | 3.0. (1.4, 5.3) | NA | NA | NA | NA | NA | NA | NA | NA | NA |
|  | 2.2.2 Hodgkin classic, other | 19/5 | 3.5 (2.1, 5.4) | 3.7 (1.6, 6.7) | 18/3 | 5.8 (3.5, 9.2) | 4.1 (2.1, 7.0) | 12/1 | 9.0 (4.7, 15.8) | 2.9 (1.3, 5.4) | NA | NA | NA | NA | NA | NA | NA | NA | NA |
|  | **5. Blood and lymphatic vessel tumours** | **19/1** | **22.2 (13.4, 34.7)** | **38.1 (22.2, 60.5)** | **15/1** | **29.5 (16.5, 48.6)** | **30.4 (16.6, 50.9)** | **12/0** | **55.3 (28.6, 96.6)** | **24.7 (12.6, 43.6)** | **NA** | **NA** | **NA** | **NA** | **NA** | **NA** | **NA** | **NA** | **NA** |
|  | 5.2 Malignant blood and lymphatic vessel tumours, all sites | 19/1 | 22.2 (13.4, 34.7) | 38.1 (22.2, 60.5) | 15/1 | 29.5 (16.5, 48.6) | 30.4 (16.6, 50.9) | 12/0 | 55.3 (28.6, 96.6) | 24.7 (12.6, 43.6) | NA | NA | NA | NA | NA | NA | NA | NA | NA |
|  | 5.2.1 Kaposi sarcoma | 19/1 | 23.7 (14.3, 37.0) | 41.8 (24.4, 66.3) | 15/0 | 31.2 (17.5, 51.5) | 33.3 (18.2, 55.7) | 12/0 | 58.7 (30.3, 102.6) | 27.1 (13.8, 47.6) | NA | NA | NA | NA | NA | NA | NA | NA | NA |
|  | **7. Gonadal and related tumours** | **24/23** | **1.1 (0.7, 1.6)** | **0.1 (-0.5, 0.9)** | **16/13** | **1.2 (0.7, 2.0)** | **0.2 (-0.3, 0.9)** | **NA** | **NA** | **NA** | **NA** | **NA** | **NA** | **NA** | **NA** | **NA** | **NA** | **NA** | **NA** |
|  | 7.1 Testis | 24/22 | 1.1 (0.7, 1.6) | 0.1 (-0.5, 1.0) | 16/13 | 1.3 (0.7, 2.0) | 0.2 (-0.3, 1.0) | NA | NA | NA | NA | NA | NA | NA | NA | NA | NA | NA | NA |
|  | 7.1.1 Germ cell and trophoblastic | 24/22 | 1.1 (0.7, 1.6) | 0.1 (-0.5, 1.0) | 16/13 | 1.3 (0.7, 2.0) | 0.2 (-0.3, 1.0) | NA | NA | NA | NA | NA | NA | NA | NA | NA | NA | NA | NA |
|  | 7.1.1.1 Seminoma | 12/11 | 1.0 (0.5, 1.8) | 0.1 (-0.8, 1.5) | 10/7 | 1.5 (0.7, 2.7) | 0.5 (-0.3, 1.8) | NA | NA | NA | NA | NA | NA | NA | NA | NA | NA | NA | NA |
|  | **8. Melanoma, malignant** | **18/12** | **1.5 (0.9, 2.4)** | **0.9 (-0.2, 2.5)** | **NA** | **NA** | **NA** | **NA** | **NA** | **NA** | **NA** | **NA** | **NA** | **NA** | **NA** | **NA** | **NA** | **NA** | **NA** |
|  | 8.1 Superficial spreading/low cumulative sun damage melanoma | 11/7 | 1.5 (0.8, 2.7) | 0.9 (-0.4, 3.1) | NA | NA | NA | NA | NA | NA | NA | NA | NA | NA | NA | NA | NA | NA | NA |
|  | **9. Carcinomas** | **17/12** | **1.4 (0.8, 2.2)** | **0.7 (-0.4, 2.3)** | **13/7** | **1.8 (0.9, 3.0)** | **0.9 (-0.1, 2.3)** | **NA** | **NA** | **NA** | **NA** | **NA** | **NA** | **NA** | **NA** | **NA** | **NA** | **NA** | **NA** |
| **Females** | **2. Lymphomas** | **10/5** | **2.0 (0.9, 3.6)** | **0.9 (-0.1, 2.5)** | **NA** | **NA** | **NA** | **NA** | **NA** | **NA** | **NA** | **NA** | **NA** | **NA** | **NA** | **NA** | **NA** | **NA** | **NA** |
|  | **7. Gonadal and related tumours** | **11/3** | **3.2 (1.6, 5.7)** | **2.6 (0.7, 5.6)** | **NA** | **NA** | **NA** | **NA** | **NA** | **NA** | **NA** | **NA** | **NA** | **NA** | **NA** | **NA** | **NA** | **NA** | **NA** |
|  | 7.2 Ovary | 10/3 | 3.2 (1.5, 5.8) | 2.6 (0.6, 5.8) | NA | NA | NA | NA | NA | NA | NA | NA | NA | NA | NA | NA | NA | NA | NA |
|  | **8. Melanoma, malignant** | **14/15** | **0.9 (0.5, 1.6)** | **-0.1 (-0.5, 0.6)** | **10/9** | **1.1 (0.5, 2.0)** | **0.1 (-0.3, 0.7)** | **NA** | **NA** | **NA** | **NA** | **NA** | **NA** | **NA** | **NA** | **NA** | **NA** | **NA** | **NA** |
|  | **9. Carcinomas** | **50/46** | **1.1 (0.8, 1.4)** | **0.1 (-0.2, 0.5)** | **44/30** | **1.5 (1.1, 2.0)** | **0.4 (0.1, 0.8)** | **16/11** | **1.4 (0.8, 2.3)** | **0.1 (-0.1, 0.4)** | **12/7** | **1.6 (0.8, 2.9)** | **0.1 (0.0, 0.4)** | **NA** | **NA** | **NA** | **NA** | **NA** | **NA** |
|  | 9.6 Carcinoma of breast | 20/25 | 0.8 (0.5, 1.2) | -0.3 (-0.7, 0.3) | 19/16 | 1.2 (0.7, 1.8) | 0.1 (-0.3, 0.7) | NA | NA | NA | NA | NA | NA | NA | NA | NA | NA | NA | NA |
|  | 9.6.1 Breast, infiltrating duct | 13/20 | 0.7 (0.4, 1.1) | -0.4 (-0.8, 0.2) | 13/13 | 1.0 (0.5, 1.7) | 0.0 (-0.4, 0.6) | NA | NA | NA | NA | NA | NA | NA | NA | NA | NA | NA | NA |
|  | 9.7 Carcinoma of genital sites excluding ovary and testis | 14/11 | 1.3 (0.7, 2.2) | 0.4 (-0.4, 1.5) | 13/7 | 1.8 (1.0, 3.1) | 0.7 (0.0, 1.8) | NA | NA | NA | NA | NA | NA | NA | NA | NA | NA | NA | NA |
|  | 9.7.1 Carcinoma of uterine cervix | 11/10 | 1.1 (0.6, 2.0) | 0.2 (-0.6, 1.3) | 10/6 | 1.6 (0.7, 2.9) | 0.5 (-0.2, 1.6) | NA | NA | NA | NA | NA | NA | NA | NA | NA | NA | NA | NA |

Abbreviation: NA=Not Applicable.

**Supplementary Table S5B.** Standardised incidence ratios (SIRs) and absolute excess risks (AERs) of second primary lymphomas after each distinct first primary cancer type among six-month adolescent and young adult (AYA, aged 15-39 years) cancer survivors compared to the general population in the Netherlands. Second primary cancer types are grouped according to the AYA-specific classification scheme developed by Barr and colleagues (2020). First and second cancer combinations with less than n=10 observed second cancers were excluded from the analyses. This Table presents outcomes from the sensitivity analysis.

|  | **Second primary cancers** | **2. Lymphomas** | | | **2.1 Non-Hodgkin lymphomas** | | | **2.1.3 Diffuse large B-cell (DLBCL)** | | | **2.1.6 Follicular** | | | **2.2 Hodgkin lymphoma** | | | **2.2.2 Hodgkin classic, other** | | |
| --- | --- | --- | --- | --- | --- | --- | --- | --- | --- | --- | --- | --- | --- | --- | --- | --- | --- | --- | --- |
|  | **First primary cancers** | **Obs/exp** | **SIR (95%CI)** | **AER per 10,000 person-years (95%CI)** | **Obs/exp** | **SIR (95%CI)** | **AER per 10,000 person-years (95%CI)** | **Obs/exp** | **SIR (95%CI)** | **AER per 10,000 person-years (95%CI)** | **Obs/exp** | **SIR (95%CI)** | **AER per 10,000 person-years (95%CI)** | **Obs/exp** | **SIR (95%CI)** | **AER per 10,000 person-years (95%CI)** | **Obs/exp** | **SIR (95%CI)** | **AER per 10,000 person-years (95%CI)** |
| **Males** | **1. Leukaemia’s and related disorders** | **10/3** | **3.5 (1.7, 6.5)** | **3.9 (1.1, 8.5)** | **NA** | **NA** | **NA** | **NA** | **NA** | **NA** | **NA** | **NA** | **NA** | **NA** | **NA** | **NA** | **NA** | **NA** | **NA** |
|  | **2. Lymphomas** | **46/11** | **4.1 (3.0, 5.5)** | **5.0 (3.2, 7.1)** | **29/6** | **4.5 (3.0, 6.5)** | **3.2 (1.9, 5.0)** | **17/3** | **6.2 (3.6, 9.9)** | **2.0 (1.0, 3.5)** | **NA** | **NA** | **NA** | **15/3** | **5.2 (2.9, 8.7)** | **1.7 (0.8, 3.1)** | **13/2** | **5.3 (2.8, 9.1)** | **1.5 (0.6, 2.8)** |
|  | 2.1 Non-Hodgkin lymphomas | 17/5 | 3.7 (2.1, 5.9) | 4.5 (1.9, 8.2) | NA | NA | NA | NA | NA | NA | NA | NA | NA | NA | NA | NA |  |  |  |
|  | 2.2 Hodgkin lymphoma | 24/6 | 4.1 (2.6, 6.1) | 4.7 (2.4, 7.7) | 22/3 | 6.7 (4.2, 10.1) | 4.8 (2.7, 7.7) | 13/1 | 9.2 (4.9, 15.7) | 3.0 (1.4, 5.3) | NA | NA | NA | NA | NA | NA | NA | NA | NA |
|  | 2.2.2 Hodgkin classic, other | 21/5 | 3.8 (2.4, 5.9) | 4.3 (2.1, 7.4) | 19/3 | 6.2 (3.7, 9.6) | 4.4 (2.3, 7.3) | 12/1 | 9.0 (4.7, 15.8) | 2.9 (1.3, 5.4) | NA | NA | NA | NA | NA | NA | NA | NA | NA |
|  | **5. Blood and lymphatic vessel tumours** | **19/1** | **22.3 (13.4, 34.8)** | **38.1 (22.3, 60.6)** | **15/1** | **29.5 (16.5, 48.6)** | **30.5 (16.6, 50.9)** | **12/0** | **55.4 (28.6, 96.8)** | **24.8 (12.6, 43.6)** | **NA** | **NA** | **NA** | **NA** | **NA** | **NA** | **NA** | **NA** | **NA** |
|  | 5.2 Malignant blood and lymphatic vessel tumours, all sites | 19/1 | 22.3 (13.4, 34.8) | 38.1 (22.3, 60.6) | 15/1 | 29.5 (16.5, 48.6) | 30.5 (16.6, 50.9) | 12/0 | 55.4 (28.6, 96.8) | 24.8 (12.6, 43.6) | NA | NA | NA | 14/1 | 12.7 (6.9, 21.2) | 4.7 (2.4, 8.1) | 13/1 | 13.8 (7.3, 23.6) | 4.4 (2.2, 7.7) |
|  | 5.2.1 Kaposi sarcoma | 19/1 | 23.7 (14.3, 37.1) | 41.8 (24.4, 66.3) | 15/0 | 31.3 (17.5, 51.6) | 33.4 (18.2, 55.7) | 12/0 | 58.8 (30.4, 102.7) | 27.1 (13.8, 47.7) | NA | NA | NA | NA | NA | NA | NA | NA | NA |
|  | **7. Gonadal and related tumours** | **23/22** | **1.0 (0.7, 1.6)** | **0.1 (-0.6, 0.9)** | **15/13** | **1.2 (0.7, 1.9)** | **0.2 (-0.3, 0.9)** | **NA** | **NA** | **NA** | **NA** | **NA** | **NA** | **NA** | **NA** | **NA** | **NA** | **NA** | **NA** |
|  | 7.1 Testis | 23/22 | 1.1 (0.7, 1.6) | 0.1 (-0.5, 1.0) | 15/12 | 1.2 (0.7, 2.0) | 0.2 (-0.3, 0.9) | NA | NA | NA | NA | NA | NA | NA | NA | NA | NA | NA | NA |
|  | 7.1.1 Germ cell and trophoblastic | 23/22 | 1.1 (0.7, 1.6) | 0.1 (-0.5, 1.0) | 15/12 | 1.2 (0.7, 2.0) | 0.2 (-0.3, 0.9) | NA | NA | NA | NA | NA | NA | NA | NA | NA | NA | NA | NA |
|  | 7.1.1.1 Seminoma | 12/11 | 1.1 (0.6, 1.9) | 0.1 (-0.8, 1.6) | 10/7 | 1.5 (0.7, 2.8) | 0.6 (-0.3, 1.9) | NA | NA | NA | NA | NA | NA | NA | NA | NA | NA | NA | NA |
|  | **8. Melanoma, malignant** | **18/12** | **1.5 (0.9, 2.4)** | **1.0 (-0.1, 2.6)** | **NA** | **NA** | **NA** | **NA** | **NA** | **NA** | **NA** | **NA** | **NA** | **NA** | **NA** | **NA** | **NA** | **NA** | **NA** |
|  | 8.1 Superficial spreading/low cumulative sun damage melanoma | 11/7 | 1.6 (0.8, 2.8) | 1.0 (-0.4, 3.2) | NA | NA | NA | NA | NA | NA | NA | NA | NA | NA | NA | NA | NA | NA | NA |
|  | **9. Carcinomas** | **17/12** | **1.4 (0.8, 2.2)** | **0.8 (-0.4, 2.4)** | **13/7** | **1.8 (1.0, 3.1)** | **0.9 (-0.1, 2.4)** | **NA** | **NA** | **NA** | **NA** | **NA** | **NA** | **NA** | **NA** | **NA** | **NA** | **NA** | **NA** |
| **Females** | **2. Lymphomas** | **19/5** | **3.7 (2.2, 5.8)** | **2.6 (1.2, 4.7)** | **17/3** | **5.9 (3.4, 9.5)** | **2.7 (1.3, 4.6)** | **NA** | **NA** | **NA** | **NA** | **NA** | **NA** | **NA** | **NA** | **NA** | **NA** | **NA** | **NA** |
|  | 2.2 Hodgkin lymphoma | 10/3 | 3.4 (1.6, 6.3) | 2.1 (0.6, 4.7) | NA | NA | NA | NA | NA | NA | NA | NA | NA | NA | NA | NA | NA | NA | NA |
|  | 2.2.2 Hodgkin classic, other | 10/3 | 3.5 (1.7, 6.4) | 2.2 (0.6, 4.8) | NA | NA | NA | NA | NA | NA | NA | NA | NA | NA | NA | NA | NA | NA | NA |
|  | **7. Gonadal and related tumours** | **10/3** | **2.9 (1.4, 5.4)** | **2.3 (0.5, 5.2)** | **NA** | **NA** | **NA** | **NA** | **NA** | **NA** | **NA** | **NA** | **NA** | **NA** | **NA** | **NA** | **NA** | **NA** | **NA** |
|  | **8. Melanoma, malignant** | **14/14** | **1.0 (0.5, 1.6)** | **0.0 (-0.5, 0.7)** | **10/9** | **1.1 (0.5, 2.1)** | **0.1 (-0.3, 0.7)** | **NA** | **NA** | **NA** | **NA** | **NA** | **NA** | **NA** | **NA** | **NA** | **NA** | **NA** | **NA** |
|  | **9. Carcinomas** | **50/44** | **1.1 (0.8, 1.5)** | **0.2 (-0.2, 0.6)** | **44/28** | **1.5 (1.1, 2.1)** | **0.4 (0.1, 0.9)** | **15/11** | **1.4 (0.8, 2.3)** | **0.1 (-0.1, 0.4)** | **13/7** | **1.8 (1.0, 3.2)** | **0.2 (0.0, 0.4)** | **NA** | **NA** | **NA** | **NA** | **NA** | **NA** |
|  | 9.6 Carcinoma of breast | 20/24 | 0.8 (0.5, 1.3) | -0.2 (-0.6, 0.4) | 19/15 | 1.2 (0.7, 1.9) | 0.2 (-0.2, 0.8) | NA | NA | NA | NA | NA | NA | NA | NA | NA | NA | NA | NA |
|  | 9.6.1 Breast, infiltrating duct | 14/18 | 0.8 (0.4, 1.3) | -0.3 (-0.7, 0.3) | 14/12 | 1.2 (0.6, 2.0) | 0.1 (-0.3, 0.8) | NA | NA | NA | NA | NA | NA | NA | NA | NA | NA | NA | NA |
|  | 9.7 Carcinoma of genital sites excluding ovary and testis | 14/11 | 1.3 (0.7, 2.2) | 0.4 (-0.4, 1.5) | 13/7 | 1.8 (1.0, 3.1) | 0.7 (0.0, 1.8) | NA | NA | NA | NA | NA | NA | NA | NA | NA | NA | NA | NA |
|  | 9.7.1 Carcinoma of uterine cervix | 11/10 | 1.1 (0.6, 2.0) | 0.2 (-0.6, 1.3) | 10/6 | 1.6 (0.7, 2.9) | 0.5 (-0.2, 1.6) | NA | NA | NA | NA | NA | NA | NA | NA | NA | NA | NA | NA |

Abbreviation: NA=Not Applicable.

**Supplementary Table S6A.** Standardised incidence ratios (SIRs) and absolute excess risks (AERs) of second primary central nervous system and other intracranial and intraspinal neoplasms after each distinct first primary cancer type among six-month adolescent and young adult (AYA, aged 15-39 years) cancer survivors compared to the general population in the Netherlands. Second primary cancer types are grouped according to the AYA-specific classification scheme developed by Barr and colleagues (2020). First and second cancer combinations with less than n=10 observed second cancers were excluded from the analyses. This Table presents outcomes from the main analysis.

|  | **Second primary cancers** | **3. CNS and other intracranial and intraspinal neoplasms** | | | **3.1 Astroglial and related neoplasms** | | |
| --- | --- | --- | --- | --- | --- | --- | --- |
|  | **First primary cancers** | **Obs/exp** | **SIR (95%CI)** | **AER per 10,000 person-years (95%CI)** | **Obs/exp** | **SIR (95%CI)** | **AER per 10,000 person-years (95%CI)** |
| **Males** | **7. Gonadal and related tumours** | **12/9** | **1.3 (0.7, 2.4)** | **0.2 (-0.2, 0.9)** | **11/8** | **1.4 (0.7, 2.5)** | **0.2 (-0.2, 0.8)** |
|  | 7.1 Testis | 11/9 | 1.3 (0.6, 2.3) | 0.2 (-0.2, 0.8) | 10/8 | 1.3 (0.6, 2.4) | 0.2 (-0.2, 0.8) |
|  | 7.1.1 Germ cell and trophoblastic | 11/9 | 1.3 (0.6, 2.3) | 0.2 (-0.2, 0.8) | 10/8 | 1.3 (0.6, 2.4) | 0.2 (-0.2, 0.8) |
| **Females** | **8. Melanoma, malignant** | **12/5** | **2.2 (1.1, 3.8)** | **0.5 (0.1, 1.2)** | **11/5** | **2.2 (1.1, 3.9)** | **0.4 (0.0, 1.1)** |
|  | **9. Carcinomas** | **22/17** | **1.3 (0.8, 2.0)** | **0.2 (-0.1, 0.5)** | **19/15** | **1.3 (0.8, 2.0)** | **0.1 (-0.1, 0.4)** |

Abbreviation: CNS=Central Nervous System.

**Supplementary Table S6B.** Standardised incidence ratios (SIRs) and absolute excess risks (AERs) of second primary central nervous system and other intracranial and intraspinal neoplasms after each distinct first primary cancer type among six-month adolescent and young adult (AYA, aged 15-39 years) cancer survivors compared to the general population in the Netherlands. Second primary cancer types are grouped according to the AYA-specific classification scheme developed by Barr and colleagues (2020). First and second cancer combinations with less than n=10 observed second cancers were excluded from the analyses. This Table presents outcomes from the sensitivity analysis.

|  | **Second primary cancers** | **3. CNS and other intracranial and intraspinal neoplasms** | | | **3.1 Astroglial and related neoplasms** | | |
| --- | --- | --- | --- | --- | --- | --- | --- |
|  | **First primary cancers** | **Obs/exp** | **SIR (95%CI)** | **AER per 10,000 person-years (95%CI)** | **Obs/exp** | **SIR (95%CI)** | **AER per 10,000 person-years (95%CI)** |
| **Males** | **3. CNS and other intracranial and intraspinal neoplasms** | **21/1** | **21.2 (13.1, 32.4)** | **11.2 (6.7, 17.4)** | **19/1** | **21.4 (12.9, 33.5)** | **10.1 (5.9, 16.1)** |
|  | 3.1 Astroglial and related neoplasms | 18/1 | 20.5 (12.2, 32.4) | 11.0 (6.3, 17.7) | 16/1 | 20.3 (11.6, 33.0) | 9.8 (5.4, 16.2) |
|  | **7. Gonadal and related tumours** | **13/9** | **1.5 (0.8, 2.6)** | **0.3 (-0.1, 1.0)** | **12/8** | **1.5 (0.8, 2.7)** | **0.3 (-0.1, 1.0)** |
|  | 7.1 Testis | 11/9 | 1.3 (0.6, 2.3) | 0.2 (-0.2, 0.8) | 10/8 | 1.3 (0.6, 2.4) | 0.2 (-0.2, 0.8) |
|  | 7.1.1 Germ cell and trophoblastic | 11/8 | 1.3 (0.6, 2.3) | 0.2 (-0.2, 0.8) | 10/8 | 1.3 (0.6, 2.4) | 0.2 (-0.2, 0.8) |
| **Females** | **3. CNS and other intracranial and intraspinal neoplasms** | **16/0** | **34.9 (20.0, 56.7)** | **11.6 (6.5, 19.1)** | **15/0** | **36.1 (20.2, 59.5)** | **10.9 (6.0, 18.2)** |
|  | 3.1 Astroglial and related neoplasms | 14/0 | 35.2 (19.2, 59.0) | 11.8 (6.3, 20.0) | 13/0 | 36.0 (19.2, 61.6) | 11.0 (5.7, 19.0) |
|  | **8. Melanoma, malignant** | **12/5** | **2.3 (1.2, 4.0)** | **0.5 (0.1, 1.2)** | **11/5** | **2.3 (1.1, 4.1)** | **0.5 (0.1, 1.1)** |
|  | **9. Carcinomas** | **20/16** | **1.3 (0.8, 1.9)** | **0.1 (-0.1, 0.4)** | **17/15** | **1.2 (0.7, 1.9)** | **0.1 (-0.1, 0.4)** |

Abbreviation: CNS=Central Nervous System.

**Supplementary Table S7A.** Standardised incidence ratios (SIRs) and absolute excess risks (AERs) of second primary sarcomas after each distinct first primary cancer type among six-month adolescent and young adult (AYA, aged 15-39 years) cancer survivors compared to the general population in the Netherlands. Second primary cancer types are grouped according to the AYA-specific classification scheme developed by Barr and colleagues (2020). First and second cancer combinations with less than n=10 observed second cancers were excluded from the analyses. This Table presents outcomes from the main analysis.

|  | **Second primary cancers** | **4. Sarcomas** | | | **4.1 Osteosarcoma** | | | **4.2 Chondrosarcoma** | | |
| --- | --- | --- | --- | --- | --- | --- | --- | --- | --- | --- |
|  | **First primary cancers** | **Obs/exp** | **SIR (95%CI)** | **AER per 10,000 person-years (95%CI)** | **Obs/exp** | **SIR (95%CI)** | **AER per 10,000 person-years (95%CI)** | **Obs/exp** | **SIR (95%CI)** | **AER per 10,000 person-years (95%CI)** |
| **Males** | **7. Gonadal and related tumours** | **10/5** | **1.9 (0.9, 3.4)** | **0.3 (0.0, 0.9)** | **NA** | **NA** | **NA** | **NA** | **NA** | **NA** |
|  | **9. Carcinomas** | **10/3** | **3.5 (1.7, 6.5)** | **1.1 (0.3, 2.4)** | **NA** | **NA** | **NA** | **NA** | **NA** | **NA** |
| **Females** | **8. Melanoma, malignant** | **13/6** | **2.3 (1.2, 4.0)** | **0.6 (0.1, 1.2)** | **NA** | **NA** | **NA** | **NA** | **NA** | **NA** |
|  | **9. Carcinomas** | **72/17** | **4.2 (3.3, 5.3)** | **1.5 (1.1, 2.0.)** | **10/1** | **16 (7.7, 29.4)** | **0.3 (0.1, 0.5)** | **19/1** | **12.9 (7.8, 20.2)** | **0.5 (0.3, 0.8)** |
|  | 9.6 Carcinoma of breast | 46/10 | 4.8 (3.5, 6.4) | 1.9 (1.2, 2.7) | NA | NA | NA | NA | NA | NA |
|  | 9.6.1 Breast, infiltrating duct | 33/7 | 4.4 (3.0, 6.2) | 1.7 (1.0, 2.5) | NA | NA | NA | NA | NA | NA |
|  | 9.7 Carcinoma of genital sites excluding ovary and testis | 12/4 | 3.1 (1.6, 5.3) | 1.0 (0.3, 2.1) | NA | NA | NA | 15/1 | 17.6 (9.9, 29.1) | 0.7 (0.4, 1.2) |
|  | 9.7.1 Carcinoma of uterine cervix | 11/4 | 3.1 (1.5, 5.5) | 1.0 (0.3, 2.1) | NA | NA | NA | NA | NA | NA |
|  | 9.7.1.1 Cervix, squamous | 10/3 | 4.0 (1.9, 7.3) | 1.4 (0.4, 3.0) | NA | NA | NA | NA | NA | NA |

Abbreviation: NA=Not Applicable.

**Supplementary Table S7B.** Standardised incidence ratios (SIRs) and absolute excess risks (AERs) of second primary sarcomas after each distinct first primary cancer type among six-month adolescent and young adult (AYA, aged 15-39 years) cancer survivors compared to the general population in the Netherlands. Second primary cancer types are grouped according to the AYA-specific classification scheme developed by Barr and colleagues (2020). First and second cancer combinations with less than n=10 observed second cancers were excluded from the analyses. This Table presents outcomes from the sensitivity analysis.

|  | **Second primary cancers** | **4. Sarcomas** | | | **4.2 Chondrosarcoma** | | |
| --- | --- | --- | --- | --- | --- | --- | --- |
|  | **First primary cancers** | **Obs/exp** | **SIR (95%CI)** | **AER per 10,000 person-years (95%CI)** | **Obs/exp** | **SIR (95%CI)** | **AER per 10,000 person-years (95%CI)** |
| **Males** | **4. Sarcomas** | **16/1** | **16.3 (9.3, 26.5)** | **6.0 (3.2, 9.9)** | **NA** | **NA** | **NA** |
|  | **7. Gonadal and related tumours** | **10/5** | **1.9 (0.9, 3.5)** | **0.3 (0.0, 1.0)** | **NA** | **NA** | **NA** |
|  | **9. Carcinomas** | **10/3** | **3.6 (1.7, 6.6)** | **1.1 (0.3, 2.5)** | **NA** | **NA** | **NA** |
| **Females** | **4. Sarcomas** | **13/1** | **14.2 (7.5, 24.2)** | **5.0 (2.5, 8.8)** | **NA** | **NA** | **NA** |
|  | **8. Melanoma, malignant** | **13/5** | **2.4 (1.3, 4.1)** | **0.6 (0.1, 1.3)** | **NA** | **NA** | **NA** |
|  | **9. Carcinomas** | **67/17** | **4.0 (3.1, 5.1)** | **1.4 (1.0, 1.9)** | **18/2** | **12.0 (7.1, 18.9)** | **0.5 (0.3, 0.8)** |
|  | 9.6 Carcinoma of breast | 41/9 | 4.5 (3.2, 6.1) | 1.7 (1.1, 2.5) | 14/1 | 15.8 (8.6, 26.5) | 0.7 (0.4, 1.2) |
|  | 9.6.1 Breast, infiltrating duct | 32/7 | 4.5 (3.1, 6.3) | 1.7 (1.0, 2.6) | NA | NA | NA |
|  | 9.7 Carcinoma of genital sites excluding ovary and testis | 12/4 | 3.1 (1.6, 5.3) | 1.0 (0.3, 2.1) | NA | NA | NA |
|  | 9.7.1 Carcinoma of uterine cervix | 11/4 | 3.1 (1.5, 5.5) | 1.0 (0.3, 2.1) | NA | NA | NA |
|  | 9.7.1.1 Cervix, squamous | 10/3 | 4.0 (1.9, 7.3) | 1.4 (0.4, 3.0) | NA | NA | NA |

Abbreviation: NA=Not Applicable.

**Supplementary Table S8A.** Standardised incidence ratios (SIRs) and absolute excess risks (AERs) of second primary gonadal and related tumours after each distinct first primary cancer type among six-month adolescent and young adult (AYA, aged 15-39 years) cancer survivors compared to the general population in the Netherlands. Second primary cancer types are grouped according to the AYA-specific classification scheme developed by Barr and colleagues (2020). First and second cancer combinations with less than n=10 observed second cancers were excluded from the analyses. This Table presents outcomes from the main analysis.

|  | **Second primary cancers** | **7. Gonadal and related tumours** | | | **7.1 Testis** | | | **7.1.1 Germ cell and trophoblastic** | | | **7.2 Ovary** | | | **7.2.2 Non-germ cell** | | | **7.2.2.1 Carcinoma** | | | **7.2.2.1.1 Adenocarcinoma** | | | **7.2.2.1.1.2 Cystadenocarcinoma** | | | **7.2.2.1.1.5 Endometrioid** | | | **7.2.2.1.1.6 Other adenocarcinoma** | | |
| --- | --- | --- | --- | --- | --- | --- | --- | --- | --- | --- | --- | --- | --- | --- | --- | --- | --- | --- | --- | --- | --- | --- | --- | --- | --- | --- | --- | --- | --- | --- | --- |
|  | **First primary cancers** | **Obs/exp** | **SIR (95%CI)** | **AER per 10,000 person-years (95%CI)** | **Obs/exp** | **SIR (95%CI)** | **AER per 10,000 person-years (95%CI)** | **Obs/exp** | **SIR (95%CI)** | **AER per 10,000 person-years (95%CI)** | **Obs/exp** | **SIR (95%CI)** | **AER per 10,000 person-years (95%CI)** | **Obs/exp** | **SIR (95%CI)** | **AER per 10,000 person-years (95%CI)** | **Obs/exp** | **SIR (95%CI)** | **AER per 10,000 person-years (95%CI)** | **Obs/exp** | **SIR (95%CI)** | **AER per 10,000 person-years (95%CI)** | **Obs/exp** | **SIR (95%CI)** | **AER per 10,000 person-years (95%CI)** | **Obs/exp** | **SIR (95%CI)** | **AER per 10,000 person-years (95%CI)** | **Obs/exp** | **SIR**  **(95%CI)** | **AER per 10,000 person-years (95%CI)** |
| **Males** | **2. Lymphomas** | **14/15** | **0.9  (0.5, 1.6)** | **-0.1  (-1.0, 1.2)** | **14/15** | **1.0  (0.5, 1.6)** | **-0.1  (-1.0, 1.3)** | **14/15** | **1.0  (0.5, 1.6)** | **-0.1  (-1.0, 1.3)** | **NA** | **NA** | **NA** | **NA** | **NA** | **NA** | **NA** | **NA** | **NA** | **NA** | **NA** | **NA** | **NA** | **NA** | **NA** | **NA** | **NA** | **NA** | **NA** | **NA** | **NA** |
|  | **7. Gonadal and related tumours** | **16/32** | **0.5  (0.3, 0.8)** | **-1.1  (-1.6, -0.4)** | **14/31** | **0.4  (0.2, 0.8)** | **-1.2  (-1.7, -0.5)** | **14/31** | **0.4  (0.2, 0.8)** | **-1.2  (-1.7, -0.5)** | **NA** | **NA** | **NA** | **NA** | **NA** | **NA** | **NA** | **NA** | **NA** | **NA** | **NA** | **NA** | **NA** | **NA** | **NA** | **NA** | **NA** | **NA** | **NA** | **NA** | **NA** |
|  | 7.4 Germ cell and trophoblastic excluding CNS, ovary, testis | 13/1 | 21.3  (11.3, 36.4) | 46.6  (23.7, 81.3) | 13/1 | 21.8 (11.6, 37.3) | 46.6  (23.8, 81.3) | 13/1 | 21.8  (11.6, 37.4) | 46.6  (23.8, 81.3) | NA | NA | NA | NA | NA | NA | NA | NA | NA | NA | NA | NA | NA | NA | NA | NA | NA | NA | NA | NA | NA |
|  | **8. Melanoma, malignant** | **10/13** | **0.7  (0.4, 1.4)** | **-0.5  (-1.3, 0.8)** | **10/13** | **0.8  (0.4, 1.4)** | **-0.5  (-1.3, 0.8)** | **10/13** | **0.8  (0.4, 1.4)** | **-0.5  (-1.3, 0.8)** | **NA** | **NA** | **NA** | **NA** | **NA** | **NA** | **NA** | **NA** | **NA** | **NA** | **NA** | **NA** | **NA** | **NA** | **NA** | **NA** | **NA** | **NA** | **NA** | **NA** | **NA** |
| **Females** | **9. Carcinomas** | **101/29** | **3.4  (2.8, 4.2)** | **2.0  (1.5, 2.6)** | **NA** | **NA** | **NA** | **NA** | **NA** | **NA** | **101/29** | **3.5  (2.9, 4.3)** | **2.0  (1.5, 2.6)** | **101/27** | **3.7  (3.0, 4.5)** | **2.0  (1.5, 2.6)** | **101/26** | **3.9  (3.2, 4.8)** | **2.1  (1.6, 2.7)** | **101/25** | **4.0  (3.3, 4.9)** | **2.1  (1.6, 2.7)** | **69/14** | **5.0  (3.9, 6.3)** | **1.5  (1.1, 2.0)** | **10/4** | **2.7  (1.3, 4.9)** | **0.2  (0.0, 0.4)** | **14/2** | **7.6**  **(4.1, 12.7)** | **0.3  (0.2, 0.6)** |
|  | 9.6 Carcinoma of breast | 72/16 | 4.4  (3.4, 5.5) | 2.9  (2.1, 3.8) | NA | NA | NA | NA | NA | NA | 72/16 | 4.5 (3.5, 5.7) | 2.9  (2.1, 3.8) | 72/15 | 4.7  (3.7, 5.9) | 2.9  (2.1, 3.9) | 72/14 | 5.0  (3.9, 6.3) | 3.0  (2.2, 3.9) | 72/14 | 5.1  (4.0, 6.4) | 3.0  (2.2, 3.9) | 52/8 | 6.7  (5.0, 8.8) | 2.3  (1.6, 3.1) | NA | NA | NA | 11/1 | 10.4  (5.2, 18.6) | 0.5  (0.2, 1.0) |
|  | 9.6.1 Breast, infiltrating duct | 53/13 | 4.2  (3.1, 5.5) | 2.6  (1.8, 3.7) | NA | NA | NA | NA | NA | NA | 53/12 | 4.3 (3.2, 5.6) | 2.6  (1.8, 3.7) | 53/12 | 4.5  (3.4, 5.9) | 2.7  (1.8, 3.7) | 53/11 | 4.7  (3.6, 6.2) | 2.7  (1.9, 3.8) | 53/11 | 4.8  (3.6, 6.3) | 2.7  (1.9, 3.8) | 38/6 | 6.4  (4.5, 8.7) | 2.1  (1.4, 3.0) | NA | NA | NA | NA | NA | NA |
|  | 9.7 Carcinoma of genital sites excluding ovary and testis | 16/7 | 2.3  (1.3, 3.7) | 1.1  (0.3, 2.3) | NA | NA | NA | NA | NA | NA | 16/7 | 2.4 (1.4, 3.9) | 1.1  (0.3, 2.3) | 16/6 | 2.5  (1.4, 4.0) | 1.2  (0.3, 2.4) | 16/6 | 2.6  (1.5, 4.3) | 1.2  (0.4, 2.4) | 16/6 | 2.7  (1.5, 4.3) | 1.2  (0.4, 2.4) | 10/3 | 3.1  (1.5, 5.6) | 0.8  (0.2, 1.8) | NA | NA | NA | NA | NA | NA |
|  | 9.7.1 Carcinoma of uterine cervix | 15/6 | 2.4  (1.3, 3.9) | 1.2  (0.3, 2.4) | NA | NA | NA | NA | NA | NA | 15/6 | 2.5 (1.4, 4.1) | 1.2  (0.3, 2.5) | 15/6 | 2.6  (1.4, 4.2) | 1.2  (0.3, 2.5) | 15/5 | 2.7  (1.5, 4.5) | 1.3  (0.4, 2.5) | 15/5 | 2.8  (1.6, 4.6) | 1.3  (0.4, 2.6) | 10/3 | 3.4  (1.6, 6.3) | 0.9  (0.2, 2.0) | NA | NA | NA | NA | NA | NA |

Abbreviation: CNS=Central Nervous System, NA=Not Applicable.

**Supplementary Table S8B.** Standardised incidence ratios (SIRs) and absolute excess risks (AERs) of second primary gonadal and related tumours after each distinct first primary cancer type among six-month adolescent and young adult (AYA, aged 15-39 years) cancer survivors compared to the general population in the Netherlands. Second primary cancer types are grouped according to the AYA-specific classification scheme developed by Barr and colleagues (2020). First and second cancer combinations with less than n=10 observed second cancers were excluded from the analyses. This Table presents outcomes from the sensitivity analysis for males.

|  | **Second primary cancers** | **7. Gonadal and related tumours** | | | **7.1 Testis** | | | **7.1.1 Germ cell and trophoblastic** | | | **7.1.1.1 Seminoma** | | | **7.1.1.2 Embryonal carcinoma** | | | **7.1.1.4 Teratoma** | | | **7.1.1.5 Mixed germ cell** | | |
| --- | --- | --- | --- | --- | --- | --- | --- | --- | --- | --- | --- | --- | --- | --- | --- | --- | --- | --- | --- | --- | --- | --- |
|  | **First primary cancers** | **Obs/exp** | **SIR (95%CI)** | **AER per 10,000 person-years (95%CI)** | **Obs/exp** | **SIR (95%CI)** | **AER per 10,000 person-years (95%CI)** | **Obs/exp** | **SIR (95%CI)** | **AER per 10,000 person-years (95%CI)** | **Obs/exp** | **SIR (95%CI)** | **AER per 10,000 person-years (95%CI)** | **Obs/exp** | **SIR (95%CI)** | **AER per 10,000 person-years (95%CI)** | **Obs/exp** | **SIR (95%CI)** | **AER per 10,000 person-years (95%CI)** | **Obs/exp** | **SIR (95%CI)** | **AER per 10,000 person-years (95%CI)** |
| **Males** | **2. Lymphomas** | **14/15** | **0.9  (0.5, 1.6)** | **-0.1  (-1.0, 1.2)** | **14/15** | **1.0  (0.5, 1.6)** | **-0.1  (-1.0, 1.3)** | **14/15** | **1.0  (0.5, 1.6)** | **-0.1  (-1.0, 1.3)** | **NA** | **NA** | **NA** | **NA** | **NA** | **NA** | **NA** | **NA** | **NA** | **NA** | **NA** | **NA** |
|  | **7. Gonadal and related tumours** | **293/31** | **9.4  (8.4, 10.5)** | **19.0  (16.6, 21.6)** | **291/30** | **9.6  (8.5, 10.7)** | **18.9  (16.5, 21.5)** | **291/30** | **9.6  (8.5, 10.7)** | **18.9  (16.5, 21.5)** | **194/19** | **10.2  (8.8, 11.7)** | **12.7  (10.8, 14.8)** | **27/3** | **10.8  (7.1, 15.7)** | **1.8  (1.1, 2.7)** | **10/1** | **8.6**  **(4.1, 15.8)** | **0.6**  **(0.3, 1.2)** | **54/7** | **8.1**  **(6.1, 10.5)** | **3.4**  **(2.5, 4.6)** |
|  | 7.1 Testis | 279/30 | 9.2  (8.2, 10.4) | 18.6  (16.2, 21.1) | 277/30 | 9.4  (8.3, 10.5) | 18.5  (16.1, 21.0) | 277/30 | 9.4  (8.3, 10.5) | 18.5  (16.1, 21.0) | 185/19 | 10.0  (8.6, 11.5) | 12.4  (10.5, 14.6) | 27/2 | 11.1  (7.3, 16.2) | 1.8  (1.1, 2.7) | 10/1 | 8.9  (4.3, 16.4) | 0.7  (0.3, 1.3) | 10/1 | 8.9  (4.3, 16.4) | 0.7  (0.3, 1.3) |
|  | 7.1.1 Germ cell and trophoblastic | 279/30 | 9.2  (8.2, 10.4) | 18.6  (16.2, 21.2) | 277/30 | 9.4  (8.3, 10.5) | 18.5  (16.1, 21.1) | 277/29 | 9.4  (8.3, 10.6) | 18.5  (16.1, 21.1) | 185/19 | 10.0  (8.6, 11.5) | 12.4  (10.5, 14.6) | 27/2 | 11.2  (7.4, 16.2) | 1.8  (1.1, 2.8) | 10/1 | 8.9  (4.3, 16.4) | 0.7  (0.3, 1.3) | 49/6 | 7.6  (5.6, 10.0) | 3.2  (2.2, 4.4) |
|  | 7.1.1.1 Seminoma | 146/13 | 11.1  (9.4, 13.1) | 21.5  (17.8, 25.7) | 145/13 | 11.3  (9.5, 13.3) | 21.4  (17.7, 25.6) | 145/13 | 11.3  (9.6, 13.3) | 21.4  (17.7, 25.6) | 108/9 | 12.5  (10.3, 15.1) | 16.1  (13.0, 19.7) | 13/1 | 14.4  (7.7, 24.6) | 2.0  (1.0, 3.5) | NA | NA | NA | 18/2 | 7.4  (4.4, 11.7) | 2.5  (1.3, 4.2) |
|  | 7.1.1.2 Embryonal carcinoma | 28/5 | 5.8  (3.9, 8.5) | 11.5  (6.9, 17.7) | 27/5 | 5.8  (3.8, 8.4) | 11.1  (6.5, 17.2) | 27/5 | 5.8  (3.8, 8.4) | 11.1  (6.5, 17.2) | 18/3 | 6.5  (3.8, 10.2) | 7.6  (3.9, 12.7) | NA | NA | NA | NA | NA | NA | NA | NA | NA |
|  | 7.1.1.4 Teratoma | 43/5 | 8.5  (6.1, 11.4) | 16.9  (11.6, 23.5) | 43/5 | 8.7  (6.3, 11.7) | 16.9  (11.6, 23.6) | 43/5 | 8.7  (6.3, 11.7) | 16.9  (11.6, 23.6) | 22/3 | 7.4  (4.7, 11.2) | 8.5  (4.8, 13.5) | NA | NA | NA | NA | NA | NA | 12/1 | 10.2  (5.3, 17.9) | 4.8  (2.2, 8.8) |
|  | 7.1.1.5 Mixed germ cell | 44/5 | 9.1  (6.6, 12.2) | 20.1  (13.9, 27.8) | 44/5 | 9.3  (6.8, 12.5) | 20.2  (14.0, 27.9) | 44/5 | 9.4  (6.8, 12.6) | 20.2  (14.0, 27.9) | 26/3 | 9.4  (6.1, 13.7) | 11.9  (7.3, 18.1) | NA | NA | NA | NA | NA | NA | 11/1 | 9.5  (4.8, 17.1) | 5.1  (2.2, 9.5) |
|  | 7.1.1.6 Choriocarcinoma and other trophoblastic | 10/1 | 6.9  (3.3, 12.7) | 14.0  (5.5, 27.8) | 10/1 | 7.1  (3.4, 13.0) | 14.1  (5.5, 27.9) | 10/1 | 7.1  (3.4, 13.0) | 14.1  (5.6, 27.9) | NA | NA | NA | NA | NA | NA | NA | NA | NA | NA | NA | NA |
|  | 7.4 Germ cell and trophoblastic excluding CNS, ovary, testis | 13/1 | 21.3  (11.3, 36.4) | 46.6  (23.7, 81.3) | 13/1 | 21.8  (11.6, 37.3) | 46.6  (23.8, 81.3) | 13/1 | 21.8  (11.6, 37.4) | 46.6  (23.8, 81.3) | NA | NA | NA | NA | NA | NA | NA | NA | NA | NA | NA | NA |
|  | **8. Melanoma, malignant** | **10/13** | **0.8  (0.4, 1.4)** | **-0.5  (-1.3, 0.8)** | **10/13** | **0.8  (0.4, 1.4)** | **-0.4  (-1.2, 0.9)** | **10/13** | **0.8  (0.4, 1.4)** | **-0.4  (-1.2, 0.9)** | **NA** | **NA** | **NA** | **NA** | **NA** | **NA** | **NA** | **NA** | **NA** | **NA** | **NA** | **NA** |

Abbreviation: CNS=Central Nervous System, NA=Not Applicable.

**Supplementary Table S8C.** Standardised incidence ratios (SIRs) and absolute excess risks (AERs) of second primary gonadal and related tumours after each distinct first primary cancer type among six-month adolescent and young adult (AYA, aged 15-39 years) cancer survivors compared to the general population in the Netherlands. Second primary cancer types are grouped according to the AYA-specific classification scheme developed by Barr and colleagues (2020). First and second cancer combinations with less than n=10 observed second cancers were excluded from the analyses. This Table presents outcomes from the sensitivity analysis for females.

|  | **Second primary cancers** | **7. Gonadal and related tumours** | | | **7.2 Ovary** | | | **7.2.2 Non-germ cell** | | | **7.2.2.1 Carcinoma** | | | **7.2.2.1.1 Adenocarcinoma** | | | **7.2.2.1.1.2 Cystadenocarcinoma** | | | **7.2.2.1.1.5 Endometrioid** | | | **7.2.2.1.1.6 Other adenocarcinoma** | | |
| --- | --- | --- | --- | --- | --- | --- | --- | --- | --- | --- | --- | --- | --- | --- | --- | --- | --- | --- | --- | --- | --- | --- | --- | --- | --- |
|  | **First primary cancers** | **Obs/exp** | **SIR (95%CI)** | **AER per 10,000 person-years (95%CI)** | **Obs/exp** | **SIR (95%CI)** | **AER per 10,000 person-years (95%CI)** | **Obs/exp** | **SIR (95%CI)** | **AER per 10,000 person-years (95%CI)** | **Obs/exp** | **SIR (95%CI)** | **AER per 10,000 person-years (95%CI)** | **Obs/exp** | **SIR (95%CI)** | **AER per 10,000 person-years (95%CI)** | **Obs/exp** | **SIR (95%CI)** | **AER per 10,000 person-years (95%CI)** | **Obs/exp** | **SIR (95%CI)** | **AER per 10,000 person-years (95%CI)** | **Obs/exp** | **SIR (95%CI)** | **AER per 10,000 person-years (95%CI)** |
| **Females** | **7. Gonadal and related tumours** | **23/2** | **10.8  (6.9, 16.2)** | **7.3  (4.3, 11.3)** | **23/2** | **11.2  (7.1, 16.8)** | **7.3 (4.4, 11.3)** | **21/2** | **10.8  (6.7, 16.5)** | **6.6  (3.9, 10.5)** | **21/2** | **11.5  (7.1, 17.5)** | **6.7  (3.9, 10.6)** | **21/2** | **11.7  (7.2, 17.9)** | **6.7  (3.9, 10.6)** | **16/1** | **16.4  (9.4, 26.6)** | **5.2  (2.9, 8.7)** | **NA** | **NA** | **NA** | **NA** | **NA** | **NA** |
|  | 7.2 Ovary | 22/2 | 11.3  (7.1, 17.1) | 7.7  (4.6, 12.1) | 22/2 | 11.7  (7.3, 17.7) | 7.8  (4.6, 12.1) | 20/2 | 11.2  (6.9, 17.3) | 7.0  (4.0, 11.2) | 20/2 | 11.9  (7.3, 18.4) | 7.1  (4.1, 11.3) | 20/2 | 12.1  (7.4, 18.7) | 7.1  (4.1, 11.3) | 16/1 | 17.8  (10.2, 28.9) | 5.8  (3.2, 9.7) | NA | NA | NA | NA | NA | NA |
|  | 7.2.2 Non-germ cell | 20/2 | 11.5  (7.0, 17.8) | 8.4  (4.8, 13.4) | 20/2 | 11.9  (7.3, 18.4) | 8.4  (4.8, 13.4) | 20/2 | 12.5  (7.6, 19.3) | 8.5  (4.9, 13.5) | 20/2 | 13.2  (8.1, 20.4) | 8.5  (4.9, 13.5) | 20/1 | 13.5  (8.2, 20.8) | 8.5  (4.9, 13.5) | 16/1 | 19.7  (11.3, 32.0) | 7.0  (3.8, 11.6) | NA | NA | NA | NA | NA | NA |
|  | 7.2.2.1 Carcinoma | 20/2 | 12.0  (7.3, 18.5) | 8.8  (5.0, 14.0) | 20/2 | 12.4  (7.6, 19.1) | 8.8  (5.1, 14.0) | 20/2 | 13.0  (8.0, 20.1) | 8.8  (5.1, 14.0) | 20/1 | 13.8  (8.4, 21.3) | 8.9  (5.1, 14.1) | 20/1 | 14.1  (8.6, 21.7) | 8.9  (5.2, 14.1) | 16/1 | 20.6  (11.8, 33.4) | 7.3  (4.0, 12.1) | NA | NA | NA | NA | NA | NA |
|  | 7.2.2.1.1 Adenocarcinoma | 20/2 | 12.2  (7.5, 18.9) | 9.0  (5.2, 14.3) | 20/2 | 12.6  (7.7, 19.5) | 9.0  (5.2, 14.3) | 20/2 | 13.2  (8.1, 20.5) | 9.0  (5.2, 14.3) | 20/1 | 14.0  (8.6, 21.7) | 9.1  (5.3, 14.4) | 20/1 | 14.3  (8.7, 22.1) | 9.1  (5.3, 14.4) | 16/1 | 20.9  (12.0, 34.0) | 7.4  (4.1, 12.3) | NA | NA | NA | NA | NA | NA |
|  | 7.2.2.1.1.2 Cystadenocarcinoma | 17/1 | 13.1  (7.6, 21.0) | 9.5  (5.2, 15.7) | 17/1 | 13.5  (7.9, 21.6) | 9.5  (5.2, 15.7) | 17/1 | 14.2  (8.3, 22.8) | 9.6  (5.3, 15.8) | 17/1 | 15.1  (8.8, 24.1) | 9.6  (5.3, 15.8) | 17/1 | 15.4  (9.0, 24.6) | 9.6  (5.3, 15.8) | 15/1 | 24.9  (13.9, 41.1) | 8.7 (4.7, 14.6) | NA | NA | NA | NA | NA | NA |
|  | **9. Carcinomas** | **94/28** | **3.3  (2.7, 4.1)** | **1.9  (1.3, 2.5)** | **94/27** | **3.4  (2.8, 4.2)** | **1.9  (1.4, 2.5)** | **94/26** | **3.6  (2.9, 4.4)** | **1.9  (1.4, 2.5)** | **94/25** | **3.8  (3.1, 4.6)** | **2.0  (1.4, 2.6)** | **94/24** | **3.9  (3.1, 4.7)** | **2.0  (1.5, 2.6)** | **62/13** | **4.7  (3.6, 6.0)** | **1.4  (1.0, 1.9)** | **10/4** | **2.8  (1.3, 5.1)** | **0.2  (0.0, 0.4)** | **14/2** | **7.8  (4.3, 13.1)** | **0.3  (0.2, 0.6)** |
|  | 9.6 Carcinoma of breast | 65/15 | 4.2 (3.3, 5.4) | 2.7  (1.9, 3.6) | 65/15 | 4.4  (3.4, 5.6) | 2.7  (1.9, 3.7) | 65/14 | 4.5  (3.5, 5.8) | 2.7  (1.9, 3.7) | 65/14 | 4.8  (3.7, 6.1) | 2.8  (2.0, 3.7) | 65/13 | 4.9  (3.8, 6.2) | 2.8  (2.0, 3.7) | 45/7 | 6.2  (4.5, 8.3) | 2.0  (1.4, 2.9) | NA | NA | NA | 11/1 | 10.9  (5.5, 19.6) | 0.5  (0.2, 1.0) |
|  | 9.6.1 Breast, infiltrating duct | 49/12 | 4.1  (3.0, 5.4) | 2.5  (1.7, 3.6) | 49/12 | 4.2  (3.1, 5.6) | 2.5  (1.7, 3.6) | 49/11 | 4.4  (3.3, 5.8) | 2.6  (1.7, 3.6) | 49/10 | 4.7  (3.5, 6.2) | 2.6  (1.7, 3.7) | 49/10 | 4.8  (3.5, 6.3) | 2.6  (1.8, 3.7) | 34/6 | 6.1  (4.2, 8.5) | 1.9  (1.2, 2.8) | NA | NA | NA | NA | NA | NA |
|  | 9.7 Carcinoma of genital sites excluding ovary and testis | 16/7 | 2.3  (1.3, 3.7) | 1.1  (0.3, 2.3) | 16/7 | 2.4 (1.4, 3.9) | 1.1  (0.3, 2.3) | 16/6 | 2.5  (1.4, 4.0) | 1.2  (0.3, 2.4) | 16/6 | 2.6  (1.5, 4.3) | 1.2  (0.4, 2.4) | 16/6 | 2.7  (1.5, 4.3) | 1.2  (0.4, 2.4) | 10/3 | 3.1  (1.5, 5.6) | 0.8  (0.2, 1.8) | NA | NA | NA | NA | NA | NA |
|  | 9.7.1 Carcinoma of uterine cervix | 15/6 | 2.4  (1.3, 3.9) | 1.2  (0.3, 2.4) | 15/6 | 2.5 (1.4, 4.1) | 1.2  (0.3, 2.5) | 15/6 | 2.6  (1.4, 4.2) | 1.2  (0.3, 2.5) | 15/5 | 2.7  (1.5, 4.5) | 1.3  (0.4, 2.5) | 15/5 | 2.8  (1.6, 4.6) | 1.3  (0.4, 2.6) | 10/3 | 3.4  (1.6, 6.3) | 0.9  (0.2, 2.0) | NA | NA | NA | NA | NA | NA |

Abbreviation: NA=Not Applicable.

**Supplementary Table S9A.** Standardised incidence ratios (SIRs) and absolute excess risks (AERs) of second primary melanomas after each distinct first primary cancer type among six-month adolescent and young adult (AYA, aged 15-39 years) cancer survivors compared to the general population in the Netherlands. Second primary cancer types are grouped according to the AYA-specific classification scheme developed by Barr and colleagues (2020). First and second cancer combinations with less than n=10 observed second cancers were excluded from the analyses. This Table presents outcomes from the main analysis.

|  | **Second primary cancers** | **8. Melanoma, malignant** | | | **8.1 Superficial spreading/low cumulative sun damage melanoma** | | | **8.2 Nodular melanoma** | | | **8.3 Other malignant** | | |
| --- | --- | --- | --- | --- | --- | --- | --- | --- | --- | --- | --- | --- | --- |
|  | **First primary cancers** | **Obs/exp** | **SIR (95%CI)** | **AER per 10,000 person-years (95%CI)** | **Obs/exp** | **SIR (95%CI)** | **AER per 10,000 person-years (95%CI)** | **Obs/exp** | **SIR (95%CI)** | **AER per 10,000 person-years (95%CI)** | **Obs/exp** | **SIR (95%CI)** | **AER per 10,000 person-years (95%CI)** |
| **Males** | **2. Lymphomas** | **27/15** | **1.8 (1.2, 2.7)** | **1.8 (0.4, 3.5)** | **23/11** | **2.0 (1.3, 3.0)** | **1.6 (0.5, 3.3)** | **NA** | **NA** | **NA** | **NA** | **NA** | **NA** |
|  | 2.1 Non-Hodgkin lymphomas | 10/6 | 1.6 (0.8, 3.0) | 1.4 (-0.5, 4.4) | NA | NA | NA | NA | NA | NA | NA | NA | NA |
|  | 2.2 Hodgkin lymphoma | 15/8 | 2.0 (1.1, 3.3) | 1.9 (0.2, 4.4) | 13/6 | 2.2 (1.2, 3.8) | 1.8 (0.3, 4.2) | NA | NA | NA | NA | NA | NA |
|  | 2.2.2 Hodgkin classic, other | 15/7 | 2.1 (1.2, 3.5) | 2.2 (0.4, 4.9) | 13/5 | 2.4 (1.3, 4.1) | 2.1 (0.4, 4.6) | NA | NA | NA | NA | NA | NA |
|  | **7. Gonadal and related tumours** | **45/30** | **1.5 (1.1, 2.0)** | **1.1 (0.2, 2.2)** | **27/23** | **1.2 (0.8, 1.7)** | **0.3 (-0.4, 1.1)** | **NA** | **NA** | **NA** | **13/5** | **2.9 (1.5, 4.9)** | **0.6 (0.2, 1.3)** |
|  | 7.1 Testis | 44/29 | 1.5 (1.1, 2.0) | 1.1 (0.2, 2.2) | 26/23 | 1.1 (0.7, 1.7) | 0.2 (-0.4, 1.1) | NA | NA | NA | 13/4 | 2.9 (1.6, 5.0) | 0.6 (0.2, 1.3) |
|  | 7.1.1 Germ cell and trophoblastic | 44/29 | 1.5 (1.1, 2.0) | 1.1 (0.2, 2.2) | 26/23 | 1.1 (0.7, 1.7) | 0.2 (-0.4, 1.1) | NA | NA | NA | 13/4 | 2.9 (1.6, 5.0) | 0.6 (0.2, 1.3) |
|  | 7.1.1.1 Seminoma | 22/16 | 1.4 (0.9, 2.1) | 1.0 (-0.3, 2.8) | 14/12 | 1.1 (0.6, 1.9) | 0.3 (-0.7, 1.8) | NA | NA | NA | NA | NA | NA |
|  | 7.1.1.4 Teratoma | 10/5 | 2.2 (1.1, 4.0) | 2.4 (0.1, 6.0) | NA | NA | NA | NA | NA | NA | NA | NA | NA |
|  | **9. Carcinomas** | **28/17** | **1.6 (1.1, 2.4)** | **1.7 (0.2, 3.7)** | **21/13** | **1.6 (1.0, 2.4)** | **1.2 (0.0, 2.9)** | **NA** | **NA** | **NA** | **NA** | **NA** | **NA** |
| **Females** | **2. Lymphomas** | **25/17** | **1.5 (1.0, 2.2)** | **1.5 (-0.1, 3.8)** | **19/14** | **1.4 (0.8, 2.1)** | **1.0 (-0.5, 3.0)** | **NA** | **NA** | **NA** | **NA** | **NA** | **NA** |
|  | 2.2 Hodgkin lymphoma | 18/10 | 1.9 (1.1, 3.0) | 2.5 (0.3, 5.7) | 12/8 | 1.5 (0.8, 2.6) | 1.2 (-0.5, 3.9) | NA | NA | NA | NA | NA | NA |
|  | 2.2.2 Hodgkin classic, other | 18/9 | 1.9 (1.1, 3.0) | 2.7 (0.4, 5.9) | 12/8 | 1.5 (0.8, 2.7) | 1.3 (-0.5, 4.1) | NA | NA | NA | NA | NA | NA |
|  | **4. Sarcomas** | **17/8** | **2.0 (1.2, 3.2)** | **3.5 (0.6, 7.7)** | **13/7** | **1.9 (1.0, 3.2)** | **2.5 (0.0, 6.3)** | **NA** | **NA** | **NA** | **NA** | **NA** | **NA** |
|  | **7. Gonadal and related tumours** | **16/12** | **1.4 (0.8, 2.2)** | **1.5 (-0.8, 5.0)** | **12/9** | **1.3 (0.7, 2.2)** | **0.9 (-1.1, 4.0)** | **NA** | **NA** | **NA** | **NA** | **NA** | **NA** |
|  | 7.2 Ovary | 15/11 | 1.4 (0.8, 2.3) | 1.7 (-0.8, 5.4) | 11/9 | 1.3 (0.6, 2.3) | 0.9 (-1.2, 4.3) | NA | NA | NA | NA | NA | NA |
|  | 7.2.2 Non-germ cell | 14/9 | 1.5 (0.8, 2.5) | 2.1 (-0.8, 6.5) | 10/8 | 1.3 (0.6, 2.4) | 1.1 (-1.3, 4.9) | NA | NA | NA | NA | NA | NA |
|  | 7.2.2.1 Carcinoma | 14/9 | 1.6 (0.9, 2.6) | 2.4 (-0.6, 6.9) | 10/7 | 1.4 (0.7, 2.5) | 1.3 (-1.2, 5.3) | NA | NA | NA | NA | NA | NA |
|  | 7.2.2.1.1 Adenocarcinoma | 13/9 | 1.5 (0.8, 2.5) | 2.0 (-0.9, 6.5) | 10/7 | 1.4 (0.7, 2.6) | 1.4 (-1.1, 5.4) | NA | NA | NA | NA | NA | NA |
|  | 7.2.2.1.1.2 Cystadenocarcinoma | 12/7 | 1.7 (0.9, 3.0) | 3.0 (-0.5, 8.4) | NA | NA | NA | NA | NA | NA | NA | NA | NA |
|  | **9. Carcinomas** | **146/157** | **0.9 (0.8, 1.1)** | **-0.3 (-0.9, 0.4)** | **102/128** | **0.8 (0.7, 1.0)** | **-0.7 (-1.2, -0.1)** | **18/8** | **2.1 (1.3, 3.4)** | **0.3 (0.1, 0.6)** | **26/21** | **1.3 (0.8, 1.8)** | **0.1 (-0.1, 0.5)** |
|  | 9.6 Carcinoma of breast | 93/86 | 1.1 (0.9, 1.3) | 0.3 (-0.6, 1.4) | 66/70 | 0.9 (0.7, 1.2) | -0.2 (-1.0, 0.7) | 11/5 | 2.3 (1.2, 4.2) | 0.3 (0.0, 0.8) | 16/12 | 1.4 (0.8, 2.2) | 0.2 (-0.1, 0.7) |
|  | 9.6.1 Breast, infiltrating duct | 74/68 | 1.1 (0.9, 1.4) | 0.4 (-0.6, 1.6) | 56/55 | 1.0 (0.8, 1.3) | 0.1 (-0.8, 1.2) | NA | NA | NA | 10/9 | 1.1 (0.5, 2.0) | 0.1 (-0.3, 0.6) |
|  | 9.7 Carcinoma of genital sites excluding ovary and testis | 29/37 | 0.8 (0.5, 1.1) | -0.9 (-2.1, 0.6) | 18/30 | 0.6 (0.4, 0.9) | -1.5 (-2.3, -0.2) | NA | NA | NA | NA | NA | NA |
|  | 9.7.1 Carcinoma of uterine cervix | 29/33 | 0.9 (0.6, 1.2) | -0.6 (-1.9, 1.1) | 18/27 | 0.7 (0.4, 1.0) | -1.2 (-2.2, 0.2) | NA | NA | NA | NA | NA | NA |
|  | 9.7.1.1 Cervix, squamous | 17/24 | 0.7 (0.4, 1.2) | -1.2 (-2.6, 0.7) | 11/19 | 0.6 (0.3, 1.0) | -1.6 (-2.6, 0.1) | NA | NA | NA | NA | NA | NA |

Abbreviation: NA=Not Applicable.

**Supplementary Table S9B.** Standardised incidence ratios (SIRs) and absolute excess risks (AERs) of second primary melanomas after each distinct first primary cancer type among six-month adolescent and young adult (AYA, aged 15-39 years) cancer survivors compared to the general population in the Netherlands. Second primary cancer types are grouped according to the AYA-specific classification scheme developed by Barr and colleagues (2020). First and second cancer combinations with less than n=10 observed second cancers were excluded from the analyses. This Table presents outcomes from the sensitivity analysis.

|  | **Second primary cancers** | **8. Melanoma, malignant** | | | **8.1 Superficial spreading/low cumulative sun damage melanoma** | | | **8.2 Nodular melanoma** | | | **8.3 Other malignant** | | |
| --- | --- | --- | --- | --- | --- | --- | --- | --- | --- | --- | --- | --- | --- |
|  | **First primary cancers** | **Obs/exp** | **SIR (95%CI)** | **AER per 10,000 person-years (95%CI)** | **Obs/exp** | **SIR (95%CI)** | **AER per 10,000 person-years (95%CI)** | **Obs/exp** | **SIR (95%CI)** | **AER per 10,000 person-years (95%CI)** | **Obs/exp** | **SIR (95%CI)** | **AER per 10,000 person-years (95%CI)** |
| **Males** | **2. Lymphomas** | **27/15** | **1.8 (1.2, 2.7)** | **1.8 (0.4, 3.5)** | **23/11** | **2.0 (1.3, 3.0)** | **1.7 (0.5, 3.3)** | **NA** | **NA** | **NA** | **NA** | **NA** | **NA** |
|  | 2.1 Non-Hodgkin lymphomas | 10/6 | 1.6 (0.8, 3.0) | 1.4 (-0.5, 4.4) | NA | NA | NA | NA | NA | NA | NA | NA | NA |
|  | 2.2 Hodgkin lymphoma | 15/7 | 2.0 (1.1, 3.3) | 1.9 (0.2, 4.4) | 13/6 | 2.2 (1.2, 3.8) | 1.8 (0.3, 4.2) | NA | NA | NA | NA | NA | NA |
|  | 2.2.2 Hodgkin classic, other | 15/7 | 2.1 (1.2, 3.5) | 2.2 (0.4, 4.9) | 13/5 | 2.4 (1.3, 4.1) | 2.1 (0.4, 4.6) | NA | NA | NA | NA | NA | NA |
|  | **7. Gonadal and related tumours** | **42/29** | **1.4 (1.0, 1.9)** | **0.9 (0.1, 2.0)** | **25/23** | **1.1 (0.7, 1.6)** | **0.2 (-0.5, 1.0)** | **NA** | **NA** | **NA** | **12/4** | **2.7 (1.4, 4.7)** | **0.5 (0.1, 1.2)** |
|  | 7.1 Testis | 41/28 | 1.4 (1.0, 2.0) | 0.9 (0.1, 2.0) | 24/22 | 1.1 (0.7, 1.6) | 0.1 (-0.5, 1.0) | NA | NA | NA | 12/4 | 2.8 (1.4, 4.8) | 0.6 (0.1, 1.2) |
|  | 7.1.1 Germ cell and trophoblastic | 41/28 | 1.4 (1.0, 2.0) | 0.9 (0.1, 2.0) | 24/22 | 1.1 (0.7, 1.6) | 0.1 (-0.5, 1.0) | NA | NA | NA | 12/4 | 2.8 (1.4, 4.8) | 0.6 (0.1, 1.2) |
|  | 7.1.1.1 Seminoma | 20/15 | 1.3 (0.8, 2.0) | 0.8 (-0.5, 2.5) | 13/12 | 1.1 (0.6, 1.9) | 0.2 (-0.8, 1.7) | NA | NA | NA | NA | NA | NA |
|  | 7.1.1.4 Teratoma | 10/4 | 2.2 (1.1, 4.1) | 2.5 (0.2, 6.2) | NA | NA | NA | NA | NA | NA | NA | NA | NA |
|  | **8. Melanoma, malignant** | **211/16** | **13.3 (11.5, 15.2)** | **30.3 (26.0, 35.0)** | **168/12** | **13.6 (11.6, 15.8)** | **24.2 (20.4, 28.4)** | **21/1** | **19.6 (12.1, 29.9)** | **3.1 (1.9, 4.8)** | **22/2** | **9.1 (5.7, 13.7)** | **3.0 (1.8, 4.8)** |
|  | 8.1 Superficial spreading/low cumulative sun damage melanoma | 140/10 | 14.6 (12.2, 17.2) | 32.9 (27.3, 39.3) | 115/8 | 15.3 (12.6, 18.3) | 27.1 (22.1, 32.9) | 14/1 | 22.0 (12.0, 36.8) | 3.4 (1.8, 5.8) | 11/1 | 7.6 (3.8, 13.5) | 2.4 (1.0, 4.6) |
|  | 8.2 Nodular melanoma | 26/2 | 15.5 (10.1, 22.7) | 35.4 (22.3, 53.0) | 14/1 | 10.8 (5.9, 18.0) | 18.5 (9.2, 32.3) | NA | NA | NA | NA | NA | NA |
|  | 8.3 Other malignant | 45/5 | 9.8 (7.1, 13.1) | 22.5 (15.8, 31.0) | 39/4 | 10.9 (7.8, 14.9) | 19.8 (13.5, 27.8) | NA | NA | NA | NA | NA | NA |
|  | **9. Carcinomas** | **29/17** | **1.7 (1.2, 2.5)** | **1.9 (0.4, 3.9)** | **22/13** | **1.7 (1.1, 2.6)** | **1.4 (0.1, 3.2)** | **NA** | **NA** | **NA** | **NA** | **NA** | **NA** |
| **Females** | **2. Lymphomas** | **25/17** | **1.5 (1.0, 2.2)** | **1.5 (-0.1, 3.8)** | **19/14** | **1.4 (0.8, 2.1)** | **1.0 (-0.5, 3.0)** | **NA** | **NA** | **NA** | **NA** | **NA** | **NA** |
|  | 2.2 Hodgkin lymphoma | 18/10 | 1.9 (1.1, 3.0) | 2.5 (0.3, 5.7) | 12/8 | 1.5 (0.8, 2.6) | 1.2 (-0.5, 3.9) | NA | NA | NA | NA | NA | NA |
|  | 2.2.2 Hodgkin classic, other | 18/9 | 1.9 (1.1, 3.0) | 2.7 (0.4, 5.9) | 12/8 | 1.5 (0.8, 2.7) | 1.3 (-0.5, 4.1) | NA | NA | NA | NA | NA | NA |
|  | **4. Sarcomas** | **17/8** | **2.0 (1.2, 3.2)** | **3.5 (0.6, 7.7)** | **13/7** | **1.9 (1.0, 3.2)** | **2.5 (0.0, 6.3)** | **NA** | **NA** | **NA** | **NA** | **NA** | **NA** |
|  | **7. Gonadal and related tumours** | **15/11** | **1.3 (0.7, 2.2)** | **1.2 (-1.1, 4.6)** | **11/9** | **1.2 (0.6, 2.1)** | **0.6 (-1.4, 3.6)** | **NA** | **NA** | **NA** | **NA** | **NA** | **NA** |
|  | 7.2 Ovary | 14/10 | 1.3 (0.7, 2.2) | 1.4 (-1.1, 5.0) | 10/9 | 1.2 (0.6, 2.2) | 0.6 (-1.4, 3.8) | NA | NA | NA | NA | NA | NA |
|  | 7.2.2 Non-germ cell | 13/9 | 1.4 (0.7, 2.4) | 1.7 (-1.1, 6.0) | NA | NA | NA | NA | NA | NA | NA | NA | NA |
|  | 7.2.2.1 Carcinoma | 13/9 | 1.5 (0.8, 2.5) | 2.0 (-0.9, 6.4) | NA | NA | NA | NA | NA | NA | NA | NA | NA |
|  | 7.2.2.1.1 Adenocarcinoma | 12/9 | 1.4 (0.7, 2.4) | 1.6 (-1.2, 6.0) | NA | NA | NA | NA | NA | NA | NA | NA | NA |
|  | 7.2.2.1.1.2 Cystadenocarcinoma | 11/7 | 1.6 (0.8, 2.8) | 2.4 (-0.9, 7.7) | NA | NA | NA | NA | NA | NA | NA | NA | NA |
|  | **8. Melanoma, malignant** | **413/51** | **8.1 (7.4, 9.0)** | **27.8 (24.8, 31.0)** | **331/42** | **7.9 (7.1, 8.8)** | **22.2 (19.5, 25.1)** | **20/3** | **7.7 (4.7, 11.9)** | **1.3 (0.7, 2.2)** | **62/6** | **9.6 (7.4, 12.4)** | **4.3 (3.2, 5.6)** |
|  | 8.1 Superficial spreading/low cumulative sun damage melanoma | 286/33 | 8.7 (7.7, 9.7) | 29.5 (25.8, 33.6) | 230/27 | 8.5 (7.4, 9.6) | 23.7 (20.3, 27.4) | 14/2 | 8.4 (4.6, 14.0) | 1.4 (0.7, 2.5) | 42/4 | 10.1 (7.3, 13.7) | 4.4 (3.0, 6.1) |
|  | 8.2 Nodular melanoma | 40/4 | 9.8 (7.0, 13.4) | 32.9 (22.4, 46.1) | 33/3 | 9.9 (6.8, 13.9) | 27.1 (17.7, 39.3) | NA | NA | NA | NA | NA | NA |
|  | 8.3 Other malignant | 87/14 | 6.4 (5.1, 7.9) | 21.7 (16.6, 27.7) | 68/11 | 6.1 (4.8, 7.8) | 16.8 (12.3, 22.2) | NA | NA | NA | 15/2 | 8.5 (4.8, 14.1) | 3.9 (2.0, 6.8) |
|  | **9. Carcinomas** | **142/152** | **0.9 (0.8, 1.1)** | **-0.3 (-0.9, 0.4)** | **99/123** | **0.8 (0.7, 1.0)** | **-0.7 (-1.2, -0.1)** | **17/8** | **2.1 (1.2, 3.3)** | **0.2 (0.0, 0.5)** | **26/20** | **1.3 (0.8, 1.9)** | **0.2 (-0.1, 0.5)** |
|  | 9.6 Carcinoma of breast | 89/81 | 1.1 (0.9, 1.3) | 0.4 (-0.5, 1.5) | 63/66 | 1.0 (0.7, 1.2) | -0.2 (-0.9, 0.8) | 10/4 | 2.2 (1.1, 4.1) | 0.3 (0.0, 0.7) | 16/11 | 1.4 (0.8, 2.3) | 0.3 (-0.1, 0.8) |
|  | 9.6.1 Breast, infiltrating duct | 71/64 | 1.1 (0.9, 1.4) | 0.5 (-0.6, 1.7) | 54/52 | 1.0 (0.8, 1.4) | 0.1 (-0.8, 1.3) | NA | NA | NA | 10/9 | 1.2 (0.6, 2.1) | 0.1 (-0.3, 0.7) |
|  | 9.7 Carcinoma of genital sites excluding ovary and testis | 29/37 | 0.8 (0.5, 1.1) | -0.9 (-2.1, 0.6) | 18/30 | 0.6 (0.4, 0.9) | -1.5 (-2.3, -0.2) | NA | NA | NA | NA | NA | NA |
|  | 9.7.1 Carcinoma of uterine cervix | 29/33 | 0.9 (0.6, 1.2) | -0.6 (-1.9, 1.1) | 18/27 | 0.7 (0.4, 1.0) | -1.2 (-2.2, 0.2) | NA | NA | NA | NA | NA | NA |
|  | 9.7.1.1 Cervix, squamous | 17/24 | 0.7 (0.4, 1.2) | -1.2 (-2.6, 0.7) | 11/19 | 0.6 (0.3, 1.0) | -1.6 (-2.6, 0.1) | NA | NA | NA | NA | NA | NA |

Abbreviation: NA=Not Applicable.

**Supplementary Table S10A.** Standardised incidence ratios (SIRs) and absolute excess risks (AERs) of second primary carcinomas after each distinct first primary cancer type among six-month adolescent and young adult (AYA, aged 15-39 years) cancer survivors compared to the general population in the Netherlands. Second primary cancer types are grouped according to the AYA-specific classification scheme developed by Barr and colleagues (2020). First and second cancer combinations with less than n=10 observed second cancers were excluded from the analyses. This Table presents outcomes from the main analysis.

|  | **Second primary cancers** | **9. Carcinomas** | | |
| --- | --- | --- | --- | --- |
|  | **First primary cancers** | **Obs/exp** | **SIR (95%CI)** | **AER per 10,000 person-years (95%CI)** |
| **Males** | **1. Leukaemia’s and related disorders** | **42/14** | **3.0 (2.1, 4.0)** | **15.2 (8.8, 23.2)** |
|  | 1.2 Acute myeloid leukaemia | 13/3 | 4.0 (2.1, 6.9) | 21.6 (8.2, 42.0) |
|  | **2. Lymphomas** | **197/57** | **3.4 (3.0, 4.0)** | **19.9 (16.1, 24.1)** |
|  | 2.1 Non-Hodgkin lymphomas | 77/25 | 3.1 (2.4, 3.8) | 18.9 (13.0, 25.8) |
|  | 2.1.3 Diffuse large B-cell (DLBCL) | 32/11 | 3.0 (2.1, 4.3) | 19.3 (10.2, 31.2) |
|  | 2.1.6 Follicular | 20/5 | 3.7 (2.3, 5.7) | 30.2 (14.1, 52.7) |
|  | 2.1.9 Other non-Hodgkin lymphoma NOS | 10/2 | 4.3 (2.1, 8.0) | 38.0. (12.3, 79.4) |
|  | 2.2 Hodgkin lymphoma | 109/28 | 3.9 (3.2, 4.8) | 20.9 (15.9, 26.7) |
|  | 2.2.2 Hodgkin classic, other | 104/26 | 4.0 (3.3, 4.9) | 21.5 (16.3, 27.6) |
|  | **3. CNS and other intracranial and intraspinal neoplasms** | **17/10** | **1.6 (0.9, 2.6)** | **3.6 (-0.3, 9.4)** |
|  | 3.1 Astroglial and related neoplasms | 15/9 | 1.6 (0.9, 2.6) | 3.6 (-0.6, 9.9) |
|  | **4. Sarcomas** | **44/21** | **2.1 (1.5, 2.8)** | **9.0. (4.3, 15.0)** |
|  | 4.4 Fibromatous neoplasms | 16/8 | 2.0 (1.2, 3.3) | 10.6 (1.7, 23.5) |
|  | 4.4.3 Other fibromatous neoplasms | 13/7 | 2.0 (1.1, 3.4) | 10.2 (0.6, 24.8) |
|  | **5. Blood and lymphatic vessel tumours** | **18/5** | **3.7 (2.2, 5.9)** | **27.6 (12.2, 49.6)** |
|  | 5.2 Malignant blood and lymphatic vessel tumours, all sites | 18/5 | 3.7 (2.2, 5.9) | 27.6 (12.2, 49.6) |
|  | 5.2.1 Kaposi sarcoma | 17/5 | 3.7 (2.1, 5.8) | 28.3 (12.0, 51.8) |
|  | **7. Gonadal and related tumours** | **184/112** | **1.6 (1.4, 1.9)** | **5.1 (3.3, 7.1)** |
|  | 7.1 Testis | 176/110 | 1.6 (1.4, 1.9) | 4.8 (3.0, 6.8) |
|  | 7.1.1 Germ cell and trophoblastic | 174/110 | 1.6 (1.4, 1.8) | 4.7 (2.9, 6.7) |
|  | 7.1.1.1 Seminoma | 95/64 | 1.5 (1.2, 1.8) | 4.9 (2.0, 8.2) |
|  | 7.1.1.2 Embryonal carcinoma | 23/13 | 1.7 (1.1, 2.6) | 4.8 (0.7, 10.4) |
|  | 7.1.1.4 Teratoma | 26/17 | 1.5 (1.0, 2.2) | 3.8 (-0.1, 9.1) |
|  | 7.1.1.5 Mixed germ cell | 20/10 | 2.0 (1.2, 3.1) | 5.1 (1.2, 10.6) |
|  | **8. Melanoma, malignant** | **92/69** | **1.3 (1.1, 1.6)** | **3.6 (0.8, 6.7)** |
|  | 8.1 Superficial spreading/low cumulative sun damage melanoma | 63/40 | 1.6 (1.2, 2.0) | 5.6 (2.0, 9.9) |
|  | 8.3 Other malignant | 26/21 | 1.2 (0.8, 1.8) | 2.9 (-2.1, 9.5) |
|  | **9. Carcinomas** | **279/78** | **3.6 (3.2, 4.0)** | **31.4 (26.4, 36.8)** |
|  | 9.1 Thyroid carcinoma | 15/10 | 1.5 (0.8, 2.4) | 4.7 (-1.9, 14.4) |
|  | 9.2 Other carcinoma of head and neck | 76/14 | 5.6 (4.4, 7.0) | 56.8 (42.1, 74.2) |
|  | 9.2.2 Oral cavity, lip, and pharynx | 51/8 | 6.6 (4.9, 8.7) | 76.2 (53.2, 104.4) |
|  | 9.2.2.1 Oral cavity, lip, and pharynx, squamous | 48/6 | 7.7 (5.7, 10.2) | 93.3 (65.2, 128.3) |
|  | 9.2.4 Other carcinoma of head and neck | 17/3 | 5.3 (3.1, 8.4) | 71.4 (34.6, 124.4) |
|  | 9.3 Carcinoma of gastrointestinal tract | 107/28 | 3.8 (3.1, 4.6) | 34.1 (25.8, 43.7) |
|  | 9.3.4 Carcinoma of colon | 62/15 | 4.2 (3.2, 5.4) | 37.8 (26.2, 51.9) |
|  | 9.3.4.2 Colon excluding appendix | 57/12 | 4.6 (3.5, 6.0) | 50.6 (34.9, 69.7) |
|  | 9.3.4.2.2 Colon excluding appendix, adenocarcinoma | 55/12 | 4.5 (3.4, 5.9) | 49.8 (34.0, 69.0) |
|  | 9.3.5 Carcinoma of rectum | 23/7 | 3.2 (2.1, 4.9) | 27.7 (13.0, 47.7) |
|  | 9.3.5.2 Rectum, adenocarcinoma | 21/6 | 3.3 (2.0, 5.0) | 28.5 (12.8, 50.3) |
|  | 9.4 Carcinoma of lung, bronchus, and trachea | 10/4 | 2.8 (1.3, 5.1) | 18.9 (3.5, 43.9) |
|  | 9.5 Carcinoma of skin (if collected) | 31/7 | 4.5 (3.1, 6.5) | 45.6 (26.8, 70.1) |
|  | 9.8 Carcinoma of urinary tract | 31/12 | 2.5 (1.7, 3.6) | 21.7 (10.2, 36.8) |
|  | 9.8.1 Carcinoma of kidney | 17/8 | 2.2 (1.3, 3.5) | 16.5 (3.9, 34.5) |
|  | 9.8.1.1 Kidney, adenocarcinoma | 17/8 | 2.2 (1.3, 3.6) | 16.7 (4.1, 34.9) |
|  | 9.8.1.1.1 Kidney, renal cell | 16/7 | 2.2 (1.3, 3.6) | 16.9 (3.8, 36.1) |
|  | 9.8.2 Carcinoma of bladder | 13/4 | 3.4 (1.8, 5.7) | 35.6 (11.9, 71.7) |
|  | 9.8.2.1 Urinary bladder, transitional cell carcinoma | 10/4 | 2.7 (1.3, 5.0) | 27.0 (4.8, 62.9) |
| **Females** | **1. Leukaemia’s and related disorders** | **45/30** | **1.5 (1.1, 2.0)** | **9.5 (1.9, 19.1)** |
|  | 1.2 Acute myeloid leukaemia | 20/11 | 1.8 (1.1, 2.8) | 16.6 (2.0, 37.0) |
|  | 1.2.2 Other acute myeloid leukaemia | 13/8 | 1.5 (0.8, 2.6) | 11.0 (-3.6, 33.2) |
|  | **2. Lymphomas** | **251/96** | **2.6 (2.3, 3.0)** | **29.4 (23.7, 35.7)** |
|  | 2.1 Non-Hodgkin lymphomas | 76/40 | 1.9 (1.5, 2.4) | 20.9 (11.6, 31.9) |
|  | 2.1.3 Diffuse large B-cell (DLBCL) | 26/14 | 1.8 (1.2, 2.6) | 17.7 (4.0, 36.1) |
|  | 2.1.5 Anaplastic T-cell and null-cell excluding NK/T-cell | 14/6 | 2.4 (1.3, 4.1) | 29.5 (6.9, 63.3) |
|  | 2.1.6 Follicular | 19/11 | 1.8 (1.1, 2.7) | 22.1 (1.7, 50.9) |
|  | 2.2 Hodgkin lymphoma | 164/50 | 3.3 (2.8, 3.8) | 34.3 (27.0, 42.5) |
|  | 2.2.2 Hodgkin classic, other | 162/49 | 3.3 (2.8, 3.8) | 34.9 (27.4, 43.2) |
|  | **3. CNS and other intracranial and intraspinal neoplasms** | **29/22** | **1.3 (0.9, 1.9)** | **5.1 (-2.0, 14.6)** |
|  | 3.1 Astroglial and related neoplasms | 26/19 | 1.4 (0.9, 2.0) | 5.8 (-2.0, 16.3) |
|  | 3.1.4 Other astrocytoma/astroglial neoplasms | 16/11 | 1.4 (0.8, 2.4) | 7.1 (-2.7, 21.5) |
|  | 3.1.4.3 Other astrocytoma/astroglial, invasive | 13/10 | 1.3 (0.7, 2.2) | 4.9 (-5.0, 19.8) |
|  | **4. Sarcomas** | **72/50** | **1.4 (1.1, 1.8)** | **8.8 (2.4, 16.5)** |
|  | 4.4 Fibromatous neoplasms | 16/19 | 0.9 (0.5, 1.4) | -3.4 (-12.0, 9.2) |
|  | 4.4.3 Other fibromatous neoplasms | 12/16 | 0.8 (0.4, 1.4) | -5.3 (-14.0, 8.2) |
|  | 4.5 Liposarcoma | 14/6 | 2.4 (1.3, 4.0) | 35.5 (7.8, 76.9) |
|  | **7. Gonadal and related tumours** | **90/75** | **1.2 (1.0, 1.5)** | **5.3 (-0.8, 12.5)** |
|  | 7.2 Ovary | 83/69 | 1.2 (1.0, 1.5) | 5.5 (-0.9, 13.2) |
|  | 7.2.1 Germ cell and trophoblastic | 10/7 | 1.5 (0.7, 2.8) | 8.0 (-4.4, 28.2) |
|  | 7.2.2 Non-germ cell | 73/62 | 1.2 (0.9, 1.5) | 5.1 (-2.1, 13.6) |
|  | 7.2.2.1 Carcinoma | 70/59 | 1.2 (0.9, 1.5) | 5.1 (-2.3, 13.8) |
|  | 7.2.2.1.1 Adenocarcinoma | 70/58 | 1.2 (0.9, 1.5) | 5.6 (-1.8, 14.6) |
|  | 7.2.2.1.1.2 Cystadenocarcinoma | 58/46 | 1.2 (0.9, 1.6) | 6.9 (-1.4, 17.1) |
|  | **8. Melanoma, malignant** | **401/324** | **1.2 (1.1, 1.4)** | **5.7 (2.9, 8.8)** |
|  | 8.1 Superficial spreading/low cumulative sun damage melanoma | 257/207 | 1.2 (1.1, 1.4) | 5.6 (2.2, 9.4) |
|  | 8.2 Nodular melanoma | 39/26 | 1.5 (1.1, 2.1) | 11.4 (1.5, 23.9) |
|  | 8.3 Other malignant | 105/90 | 1.2 (0.9, 1.4) | 4.2 (-1.3, 10.6) |
|  | **9. Carcinomas** | **1,168/1,046** | **1.1 (1.1, 1.2)** | **3.4 (1.5, 5.3)** |
|  | 9.1 Thyroid carcinoma | 90/74 | 1.2 (1.0, 1.5) | 4.8 (-0.6, 11.2) |
|  | 9.1.3 Papillary | 37/37 | 1.0 (0.7, 1.4) | 0.0 (-6.5, 8.3) |
|  | 9.1.4 Follicular | 17/12 | 1.4 (0.8, 2.2) | 8.7 (-4.8, 28.1) |
|  | 9.1.5 Papillary with follicular variant | 26/20 | 1.3 (0.8, 1.9) | 7.4 (-4.0, 22.8) |
|  | 9.2 Other carcinoma of head and neck | 63/21 | 3.0 (2.3, 3.8) | 48.8 (31.8, 69.3) |
|  | 9.2.2 Oral cavity, lip, and pharynx | 38/10 | 3.9 (2.8, 5.4) | 76.7 (46.6, 115.0) |
|  | 9.2.2.1 Oral cavity, lip, and pharynx, squamous | 34/7 | 5.0 (3.5, 7.0) | 112.1 (69.1, 167.8) |
|  | 9.2.3 Salivary gland | 11/6 | 1.7 (0.9, 3.1) | 15.9 (-2.9, 45.4) |
|  | 9.2.4 Other carcinoma of head and neck | 10/3 | 3.3 (1.6, 6.1) | 67.5 (17.2, 148.8) |
|  | 9.3 Carcinoma of gastrointestinal tract | 125/63 | 2.0 (1.7, 2.4) | 24.3 (16.1, 33.7) |
|  | 9.3.4 Carcinoma of colon | 77/36 | 2.1 (1.7, 2.6) | 26.6 (16.0, 39.2) |
|  | 9.3.4.1 Appendix | 14/13 | 1.1 (0.6, 1.8) | 1.8 (-7.2, 15.4) |
|  | 9.3.4.1.1 NET | 13/11 | 1.1 (0.6, 2.0) | 2.6 (-6.8, 16.9) |
|  | 9.3.4.2 Colon excluding appendix | 63/24 | 2.7 (2.0, 3.4) | 47.7 (30.0, 69.1) |
|  | 9.3.4.2.2 Colon excluding appendix, adenocarcinoma | 61/23 | 2.6 (2.0, 3.4) | 47.2 (29.3, 68.9) |
|  | 9.3.5 Carcinoma of rectum | 23/16 | 1.5 (0.9, 2.2) | 13.2 (-1.8, 33.7) |
|  | 9.3.5.2 Rectum, adenocarcinoma | 18/14 | 1.3 (0.8, 2.1) | 8.9 (-6.4, 30.7) |
|  | 9.4 Carcinoma of lung, bronchus, and trachea | 27/15 | 1.8 (1.2, 2.7) | 23.0 (5.9, 45.8) |
|  | 9.4.2 Non-small cell carcinoma | 27/14 | 1.9 (1.2, 2.8) | 24.3 (6.7, 47.8) |
|  | 9.4.2.1 Non-small cell, adenocarcinoma | 13/3 | 3.8 (2.0, 6.5) | 74.8 (27.3, 147.1) |
|  | 9.5 Carcinoma of skin (if collected) | 41/18 | 2.3 (1.7, 3.1) | 37.5 (18.8, 61.2) |
|  | 9.6 Carcinoma of breast | 429/585 | 0.7 (0.7, 0.8) | -8.0 (-10.1, -5.8) |
|  | 9.6.1 Breast, infiltrating duct | 333/453 | 0.7 (0.7, 0.8) | -7.8 (-10.0, -5.3) |
|  | 9.6.2 Breast, adenocarcinoma | 49/57 | 0.9 (0.6, 1.1) | -4.7 (-12.0, 4.4) |
|  | 9.6.3 Breast, lobular | 19/31 | 0.6 (0.4, 1.0) | -12.9 (-20.9, -1.6) |
|  | 9.6.5 Breast, medullary | 10/20 | 0.5 (0.2, 0.9) | -16.3 (-24.8, -2.6) |
|  | 9.7 Carcinoma of genital sites excluding ovary and testis | 352/250 | 1.4 (1.3, 1.6) | 12.3 (8.0, 17.0) |
|  | 9.7.1 Carcinoma of uterine cervix | 306/225 | 1.4 (1.2, 1.5) | 10.7 (6.3, 15.5) |
|  | 9.7.1.1 Cervix, squamous | 207/159 | 1.3 (1.1, 1.5) | 8.9 (3.8, 14.6) |
|  | 9.7.1.2 Cervix, adenosquamous | 12/7 | 1.6 (0.8, 2.9) | 18.5 (-4.5, 54.1) |
|  | 9.7.1.3 Cervix, adenocarcinoma | 66/40 | 1.6 (1.3, 2.1) | 17.8 (7.4, 30.3) |
|  | 9.7.1.4 Cervix, other | 21/18 | 1.2 (0.7, 1.8) | 5.5 (-9.7, 26.6) |
|  | 9.7.2 Corpus uteri | 16/13 | 1.3 (0.7, 2.0) | 8.7 (-9.8, 35.5) |
|  | 9.7.2.1 Corpus uteri, adenocarcinoma | 13/10 | 1.3 (0.7, 2.3) | 11.2 (-10.2, 43.8) |
|  | 9.7.2.1.2 Corpus uteri, other adenocarcinoma | 13/7 | 1.8 (0.9, 3.0) | 30.4 (-2.6, 80.5) |
|  | 9.7.3 Carcinoma of vulva and vagina | 30/11 | 2.8 (1.9, 3.9) | 53.9 (26.4, 90.0) |
|  | 9.8 Carcinoma of urinary tract | 29/15 | 1.9 (1.3, 2.7) | 25.0 (7.6, 48.1) |
|  | 9.8.1 Carcinoma of kidney | 13/11 | 1.2 (0.6, 2.0) | 5.3 (-9.9, 28.5) |
|  | 9.8.1.1 Kidney, adenocarcinoma | 13/11 | 1.2 (0.6, 2.0) | 5.3 (-9.9, 28.5) |
|  | 9.8.1.1.1 Kidney, renal cell | 12/10 | 1.2 (0.6, 2.1) | 5.2 (-10.7, 29.8) |
|  | 9.8.2 Carcinoma of bladder | 13/4 | 3.6 (1.9, 6.2) | 74.0 (26.1, 146.8) |
|  | 9.8.2.1 Urinary bladder, transitional cell carcinoma | 11/3 | 4.0 (2.0, 7.1) | 82.8 (27.5, 170.0) |
|  | 9.9 Other invasive carcinomas | 12/5 | 2.3 (1.2, 4.0) | 34.6 (5.1, 80.1) |

Abbreviation: NOS=Not otherwise specified, CNS=Central Nervous System.

**Supplementary Table S10B.** Standardised incidence ratios (SIRs) and absolute excess risks (AERs) of second primary carcinomas after each distinct first primary cancer type among six-month adolescent and young adult (AYA, aged 15-39 years) cancer survivors compared to the general population in the Netherlands. Second primary cancer types are grouped according to the AYA-specific classification scheme developed by Barr and colleagues (2020). First and second cancer combinations with less than n=10 observed second cancers were excluded from the analyses. This Table presents outcomes from the sensitivity analysis.

|  | **Second primary cancers** | **9. Carcinomas** | | |
| --- | --- | --- | --- | --- |
|  | **First primary cancers** | **Obs/exp** | **SIR (95%CI)** | **AER per 10,000 person-years (95%CI)** |
| **Males** | **1. Leukaemia’s and related disorders** | **42/14** | **3.0 (2.1, 4.0)** | **15.2 (8.8, 23.2)** |
|  | 1.2 Acute myeloid leukaemia | 13/3 | 4.0 (2.2, 6.9) | 21.5 (8.1, 41.8) |
|  | **2. Lymphomas** | **196/57** | **3.4 (3.0, 4.0)** | **19.8 (16.0, 24.0)** |
|  | 2.1 Non-Hodgkin lymphomas | 76/25 | 3.0 (2.4, 3.8) | 18.6 (12.7, 25.5) |
|  | 2.1.3 Diffuse large B-cell (DLBCL) | 31/11 | 2.9 (2.0, 4.2) | 18.5 (9.5, 30.3) |
|  | 2.1.6 Follicular | 20/5 | 3.8 (2.3, 5.8) | 30.6 (14.4, 53.3) |
|  | 2.1.9 Other non-Hodgkin lymphoma NOS | 10/2 | 4.3 (2.1, 8.0) | 38.0 (12.3, 79.4) |
|  | 2.2 Hodgkin lymphoma | 109/28 | 4.0 (3.2, 4.8) | 20.9 (15.9, 26.7) |
|  | 2.2.2 Hodgkin classic, other | 104/26 | 4.0 (3.3, 4.9) | 21.6 (16.3, 27.6) |
|  | **3. CNS and other intracranial and intraspinal neoplasms** | **17/10** | **1.6 (0.9, 2.6)** | **3.7 (-0.3, 9.4)** |
|  | 3.1 Astroglial and related neoplasms | 15/9 | 1.6 (0.9, 2.6) | 3.6 (-0.6, 9.9) |
|  | **4. Sarcomas** | **44/21** | **2.1 (1.5, 2.8)** | **9.1 (4.3, 15.1)** |
|  | 4.4 Fibromatous neoplasms | 16/8 | 2.0 (1.2, 3.3) | 10.6 (1.7, 23.6) |
|  | 4.4.3 Other fibromatous neoplasms | 13/7 | 2.0 (1.1, 3.4) | 10.2 (0.6, 24.9) |
|  | **5. Blood and lymphatic vessel tumours** | **18/5** | **3.7 (2.2, 5.9)** | **27.7 (12.2, 49.6)** |
|  | 5.2 Malignant blood and lymphatic vessel tumours, all sites | 18/5 | 3.7 (2.2, 5.9) | 27.7 (12.2, 49.6) |
|  | 5.2.1 Kaposi sarcoma | 17/5 | 3.7 (2.1, 5.9) | 28.4 (12.1, 51.8) |
|  | **7. Gonadal and related tumours** | **181/110** | **1.6 (1.4, 1.9)** | **5.2 (3.3, 7.2)** |
|  | 7.1 Testis | 173/108 | 1.6 (1.4, 1.9) | 4.9 (3.0, 6.9) |
|  | 7.1.1 Germ cell and trophoblastic | 171/108 | 1.6 (1.4, 1.8) | 4.7 (2.9, 6.8) |
|  | 7.1.1.1 Seminoma | 92/63 | 1.5 (1.2, 1.8) | 4.8 (1.9, 8.1) |
|  | 7.1.1.2 Embryonal carcinoma | 23/13 | 1.8 (1.1, 2.7) | 5.0 (0.8, 10.7) |
|  | 7.1.1.4 Teratoma | 27/17 | 1.6 (1.1, 2.3) | 4.6 (0.5, 10.0) |
|  | 7.1.1.5 Mixed germ cell | 19/10 | 2.0 (1.2, 3.0) | 4.8 (0.9, 10.2) |
|  | **8. Melanoma, malignant** | **82/66** | **1.2 (1.0, 1.5)** | **2.4 (-0.2, 5.5)** |
|  | 8.1 Superficial spreading/low cumulative sun damage melanoma | 55/39 | 1.4 (1.1, 1.8) | 4.1 (0.7, 8.3) |
|  | 8.3 Other malignant | 24/20 | 1.2 (0.8, 1.8) | 2.0 (-2.8, 8.6) |
|  | **9. Carcinomas** | **352/76** | **4.6 (4.1, 5.1)** | **43.5 (37.9, 49.6)** |
|  | 9.1 Thyroid carcinoma | 16/10 | 1.5 (0.9, 2.5) | 5.7 (-1.2, 15.6) |
|  | 9.2 Other carcinoma of head and neck | 90/13 | 6.7 (5.4, 8.3) | 70.3 (54.1, 89.2) |
|  | 9.2.2 Oral cavity, lip, and pharynx | 59/8 | 7.8 (5.9, 10.0) | 91.0 (66.1, 121.3) |
|  | 9.2.2.1 Oral cavity, lip, and pharynx, squamous | 56/6 | 9.2 (6.9, 11.9) | 112.3 (81.5, 150.0) |
|  | 9.2.4 Other carcinoma of head and neck | 23/3 | 7.5 (4.7, 11.2) | 105.6 (61.0, 166.7) |
|  | 9.3 Carcinoma of gastrointestinal tract | 132/27 | 4.8 (4.0, 5.7) | 45.7 (36.3, 56.4) |
|  | 9.3.4 Carcinoma of colon | 85/14 | 6.0 (4.8, 7.4) | 58.0 (44.0, 74.5) |
|  | 9.3.4.2 Colon excluding appendix | 78/12 | 6.6 (5.2, 8.3) | 77.3 (58.2, 99.9) |
|  | 9.3.4.2.2 Colon excluding appendix, adenocarcinoma | 76/12 | 6.6 (5.2, 8.2) | 77.2 (57.8, 100.1) |
|  | 9.3.5 Carcinoma of rectum | 24/7 | 3.4 (2.2, 5.0) | 29.4 (14.4, 49.8) |
|  | 9.3.5.2 Rectum, adenocarcinoma | 22/6 | 3.4 (2.1, 5.2) | 30.5 (14.4, 52.7) |
|  | 9.4 Carcinoma of lung, bronchus, and trachea | 16/4 | 4.5 (2.6, 7.3) | 37.4 (16.8, 67.4) |
|  | 9.4.2 Non-small cell carcinoma | 13/3 | 3.9 (2.1, 6.7) | 30.7 (11.4, 60.0) |
|  | 9.5 Carcinoma of skin (if collected) | 33/7 | 4.9 (3.4, 6.9) | 49.7 (30.3, 75.0) |
|  | 9.8 Carcinoma of urinary tract | 56/12 | 4.8 (3.6, 6.2) | 53.8 (37.2, 74.1) |
|  | 9.8.1 Carcinoma of kidney | 37/7 | 5.2 (3.6, 7.1) | 56.4 (35.7, 82.9) |
|  | 9.8.1.1 Kidney, adenocarcinoma | 37/7 | 5.2 (3.7, 7.2) | 57.0 (36.1, 83.7) |
|  | 9.8.1.1.1 Kidney, renal cell | 33/7 | 4.9 (3.4, 6.9) | 53.5 (32.6, 80.7) |
|  | 9.8.2 Carcinoma of bladder | 16/4 | 4.1 (2.4, 6.7) | 47.5 (20.7, 86.7) |
|  | 9.8.2.1 Urinary bladder, transitional cell carcinoma | 13/4 | 3.5 (1.9, 6.1) | 40.0 (14.0, 79.5) |
| **Females** | **1. Leukaemia’s and related disorders** | **45/30** | **1.5 (1.1, 2.0)** | **9.5 (1.9, 19.1)** |
|  | 1.2 Acute myeloid leukaemia | 20/11 | 1.8 (1.1, 2.8) | 16.6 (2.0, 37.0) |
|  | 1.2.2 Other acute myeloid leukaemia | 13/8 | 1.5 (0.8, 2.6) | 11.0 (-3.6, 33.2) |
|  | **2. Lymphomas** | **251/96** | **2.6 (2.3, 3.0)** | **29.5 (23.8, 35.8)** |
|  | 2.1 Non-Hodgkin lymphomas | 76/39 | 1.9 (1.5, 2.4) | 21.0 (11.7, 32.0) |
|  | 2.1.3 Diffuse large B-cell (DLBCL) | 26/14 | 1.8 (1.2, 2.7) | 17.9 (4.1, 36.3) |
|  | 2.1.5 Anaplastic T-cell and null-cell excluding NK/T-cell | 14/6 | 2.4 (1.3, 4.1) | 29.5 (6.9, 63.3) |
|  | 2.1.6 Follicular | 19/11 | 1.8 (1.1, 2.8) | 22.3 (1.8, 51.2) |
|  | 2.2 Hodgkin lymphoma | 164/50 | 3.3 (2.8, 3.8) | 34.3 (27.0, 42.5) |
|  | 2.2.2 Hodgkin classic, other | 162/49 | 3.3 (2.8, 3.8) | 34.9 (27.5, 43.2) |
|  | **3. CNS and other intracranial and intraspinal neoplasms** | **29/22** | **1.3 (0.9, 1.9)** | **5.3 (-1.9, 14.8)** |
|  | 3.1 Astroglial and related neoplasms | 26/19 | 1.4 (0.9, 2.0) | 6.0 (-1.8, 16.5) |
|  | 3.1.4 Other astrocytoma/astroglial neoplasms | 16/11 | 1.5 (0.8, 2.4) | 7.3 (-2.6, 21.8) |
|  | 3.1.4.3 Other astrocytoma/astroglial, invasive | 13/10 | 1.3 (0.7, 2.2) | 5.1 (-4.8, 20.1) |
|  | **4. Sarcomas** | **72/50** | **1.4 (1.1, 1.8)** | **8.9 (2.5, 16.6)** |
|  | 4.4 Fibromatous neoplasms | 16/19 | 0.9 (0.5, 1.4) | -3.3 (-11.9, 9.3) |
|  | 4.4.3 Other fibromatous neoplasms | 12/15 | 0.8 (0.4, 1.4) | -5.2 (-14.0, 8.3) |
|  | 4.5 Liposarcoma | 14/6 | 2.4 (1.3, 4.0) | 35.7 (7.9, 77.2) |
|  | **7. Gonadal and related tumours** | **88/74** | **1.2 (1.0, 1.5)** | **4.8 (-1.3, 12.0)** |
|  | 7.2 Ovary | 81/68 | 1.2 (0.9, 1.5) | 5.0 (-1.4, 12.6) |
|  | 7.2.1 Germ cell and trophoblastic | 10/7 | 1.5 (0.7, 2.8) | 8.1 (-4.4, 28.4) |
|  | 7.2.2 Non-germ cell | 71/61 | 1.2 (0.9, 1.5) | 4.4 (-2.8, 12.9) |
|  | 7.2.2.1 Carcinoma | 68/59 | 1.2 (0.9, 1.5) | 4.4 (-2.9, 13.1) |
|  | 7.2.2.1.1 Adenocarcinoma | 68/58 | 1.2 (0.9, 1.5) | 4.9 (-2.5, 13.8) |
|  | 7.2.2.1.1.2 Cystadenocarcinoma | 56/46 | 1.2 (0.9, 1.6) | 6.1 (-2.2, 16.2) |
|  | **8. Melanoma, malignant** | **382/312** | **1.2 (1.1, 1.4)** | **5.4 (2.5, 8.5)** |
|  | 8.1 Superficial spreading/low cumulative sun damage melanoma | 246/200 | 1.2 (1.1, 1.4) | 5.3 (1.9, 9.2) |
|  | 8.2 Nodular melanoma | 35/25 | 1.4 (1.0, 2.0) | 9.5 (-0.2, 22.0) |
|  | 8.3 Other malignant | 101/87 | 1.2 (0.9, 1.4) | 4.2 (-1.4, 10.6) |
|  | **9. Carcinomas** | **2,479/1,006** | **2.5 (2.4, 2.6)** | **41.7 (38.9, 44.5)** |
|  | 9.1 Thyroid carcinoma | 90/74 | 1.2 (1.0, 1.5) | 4.8 (-0.6, 11.2) |
|  | 9.1.3 Papillary | 37/37 | 1.0 (0.7, 1.4) | 0.0 (-6.5, 8.3) |
|  | 9.1.4 Follicular | 17/12 | 1.4 (0.8, 2.2) | 8.7 (-4.8, 28.1) |
|  | 9.1.5 Papillary with follicular variant | 26/20 | 1.3 (0.8, 1.9) | 7.4 (-4.0, 22.8) |
|  | 9.2 Other carcinoma of head and neck | 81/21 | 3.9 (3.1, 4.8) | 70.8 (51.2, 94.0) |
|  | 9.2.2 Oral cavity, lip, and pharynx | 51/9 | 5.4 (4.0, 7.1) | 114.4 (78.6, 158.5) |
|  | 9.2.2.1 Oral cavity, lip, and pharynx, squamous | 47/7 | 7.2 (5.3, 9.6) | 170.8 (118.2, 236.2) |
|  | 9.2.3 Salivary gland | 12/6 | 1.9 (1.0, 3.3) | 19.3 (-0.5, 49.8) |
|  | 9.2.3.2 Salivary gland, other malignant | 10/5 | 2.1 (1.0, 3.9) | 26.8 (0.7, 68.9) |
|  | 9.2.4 Other carcinoma of head and neck | 12/3 | 4.1 (2.1, 7.1) | 89.5 (32.2, 178.1) |
|  | 9.3 Carcinoma of gastrointestinal tract | 131/63 | 2.1 (1.7, 2.5) | 26.9 (18.5, 36.6) |
|  | 9.3.4 Carcinoma of colon | 83/36 | 2.3 (1.8, 2.9) | 31.0 (19.8, 44.2) |
|  | 9.3.4.1 Appendix | 14/13 | 1.1 (0.6, 1.9) | 1.9 (-7.2, 15.5) |
|  | 9.3.4.1.1 NET | 13/11 | 1.1 (0.6, 2.0) | 2.6 (-6.8, 16.9) |
|  | 9.3.4.2 Colon excluding appendix | 69/23 | 2.9 (2.3, 3.7) | 56.0 (37.2, 78.6) |
|  | 9.3.4.2.2 Colon excluding appendix, adenocarcinoma | 67/23 | 2.9 (2.3, 3.7) | 55.7 (36.7, 78.6) |
|  | 9.3.5 Carcinoma of rectum | 23/16 | 1.5 (0.9, 2.2) | 13.2 (-1.8, 33.7) |
|  | 9.3.5.2 Rectum, adenocarcinoma | 18/14 | 1.3 (0.8, 2.1) | 8.9 (-6.4, 30.7) |
|  | 9.4 Carcinoma of lung, bronchus, and trachea | 33/14 | 2.3 (1.6, 3.2) | 35.2 (15.8, 60.3) |
|  | 9.4.2 Non-small cell carcinoma | 33/14 | 2.4 (1.6, 3.3) | 36.9 (17.0, 62.7) |
|  | 9.4.2.1 Non-small cell, adenocarcinoma | 16/3 | 4.8 (2.8, 7.8) | 101.3 (46.5, 181.0) |
|  | 9.4.2.3 Non-small cell, other | 10/2 | 4.9 (2.3, 9.0) | 116.8 (40.3, 240.0) |
|  | 9.5 Carcinoma of skin (if collected) | 41/18 | 2.3 (1.7, 3.1) | 37.5 (18.8, 61.2) |
|  | 9.6 Carcinoma of breast | 1,696/547 | 3.1 (3.0, 3.3) | 61.9 (57.6, 66.3) |
|  | 9.6.1 Breast, infiltrating duct | 1,294/424 | 3.1 (2.9, 3.2) | 59.1 (54.3, 64.0) |
|  | 9.6.10 Breast, other | 51/15 | 3.3 (2.5, 4.3) | 76.1 (48.2, 110.4) |
|  | 9.6.2 Breast, adenocarcinoma | 162/54 | 3.0 (2.6, 3.5) | 64.8 (50.4, 81.0) |
|  | 9.6.3 Breast, lobular | 100/29 | 3.5 (2.8, 4.2) | 79.6 (58.8, 103.8) |
|  | 9.6.5 Breast, medullary | 70/18 | 3.9 (3.1, 5.0) | 93.4 (65.8, 126.4) |
|  | 9.7 Carcinoma of genital sites excluding ovary and testis | 358/249 | 1.4 (1.3, 1.6) | 13.1 (8.7, 17.8) |
|  | 9.7.1 Carcinoma of uterine cervix | 308/225 | 1.4 (1.2, 1.5) | 11.0 (6.5, 15.8) |
|  | 9.7.1.1 Cervix, squamous | 208/159 | 1.3 (1.1, 1.5) | 9.1 (4.0, 14.8) |
|  | 9.7.1.2 Cervix, adenosquamous | 12/7 | 1.6 (0.8, 2.9) | 18.5 (-4.5, 54.1) |
|  | 9.7.1.3 Cervix, adenocarcinoma | 67/40 | 1.7 (1.3, 2.1) | 18.6 (8.0, 31.2) |
|  | 9.7.1.4 Cervix, other | 21/18 | 1.2 (0.7, 1.8) | 5.5 (-9.7, 26.6) |
|  | 9.7.2 Corpus uteri | 17/13 | 1.3 (0.8, 2.1) | 11.3 (-7.7, 38.8) |
|  | 9.7.2.1 Corpus uteri, adenocarcinoma | 14/10 | 1.4 (0.8, 2.4) | 14.7 (-7.7, 48.2) |
|  | 9.7.2.1.2 Corpus uteri, other adenocarcinoma | 14/7 | 1.9 (1.0, 3.2) | 35.9 (1.4, 87.4) |
|  | 9.7.3 Carcinoma of vulva and vagina | 33/11 | 3.0 (2.1, 4.3) | 62.6 (33.5, 100.3) |
|  | 9.8 Carcinoma of urinary tract | 37/15 | 2.5 (1.8, 3.5) | 41.9 (21.3, 68.3) |
|  | 9.8.1 Carcinoma of kidney | 19/10 | 1.8 (1.1, 2.8) | 22.3 (2.6, 50.2) |
|  | 9.8.1.1 Kidney, adenocarcinoma | 19/10 | 1.8 (1.1, 2.8) | 22.3 (2.6, 50.2) |
|  | 9.8.1.1.1 Kidney, renal cell | 18/10 | 1.9 (1.1, 2.9) | 23.7 (2.8, 53.5) |
|  | 9.8.2 Carcinoma of bladder | 14/4 | 4.0 (2.2, 6.6) | 83.9 (33.0, 160.0) |
|  | 9.8.2.1 Urinary bladder, transitional cell carcinoma | 12/3 | 4.5 (2.3, 7.8) | 95.6 (36.1, 187.7) |
|  | 9.9 Other invasive carcinomas | 12/5 | 2.3 (1.2, 4.0) | 34.6 (5.1, 80.1) |

Abbreviation: NOS=Not otherwise specified, CNS=Central Nervous System.

**Supplementary Table S11A.** Standardised incidence ratios (SIRs) and absolute excess risks (AERs) of second primary thyroid carcinomas after each distinct first primary cancer type among six-month adolescent and young adult (AYA, aged 15-39 years) cancer survivors compared to the general population in the Netherlands. Second primary cancer types are grouped according to the AYA-specific classification scheme developed by Barr and colleagues (2020). First and second cancer combinations with less than n=10 observed second cancers were excluded from the analyses. This Table presents outcomes from the main analysis.

|  | **Second primary cancers** | **9.1 Thyroid carcinoma** | | | **9.1.3 Papillary** | | |
| --- | --- | --- | --- | --- | --- | --- | --- |
|  | **First primary cancers** | **Obs/exp** | **SIR (95%CI)** | **AER per 10,000 person-years (95%CI)** | **Obs/exp** | **SIR (95%CI)** | **AER per 10,000 person-years (95%CI)** |
| **Males** | **2. Lymphomas** | **10/2** | **5.6 (2.7, 10.3)** | **1.2 (0.4, 2.4)** | **NA** | **NA** | **NA** |
|  | **7. Gonadal and related tumours** | **11/4** | **2.9 (1.5, 5.2)** | **0.5 (0.1, 1.1)** | **10/2** | **4.1 (2.0, 7.6)** | **0.5 (0.2, 1.1)** |
|  | 7.1 Testis | 10/4 | 2.7 (1.3, 5.0) | 0.5 (0.1, 1.1) | NA | NA | NA |
|  | 7.1.1 Germ cell and trophoblastic | 10/4 | 2.7 (1.3, 5.0) | 0.5 (0.1, 1.1) | NA | NA | NA |
| **Females** | **2. Lymphomas** | **16/4** | **4.1 (2.4, 6.7)** | **2.3 (1.0, 4.2)** | **12/3** | **4.2 (2.2, 7.3)** | **1.7 (0.6, 3.4)** |
|  | 2.2 Hodgkin lymphoma | 12/2 | 5.2 (2.7, 9.1) | 2.9 (1.2, 5.6) | NA | NA | NA |
|  | 2.2.2 Hodgkin classic, other | 12/2 | 5.3 (2.8, 9.3) | 3.0 (1.2, 5.8) | NA | NA | NA |
|  | **8. Melanoma, malignant** | **18/11** | **1.6 (0.9, 2.5)** | **0.5 (-0.1, 1.3)** | **16/8** | **1.9 (1.1, 3.1)** | **0.6 (0.1, 1.3)** |
|  | 8.1 Superficial spreading/low cumulative sun damage melanoma | 12/8 | 1.6 (0.8, 2.8) | 0.5 (-0.1, 1.5) | 10/6 | 1.8 (0.9, 3.3) | 0.5 (-0.1, 1.4) |
|  | **9. Carcinomas** | **37/31** | **1.2 (0.8, 1.6)** | **0.2 (-0.1, 0.5)** | **20/23** | **0.9 (0.5, 1.3)** | **-0.1 (-0.3, 0.2)** |
|  | 9.6 Carcinoma of breast | 32/17 | 1.9 (1.3, 2.7) | 0.8 (0.3, 1.4) | 18/12 | 1.4 (0.9, 2.3) | 0.3 (-0.1, 0.8) |
|  | 9.6.1 Breast, infiltrating duct | 28/13 | 2.1 (1.4, 3.0) | 0.9 (0.3, 1.8) | 15/10 | 1.5 (0.8, 2.5) | 0.3 (-0.1, 1.0) |

Abbreviation: NA=Not Applicable.

**Supplementary Table S11B.** Standardised incidence ratios (SIRs) and absolute excess risks (AERs) of second primary thyroid carcinomas after each distinct first primary cancer type among six-month adolescent and young adult (AYA, aged 15-39 years) cancer survivors compared to the general population in the Netherlands. Second primary cancer types are grouped according to the AYA-specific classification scheme developed by Barr and colleagues (2020). First and second cancer combinations with less than n=10 observed second cancers were excluded from the analyses. This Table presents outcomes from the sensitivity analysis.

|  | **Second primary cancers** | **9.1 Thyroid carcinoma** | | | **9.1.3 Papillary** | | |
| --- | --- | --- | --- | --- | --- | --- | --- |
|  | **First primary cancers** | **Obs/exp** | **SIR (95%CI)** | **AER per 10,000 person-years (95%CI)** | **Obs/exp** | **SIR (95%CI)** | **AER per 10,000 person-years (95%CI)** |
| **Males** | **2. Lymphomas** | **10/2** | **5.6 (2.7, 10.4)** | **1.2 (0.4, 2.4)** | **NA** | **NA** | **NA** |
|  | **7. Gonadal and related tumours** | **11/4** | **3.0 (1.5, 5.4)** | **0.5 (0.1, 1.2)** | **10/2** | **4.2 (2.0, 7.8)** | **0.6 (0.2, 1.2)** |
|  | 7.1 Testis | 10/4 | 2.8 (1.3, 5.1) | 0.5 (0.1, 1.1) | NA | NA | NA |
|  | 7.1.1 Germ cell and trophoblastic | 10/4 | 2.8 (1.3, 5.1) | 0.5 (0.1, 1.1) | NA | NA | NA |
| **Females** | **2. Lymphomas** | **16/4** | **4.1 (2.4, 6.7)** | **2.3 (1.0, 4.2)** | **12/3** | **4.2 (2.2, 7.3)** | **1.7 (0.6, 3.4)** |
|  | 2.2 Hodgkin lymphoma | 12/2 | 5.2 (2.7, 9.1) | 2.9 (1.2, 5.6) | NA | NA | NA |
|  | 2.2.2 Hodgkin classic, other | 12/2 | 5.3 (2.8, 9.3) | 3.0 (1.2, 5.8) | NA | NA | NA |
|  | **8. Melanoma, malignant** | **17/11** | **1.6 (0.9, 2.5)** | **0.5 (-0.1, 1.2)** | **15/8** | **1.8 (1.0, 3.0)** | **0.5 (0.0, 1.3)** |
|  | 8.1 Superficial spreading/low cumulative sun damage melanoma | 12/7 | 1.7 (0.9, 2.9) | 0.6 (-0.1, 1.6) | 10/5 | 1.9 (0.9, 3.4) | 0.5 (-0.1, 1.5) |
|  | **9. Carcinomas** | **34/30** | **1.1 (0.8, 1.6)** | **0.1 (-0.2, 0.5)** | **18/22** | **0.8 (0.5, 1.3)** | **-0.1 (-0.3, 0.2)** |
|  | 9.6 Carcinoma of breast | 29/16 | 1.8 (1.2, 2.6) | 0.7 (0.2, 1.4) | 16/12 | 1.4 (0.8, 2.2) | 0.2 (-0.1, 0.8) |
|  | 9.6.1 Breast, infiltrating duct | 26/13 | 2.0 (1.3, 3.0) | 0.9 (0.3, 1.7) | 14/9 | 1.5 (0.8, 2.5) | 0.3 (-0.1, 1.0) |

Abbreviation: NA=Not Applicable.

**Supplementary Table S12A.** Standardised incidence ratios (SIRs) and absolute excess risks (AERs) of second primary other carcinomas of the head and neck after each distinct first primary cancer type among six-month adolescent and young adult (AYA, aged 15-39 years) cancer survivors compared to the general population in the Netherlands. Second primary cancer types are grouped according to the AYA-specific classification scheme developed by Barr and colleagues (2020). First and second cancer combinations with less than n=10 observed second cancers were excluded from the analyses. This Table presents outcomes from the main analysis.

|  | **Second primary cancers** | **9.2 Other carcinoma of head and neck** | | | **9.2.2 Oral cavity, lip, and pharynx** | | | **9.2.2.1 Oral cavity, lip, and pharynx, squamous** | | | **9.2.4 Other carcinoma of head and neck** | | |
| --- | --- | --- | --- | --- | --- | --- | --- | --- | --- | --- | --- | --- | --- |
|  | **First primary cancers** | **Obs/exp** | **SIR (95%CI)** | **AER per 10,000 person-years (95%CI)** | **Obs/exp** | **SIR (95%CI)** | **AER per 10,000 person-years (95%CI)** | **Obs/exp** | **SIR (95%CI)** | **AER per 10,000 person-years (95%CI)** | **Obs/exp** | **SIR (95%CI)** | **AER per 10,000 person-years (95%CI)** |
| **Males** | **2. Lymphomas** | **20/5** | **4.3 (2.6, 6.6)** | **2.2 (1.1, 3.7)** | **12/3** | **4.0 (2.0, 6.9)** | **1.3 (0.5, 2.6)** | **10/3** | **3.4 (1.6, 6.3)** | **1.0 (0.3, 2.2)** | NA | NA | NA |
|  | 2.1 Non-Hodgkin lymphomas | 10/2 | 4.9 (2.4, 9.1) | 2.9 (1.0, 5.9) | NA | NA | NA | NA | NA | NA | NA | NA | NA |
|  | **9. Carcinomas** | **52/6** | **8.4 (6.3, 11.1)** | **7.1 (5.1, 9.7)** | **47/4** | **11.6 (8.5, 15.4)** | **6.7 (4.8, 9.1)** | **46/4** | **11.7 (8.6, 15.6)** | **6.6 (4.6, 9.0.)** | NA | NA | NA |
|  | 9.2 Other carcinoma of head and neck | 34/1 | 31.1 (21.6, 43.5) | 30.0 (20.5, 42.3) | 32/1 | 44.4 (30.4, 62.7) | 28.5 (19.3, 40.5) | 32/1 | 46.0 (31.5, 64.9) | 28.5 (19.3, 40.5) | NA | NA | NA |
|  | 9.2.2 Oral cavity, lip, and pharynx | 25/1 | 40.9 (26.5, 60.4) | 42.9 (27.4, 63.9) | 23/0 | 56.8 (36.0, 85.3) | 39.8 (24.9, 60.0) | 23/0 | 58.8 (37.3, 88.2) | 39.8 (25.0, 60.0) | NA | NA | NA |
|  | 9.2.2.1 Oral cavity, lip, and pharynx, squamous | 24/0 | 48.3 (30.9, 71.8) | 52.5 (33.3, 78.7) | 22/0 | 66.9 (41.9, 101.2) | 48.4 (30.1, 73.7) | 22/0 | 69.1 (43.3, 104.7) | 48.5 (30.1, 73.8) | NA | NA | NA |
|  | 9.5 Carcinoma of skin (if collected) | 10/1 | 18.6 (8.9, 34.2) | 17.8 (8.0, 33.6) | NA | NA | NA | NA | NA | NA | NA | NA | NA |
| **Females** | **9. Carcinomas** | **62/21** | **3.0 (2.3, 3.8)** | **1.1 (0.7, 1.6)** | **42/14** | **3.0 (2.2, 4.1)** | **0.8 (0.4, 1.2)** | **40/12** | **3.2 (2.3, 4.4)** | **0.8 (0.4, 1.2)** | **12/4** | **3.0 (1.6, 5.2)** | **0.2 (0.1, 0.5)** |
|  | 9.2 Other carcinoma of head and neck | 22/0 | 53.6 (33.6, 81.2) | 25.2 (15.6, 38.3) | 17/0 | 62.4 (36.4, 99.9) | 19.5 (11.2, 31.4) | 17/0 | 70.3 (41.0, 112.6) | 19.5 (11.3, 31.4) | NA | NA | NA |
|  | 9.2.2 Oral cavity, lip, and pharynx | 15/0 | 79.4 (44.5, 131.0) | 40.1 (22.2, 66.4) | 10/0 | 79.4 (38.1, 146.0) | 26.7 (12.6, 49.4) | 10/0 | 89.6 (43.0, 164.8) | 26.8 (12.7, 49.5) | NA | NA | NA |
|  | 9.2.2.1 Oral cavity, lip, and pharynx, squamous | 15/0 | 112.1 (62.7, 184.8) | 61.2 (34.0, 101.3) | 10/0 | 111.1 (53.3, 204.3) | 40.8 (19.4, 75.4) | 10/0 | 125.0 (59.9, 229.8) | 40.9 (19.4, 75.4) | NA | NA | NA |
|  | 9.6 Carcinoma of breast | 14/12 | 1.2 (0.7, 2.0) | 0.1 (-0.2, 0.6) | 10/8 | 1.3 (0.6, 2.3) | 0.1 (-0.2, 0.5) | NA | NA | NA | NA | NA | NA |
|  | 9.6.1 Breast, infiltrating duct | 14/9 | 1.6 (0.9, 2.6) | 0.3 (-0.1, 0.9) | 10/6 | 1.7 (0.8, 3.1) | 0.3 (-0.1, 0.8) | NA | NA | NA | NA | NA | NA |
|  | 9.7 Carcinoma of genital sites excluding ovary and testis | 15/5 | 3.0 (1.7, 5.0) | 1.2 (0.4, 2.4) | 10/3 | 3.0 (1.4, 5.5) | 0.8 (0.2, 1.8) | 10/3 | 3.3 (1.6, 6.1) | 0.8 (0.2, 1.9) | NA | NA | NA |

Abbreviation: NA=Not Applicable.

**Supplementary Table S12B.** Standardised incidence ratios (SIRs) and absolute excess risks (AERs) of second primary other carcinomas of the head and neck after each distinct first primary cancer type among six-month adolescent and young adult (AYA, aged 15-39 years) cancer survivors compared to the general population in the Netherlands. Second primary cancer types are grouped according to the AYA-specific classification scheme developed by Barr and colleagues (2020). First and second cancer combinations with less than n=10 observed second cancers were excluded from the analyses. This Table presents outcomes from the sensitivity analysis.

|  | **Second primary cancers** | **9.2 Other carcinoma of head and neck** | | | **9.2.2 Oral cavity, lip, and pharynx** | | | **9.2.2.1 Oral cavity, lip, and pharynx, squamous** | | | **9.2.4 Other carcinoma of head and neck** | | |
| --- | --- | --- | --- | --- | --- | --- | --- | --- | --- | --- | --- | --- | --- |
|  | **First primary cancers** | **Obs/exp** | **SIR (95%CI)** | **AER per 10,000 person-years (95%CI)** | **Obs/exp** | **SIR (95%CI)** | **AER per 10,000 person-years (95%CI)** | **Obs/exp** | **SIR (95%CI)** | **AER per 10,000 person-years (95%CI)** | **Obs/exp** | **SIR (95%CI)** | **AER per 10,000 person-years (95%CI)** |
| **Males** | **2. Lymphomas** | **20/5** | **4.3 (2.6, 6.7)** | **2.2 (1.1, 3.7)** | **12/3** | **4.0 (2.1, 6.9)** | **1.3 (0.5, 2.6)** | **10/3** | **3.4 (1.6, 6.3)** | **1.0 (0.3, 2.2)** | **NA** | **NA** | **NA** |
|  | 2.1 Non-Hodgkin lymphomas | 10/2 | 5.0 (2.4, 9.1) | 2.9 (1.0, 6.0) | NA | NA | NA | NA | NA | NA | NA | NA | NA |
|  | **9. Carcinomas** | **67/6** | **11.1 (8.6, 14.1)** | **9.6 (7.2, 12.5)** | **57/4** | **14.3 (10.8, 18.5)** | **8.4 (6.2, 11.0)** | **56/4** | **14.6 (11.0, 18.9)** | **8.2 (6.1, 10.9)** | **10/1** | **6.9 (3.3, 12.7)** | **1.3 (0.5, 2.7)** |
|  | 9.2 Other carcinoma of head and neck | 49/1 | 45.6 (33.7, 60.3) | 44.0 (32.3, 58.5) | 42/1 | 59.3 (42.7, 80.2) | 37.9 (27.1, 51.4) | 42/1 | 61.4 (44.2, 83.0) | 37.9 (27.1, 51.5) | NA | NA | NA |
|  | 9.2.2 Oral cavity, lip, and pharynx | 34/1 | 56.3 (39.0, 78.7) | 59.1 (40.6, 83.0) | 33/0 | 82.7 (56.9, 116.1) | 57.7 (39.5, 81.3) | 33/0 | 85.5 (58.9, 120.1) | 57.7 (39.5, 81.3) | NA | NA | NA |
|  | 9.2.2.1 Oral cavity, lip, and pharynx, squamous | 33/0 | 67.5 (46.4, 94.7) | 73.2 (50.0, 103.3) | 32/0 | 98.9 (67.7, 139.7) | 71.3 (48.6, 101.0) | 32/0 | 102.3 (70.0, 144.5) | 71.4 (48.6, 101.0) | NA | NA | NA |
|  | 9.2.4 Other carcinoma of head and neck | 12/0 | 48.5 (25.1, 84.7) | 62.3 (31.6, 109.8) | NA | NA | NA | NA | NA | NA | NA | NA | NA |
|  | 9.5 Carcinoma of skin (if collected) | 10/1 | 18.9 (9.1, 34.7) | 17.9 (8.1, 33.8) | NA | NA | NA | NA | NA | NA | NA | NA | NA |
| **Females** | **9. Carcinomas** | **76/20** | **3.8 (3.0, 4.8)** | **1.6 (1.1, 2.1)** | **52/13** | **3.9 (2.9, 5.1)** | **1.1 (0.7, 1.6)** | **51/12** | **4.3 (3.2, 5.6)** | **1.1 (0.7, 1.6)** | **13/4** | **3.4 (1.8, 5.8)** | **0.3 (0.1, 0.5)** |
|  | 9.2 Other carcinoma of head and neck | 40/0 | 99.4 (71.0, 135.4) | 46.6 (33.1, 63.6) | 30/0 | 112.3 (75.8, 160.4) | 35.0 (23.5, 50.1) | 30/0 | 126.6 (85.4, 180.7) | 35.0 (23.5, 50.1) | NA | NA | NA |
|  | 9.2.2 Oral cavity, lip, and pharynx | 28/0 | 153.4 (102.0, 221.7) | 76.5 (50.6, 110.7) | 23/0 | 189.2 (119.9, 283.9) | 62.9 (39.7, 94.5) | 23/0 | 213.5 (135.3, 320.3) | 62.9 (39.8, 94.6) | NA | NA | NA |
|  | 9.2.2.1 Oral cavity, lip, and pharynx, squamous | 28/0 | 219.6 (145.9, 317.4) | 117.6 (77.9, 170.2) | 23/0 | 268.7 (170.3, 403.1) | 96.7 (61.1, 145.2) | 23/0 | 302.0 (191.4, 453.2) | 96.7 (61.2, 145.3) | NA | NA | NA |
|  | 9.6 Carcinoma of breast | 10/11 | 0.9 (0.4, 1.7) | 0.0 (-0.3, 0.4) | NA | NA | NA | NA | NA | NA | NA | NA | NA |
|  | 9.6.1 Breast, infiltrating duct | 10/8 | 1.2 (0.6, 2.2) | 0.1 (-0.2, 0.7) | NA | NA | NA | NA | NA | NA | NA | NA | NA |
|  | 9.7 Carcinoma of genital sites excluding ovary and testis | 15/5 | 3.0 (1.0.7, 5) | 1.2 (0.4, 2.4) | 10/3 | 3.0 (1.4, 5.5) | 0.8 (0.2, 1.8) | 10/3 | 3.3 (1.6, 6.1) | 0.8 (0.2, 1.9) | NA | NA | NA |
|  | 9.7.1 Carcinoma of uterine cervix | 11/4 | 2.5 (1.2, 4.4) | 0.9 (0.1, 2.0) | NA | NA | NA | NA | NA | NA | NA | NA | NA |

Abbreviation: NA=Not Applicable.

**Supplementary Table S13A.** Standardised incidence ratios (SIRs) and absolute excess risks (AERs) of second primary carcinomas of the gastrointestinal tract after each distinct first primary cancer type among six-month adolescent and young adult (AYA, aged 15-39 years) cancer survivors compared to the general population in the Netherlands. Second primary cancer types are grouped according to the AYA-specific classification scheme developed by Barr and colleagues (2020). First and second cancer combinations with less than n=10 observed second cancers were excluded from the analyses. This Table presents outcomes from the main analysis.

|  | **Second primary cancers** | **9.3 Carcinoma of gastrointestinal tract** | | | **9.3.1 Carcinoma of oesophagus** | | | **9.3.2 Carcinoma of stomach** | | | **9.3.2.3 Stomach, other adenocarcinoma** | | | **9.3.4 Carcinoma of colon** | | | **9.3.4.2 Colon excluding appendix** | | | **9.3.4.2.2 Colon excluding appendix, adenocarcinoma** | | | | **9.3.5 Carcinoma of rectum** | | | **9.3.5.2 Rectum, adenocarcinoma** | | | | **9.3.9 Carcinoma of pancreas** | | | | **9.3.9.1 Pancreas, neuroendocrine** | | | **9.3.9.1.1 NET** | | | | **9.3.9.2 Pancreas, adenocarcinoma** | | |
| --- | --- | --- | --- | --- | --- | --- | --- | --- | --- | --- | --- | --- | --- | --- | --- | --- | --- | --- | --- | --- | --- | --- | --- | --- | --- | --- | --- | --- | --- | --- | --- | --- | --- | --- | --- | --- | --- | --- | --- | --- | --- | --- | --- | --- |
|  | **First primary cancers** | **Obs/exp** | **SIR (95%CI)** | **AER per 10,000 person-years (95%CI)** | **Obs/exp** | **SIR (95%CI)** | **AER per 10,000 person-years (95%CI)** | **Obs/exp** | **SIR (95%CI)** | **AER per 10,000 person-years (95%CI)** | **Obs/exp** | **SIR (95%CI)** | **AER per 10,000 person-years (95%CI)** | **Obs/exp** | **SIR (95%CI)** | **AER per 10,000 person-years (95%CI)** | **Obs/exp** | **SIR (95%CI)** | **AER per 10,000 person-years (95%CI)** | **Obs/exp** | **SIR (95%CI)** | **AER per 10,000 person-years (95%CI)** | **Obs/exp** | | **SIR (95%CI)** | **AER per 10,000 person-years (95%CI)** | **Obs/exp** | **SIR (95%CI)** | **AER per 10,000 person-years (95%CI)** | **Obs/exp** | | **SIR (95%CI)** | **AER per 10,000 person-years (95%CI)** | **Obs/exp** | | **SIR (95%CI)** | **AER per 10,000 person-years (95%CI)** | **Obs/exp** | **SIR (95%CI)** | **AER per 10,000 person-years (95%CI)** | **Obs/exp** | | **SIR (95%CI)** | **AER per 10,000 person-years (95%CI)** |
| **Males** | **1. Leukaemia’s and related disorders** | **11/6** | **2.0  (1.0, 3.6)** | **3.0  (0.0, 7.7)** | **NA** | **NA** | **NA** | **NA** | **NA** | **NA** | **NA** | **NA** | **NA** | **NA** | **NA** | **NA** | **NA** | **NA** | **NA** | **NA** | **NA** | **NA** | **NA** | | **NA** | **NA** | **NA** | **NA** | **NA** | **NA** | | **NA** | **NA** | **NA** | | **NA** | **NA** | **NA** | **NA** | **NA** | **NA** | | **NA** | **NA** |
|  | **2. Lymphomas** | **67/22** | **3.0  (2.3, 3.8)** | **6.4  (4.2, 8.9)** | **NA** | **NA** | **NA** | **16/2** | **8.0  (4.6, 13.0)** | **2.0  (1.0, 3.4)** | **NA** | **NA** | **NA** | **16/8** | **2.0  (1.1, 3.2)** | **1.1  (0.1, 2.5)** | **16/7** | **2.2  (1.2, 3.5)** | **1.2  (0.3, 2.7)** | **15/7** | **2.1  (1.2, 3.4)** | **1.1  (0.2, 2.5)** | **NA** | | **NA** | **NA** | **NA** | **NA** | **NA** | **NA** | | **NA** | **NA** | **NA** | | **NA** | **NA** | **NA** | **NA** | **NA** | **NA** | | **NA** | **NA** |
|  | 2.1 Non-Hodgkin lymphomas | 20/10 | 2.1  (1.3, 3.2) | 3.7  (0.9, 7.7) | **NA** | **NA** | **NA** | **NA** | **NA** | **NA** | **NA** | **NA** | **NA** | **NA** | **NA** | **NA** | **NA** | **NA** | **NA** | **NA** | **NA** | **NA** | **NA** | | **NA** | **NA** | **NA** | **NA** | **NA** | **NA** | | **NA** | **NA** | **NA** | | **NA** | **NA** | **NA** | **NA** | **NA** | **NA** | | **NA** | **NA** |
|  | 2.2 Hodgkin lymphoma | 45/11 | 4.1  (3.0, 5.6) | 8.8  (5.6, 12.7) | **NA** | **NA** | **NA** | 12/1 | 12.1  (6.2, 21.1) | 2.8  (1.3, 5.1) | **NA** | **NA** | **NA** | 11/4 | 2.8  (1.4, 5.0) | 1.8  (0.4, 4.0) | 11/4 | 3.1  (1.5, 5.6) | 1.9  (0.5, 4.1) | 10/3 | 2.9  (1.4, 5.3) | 1.7  (0.3, 3.8) | **NA** | | **NA** | **NA** | **NA** | **NA** | **NA** | **NA** | | **NA** | **NA** | **NA** | | **NA** | **NA** | **NA** | **NA** | **NA** | **NA** | | **NA** | **NA** |
|  | 2.2.2 Hodgkin classic, other | 43/10 | 4.2  (3.1, 5.7) | 9.1  (5.8, 13.2) | **NA** | **NA** | **NA** | 12/1 | 12.9  (6.7, 22.5) | 3.1  (1.5, 5.5) | **NA** | **NA** | **NA** | 10/4 | 2.7  (1.3, 4.9) | 1.7  (0.3, 4.1) | 10/3 | 3.0  (1.4, 5.5) | 1.8  (0.4, 4.2) | **NA** | **NA** | **NA** | **NA** | | **NA** | **NA** | **NA** | **NA** | **NA** | **NA** | | **NA** | **NA** | **NA** | | **NA** | **NA** | **NA** | **NA** | **NA** | **NA** | | **NA** | **NA** |
|  | **4. Sarcomas** | **17/8** | **2.1  (1.2, 3.3)** | **3.5  (0.7, 7.5)** | **NA** | **NA** | **NA** | **NA** | **NA** | **NA** | **NA** | **NA** | **NA** | **NA** | **NA** | **NA** | **NA** | **NA** | **NA** | **NA** | **NA** | **NA** | **NA** | | **NA** | **NA** | **NA** | **NA** | **NA** | **NA** | | **NA** | **NA** | **NA** | | **NA** | **NA** | **NA** | **NA** | **NA** | **NA** | | **NA** | **NA** |
|  | **7. Gonadal and related tumours** | **71/44** | **1.6  (1.3, 2.0)** | **1.9  (0.8, 3.2)** | **NA** | **NA** | **NA** | **11/4** | **2.8  (1.4, 4.9)** | **0.5  (0.1, 1.1)** | **NA** | **NA** | **NA** | **18/16** | **1.1  (0.7, 1.8)** | **0.1  (-0.4, 0.9)** | **17/15** | **1.2  (0.7, 1.9)** | **0.2  (-0.3, 0.9)** | **17/14** | **1.2  (0.7, 1.9)** | **0.2  (-0.3, 0.9)** | **11/11** | | **1.0  (0.5, 1.7)** | **0.0  (-0.4, 0.6)** | **11/11** | **1.0  (0.5, 1.9)** | **0.0  (-0.4, 0.6)** | **12/3** | | **3.5  (1.8, 6.2)** | **0.6  (0.2, 1.2)** | **NA** | | **NA** | **NA** | **NA** | **NA** | **NA** | **12/3** | | **4.4  (2.3, 7.6)** | **0.7  (0.2, 1.3)** |
|  | 7.1 Testis | 68/43 | 1.6  (1.2, 2.0) | 1.8  (0.7, 3.1) | **NA** | **NA** | **NA** | 10/4 | 2.6  (1.2, 4.7) | 0.4  (0.1, 1.1) | **NA** | **NA** | **NA** | 17/16 | 1.1  (0.6, 1.7) | 0.1  (-0.4, 0.8) | 16/14 | 1.1  (0.6, 1.8) | 0.1  (-0.4, 0.9) | 16/14 | 1.1  (0.7, 1.9) | 0.1  (-0.4, 0.9) | 10/11 | | 0.9  (0.4, 1.6) | -0.1  (-0.5, 0.5) | 10/10 | 1.0  (0.5, 1.8) | 0.0  (-0.4, 0.6) | 12/3 | | 3.6  (1.9, 6.3) | 0.6  (0.2, 1.3) | **NA** | | **NA** | **NA** | **NA** | **NA** | **NA** | 12/3 | | 4.5  (2.3, 7.8) | 0.7  (0.3, 1.3) |
|  | 7.1.1 Germ cell and trophoblastic | 66/43 | 1.5  (1.2, 1.9) | 1.7  (0.6, 3.0) | **NA** | **NA** | **NA** | **NA** | **NA** | **NA** | **NA** | **NA** | **NA** | 17/16 | 1.1  (0.6, 1.7) | 0.1  (-0.4, 0.8) | 16/14 | 1.1  (0.6, 1.8) | 0.1  (-0.4, 0.9) | 16/14 | 1.1  (0.7, 1.9) | 0.1  (-0.4, 0.9) | 10/11 | | 0.9  (0.4, 1.6) | -0.1  (-0.5, 0.5) | 10/10 | 1.0  (0.5, 1.8) | 0.0  (-0.4, 0.6) | 12/3 | | 3.6  (1.9, 6.3) | 0.6  (0.2, 1.3) | **NA** | | **NA** | **NA** | **NA** | **NA** | **NA** | 12/3 | | 4.5  (2.3, 7.8) | 0.7  (0.3, 1.3) |
|  | 7.1.1.1 Seminoma | 37/25 | 1.5  (1.0, 2.0) | 1.9 (0.2, 4.1) | **NA** | **NA** | **NA** | **NA** | **NA** | **NA** | **NA** | **NA** | **NA** | **NA** | **NA** | **NA** | **NA** | **NA** | **NA** | **NA** | **NA** | **NA** | **NA** | | **NA** | **NA** | **NA** | **NA** | **NA** | 10/2 | | 5.1  (2.5, 9.4) | 1.3  (0.4, 2.6) | **NA** | | **NA** | **NA** | **NA** | **NA** | **NA** | 10/2 | | 6.2  (3.0, 11.5) | 1.3  (0.5, 2.6) |
|  | 7.1.1.4 Teratoma | 10/7 | 1.5  (0.7, 2.7) | 1.4  (-0.8, 5.1) | **NA** | **NA** | **NA** | **NA** | **NA** | **NA** | **NA** | **NA** | **NA** | **NA** | **NA** | **NA** | **NA** | **NA** | **NA** | **NA** | **NA** | **NA** | **NA** | | **NA** | **NA** | **NA** | **NA** | **NA** | **NA** | | **NA** | **NA** | **NA** | | **NA** | **NA** | **NA** | **NA** | **NA** | **NA** | | **NA** | **NA** |
|  | **8. Melanoma, malignant** | **22/27** | **0.8  (0.5, 1.3)** | **-0.7  (-1.9, 1.0)** | **NA** | **NA** | **NA** | **NA** | **NA** | **NA** | **NA** | **NA** | **NA** | **NA** | **NA** | **NA** | **NA** | **NA** | **NA** | **NA** | **NA** | **NA** | **NA** | | **NA** | **NA** | **NA** | **NA** | **NA** | **NA** | | **NA** | **NA** | **NA** | | **NA** | **NA** | **NA** | **NA** | **NA** | **NA** | | **NA** | **NA** |
|  | 8.1 Superficial spreading/low cumulative sun damage melanoma | 13/16 | 0.8  (0.4, 1.4) | -0.7  (-2.2, 1.6) | **NA** | **NA** | **NA** | **NA** | **NA** | **NA** | **NA** | **NA** | **NA** | **NA** | **NA** | **NA** | **NA** | **NA** | **NA** | **NA** | **NA** | **NA** | **NA** | | **NA** | **NA** | **NA** | **NA** | **NA** | **NA** | | **NA** | **NA** | **NA** | | **NA** | **NA** | **NA** | **NA** | **NA** | **NA** | | **NA** | **NA** |
|  | **9. Carcinomas** | **102/30** | **3.4  (2.8, 4.2)** | **11.3  (8.3, 14.7)** | **12/3** | **3.5  (1.8, 6.1)** | **1.3  (0.4, 2.7)** | **NA** | **NA** | **NA** | **NA** | **NA** | **NA** | **23/11** | **2.1  (1.3, 3.2)** | **1.9  (0.6, 3.7)** | **22/10** | **2.2  (1.4, 3.3)** | **1.9  (0.6, 3.6)** | **22/10** | **2.2  (1.4, 3.4)** | **1.9  (0.6, 3.7)** | **24/8** | | **3.1  (2.0, 4.7)** | **2.5  (1.2, 4.4)** | **23/7** | **3.2  (2.0, 4.8)** | **2.5  (1.1, 4.3)** | **13/2** | | **5.5  (2.9, 9.5)** | **1.7  (0.7, 3.1)** | **NA** | | **NA** | **NA** | **NA** | **NA** | **NA** | **NA** | | **NA** | **NA** |
|  | 9.2 Other carcinoma of head and neck | 12/5 | 2.3  (1.2, 4.0) | 6.2  (0.9, 14.4) | **NA** | **NA** | **NA** | **NA** | **NA** | **NA** | **NA** | **NA** | **NA** | **NA** | **NA** | **NA** | **NA** | **NA** | **NA** | **NA** | **NA** | **NA** | **NA** | | **NA** | **NA** | **NA** | **NA** | **NA** | **NA** | | **NA** | **NA** | **NA** | | **NA** | **NA** | **NA** | **NA** | **NA** | **NA** | | **NA** | **NA** |
|  | 9.3 Carcinoma of gastrointestinal tract | 57/11 | 5.3  (4.0, 6.9) | 20.0  (14.0, 27.3) | **NA** | **NA** | **NA** | **NA** | **NA** | **NA** | **NA** | **NA** | **NA** | 14/4 | 3.6  (2.0, 6.0) | 4.4  (1.6, 8.5) | 13/4 | 3.6  (1.9, 6.1) | 4.0  (1.4, 8.0) | 13/4 | 3.7  (1.9, 6.3) | 4.1  (1.5, 8.1) | 17/3 | | 6.2  (3.6, 9.9) | 6.2  (3.1, 10.6) | 16/3 | 6.2  (3.5, 10.0) | 5.8  (2.8, 10.1) | **NA** | | **NA** | **NA** | **NA** | | **NA** | **NA** | **NA** | **NA** | **NA** | **NA** | | **NA** | **NA** |
|  | 9.3.4 Carcinoma of colon | 34/6 | 6.0  (4.2, 8.4) | 22.8  (14.4, 33.6) | **NA** | **NA** | **NA** | **NA** | **NA** | **NA** | **NA** | **NA** | **NA** | **NA** | **NA** | **NA** | **NA** | **NA** | **NA** | **NA** | **NA** | **NA** | 15/1 | | 10.3  (5.8, 17.0) | 10.9  (5.6, 18.7) | 14/1 | 10.2  (5.6, 17.1) | 10.1  (5.0, 17.7) | **NA** | | **NA** | **NA** | **NA** | | **NA** | **NA** | **NA** | **NA** | **NA** | **NA** | | **NA** | **NA** |
|  | 9.3.4.2 Colon excluding appendix | 33/5 | 7.1  (4.9, 9.9) | 32.1  (20.5, 47.2) | **NA** | **NA** | **NA** | **NA** | **NA** | **NA** | **NA** | **NA** | **NA** | **NA** | **NA** | **NA** | **NA** | **NA** | **NA** | **NA** | **NA** | **NA** | 15/1 | | 12.4  (7.0, 20.5) | 15.6  (8.2, 26.7) | 14/1 | 12.3  (6.7, 20.6) | 14.6  (7.4, 25.3) | **NA** | | **NA** | **NA** | **NA** | | **NA** | **NA** | **NA** | **NA** | **NA** | **NA** | | **NA** | **NA** |
|  | 9.3.4.2.2 Colon excluding appendix, adenocarcinoma | 32/5 | 7.0  (4.8, 9.8) | 31.9  (20.1, 47.2) | **NA** | **NA** | **NA** | **NA** | **NA** | **NA** | **NA** | **NA** | **NA** | **NA** | **NA** | **NA** | **NA** | **NA** | **NA** | **NA** | **NA** | **NA** | 15/1 | | 12.7  (7.1, 20.9) | 16.1  (8.4, 27.4) | 14/1 | 12.5  (6.8, 20.9) | 15.0  (7.6, 26.0) | **NA** | | **NA** | **NA** | **NA** | | **NA** | **NA** | **NA** | **NA** | **NA** | **NA** | | **NA** | **NA** |
|  | 9.3.5 Carcinoma of rectum | 13/3 | 4.8  (2.5, 8.2) | 17.9  (7.3, 33.9) | **NA** | **NA** | **NA** | **NA** | **NA** | **NA** | **NA** | **NA** | **NA** | 10/1 | 10.0  (4.8, 18.4) | 15.7  (6.6, 30.3) | 10/1 | 10.7  (5.2, 19.8) | 15.8  (6.7, 30.4) | 10/1 | 11.0  (5.3, 20.2) | 15.8  (6.8, 30.4) | **NA** | | **NA** | **NA** | **NA** | **NA** | **NA** | **NA** | | **NA** | **NA** | **NA** | | **NA** | **NA** | **NA** | **NA** | **NA** | **NA** | | **NA** | **NA** |
|  | 9.3.5.2 Rectum, adenocarcinoma | 12/2 | 4.9  (2.5, 8.5) | 18.7  (7.3, 36.3) | **NA** | **NA** | **NA** | **NA** | **NA** | **NA** | **NA** | **NA** | **NA** | 10/1 | 11.0  (5.3, 20.2) | 17.8  (7.6, 34.3) | 10/1 | 11.8  (5.7, 21.7) | 18.0  (7.7, 34.4) | 10/1 | 12.1  (5.8, 22.2) | 18.0  (7.8, 34.5) | **NA** | | **NA** | **NA** | **NA** | **NA** | **NA** | **NA** | | **NA** | **NA** | **NA** | | **NA** | **NA** | **NA** | **NA** | **NA** | **NA** | | **NA** | **NA** |
|  | 9.8 Carcinoma of urinary tract | 14/5 | 3.0  (1.7, 5.1) | 10.9  (3.5, 21.9) | **NA** | **NA** | **NA** | **NA** | **NA** | **NA** | **NA** | **NA** | **NA** | **NA** | **NA** | **NA** | **NA** | **NA** | **NA** | **NA** | **NA** | **NA** | **NA** | | **NA** | **NA** | **NA** | **NA** | **NA** | **NA** | | **NA** | **NA** | **NA** | | **NA** | **NA** | **NA** | **NA** | **NA** | **NA** | | **NA** | **NA** |
|  | 9.8.1 Carcinoma of kidney | 10/3 | 3.4  (1.6, 6.3) | 12.5  (3.3, 27.4) | **NA** | **NA** | **NA** | **NA** | **NA** | **NA** | **NA** | **NA** | **NA** | **NA** | **NA** | **NA** | **NA** | **NA** | **NA** | **NA** | **NA** | **NA** | **NA** | | **NA** | **NA** | **NA** | **NA** | **NA** | **NA** | | **NA** | **NA** | **NA** | | **NA** | **NA** | **NA** | **NA** | **NA** | **NA** | | **NA** | **NA** |
|  | 9.8.1.1 Kidney, adenocarcinoma | 10/3 | 3.5  (1.7, 6.4) | 12.7  (3.4, 27.6) | **NA** | **NA** | **NA** | **NA** | **NA** | **NA** | **NA** | **NA** | **NA** | **NA** | **NA** | **NA** | **NA** | **NA** | **NA** | **NA** | **NA** | **NA** | **NA** | | **NA** | **NA** | **NA** | **NA** | **NA** | **NA** | | **NA** | **NA** | **NA** | | **NA** | **NA** | **NA** | **NA** | **NA** | **NA** | | **NA** | **NA** |
| **Females** | **2. Lymphomas** | **27/12** | **2.3  (1.5, 3.4)** | **2.9  (1.2, 5.3)** | **NA** | **NA** | **NA** | **NA** | **NA** | **NA** | **NA** | **NA** | **NA** | **NA** | **NA** | **NA** | **NA** | **NA** | **NA** | **NA** | **NA** | **NA** | **NA** | | **NA** | **NA** | **NA** | **NA** | **NA** | **NA** | | **NA** | **NA** | **NA** | | **NA** | **NA** | **NA** | **NA** | **NA** | **NA** | | **NA** | **NA** |
|  | 2.2 Hodgkin lymphoma | 16/6 | 2.8  (1.6, 4.5) | 3.1  (1.0, 6.1) | **NA** | **NA** | **NA** | **NA** | **NA** | **NA** | **NA** | **NA** | **NA** | **NA** | **NA** | **NA** | **NA** | **NA** | **NA** | **NA** | **NA** | **NA** | **NA** | | **NA** | **NA** | **NA** | **NA** | **NA** | **NA** | | **NA** | **NA** | **NA** | | **NA** | **NA** | **NA** | **NA** | **NA** | **NA** | | **NA** | **NA** |
|  | 2.2.2 Hodgkin classic, other | 16/6 | 2.8  (1.6, 4.6) | 3.2  (1.1, 6.3) | **NA** | **NA** | **NA** | **NA** | **NA** | **NA** | **NA** | **NA** | **NA** | **NA** | **NA** | **NA** | **NA** | **NA** | **NA** | **NA** | **NA** | **NA** | **NA** | | **NA** | **NA** | **NA** | **NA** | **NA** | **NA** | | **NA** | **NA** | **NA** | | **NA** | **NA** | **NA** | **NA** | **NA** | **NA** | | **NA** | **NA** |
|  | **4. Sarcomas** | **12/6** | **1.9  (1.0, 3.4)** | **2.4  (0.0, 6.1)** | **NA** | **NA** | **NA** | **NA** | **NA** | **NA** | **NA** | **NA** | **NA** | **NA** | **NA** | **NA** | **NA** | **NA** | **NA** | **NA** | **NA** | **NA** | **NA** | | **NA** | **NA** | **NA** | **NA** | **NA** | **NA** | | **NA** | **NA** | **NA** | | **NA** | **NA** | **NA** | **NA** | **NA** | **NA** | | **NA** | **NA** |
|  | **7. Gonadal and related tumours** | **12/10** | **1.2  (0.6, 2.1)** | **0.8  (-1.2, 3.9)** | **NA** | **NA** | **NA** | **NA** | **NA** | **NA** | **NA** | **NA** | **NA** | **NA** | **NA** | **NA** | **NA** | **NA** | **NA** | **NA** | **NA** | **NA** | **NA** | | **NA** | **NA** | **NA** | **NA** | **NA** | **NA** | | **NA** | **NA** | **NA** | | **NA** | **NA** | **NA** | **NA** | **NA** | **NA** | | **NA** | **NA** |
|  | 7.2 Ovary | 12/9 | 1.3  (0.7, 2.3) | 1.1  (-1.1, 4.6) | **NA** | **NA** | **NA** | **NA** | **NA** | **NA** | **NA** | **NA** | **NA** | **NA** | **NA** | **NA** | **NA** | **NA** | **NA** | **NA** | **NA** | **NA** | **NA** | | **NA** | **NA** | **NA** | **NA** | **NA** | **NA** | | **NA** | **NA** | **NA** | | **NA** | **NA** | **NA** | **NA** | **NA** | **NA** | | **NA** | **NA** |
|  | 7.2.2 Non-germ cell | 12/8 | 1.5  (0.8, 2.5) | 1.7  (-0.9, 5.8) | **NA** | **NA** | **NA** | **NA** | **NA** | **NA** | **NA** | **NA** | **NA** | **NA** | **NA** | **NA** | **NA** | **NA** | **NA** | **NA** | **NA** | **NA** | **NA** | | **NA** | **NA** | **NA** | **NA** | **NA** | **NA** | | **NA** | **NA** | **NA** | | **NA** | **NA** | **NA** | **NA** | **NA** | **NA** | | **NA** | **NA** |
|  | 7.2.2.1 Carcinoma | 12/8 | 1.5  (0.8, 2.7) | 1.9  (-0.8, 6.2) | **NA** | **NA** | **NA** | **NA** | **NA** | **NA** | **NA** | **NA** | **NA** | **NA** | **NA** | **NA** | **NA** | **NA** | **NA** | **NA** | **NA** | **NA** | **NA** | | **NA** | **NA** | **NA** | **NA** | **NA** | **NA** | | **NA** | **NA** | **NA** | | **NA** | **NA** | **NA** | **NA** | **NA** | **NA** | | **NA** | **NA** |
|  | 7.2.2.1.1 Adenocarcinoma | 12/8 | 1.5  (0.8, 2.7) | 2.0  (-0.8, 6.4) | **NA** | **NA** | **NA** | **NA** | **NA** | **NA** | **NA** | **NA** | **NA** | **NA** | **NA** | **NA** | **NA** | **NA** | **NA** | **NA** | **NA** | **NA** | **NA** | | **NA** | **NA** | **NA** | **NA** | **NA** | **NA** | | **NA** | **NA** | **NA** | | **NA** | **NA** | **NA** | **NA** | **NA** | **NA** | | **NA** | **NA** |
|  | 7.2.2.1.1.2 Cystadenocarcinoma | 10/6 | 1.6  (0.8, 3.0) | 2.3 (-0.8, 7.4) | **NA** | **NA** | **NA** | **NA** | **NA** | **NA** | **NA** | **NA** | **NA** | **NA** | **NA** | **NA** | **NA** | **NA** | **NA** | **NA** | **NA** | **NA** | **NA** | | **NA** | **NA** | **NA** | **NA** | **NA** | **NA** | | **NA** | **NA** | **NA** | | **NA** | **NA** | **NA** | **NA** | **NA** | **NA** | | **NA** | **NA** |
|  | **8. Melanoma, malignant** | **39/40** | **1.0  (0.7, 1.3)** | **-0.1  (-0.9, 1.0)** | **NA** | **NA** | **NA** | **NA** | **NA** | **NA** | **NA** | **NA** | **NA** | **16/18** | **0.9  (0.5, 1.4)** | **-0.2  (-0.7, 0.6)** | **14/16** | **0.9  (0.5, 1.4)** | **-0.2  (-0.6, 0.5)** | **14/16** | **0.9  (0.5, 1.5)** | **-0.2  (-0.6, 0.6)** | **NA** | | **NA** | **NA** | **NA** | **NA** | **NA** | **NA** | | **NA** | **NA** | **NA** | | **NA** | **NA** | **NA** | **NA** | **NA** | **NA** | | **NA** | **NA** |
|  | 8.1 Superficial spreading/low cumulative sun damage melanoma | 24/25 | 1.0  (0.6, 1.4) | -0.1  (-1.1, 1.2) | **NA** | **NA** | **NA** | **NA** | **NA** | **NA** | **NA** | **NA** | **NA** | 10/12 | 0.9  (0.4, 1.6) | -0.2  (-0.8, 0.8) | 10/10 | 1.0  (0.5, 1.8) | 0.0  (-0.6, 0.9) | 10/10 | 1.0  (0.5, 1.8) | 0.0  (-0.6, 0.9) | **NA** | | **NA** | **NA** | **NA** | **NA** | **NA** | **NA** | | **NA** | **NA** | **NA** | | **NA** | **NA** | **NA** | **NA** | **NA** | **NA** | | **NA** | **NA** |
|  | **9. Carcinomas** | **228/138** | **1.7  (1.4, 1.9)** | **2.5  (1.7, 3.4)** | **22/5** | **4.3  (2.7, 6.5)** | **0.5  (0.2, 0.8)** | **19/9** | **2.0  (1.2, 3.2)** | **0.3  (0.1, 0.6)** | **15/7** | **2.2  (1.2, 3.7)** | **0.2  (0.0, 0.5)** | **78/63** | **1.2  (1.0, 1.5)** | **0.4  (0.0, 0.9)** | **75/57** | **1.3  (1.0, 1.6)** | **0.5  (0.1, 1.0)** | **74/56** | **1.3  (1.0, 1.7)** | **0.5  (0.1, 1.0)** | **53/32** | | **1.7  (1.3, 2.2)** | **0.6  (0.2, 1.0)** | **52/30** | **1.7  (1.3, 2.2)** | **0.6  (0.2, 1.0)** | **31/14** | | **2.3  (1.6, 3.2)** | **0.5  (0.2, 0.8)** | **11/2** | | **4.5  (2.3, 8.1)** | **0.2  (0.1, 0.5)** | **11/2** | **5.9  (2.9, 10.5)** | **0.3  (0.1, 0.5)** | **19/11** | | **1.8  (1.1, 2.7)** | **0.2  (0.0, 0.5)** |
|  | 9.1 Thyroid carcinoma | 16/9 | 1.7  (1.0, 2.8) | 2.1  (0.0, 5.2) | **NA** | **NA** | **NA** | **NA** | **NA** | **NA** | **NA** | **NA** | **NA** | **NA** | **NA** | **NA** | **NA** | **NA** | **NA** | **NA** | **NA** | **NA** | **NA** | | **NA** | **NA** | **NA** | **NA** | **NA** | **NA** | | **NA** | **NA** | **NA** | | **NA** | **NA** | **NA** | **NA** | **NA** | **NA** | | **NA** | **NA** |
|  | 9.3 Carcinoma of gastrointestinal tract | 35/8 | 4.3  (3.0, 6.0) | 10.6  (6.4, 15.9) | **NA** | **NA** | **NA** | **NA** | **NA** | **NA** | **NA** | **NA** | **NA** | **NA** | **NA** | **NA** | **NA** | **NA** | **NA** | **NA** | **NA** | **NA** | 13/2 | | 7.0  (3.7, 12.0) | 4.4  (2.0, 8.0) | 12/2 | 6.8  (3.5, 11.8) | 4.0  (1.7, 7.5) | **NA** | | **NA** | **NA** | **NA** | | **NA** | **NA** | **NA** | **NA** | **NA** | **NA** | | **NA** | **NA** |
|  | 9.3.4 Carcinoma of colon | 22/5 | 4.7  (3.0, 7.2) | 11.4  (6.0, 18.8) | **NA** | **NA** | **NA** | **NA** | **NA** | **NA** | **NA** | **NA** | **NA** | **NA** | **NA** | **NA** | **NA** | **NA** | **NA** | **NA** | **NA** | **NA** | 12/1 | | 11.3  (5.8, 19.8) | 7.2  (3.4, 13.0) | 11/1 | 10.8  (5.4, 19.4) | 6.5  (2.9, 12.2) | **NA** | | **NA** | **NA** | **NA** | | **NA** | **NA** | **NA** | **NA** | **NA** | **NA** | | **NA** | **NA** |
|  | 9.3.4.2 Colon excluding appendix | 20/3 | 6.4  (3.9, 9.9) | 20.5  (11.1, 33.7) | **NA** | **NA** | **NA** | **NA** | **NA** | **NA** | **NA** | **NA** | **NA** | **NA** | **NA** | **NA** | **NA** | **NA** | **NA** | **NA** | **NA** | **NA** | 11/1 | | 15.4  (7.7, 27.5) | 12.5  (5.8, 23.0) | 10/1 | 14.6  (7.0, 26.8) | 11.3  (5.0, 21.5) | **NA** | | **NA** | **NA** | **NA** | | **NA** | **NA** | **NA** | **NA** | **NA** | **NA** | | **NA** | **NA** |
|  | 9.3.4.2.2 Colon excluding appendix, adenocarcinoma | 19/3 | 6.2  (3.7, 9.7) | 19.9  (10.5, 33.2) | **NA** | **NA** | **NA** | **NA** | **NA** | **NA** | **NA** | **NA** | **NA** | **NA** | **NA** | **NA** | **NA** | **NA** | **NA** | **NA** | **NA** | **NA** | 11/1 | | 15.6  (7.8, 27.9) | 12.9  (6.0, 23.7) | 10/1 | 14.8  (7.1, 27.2) | 11.7  (5.1, 22.1) | **NA** | | **NA** | **NA** | **NA** | | **NA** | **NA** | **NA** | **NA** | **NA** | **NA** | | **NA** | **NA** |
|  | 9.6 Carcinoma of breast | 105/77 | 1.4  (1.1, 1.6) | 1.4  (0.4, 2.6) | 14/3 | 4.8  (2.7, 8.1) | 0.6  (0.2, 1.1) | 11/5 | 2.1  (1.1, 3.8) | 0.3  (0.0, 0.7) | **NA** | **NA** | **NA** | 40/35 | 1.1  (0.8, 1.5) | 0.2  (-0.3, 1.0) | 39/32 | 1.2  (0.9, 1.7) | 0.4  (-0.2, 1.1) | 38/31 | 1.2  (0.9, 1.7) | 0.3  (-0.2, 1.1) | 22/18 | | 1.2  (0.8, 1.9) | 0.2  (-0.2, 0.8) | 22/17 | 1.3  (0.8, 1.9) | 0.3  (-0.2, 0.8) | 14/8 | | 1.8  (1.0, 3.1) | 0.3  (0.0, 0.8) | **NA** | | **NA** | **NA** | **NA** | **NA** | **NA** | 11/6 | | 1.8  (0.9, 3.2) | 0.3  (0.0, 0.7) |
|  | 9.6.1 Breast, infiltrating duct | 78/59 | 1.3  (1.0, 1.7) | 1.2  (0.2, 2.5) | 11/2 | 5.0  (2.5, 9,0) | 0.6  (0.2, 1.1) | **NA** | **NA** | **NA** | **NA** | **NA** | **NA** | 32/27 | 1.2  (0.8, 1.7) | 0.3  (-0.3, 1.2) | 32/24 | 1.3  (0.9, 1.9) | 0.5  (-0.2, 1.3) | 31/24 | 1.3  (0.9, 1.8) | 0.5  (-0.2, 1.3) | 15/14 | | 1.1  (0.6, 1.8) | 0.1  (-0.3, 0.7) | 15/13 | 1.2  (0.6, 1.9) | 0.1  (-0.3, 0.8) | 10/6 | | 1.7  (0.8, 3.2) | 0.3  (-0.1, 0.8) | **NA** | | **NA** | **NA** | **NA** | **NA** | **NA** | **NA** | | **NA** | **NA** |
|  | 9.6.2 Breast, adenocarcinoma | 15/8 | 1.9  (1.1, 3.1) | 4.0  (0.3, 9.6) | **NA** | **NA** | **NA** | **NA** | **NA** | **NA** | **NA** | **NA** | **NA** | **NA** | **NA** | **NA** | **NA** | **NA** | **NA** | **NA** | **NA** | **NA** | **NA** | | **NA** | **NA** | **NA** | **NA** | **NA** | **NA** | | **NA** | **NA** | **NA** | | **NA** | **NA** | **NA** | **NA** | **NA** | **NA** | | **NA** | **NA** |
|  | 9.7 Carcinoma of genital sites excluding ovary and testis | 50/34 | 1.5  (1.1, 2.0) | 2.0  (0.4, 3.9) | **NA** | **NA** | **NA** | **NA** | **NA** | **NA** | **NA** | **NA** | **NA** | 17/15 | 1.1  (0.6, 1.8) | 0.2  (-0.7, 1.4) | 15/14 | 1.1  (0.6, 1.8) | 0.1  (-0.7, 1.3) | 15/14 | 1.1  (0.6, 1.8) | 0.2  (-0.6, 1.3) | 14/8 | | 1.8  (1.0, 3.0) | 0.8  (0.0, 1.9) | 14/7 | 1.9  (1.0, 3.2) | 0.8  (0.0, 1.9) | 10/3 | | 3.0  (1.5, 5.6) | 0.8  (0.2, 1.8) | **NA** | | **NA** | **NA** | **NA** | **NA** | **NA** | **NA** | | **NA** | **NA** |
|  | 9.7.1 Carcinoma of uterine cervix | 42/30 | 1.4  (1.0, 1.9) | 1.6  (0.0, 3.5) | **NA** | **NA** | **NA** | **NA** | **NA** | **NA** | **NA** | **NA** | **NA** | 13/14 | 0.9  (0.5, 1.6) | -0.1  (-0.9, 1.1) | 12/13 | 1.0  (0.5, 1.7) | -0.1  (-0.8, 1.1) | 12/12 | 1.0  (0.5, 1.7) | 0.0  (-0.8, 1.1) | 12/7 | | 1.7  (0.9, 3.0) | 0.7  (-0.1, 1.9) | 12/7 | 1.8  (0.9, 3.1) | 0.7  (-0.1, 1.9) | **NA** | | **NA** | **NA** | **NA** | | **NA** | **NA** | **NA** | **NA** | **NA** | **NA** | | **NA** | **NA** |
|  | 9.7.1.1 Cervix, squamous | 29/21 | 1.4  (0.9, 2.0) | 1.4  (-0.4, 3.8) | **NA** | **NA** | **NA** | **NA** | **NA** | **NA** | **NA** | **NA** | **NA** | 10/10 | 1.0  (0.5, 1.9) | 0.0  (-0.9, 1.6) | **NA** | **NA** | **NA** | **NA** | **NA** | **NA** | **NA** | | **NA** | **NA** | **NA** | **NA** | **NA** | **NA** | | **NA** | **NA** | **NA** | | **NA** | **NA** | **NA** | **NA** | **NA** | **NA** | | **NA** | **NA** |
|  | 9.7.1.3 Cervix, adenocarcinoma | 11/5 | 2.1  (1.0, 3.7) | 4.0  (0.2, 10.1) | **NA** | **NA** | **NA** | **NA** | **NA** | **NA** | **NA** | **NA** | **NA** | **NA** | **NA** | **NA** | **NA** | **NA** | **NA** | **NA** | **NA** | **NA** | **NA** | | **NA** | **NA** | **NA** | **NA** | **NA** | **NA** | | **NA** | **NA** | **NA** | | **NA** | **NA** | **NA** | **NA** | **NA** | **NA** | | **NA** | **NA** |

Abbreviation: NET=Neuroendocrine tumour, NA=Not Applicable.

**Supplementary Table S13B.** Standardised incidence ratios (SIRs) and absolute excess risks (AERs) of second primary carcinomas of the gastrointestinal tract after each distinct first primary cancer type among six-month adolescent and young adult (AYA, aged 15-39 years) cancer survivors compared to the general population in the Netherlands. Second primary cancer types are grouped according to the AYA-specific classification scheme developed by Barr and colleagues (2020). First and second cancer combinations with less than n=10 observed second cancers were excluded from the analyses. This Table presents outcomes from the sensitivity analysis.

|  | **Second primary cancers** | **9.3 Carcinoma of gastrointestinal tract** | | | **9.3.1 Carcinoma of oesophagus** | | | **9.3.2 Carcinoma of stomach** | | | **9.3.2.3 Stomach, other adenocarcinoma** | | | **9.3.4 Carcinoma of colon** | | | **9.3.4.2 Colon excluding appendix** | | | **9.3.4.2.2 Colon excluding appendix, adenocarcinoma** | | | **9.3.5 Carcinoma of rectum** | | | **9.3.5.2 Rectum, adenocarcinoma** | | | **9.3.9 Carcinoma of pancreas** | | | **9.3.9.1 Pancreas, neuroendocrine** | | | **9.3.9.1.1 NET** | | | **9.3.9.2 Pancreas, adenocarcinoma** | | |
| --- | --- | --- | --- | --- | --- | --- | --- | --- | --- | --- | --- | --- | --- | --- | --- | --- | --- | --- | --- | --- | --- | --- | --- | --- | --- | --- | --- | --- | --- | --- | --- | --- | --- | --- | --- | --- | --- | --- | --- | --- |
|  | **First primary cancers** | **Obs/exp** | **SIR (95%CI)** | **AER per 10,000 person-years (95%CI)** | **Obs/exp** | **SIR (95%CI)** | **AER per 10,000 person-years (95%CI)** | **Obs/exp** | **SIR (95%CI)** | **AER per 10,000 person-years (95%CI)** | **Obs/exp** | **SIR (95%CI)** | **AER per 10,000 person-years (95%CI)** | **Obs/exp** | **SIR (95%CI)** | **AER per 10,000 person-years (95%CI)** | **Obs/exp** | **SIR (95%CI)** | **AER per 10,000 person-years (95%CI)** | **Obs/exp** | **SIR (95%CI)** | **AER per 10,000 person-years (95%CI)** | **Obs/exp** | **SIR (95%CI)** | **AER per 10,000 person-years (95%CI)** | **Obs/exp** | **SIR (95%CI)** | **AER per 10,000 person-years (95%CI)** | **Obs/exp** | **SIR (95%CI)** | **AER per 10,000 person-years (95%CI)** | **Obs/exp** | **SIR (95%CI)** | **AER per 10,000 person-years (95%CI)** | **Obs/exp** | **SIR (95%CI)** | **AER per 10,000 person-years (95%CI)** | **Obs/exp** | **SIR (95%CI)** | **AER per 10,000 person-years (95%CI)** |
| **Males** | **1. Leukaemia’s and related disorders** | **11/6** | **2.0  (1.0, 3.6)** | **3.0  (0.0, 7.7)** | **NA** | **NA** | **NA** | **NA** | **NA** | **NA** | **NA** | **NA** | **NA** | **NA** | **NA** | **NA** | **NA** | **NA** | **NA** | **NA** | **NA** | **NA** | **NA** | **NA** | **NA** | **NA** | **NA** | **NA** | **NA** | **NA** | **NA** | **NA** | **NA** | **NA** | **NA** | **NA** | **NA** | **NA** | **NA** | **NA** |
|  | **2. Lymphomas** | **67/22** | **3.0  (2.3, 3.8)** | **6.4  (4.2, 9.0)** | **NA** | **NA** | **NA** | **16/2** | **8.0  (4.6, 13.0)** | **2.0  (1.0, 3.4)** | **NA** | **NA** | **NA** | **16/8** | **2.0  (1.1, 3.2)** | **1.1  (0.2, 2.6)** | **16/7** | **2.2  (1.2, 3.5)** | **1.2  (0.3, 2.7)** | **15/7** | **2.1  (1.2, 3.5)** | **1.1  (0.2, 2.5)** | **NA** | **NA** | **NA** | **NA** | **NA** | **NA** | **NA** | **NA** | **NA** | **NA** | **NA** | **NA** | **NA** | **NA** | **NA** | **NA** | **NA** | **NA** |
|  | 2.1 Non-Hodgkin lymphomas | 20/10 | 2.1  (1.3, 3.2) | 3.8  (0.9, 7.7) | NA | NA | NA | NA | NA | NA | NA | NA | NA | NA | NA | NA | NA | NA | NA | NA | NA | NA | NA | NA | NA | NA | NA | NA | NA | NA | NA | NA | NA | NA | NA | NA | NA | NA | NA | NA |
|  | 2.2 Hodgkin lymphoma | 45/11 | 4.1  (3.0, 5.6) | 8.8  (5.6, 12.7) | NA | NA | NA | 12/1 | 12.1  (6.3, 21.1) | 2.8  (1.3, 5.1) | NA | NA | NA | 11/4 | 2.8  (1.4, 5.0) | 1.8  (0.4, 4.0) | 11/4 | 3.1  (1.5, 5.6) | 1.9  (0.5, 4.1) | 10/3 | 2.9  (1.4, 5.3) | 1.7  (0.3, 3.8) | NA | NA | NA | NA | NA | NA | NA | NA | NA | NA | NA | NA | NA | NA | NA | NA | NA | NA |
|  | 2.2.2 Hodgkin classic, other | 43/10 | 4.2  (3.1, 5.7) | 9.1  (5.8, 13.2) | NA | NA | NA | 12/1 | 12.9  (6.7, 22.5) | 3.1  (1.5, 5.5) | NA | NA | NA | 10/4 | 2.7  (1.3, 4.9) | 1.7  (0.3, 4.1) | 10/3 | 3.0  (1.4, 5.5) | 1.8  (0.4, 4.2) | NA | NA | NA | NA | NA | NA | NA | NA | NA | NA | NA | NA | NA | NA | NA | NA | NA | NA | NA | NA | NA |
|  | **4. Sarcomas** | **17/8** | **2.1  (1.2, 3.3)** | **3.5  (0.7, 7.5)** | **NA** | **NA** | **NA** | **NA** | **NA** | **NA** | **NA** | **NA** | **NA** | **NA** | **NA** | **NA** | **NA** | **NA** | **NA** | **NA** | **NA** | **NA** | **NA** | **NA** | **NA** | **NA** | **NA** | **NA** | **NA** | **NA** | **NA** | **NA** | **NA** | **NA** | **NA** | **NA** | **NA** | **NA** | **NA** | **NA** |
|  | **7. Gonadal and related tumours** | **71/43** | **1.6  (1.3, 2.1)** | **2.0  (0.9, 3.4)** | **NA** | **NA** | **NA** | **10/4** | **2.6  (1.2, 4.7)** | **0.4  (0.1, 1.1)** | **NA** | **NA** | **NA** | **18/16** | **1.1  (0.7, 1.8)** | **0.2  (-0.4, 0.9)** | **17/14** | **1.2  (0.7, 1.9)** | **0.2  (-0.3, 0.9)** | **17/14** | **1.2  (0.7, 2.0)** | **0.2  (-0.3, 1.0)** | **11/11** | **1.0  (0.5, 1.8)** | **0.0  (-0.4, 0.6)** | **11/10** | **1.1  (0.5, 1.9)** | **0.0  (-0.4, 0.7)** | **13/3** | **3.9  (2.1, 6.7)** | **0.7  (0.3, 1.4)** | **NA** | **NA** | **NA** | **NA** | **NA** | **NA** | **13/3** | **4.8  (2.6, 8.3)** | **0.7  (0.3, 1.4)** |
|  | 7.1 Testis | 68/42 | 1.6  (1.2, 2.0) | 1.9  (0.8, 3.3) | NA | NA | NA | NA | NA | NA | NA | NA | NA | 17/16 | 1.1  (0.6, 1.8) | 0.1  (-0.4, 0.9) | 16/14 | 1.1  (0.7, 1.9) | 0.1  (-0.4, 0.9) | 16/14 | 1.2  (0.7, 1.9) | 0.2  (-0.3, 0.9) | 10/11 | 0.9  (0.4, 1.7) | -0.1  (-0.5, 0.6) | 10/10 | 1.0  (0.5, 1.8) | 0.0  (-0.4, 0.6) | 16/3 | 6.3  (3.6, 10.2) | 5.9  (2.9, 10.2) | NA | NA | NA | NA | NA | NA | 13/3 | 4.9  (2.6, 8.4) | 0.8  (0.3, 1.5) |
|  | 7.1.1 Germ cell and trophoblastic | 66/42 | 1.6  (1.2, 2.0) | 1.8  (0.6, 3.1) | NA | NA | NA | NA | NA | NA | NA | NA | NA | 17/15 | 1.1  (0.6, 1.8) | 0.1  (-0.4, 0.9) | 16/14 | 1.1  (0.7, 1.9) | 0.2  (-0.4, 0.9) | 16/14 | 1.2  (0.7, 1.9) | 0.2  (-0.3, 0.9) | 10/11 | 0.9  (0.4, 1.7) | -0.1  (-0.5, 0.6) | 10/10 | 1.0  (0.5, 1.8) | 0.0  (-0.4, 0.6) | 13/3 | 4.0  (2.1, 6.8) | 0.7  (0.3, 1.4) | NA | NA | NA | NA | NA | NA | 13/3 | 4.9  (2.6, 8.5) | 0.8  (0.3, 1.5) |
|  | 7.1.1.1 Seminoma | 36/24 | 1.5  (1.0, 2.0) | 1.9  (0.1, 4.1) | NA | NA | NA | NA | NA | NA | NA | NA | NA | NA | NA | NA | NA | NA | NA | NA | NA | NA | NA | NA | NA | NA | NA | NA | 10/2 | 5.3 (2.5, 9.7) | 1.3  (0.5, 2.7) | NA | NA | NA | NA | NA | NA | 10/2 | 6.4  (3.1, 11.8) | 1.4  (0.5, 2.7) |
|  | 7.1.1.4 Teratoma | 11/7 | 1.7  (0.8, 3.0) | 2.0  (-0.5, 5.8) | NA | NA | NA | NA | NA | NA | NA | NA | NA | NA | NA | NA | NA | NA | NA | NA | NA | NA | NA | NA | NA | NA | NA | NA | NA | NA | NA | NA | NA | NA | NA | NA | NA | NA | NA | NA |
|  | **8. Melanoma, malignant** | **18/26** | **0.7  (0.4, 1.1)** | **-1.2  (-2.3, 0.4)** | **NA** | **NA** | **NA** | **NA** | **NA** | **NA** | **NA** | **NA** | **NA** | **NA** | **NA** | **NA** | **NA** | **NA** | **NA** | **NA** | **NA** | **NA** | **NA** | **NA** | **NA** | **NA** | **NA** | **NA** | **NA** | **NA** | **NA** | **NA** | **NA** | **NA** | **NA** | **NA** | **NA** | **NA** | **NA** | **NA** |
|  | 8.1 Superficial spreading/low cumulative sun damage melanoma | 11/15 | 0.7  (0.4, 1.3) | -1.1  (-2.5, 1.1) | NA | NA | NA | NA | NA | NA | NA | NA | NA | NA | NA | NA | NA | NA | NA | NA | NA | NA | NA | NA | NA | NA | NA | NA | NA | NA | NA | NA | NA | NA | NA | NA | NA | NA | NA | NA |
|  | **9. Carcinomas** | **131/29** | **4.5  (3.8, 5.3)** | **16.1  (12.7, 19.9)** | **12/3** | **3.5  (1.8, 6.2)** | **1.4  (0.4, 2.8)** | **NA** | **NA** | **NA** | **NA** | **NA** | **NA** | **53/11** | **5.0  (3.7, 6.5)** | **6.7  (4.6, 9.3)** | **52/10** | **5.3  (3.9, 6.9)** | **6.7  (4.6, 9.2)** | **52/10** | **5.4  (4.0, 7.1)** | **6.7  (4.6, 9.2)** | **24/8** | **3.2  (2.0, 4.8)** | **2.6  (1.2, 4.5)** | **23/7** | **3.3  (2.1, 4.9)** | **2.5  (1.2, 4.3)** | **13/2** | **5.6  (3.0, 9.7)** | **1.7  (0.7, 3.1)** | **NA** | **NA** | **NA** | **NA** | **NA** | **NA** | **NA** | **NA** | **NA** |
|  | 9.2 Other carcinoma of head and neck | 12/5 | 2.4  (1.2, 4.1) | 6.4  (1.0, 14.6) | NA | NA | NA | NA | NA | NA | NA | NA | NA | NA | NA | NA | NA | NA | NA | NA | NA | NA | NA | NA | NA | NA | NA | NA | NA | NA | NA | NA | NA | NA | NA | NA | NA | NA | NA | NA |
|  | 9.3 Carcinoma of gastrointestinal tract | 85/10 | 8.1  (6.5, 10.1) | 32.6  (25.1, 41.3) | NA | NA | NA | NA | NA | NA | NA | NA | NA | 42/4 | 11.0  (7.9, 14.9) | 16.7  (11.5, 23.1) | 41/4 | 11.6  (8.3, 15.7) | 16.4  (11.3, 22.7) | 41/3 | 11.8  (8.5, 16.0) | 16.4  (11.3, 22.8) | 17/3 | 6.3  (3.7, 10.1) | 6.2  (3.1, 10.7) | 16/3 | 6.3  (3.6, 10.2) | 5.9  (2.9, 10.2) | NA | NA | NA | NA | NA | NA | NA | NA | NA | NA | NA | NA |
|  | 9.3.4 Carcinoma of colon | 60/5 | 11.1  (8.5, 14.3) | 44.7  (33.1, 58.9) | NA | NA | NA | NA | NA | NA | NA | NA | NA | 28/2 | 14.2  (9.4, 20.5) | 21.3  (13.6, 31.6) | 28/2 | 15.3  (10.2, 22.1) | 21.5  (13.8, 31.7) | 28/2 | 15.6  (10.4, 22.6) | 21.5  (13.8, 31.7) | 14/1 | 10.0  (5.5, 16.8) | 10.3  (5.1, 18.1) | 13/1 | 9.9  (5.3, 16.9) | 9.6  (4.6, 17.1) | NA | NA | NA | NA | NA | NA | NA | NA | NA | NA | NA | NA |
|  | 9.3.4.2 Colon excluding appendix | 57/4 | 12.8  (9.7, 16.6) | 61.4  (45.2, 81.0) | NA | NA | NA | NA | NA | NA | NA | NA | NA | 26/2 | 16.0  (10.5, 23.5) | 28.5  (17.9, 42.6) | 26/2 | 17.2  (11.2, 25.2) | 28.6  (18.1, 42.7) | 26/1 | 17.5  (11.4, 25.7) | 28.6  (18.1, 42.7) | 14/1 | 12.2  (6.7, 20.5) | 15.0  (7.6, 26.1) | 13/1 | 12.0  (6.4, 20.5) | 13.9  (6.8, 24.7) | NA | NA | NA | NA | NA | NA | NA | NA | NA | NA | NA | NA |
|  | 9.3.4.2.2 Colon excluding appendix, adenocarcinoma | 56/4 | 12.8  (9.7, 16.6) | 61.8  (45.4, 81.9) | NA | NA | NA | NA | NA | NA | NA | NA | NA | 26/2 | 16.3  (10.7, 23.9) | 29.2  (18.4, 43.7) | 26/1 | 17.5  (11.4, 25.6) | 29.4 (18.6, 43.9) | 26/1 | 17.8 (11.6, 26.1) | 29.4  (18.6, 43.9) | 14/1 | 12.4  (6.8, 20.8) | 15.4  (7.8, 26.8) | 13/1 | 12.2  (6.5, 20.8) | 14.3  (7.0, 25.4) | NA | NA | NA | NA | NA | NA | NA | NA | NA | NA | NA | NA |
|  | 9.3.5 Carcinoma of rectum | 14/3 | 5.2  (2.8, 8.7) | 19.6  (8.6, 36.2) | NA | NA | NA | NA | NA | NA | NA | NA | NA | 10/1 | 10.0  (4.8, 18.4) | 15.7  (6.6, 30.3) | 10/1 | 10.7  (5.2, 19.8) | 15.8  (6.7, 30.4) | 10/1 | 11.0  (5.3, 20.2) | 15.8  (6.8, 30.4) | NA | NA | NA | NA | NA | NA | NA | NA | NA | NA | NA | NA | NA | NA | NA | NA | NA | NA |
|  | 9.3.5.2 Rectum, adenocarcinoma | 13/2 | 5.3  (2.8, 9.0) | 20.7  (8.8, 38.8) | NA | NA | NA | NA | NA | NA | NA | NA | NA | 10/1 | 11.0  (5.3, 20.2) | 17.9  (7.6, 34.3) | 10/1 | 11.8  (5.7, 21.7) | 18 (7.8, 34.5) | 10/1 | 12.1 (5.8, 22.2) | 18.0  (7.8, 34.5) | NA | NA | NA | NA | NA | NA | NA | NA | NA | NA | NA | NA | NA | NA | NA | NA | NA | NA |
|  | 9.5 Carcinoma of skin (if collected) | 11/3 | 4.3  (2.1, 7.6) | 15.9  (5.5, 32.3) | NA | NA | NA | NA | NA | NA | NA | NA | NA | NA | NA | NA | NA | NA | NA | NA | NA | NA | NA | NA | NA | NA | NA | NA | NA | NA | NA | NA | NA | NA | NA | NA | NA | NA | NA | NA |
|  | 9.8 Carcinoma of urinary tract | 13/4 | 2.9  (1.6, 5.0) | 10.4  (3.0, 21.7) | NA | NA | NA | NA | NA | NA | NA | NA | NA | NA | NA | NA | NA | NA | NA | NA | NA | NA | NA | NA | NA | NA | NA | NA | NA | NA | NA | NA | NA | NA | NA | NA | NA | NA | NA | NA |
| **Females** | **2. Lymphomas** | **27/12** | **2.3  (1.5, 3.4)** | **2.9  (1.2, 5.3)** | **NA** | **NA** | **NA** | **NA** | **NA** | **NA** | **NA** | **NA** | **NA** | **NA** | **NA** | **NA** | **NA** | **NA** | **NA** | **NA** | **NA** | **NA** | **NA** | **NA** | **NA** | **NA** | **NA** | **NA** | **NA** | **NA** | **NA** | **NA** | **NA** | **NA** | **NA** | **NA** | **NA** | **NA** | **NA** | **NA** |
|  | 2.2 Hodgkin lymphoma | 16/6 | 2.8  (1.6, 4.5) | 3.1  (1.0, 6.1) | NA | NA | NA | NA | NA | NA | NA | NA | NA | NA | NA | NA | NA | NA | NA | NA | NA | NA | NA | NA | NA | NA | NA | NA | NA | NA | NA | NA | NA | NA | NA | NA | NA | NA | NA | NA |
|  | 2.2.2 Hodgkin classic, other | 16/6 | 2.8  (1.6, 4.6) | 3.2  (1.1, 6.3) | NA | NA | NA | NA | NA | NA | NA | NA | NA | NA | NA | NA | NA | NA | NA | NA | NA | NA | NA | NA | NA | NA | NA | NA | NA | NA | NA | NA | NA | NA | NA | NA | NA | NA | NA | NA |
|  | **4. Sarcomas** | **12/6** | **1.9  (1.0, 3.4)** | **2.4  (0.0, 6.1)** | **NA** | **NA** | **NA** | **NA** | **NA** | **NA** | **NA** | **NA** | **NA** | **NA** | **NA** | **NA** | **NA** | **NA** | **NA** | **NA** | **NA** | **NA** | **NA** | **NA** | **NA** | **NA** | **NA** | **NA** | **NA** | **NA** | **NA** | **NA** | **NA** | **NA** | **NA** | **NA** | **NA** | **NA** | **NA** | **NA** |
|  | **7. Gonadal and related tumours** | **12/10** | **1.2  (0.6, 2.2)** | **0.8  (-1.2, 3.9)** | **NA** | **NA** | **NA** | **NA** | **NA** | **NA** | **NA** | **NA** | **NA** | **NA** | **NA** | **NA** | **NA** | **NA** | **NA** | **NA** | **NA** | **NA** | **NA** | **NA** | **NA** | **NA** | **NA** | **NA** | **NA** | **NA** | **NA** | **NA** | **NA** | **NA** | **NA** | **NA** | **NA** | **NA** | **NA** | **NA** |
|  | 7.2 Ovary | 12/9 | 1.3  (0.7, 2.3) | 1.2  (-1.1, 4.6) | NA | NA | NA | NA | NA | NA | NA | NA | NA | NA | NA | NA | NA | NA | NA | NA | NA | NA | NA | NA | NA | NA | NA | NA | NA | NA | NA | NA | NA | NA | NA | NA | NA | NA | NA | NA |
|  | 7.2.2 Non-germ cell | 12/8 | 1.5  (0.8, 2.6) | 1.7  (-0.9, 5.9) | NA | NA | NA | NA | NA | NA | NA | NA | NA | NA | NA | NA | NA | NA | NA | NA | NA | NA | NA | NA | NA | NA | NA | NA | NA | NA | NA | NA | NA | NA | NA | NA | NA | NA | NA | NA |
|  | 7.2.2.1 Carcinoma | 12/8 | 1.5 (0.8, 2.7) | 2 (-0.8, 6.3) | NA | NA | NA | NA | NA | NA | NA | NA | NA | NA | NA | NA | NA | NA | NA | NA | NA | NA | NA | NA | NA | NA | NA | NA | NA | NA | NA | NA | NA | NA | NA | NA | NA | NA | NA | NA |
|  | 7.2.2.1.1 Adenocarcinoma | 12/8 | 1.6 (0.8, 2.7) | 2.1 (-0.7, 6.5) | NA | NA | NA | NA | NA | NA | NA | NA | NA | NA | NA | NA | NA | NA | NA | NA | NA | NA | NA | NA | NA | NA | NA | NA | NA | NA | NA | NA | NA | NA | NA | NA | NA | NA | NA | NA |
|  | 7.2.2.1.1.2 Cystadenocarcinoma | 10/6 | 1.6 (0.8, 3.0) | 2.4 (-0.8, 7.5) | NA | NA | NA | NA | NA | NA | NA | NA | NA | NA | NA | NA | NA | NA | NA | NA | NA | NA | NA | NA | NA | NA | NA | NA | NA | NA | NA | NA | NA | NA | NA | NA | NA | NA | NA | NA |
|  | **8. Melanoma, malignant** | **38/39** | **1.0  (0.7, 1.4)** | **0.0  (-0.9, 1.0)** | **NA** | **NA** | **NA** | **NA** | **NA** | **NA** | **NA** | **NA** | **NA** | **16/18** | **0.9  (0.5, 1.5)** | **-0.1  (-0.7, 0.6)** | **14/16** | **0.9  (0.5, 1.5)** | **-0.1  (-0.6, 0.6)** | **14/15** | **0.9  (0.5, 1.5)** | **-0.1  (-0.6, 0.6)** | **NA** | **NA** | **NA** | **NA** | **NA** | **NA** | **NA** | **NA** | **NA** | **NA** | **NA** | **NA** | **NA** | **NA** | **NA** | **NA** | **NA** | **NA** |
|  | 8.1 Superficial spreading/low cumulative sun damage melanoma | 23/24 | 0.9  (0.6, 1.4) | -0.2  (-1.1, 1.2) | NA | NA | NA | NA | NA | NA | NA | NA | NA | 10/11 | 0.9  (0.4, 1.6) | -0.1  (-0.7, 0.8) | 10/10 | 1.0  (0.5, 1.9) | 0.0  (-0.6, 1.0) | 10/10 | 1.0  (0.5, 1.9) | 0.0  (-0.6, 1.0) | NA | NA | NA | NA | NA | NA | NA | NA | NA | NA | NA | NA | NA | NA | NA | NA | NA | NA |
|  | **9. Carcinomas** | **220/131** | **1.7  (1.5, 1.9)** | **2.5  (1.7, 3.4)** | **19/5** | **3.9  (2.3, 6.1)** | **0.4  (0.2, 0.7)** | **17/9** | **1.9  (1.1, 3.0)** | **0.2  (0.0, 0.5)** | **13/7** | **2.0  (1.1, 3.4)** | **0.2  (0.0, 0.4)** | **86/60** | **1.4  (1.1, 1.8)** | **0.7  (0.2, 1.3)** | **83/54** | **1.5  (1.2, 1.9)** | **0.8  (0.3, 1.4)** | **82/53** | **1.5  (1.2, 1.9)** | **0.8  (0.3, 1.4)** | **46/30** | **1.5  (1.1, 2.0)** | **0.4  (0.1, 0.9)** | **45/29** | **1.5  (1.1, 2.1)** | **0.5  (0.1, 0.9)** | **28/13** | **2.2  (1.4, 3.1)** | **0.4  (0.2, 0.8)** | **10/2** | **4.3  (2.1, 7.9)** | **0.2  (0.1, 0.5)** | **10/2** | **5.6  (2.7, 10.3)** | **0.2  (0.1, 0.5)** | **17/10** | **1.6  (1.0, 2.6)** | **0.2  (0.0, 0.5)** |
|  | 9.1 Thyroid carcinoma | 16/9 | 1.7  (1.0, 2.8) | 2.1  (0.0, 5.2) | NA | NA | NA | NA | NA | NA | NA | NA | NA | NA | NA | NA | NA | NA | NA | NA | NA | NA | NA | NA | NA | NA | NA | NA | NA | NA | NA | NA | NA | NA | NA | NA | NA | NA | NA | NA |
|  | 9.3 Carcinoma of gastrointestinal tract | 43/8 | 5.3  (3.9, 7.2) | 13.8  (9.1, 19.7) | NA | NA | NA | NA | NA | NA | NA | NA | NA | 16/4 | 4.3  (2.5, 7.0) | 4.9  (2.2, 8.8) | 16/3 | 4.9  (2.8, 7.9) | 5.0  (2.3, 9.0) | 16/3 | 5.0  (2.8, 8.0) | 5.0 (2.3, 9.0) | 10/2 | 5.4  (2.6, 10.0) | 3.2  (1.2, 6.5) | NA | NA | NA | NA | NA | NA | NA | NA | NA | NA | NA | NA | NA | NA | NA |
|  | 9.3.4 Carcinoma of colon | 30/5 | 6.5  (4.4, 9.3) | 16.8  (10.3, 25.3) | NA | NA | NA | NA | NA | NA | NA | NA | NA | 11/2 | 5.2  (2.6, 9.3) | 5.9  (2.2, 11.6) | 11/2 | 5.9  (2.9, 10.5) | 6.0  (2.4, 11.8) | 11/2 | 6.0  (3.0, 10.7) | 6.1  (2.4, 11.8) | NA | NA | NA | NA | NA | NA | NA | NA | NA | NA | NA | NA | NA | NA | NA | NA | NA | NA |
|  | 9.3.4.2 Colon excluding appendix | 28/3 | 9.1 (6.0, 13.2) | 30.6 (19.1, 46.0) | NA | NA | NA | NA | NA | NA | NA | NA | NA | 11/1 | 7.8  (3.9, 14.0) | 11.8  (5.0, 22.5) | 11/1 | 8.6  (4.3, 15.4) | 12.0  (5.2, 22.6) | 11/1 | 8.8  (4.4, 15.7) | 12.0  (5.2, 22.7) | NA | NA | NA | NA | NA | NA | NA | NA | NA | NA | NA | NA | NA | NA | NA | NA | NA | NA |
|  | 9.3.4.2.2 Colon excluding appendix, adenocarcinoma | 27/3 | 8.9 (5.9, 13.0) | 30.3 (18.7, 45.9) | NA | NA | NA | NA | NA | NA | NA | NA | NA | 11/1 | 7.9  (4.0, 14.2) | 12.2  (5.2, 23.2) | 11/1 | 8.8  (4.4, 15.7) | 12.3  (5.4, 23.3) | 11/1 | 8.9  (4.5, 16.0) | 12.4  (5.4, 23.4) | NA | NA | NA | NA | NA | NA | NA | NA | NA | NA | NA | NA | NA | NA | NA | NA | NA | NA |
|  | 9.6 Carcinoma of breast | 89/71 | 1.2  (1.0, 1.5) | 1.0  (0.0, 2.1) | 11/3 | 4.1  (2.0, 7.3) | 0.4  (0.2, 0.9) | NA | NA | NA | NA | NA | NA | 36/32 | 1.1  (0.8, 1.5) | 0.2  (-0.4, 0.9) | 35/29 | 1.2  (0.8, 1.7) | 0.3  (-0.3, 1.0) | 34/29 | 1.2  (0.8, 1.6) | 0.3  (-0.3, 1.0) | 18/16 | 1.1  (0.7, 1.7) | 0.1  (-0.3, 0.6) | 18/16 | 1.1  (0.7, 1.8) | 0.1  (-0.3, 0.7) | 12/7 | 1.7  (0.9, 3.0) | 0.3  (0.0, 0.7) | NA | NA | NA | NA | NA | NA | NA | NA | NA |
|  | 9.6.1 Breast, infiltrating duct | 65/54 | 1.2  (0.9, 1.5) | 0.7  (-0.3, 1.9) | NA | NA | NA | NA | NA | NA | NA | NA | NA | 28/25 | 1.1  (0.7, 1.6) | 0.2  (-0.4, 1.1) | 28/22 | 1.2  (0.8, 1.8) | 0.4  (-0.3, 1.2) | 27/22 | 1.2  (0.8, 1.8) | 0.3  (-0.3, 1.2) | 12/13 | 1.0  (0.5, 1.7) | 0.0  (-0.4, 0.6) | 12/12 | 1.0  (0.5, 1.7) | 0.0  (-0.4, 0.6) | NA | NA | NA | NA | NA | NA | NA | NA | NA | NA | NA | NA |
|  | 9.6.2 Breast, adenocarcinoma | 12/7 | 1.6  (0.8, 2.8) | 2.8  (-0.7, 8.1) | NA | NA | NA | NA | NA | NA | NA | NA | NA | NA | NA | NA | NA | NA | NA | NA | NA | NA | NA | NA | NA | NA | NA | NA | NA | NA | NA | NA | NA | NA | NA | NA | NA | NA | NA | NA |
|  | 9.7 Carcinoma of genital sites excluding ovary and testis | 51/34 | 1.5  (1.1, 2.0) | 2.1  (0.5, 4.0) | NA | NA | NA | NA | NA | NA | NA | NA | NA | 18/15 | 1.2  (0.7, 1.8) | 0.3  (-0.6, 1.6) | 16/14 | 1.1  (0.7, 1.9) | 0.2  (-0.6, 1.4) | 16/14 | 1.2  (0.7, 1.9) | 0.3  (-0.5, 1.5) | 14/8 | 1.8  (1.0, 3.0) | 0.8  (0.0, 1.9) | 14/7 | 1.9  (1.0, 3.2) | 0.8  (0.0, 1.9) | 10/3 | 3.0  (1.5, 5.6) | 0.8  (0.2, 1.8) | NA | NA | NA | NA | NA | NA | NA | NA | NA |
|  | 9.7.1 Carcinoma of uterine cervix | 42/30 | 1.4 (1.0, 1.9) | 1.6 (0.0, 3.5) | NA | NA | NA | NA | NA | NA | NA | NA | NA | 13/14 | 0.9  (0.5, 1.6) | -0.1  (-0.9, 1.1) | 12/13 | 1.0  (0.5, 1.7) | -0.1  (-0.8, 1.1) | 12/12 | 1.0  (0.5, 1.7) | 0.0  (-0.8, 1.1) | 12/7 | 1.7  (0.9, 3.0) | 0.7  (-0.1, 1.9) | 12/7 | 1.8  (0.9, 3.1) | 0.7  (-0.1, 1.9) | NA | NA | NA | NA | NA | NA | NA | NA | NA | NA | NA | NA |
|  | 9.7.1.1 Cervix, squamous | 29/21 | 1.4 (0.9, 2.0) | 1.4 (-0.4, 3.8) | NA | NA | NA | NA | NA | NA | NA | NA | NA | 10/10 | 1.0  (0.5, 1.9) | 0.0  (-0.9, 1.6) | NA | NA | NA | NA | NA | NA | NA | NA | NA | NA | NA | NA | NA | NA | NA | NA | NA | NA | NA | NA | NA | NA | NA | NA |
|  | 9.7.1.3 Cervix, adenocarcinoma | 11/5 | 2.1 (1.0, 3.7) | 4.0 (0.2, 10.1) | NA | NA | NA | NA | NA | NA | NA | NA | NA | NA | NA | NA | NA | NA | NA | NA | NA | NA | NA | NA | NA | NA | NA | NA | NA | NA | NA | NA | NA | NA | NA | NA | NA | NA | NA | NA |

Abbreviation: NET=Neuroendocrine tumour, NA=Not Applicable.

**Supplementary Table S14A.** Standardised incidence ratios (SIRs) and absolute excess risks (AERs) of second primary carcinomas of the lung, bronchus, and trachea after each distinct first primary cancer type among six-month adolescent and young adult (AYA, aged 15-39 years) cancer survivors compared to the general population in the Netherlands. Second primary cancer types are grouped according to the AYA-specific classification scheme developed by Barr and colleagues (2020). First and second cancer combinations with less than n=10 observed second cancers were excluded from the analyses. This Table presents outcomes from the main analysis.

|  | **Second primary cancers** | **9.4 Carcinoma of lung, bronchus, and trachea** | | | **9.4.1 Small cell carcinoma, NEC** | | | **9.4.2 Non-small cell carcinoma** | | | **9.4.2.1 Non-small cell, adenocarcinoma** | | | **9.4.2.3 Non-small cell, other** | | |
| --- | --- | --- | --- | --- | --- | --- | --- | --- | --- | --- | --- | --- | --- | --- | --- | --- |
|  | **First primary cancers** | **Obs/exp** | **SIR (95%CI)** | **AER per 10,000 person-years (95%CI)** | **Obs/exp** | **SIR (95%CI)** | **AER per 10,000 person-years (95%CI)** | **Obs/exp** | **SIR (95%CI)** | **AER per 10,000 person-years (95%CI)** | **Obs/exp** | **SIR (95%CI)** | **AER per 10,000 person-years (95%CI)** | **Obs/exp** | **SIR (95%CI)** | **AER per 10,000 person-years (95%CI)** |
| **Males** | **2. Lymphomas** | **42/8** | **5.1 (3.7, 6.9)** | **4.8 (3.1, 6.9)** | **NA** | **NA** | **NA** | **35/7** | **4.8 (3.3, 6.7)** | **3.9 (2.4, 5.9)** | **23/5** | **5.0 (3.2, 7.5)** | **2.6 (1.4, 4.3)** | **12/2** | **5.2 (2.7, 9.1)** | **1.4 (0.6, 2.7)** |
|  | 2.1 Non-Hodgkin lymphomas | 13/4 | 3.6 (1.9, 6.2) | 3.4 (1.2, 6.8) | **NA** | **NA** | **NA** | **NA** | **NA** | **NA** | **NA** | **NA** | **NA** | **NA** | **NA** | **NA** |
|  | 2.2 Hodgkin lymphoma | 28/4 | 7.2 (4.8, 10.4) | 6.2 (3.8, 9.4) | **NA** | **NA** | **NA** | 26/3 | 7.5 (4.9, 10.9) | 5.8 (3.5, 8.9) | 18/2 | 8.2 (4.9, 13.0) | 4.1 (2.2, 6.7) | **NA** | **NA** | **NA** |
|  | 2.2.2 Hodgkin classic, other | 27/4 | 7.4 (4.9, 10.7) | 6.4 (3.9, 9.8) | **NA** | **NA** | **NA** | 25/3 | 7.6 (4.9, 11.3) | 6.0 (3.6, 9.3) | 17/2 | 8.2 (4.8, 13.2) | 4.1 (2.2, 6.9) | **NA** | **NA** | **NA** |
|  | **7. Gonadal and related tumours** | **27/16** | **1.7 (1.1, 2.5)** | **0.8 (0.1, 1.7)** | **NA** | **NA** | **NA** | **21/14** | **1.5 (0.9, 2.3)** | **0.5 (-0.1, 1.3)** | **15/9** | **1.7 (0.9, 2.7)** | **0.4 (0.0, 1.1)** | **NA** | **NA** | **NA** |
|  | 7.1 Testis | 26/16 | 1.7 (1.1, 2.5) | 0.8 (0.1, 1.6) | **NA** | **NA** | **NA** | 20/14 | 1.4 (0.9, 2.2) | 0.4 (-0.1, 1.2) | 14/9 | 1.6 (0.9, 2.6) | 0.4 (-0.1, 1.1) | **NA** | **NA** | **NA** |
|  | 7.1.1 Germ cell and trophoblastic | 26/16 | 1.7 (1.1, 2.5) | 0.8 (0.1, 1.7) | **NA** | **NA** | **NA** | 20/14 | 1.4 (0.9, 2.2) | 0.4 (-0.1, 1.2) | 14/9 | 1.6 (0.9, 2.6) | 0.4 (-0.1, 1.1) | **NA** | **NA** | **NA** |
|  | 7.1.1.1 Seminoma | 15/9 | 1.6 (0.9, 2.7) | 0.9 (-0.1, 2.5) | **NA** | **NA** | **NA** | 13/8 | 1.6 (0.8, 2.7) | 0.8 (-0.2, 2.2) | **NA** | **NA** | **NA** | **NA** | **NA** | **NA** |
|  | **8. Melanoma, malignant** | **10/10** | **1.0 (0.5, 1.9)** | **0.0 (-0.8, 1.3)** | **NA** | **NA** | **NA** | **10/9** | **1.1 (0.5, 2.1)** | **0.2 (-0.6, 1.4)** | **NA** | **NA** | **NA** | **NA** | **NA** | **NA** |
|  | **9. Carcinomas** | **46/11** | **4.0 (3.0, 5.4)** | **5.4 (3.5, 7.8)** | **NA** | **NA** | **NA** | **42/10** | **4.1 (3.0, 5.6)** | **5.0 (3.1, 7.3)** | **18/6** | **2.8 (1.7, 4.5)** | **1.8 (0.7, 3.4)** | **24/3** | **7.3 (4.7, 10.9)** | **3.2 (1.9, 5.1)** |
|  | 9.2 Other carcinoma of head and neck | 12/2 | 6.0 (3.1, 10.4) | 9.1 (3.8, 17.3) | **NA** | **NA** | **NA** | 11/2 | 6.1 (3.1, 11.0) | 8.4 (3.4, 16.3) | **NA** | **NA** | **NA** | **NA** | **NA** | **NA** |
|  | 9.3 Carcinoma of gastrointestinal tract | 15/4 | 3.7 (2.1, 6.1) | 4.7 (1.9, 8.9) | **NA** | **NA** | **NA** | 15/4 | 4.1 (2.3, 6.8) | 4.9 (2.1, 9.1) | 10/2 | 4.4 (2.1, 8.1) | 3.3 (1.1, 7.0) | **NA** | **NA** | **NA** |
| **Females** | **2. Lymphomas** | **39/6** | **6.1 (4.3, 8.4)** | **6.2 (4.1, 8.9)** | **NA** | **NA** | **NA** | **35/6** | **6.2 (4.3, 8.7)** | **5.6 (3.6, 8.2)** | **25/4** | **6.2 (4.0, 9.1)** | **4.0 (2.3, 6.2)** | **10/1** | **9.1 (4.3, 16.7)** | **1.7 (0.7, 3.3)** |
|  | 2.1 Non-Hodgkin lymphomas | 14/3 | 4.9 (2.7, 8.2) | 6.4 (2.8, 11.9) | **NA** | **NA** | **NA** | 12/3 | 4.8 (2.5, 8.4) | 5.5 (2.1, 10.6) | **NA** | **NA** | **NA** | **NA** | **NA** | **NA** |
|  | 2.2 Hodgkin lymphoma | 25/3 | 8.4 (5.4, 12.3) | 6.7 (4.0, 10.3) | **NA** | **NA** | **NA** | 23/3 | 8.7 (5.5, 13.0) | 6.2 (3.6, 9.6) | 17/2 | 8.9 (5.2, 14.2) | 4.6 (2.4, 7.6) | **NA** | **NA** | **NA** |
|  | 2.2.2 Hodgkin classic, other | 24/3 | 8.2 (5.3, 12.2) | 6.5 (3.9, 10.1) | **NA** | **NA** | **NA** | 22/3 | 8.5 (5.3, 12.8) | 6.0 (3.5, 9.5) | 16/2 | 8.5 (4.9, 13.8) | 4.4 (2.2, 7.5) | **NA** | **NA** | **NA** |
|  | **7. Gonadal and related tumours** | **19/6** | **3.3 (2.0, 5.1)** | **4.6 (1.9, 8.3)** | **NA** | **NA** | **NA** | **18/5** | **3.5 (2.1, 5.6)** | **4.5 (1.9, 8.1)** | **12/4** | **3.3 (1.7, 5.7)** | **2.9 (0.9, 6.0)** | **NA** | **NA** | **NA** |
|  | 7.2 Ovary | 19/5 | 3.5 (2.1, 5.5) | 5.2 (2.3, 9.3) | **NA** | **NA** | **NA** | 18/5 | 3.8 (2.3, 6.0) | 5.1 (2.3, 9.1) | 12/3 | 3.5 (1.8, 6.1) | 3.3 (1.1, 6.7) | **NA** | **NA** | **NA** |
|  | 7.2.2 Non-germ cell | 17/5 | 3.4 (2.0, 5.4) | 5.5 (2.2, 10.1) | **NA** | **NA** | **NA** | 16/4 | 3.7 (2.1, 6.0) | 5.3 (2.2, 9.9) | 12/3 | 3.8 (2.0, 6.6) | 4.0 (1.4, 8.1) | **NA** | **NA** | **NA** |
|  | 7.2.2.1 Carcinoma | 17/5 | 3.5 (2.1, 5.7) | 5.8 (2.4, 10.6) | **NA** | **NA** | **NA** | 16/4 | 3.8 (2.2, 6.2) | 5.6 (2.4, 10.4) | 12/3 | 4.0 (2.1, 6.9) | 4.3 (1.5, 8.5) | **NA** | **NA** | **NA** |
|  | 7.2.2.1.1 Adenocarcinoma | 17/5 | 3.6 (2.1, 5.8) | 5.9 (2.5, 10.9) | **NA** | **NA** | **NA** | 16/4 | 3.9 (2.2, 6.3) | 5.8 (2.4, 10.6) | 12/3 | 4.0 (2.1, 7.0) | 4.4 (1.6, 8.7) | **NA** | **NA** | **NA** |
|  | 7.2.2.1.1.2 Cystadenocarcinoma | 14/4 | 3.8 (2.1, 6.3) | 6.2 (2.4, 11.9) | **NA** | **NA** | **NA** | 14/3 | 4.4 (2.4, 7.3) | 6.5 (2.7, 12.2) | 11/2 | 4.7 (2.4, 8.4) | 5.2 (1.9, 10.4) | **NA** | **NA** | **NA** |
|  | **8. Melanoma, malignant** | **36/23** | **1.6 (1.1, 2.2)** | **1.0 (0.2, 2.0)** | **NA** | **NA** | **NA** | **34/20** | **1.7 (1.2, 2.4)** | **1.0 (0.3, 2.0)** | **20/15** | **1.4 (0.8, 2.1)** | **0.4 (-0.2, 1.2)** | **11/4** | **2.8 (1.4, 5.0)** | **0.5 (0.1, 1.2)** |
|  | 8.1 Superficial spreading/low cumulative sun damage melanoma | 17/14 | 1.2 (0.7, 1.9) | 0.3 (-0.5, 1.5) | **NA** | **NA** | **NA** | 17/12 | 1.4 (0.8, 2.2) | 0.5 (-0.3, 1.7) | **NA** | **NA** | **NA** | **NA** | **NA** | **NA** |
|  | 8.3 Other malignant | 15/7 | 2.2 (1.2, 3.6) | 2.3 (0.4, 5.1) | **NA** | **NA** | **NA** | 13/6 | 2.1 (1.1, 3.7) | 2.0 (0.2, 4.7) | 10/4 | 2.3 (1.1, 4.2) | 1.6 (0.1, 4.0) | **NA** | **NA** | **NA** |
|  | **9. Carcinomas** | **227/84** | **2.7 (2.4, 3.1)** | **3.9 (3.2, 4.8)** | **32/11** | **2.9 (2.0, 4.1)** | **0.6 (0.3, 0.9)** | **195/73** | **2.7 (2.3, 3.1)** | **3.4 (2.6, 4.2)** | **126/53** | **2.4 (2.0, 2.9)** | **2.0 (1.4, 2.7)** | **63/15** | **4.1 (3.1, 5.2)** | **1.3 (0.9, 1.8)** |
|  | 9.2 Other carcinoma of head and neck | 10/2 | 6.2 (3.0, 11.5) | 9.8 (3.7, 19.6) | **NA** | **NA** | **NA** | **NA** | **NA** | **NA** | **NA** | **NA** | **NA** | **NA** | **NA** | **NA** |
|  | 9.3 Carcinoma of gastrointestinal tract | 10/5 | 2.1 (1.0, 3.9) | 2.1 (0.0, 5.4) | **NA** | **NA** | **NA** | **NA** | **NA** | **NA** | **NA** | **NA** | **NA** | **NA** | **NA** | **NA** |
|  | 9.6 Carcinoma of breast | 131/47 | 2.8 (2.3, 3.3) | 4.3 (3.2, 5.5) | 20/6 | 3.2 (1.9, 4.9) | 0.7 (0.3, 1.3) | 111/41 | 2.7 (2.2, 3.2) | 3.6 (2.6, 4.7) | 76/30 | 2.6 (2.0, 3.2) | 2.4 (1.5, 3.4) | 31/9 | 3.5 (2.4, 5.0) | 1.1 (0.6, 1.8) |
|  | 9.6.1 Breast, infiltrating duct | 107/36 | 3.0 (2.4, 3.6) | 4.6 (3.4, 6.1) | 15/5 | 3.2 (1.8, 5.3) | 0.7 (0.2, 1.3) | 92/31 | 2.9 (2.4, 3.6) | 3.9 (2.8, 5.3) | 61/23 | 2.7 (2.1, 3.5) | 2.5 (1.6, 3.6) | 28/7 | 4.2 (2.8, 6.1) | 1.4 (0.8, 2.2) |
|  | 9.6.2 Breast, adenocarcinoma | 13/5 | 2.6 (1.4, 4.4) | 4.5 (1.1, 9.8) | **NA** | **NA** | **NA** | 10/4 | 2.3 (1.1, 4.2) | 3.2 (0.2, 8.0) |  |  |  | **NA** | **NA** | **NA** |
|  | 9.7 Carcinoma of genital sites excluding ovary and testis | 58/20 | 2.8 (2.1, 3.7) | 4.5 (2.8, 6.6) | **NA** | **NA** | **NA** | 50/18 | 2.8 (2.1, 3.7) | 3.9 (2.3, 5.8) | 29/13 | 2.2 (1.5, 3.2) | 1.9 (0.8, 3.5) | 19/4 | 5.1 (3.1, 8.0) | 1.8 (0.9, 3.1) |
|  | 9.7.1 Carcinoma of uterine cervix | 54/18 | 2.9 (2.2, 3.8) | 4.7 (2.9, 6.9) | **NA** | **NA** | **NA** | 46/16 | 2.9 (2.1, 3.9) | 4.0 (2.4, 6.0) | 26/12 | 2.2 (1.5, 3.3) | 1.9 (0.7, 3.5) | 18/3 | 5.4 (3.2, 8.6) | 1.9 (1.0, 3.3) |
|  | 9.7.1.1 Cervix, squamous | 40/13 | 3.1 (2.2, 4.2) | 5.1 (2.9, 7.8) | **NA** | **NA** | **NA** | 33/11 | 2.9 (2.0, 4.1) | 4.1 (2.1, 6.6) | 19/8 | 2.3 (1.4, 3.6) | 2.0 (0.6, 4.0) | 13/2 | 5.5 (2.9, 9.5) | 2.0 (0.9, 3.7) |
|  | 9.7.1.3 Cervix, adenocarcinoma | 10/3 | 3.2 (1.5, 5.9) | 4.8 (1.2, 10.6) | **NA** | **NA** | **NA** | 10/3 | 3.7 (1.8, 6.8) | 5.1 (1.4, 10.9) | **NA** | **NA** | **NA** | **NA** | **NA** | **NA** |

Abbreviation: NEC=Neuroendocrine carcinoma.

**Supplementary Table S14B.** Standardised incidence ratios (SIRs) and absolute excess risks (AERs) of second primary carcinomas of the lung, bronchus, and trachea after each distinct first primary cancer type among six-month adolescent and young adult (AYA, aged 15-39 years) cancer survivors compared to the general population in the Netherlands. Second primary cancer types are grouped according to the AYA-specific classification scheme developed by Barr and colleagues (2020). First and second cancer combinations with less than n=10 observed second cancers were excluded from the analyses. This Table presents outcomes from the sensitivity analysis.

|  | **Second primary cancers** | **9.4 Carcinoma of lung, bronchus, and trachea** | | | **9.4.1 Small cell carcinoma, NEC** | | | **9.4.2 Non-small cell carcinoma** | | | **9.4.2.1 Non-small cell, adenocarcinoma** | | | **9.4.2.3 Non-small cell, other** | | |
| --- | --- | --- | --- | --- | --- | --- | --- | --- | --- | --- | --- | --- | --- | --- | --- | --- |
|  | **First primary cancers** | **Obs/exp** | **SIR (95%CI)** | **AER per 10,000 person-years (95%CI)** | **Obs/exp** | **SIR (95%CI)** | **AER per 10,000 person-years (95%CI)** | **Obs/exp** | **SIR (95%CI)** | **AER per 10,000 person-years (95%CI)** | **Obs/exp** | **SIR (95%CI)** | **AER per 10,000 person-years (95%CI)** | **Obs/exp** | **SIR (95%CI)** | **AER per 10,000 person-years (95%CI)** |
| **Males** | **2. Lymphomas** | **41/8** | **5.0 (3.6, 6.8)** | **4.7 (3.0, 6.8)** | **NA** | **NA** | **NA** | **34/7** | **4.7 (3.2, 6.5)** | **3.8 (2.3, 5.7)** | **22/5** | **4.8 (3.0, 7.3)** | **2.5 (1.3, 4.1)** | **12/2** | **5.2 (2.7, 9.2)** | **1.4 (0.6, 2.7)** |
|  | 2.1 Non-Hodgkin lymphomas | 12/4 | 3.3 (1.7, 5.8) | 3.1 (0.9, 6.3) | NA | NA | NA | 20/14 | 1.4 (0.9, 2.2) | 0.4 (-0.1, 1.2) | 14/9 | 1.6 (0.9, 2.6) | 0.4 (-0.1, 1.1) | NA | NA | NA |
|  | 2.2 Hodgkin lymphoma | 28/4 | 7.2 (4.8, 10.4) | 6.2 (3.8, 9.4) | NA | NA | NA | 45/10 | 4.5 (3.3, 6.1) | 5.5 (3.6, 7.9) | 17/6 | 2.7 (1.6, 4.4) | 1.7 (0.6, 3.3) | 27/3 | 8.4 (5.5, 12.2) | 3.8 (2.3, 5.7) |
|  | 2.2.2 Hodgkin classic, other | 27/4 | 7.4 (4.9, 10.7) | 6.4 (3.9, 9.8) | NA | NA | NA | NA | NA | NA | NA | NA | NA | NA | NA | NA |
|  | **7. Gonadal and related tumours** | **26/15** | **1.7 (1.1, 2.5)** | **0.8 (0.1, 1.6)** | **NA** | **NA** | **NA** | **26/3** | **7.5 (4.9, 10.9)** | **5.8 (3.5, 8.9)** | **18/2** | **8.2 (4.9, 13.0)** | **4.1 (2.2, 6.7)** | **NA** | **NA** | **NA** |
|  | 7.1 Testis | 25/15 | 1.6 (1.1, 2.4) | 0.7 (0.1, 1.6) | NA | NA | NA | 19/14 | 1.4 (0.8, 2.2) | 0.4 (-0.2, 1.2) | 13/9 | 1.5 (0.8, 2.6) | 0.3 (-0.1, 1.0) | NA | NA | NA |
|  | 7.1.1 Germ cell and trophoblastic | 25/15 | 1.6 (1.1, 2.4) | 0.7 (0.1, 1.6) | NA | NA | NA | 11/2 | 6.3 (3.1, 11.2) | 8.5 (3.4, 16.5) | NA | NA | NA | NA | NA | NA |
|  | 7.1.1.1 Seminoma | 14/9 | 1.6 (0.9, 2.6) | 0.8 (-0.2, 2.4) | NA | NA | NA | 14/4 | 3.9 (2.1, 6.6) | 4.6 (1.8, 8.7) | NA | NA | NA | NA | NA | NA |
|  | **9. Carcinomas** | **52/11** | **4.7 (3.5, 6.1)** | **6.4 (4.4, 9.0)** | **NA** | **NA** | **NA** | **25/3** | **7.6 (4.9, 11.3)** | **6.0 (3.6, 9.3)** | **17/2** | **8.2 (4.8, 13.2)** | **4.1 (2.2, 6.9)** | **NA** | **NA** | **NA** |
|  | 9.2 Other carcinoma of head and neck | 12/2 | 6.1 (3.2, 10.7) | 9.2 (3.9, 17.4) | NA | NA | NA | 19/14 | 1.4 (0.8, 2.2) | 0.4 (-0.2, 1.2) | 13/9 | 1.5 (0.8, 2.6) | 0.3 (-0.1, 1.0) | NA | NA | NA |
|  | 9.3 Carcinoma of gastrointestinal tract | 14/4 | 3.5 (1.9, 5.9) | 4.4 (1.6, 8.5) | NA | NA | NA | 12/8 | 1.5 (0.8, 2.6) | 0.6 (-0.3, 2.1) | NA | NA | NA | NA | NA | NA |
| **Females** | **2. Lymphomas** | **39/6** | **6.1 (4.4, 8.4)** | **6.2 (4.1, 8.9)** | **NA** | **NA** | **NA** | **35/6** | **6.3 (4.4, 8.7)** | **5.6 (3.6, 8.2)** | **25/4** | **6.2 (4.0, 9.1)** | **4.0 (2.3, 6.2)** | **10/1** | **9.1 (4.4, 16.7)** | **1.7 (0.7, 3.3)** |
|  | 2.1 Non-Hodgkin lymphomas | 14/3 | 4.9 (2.7, 8.2) | 6.4 (2.8, 11.9) | NA | NA | NA | 12/2 | 4.8 (2.5, 8.4) | 5.5 (2.1, 10.6) | NA | NA | NA | NA | NA | NA |
|  | 2.2 Hodgkin lymphoma | 25/3 | 8.4 (5.4, 12.3) | 6.7 (4.0, 10.3) | NA | NA | NA | 23/3 | 8.7 (5.5, 13.0) | 6.2 (3.6, 9.6) | 17/2 | 8.9 (5.2, 14.2) | 4.6 (2.4, 7.6) | NA | NA | NA |
|  | 2.2.2 Hodgkin classic, other | 24/3 | 8.2 (5.3, 12.2) | 6.5 (3.9, 10.1) | NA | NA | NA | 22/3 | 8.5 (5.3, 12.8) | 6.0 (3.5, 9.5) | 16/2 | 8.5 (4.9, 13.8) | 4.4 (2.2, 7.5) | NA | NA | NA |
|  | **7. Gonadal and related tumours** | **19/6** | **3.3 (2.0, 5.1)** | **4.6 (2.0, 8.3)** | **NA** | **NA** | **NA** | **18/5** | **3.6 (2.1, 5.6)** | **4.5 (2.0, 8.2)** | **12/4** | **3.3 (1.7, 5.7)** | **2.9 (0.9, 6.0)** | **NA** | **NA** | **NA** |
|  | 7.2 Ovary | 19/5 | 3.5 (2.1, 5.5) | 5.3 (2.3, 9.4) | NA | NA | NA | 18/5 | 3.8 (2.3, 6.1) | 5.1 (2.3, 9.2) | 12/3 | 3.5 (1.8, 6.2) | 3.3 (1.1, 6.8) | NA | NA | NA |
|  | 7.2.2 Non-germ cell | 17/5 | 3.4 (2.0, 5.5) | 5.5 (2.3, 10.2) | NA | NA | NA | 16/4 | 3.7 (2.1, 6.0) | 5.4 (2.2, 10.0) | 12/3 | 3.8 (2.0, 6.7) | 4.1 (1.4, 8.2) | NA | NA | NA |
|  | 7.2.2.1 Carcinoma | 17/5 | 3.6 (2.1, 5.7) | 5.9 (2.5, 10.7) | NA | NA | NA | 16/4 | 3.9 (2.2, 6.3) | 5.7 (2.4, 10.4) | 12/3 | 4.0 (2.1, 7.0) | 4.3 (1.5, 8.6) | NA | NA | NA |
|  | 7.2.2.1.1 Adenocarcinoma | 17/5 | 3.6 (2.1, 5.8) | 6.0 (2.5, 11.0) | NA | NA | NA | 16/4 | 3.9 (2.2, 6.4) | 5.8 (2.5, 10.7) | 12/3 | 4.1 (2.1, 7.1) | 4.4 (1.6, 8.8) | NA | NA | NA |
|  | 7.2.2.1.1.2 Cystadenocarcinoma | 14/4 | 3.8 (2.1, 6.4) | 6.3 (2.4, 12.0) | NA | NA | NA | 14/3 | 4.4 (2.4, 7.4) | 6.6 (2.7, 12.3) | 11/2 | 4.8 (2.4, 8.5) | 5.3 (1.9, 10.5) | NA | NA | NA |
|  | **8. Melanoma, malignant** | **33/22** | **1.5 (1.0, 2.1)** | **0.8 (0.1, 1.9)** | **NA** | **NA** | **NA** | **31/19** | **1.6 (1.1, 2.3)** | **0.9 (0.1, 1.9)** | **18/14** | **1.3 (0.8, 2.0)** | **0.3 (-0.3, 1.1)** | **10/4** | **2.6 (1.2, 4.8)** | **0.5 (0.1, 1.1)** |
|  | 8.1 Superficial spreading/low cumulative sun damage melanoma | 17/14 | 1.2 (0.7, 2.0) | 0.4 (-0.4, 1.6) | NA | NA | NA | 17/12 | 1.4 (0.8, 2.3) | 0.6 (-0.2, 1.8) | NA | NA | NA | NA | NA | NA |
|  | 8.3 Other malignant | 13/7 | 2.0 (1.0, 3.3) | 1.9 (0.1, 4.6) | NA | NA | NA | 11/6 | 1.9 (0.9, 3.4) | 1.5 (-0.1, 4.1) | NA | NA | NA | NA | NA | NA |
|  | **9. Carcinomas** | **225/80** | **2.8 (2.5, 3.2)** | **4.1 (3.3, 5.0)** | **30/10** | **2.9 (1.9, 4.1)** | **0.6 (0.3, 0.9)** | **195/70** | **2.8 (2.4, 3.2)** | **3.5 (2.8, 4.4)** | **124/50** | **2.5 (2.1, 2.9)** | **2.1 (1.5, 2.8)** | **64/15** | **4.3 (3.3, 5.5)** | **1.4 (1.0, 1.9)** |
|  | 9.2 Other carcinoma of head and neck | 10/2 | 6.4 (3.1, 11.7) | 9.9 (3.8, 19.8) | NA | NA | NA | NA | NA | NA | NA | NA | NA | NA | NA | NA |
|  | 9.3 Carcinoma of gastrointestinal tract | 10/5 | 2.1 (1.0, 3.9) | 2.1 (0.0, 5.4) | NA | NA | NA | NA | NA | NA | NA | NA | NA | NA | NA | NA |
|  | 9.4 Carcinoma of lung, bronchus, and trachea | 11/1 | 9.6 (4.8, 17.2) | 18.6 (8.2, 34.9) | NA | NA | NA | 11/1 | 11.1 (5.5, 19.9) | 18.9 (8.5, 35.2) | NA | NA | NA | NA | NA | NA |
|  | 9.4.2 Non-small cell carcinoma | 11/1 | 10.0 (5.0, 17.8) | 19.1 (8.5, 35.9) | NA | NA | NA | 11/1 | 11.5 (5.7, 20.5) | 19.4 (8.8, 36.2) | 57/21 | 2.7 (2.1, 3.6) | 2.5 (1.5, 3.6) | NA | NA | NA |
|  | 9.6 Carcinoma of breast | 123/44 | 2.8 (2.3, 3.3) | 4.3 (3.1, 5.5) | 18/6 | 3.1 (1.9, 4.9) | 0.7 (0.3, 1.2) | 105/38 | 2.8 (2.3, 3.3) | 3.6 (2.6, 4.8) | 71/27 | 2.6 (2.0, 3.3) | 2.3 (1.5, 3.3) | 30/8 | 3.6 (2.4, 5.2) | 1.2 (0.6, 1.9) |
|  | 9.6.1 Breast, infiltrating duct | 100/33 | 3.0 (2.5, 3.7) | 4.5 (3.3, 6.0) | 13/4 | 3.0 (1.6, 5.2) | 0.6 (0.2, 1.2) | 87/29 | 3.0 (2.4, 3.7) | 3.9 (2.8, 5.3) | 57/21 | 2.7 (2.1, 3.6) | 2.5 (1.5, 3.6) | 27/6 | 4.3 (2.9, 6.3) | 1.4 (0.8, 2.2) |
|  | 9.6.2 Breast, adenocarcinoma | 12/5 | 2.5 (1.3, 4.4) | 4.3 (0.9, 9.7) | NA | NA | NA | NA | NA | NA | NA | NA | NA | NA | NA | NA |
|  | 9.7 Carcinoma of genital sites excluding ovary and testis | 58/20 | 2.8 (2.2, 3.7) | 4.5 (2.8, 6.6) | NA | NA | NA | 50/18 | 2.8 (2.1, 3.7) | 3.9 (2.3, 5.8) | 29/13 | 2.2 (1.5, 3.2) | 1.9 (0.8, 3.5) | 19/4 | 5.1 (3.1, 8.0) | 1.8 (0.9, 3.1) |
|  | 9.7.1 Carcinoma of uterine cervix | 54/18 | 2.9 (2.2, 3.8) | 4.7 (2.9, 6.9) | NA | NA | NA | 46/16 | 2.9 (2.1, 3.9) | 4.0 (2.4, 6.0) | 26/12 | 2.2 (1.5, 3.3) | 1.9 (0.7, 3.5) | 18/3 | 5.4 (3.2, 8.6) | 1.9 (1.0, 3.3) |
|  | 9.7.1.1 Cervix, squamous | 40/13 | 3.1 (2.2, 4.2) | 5.1 (2.9, 7.8) | NA | NA | NA | 33/11 | 2.9 (2.0, 4.1) | 4.1 (2.1, 6.6) | 19/8 | 2.3 (1.4, 3.6) | 2.0 (0.6, 4.0) | 13/2 | 5.5 (2.9, 9.5) | 2.0 (0.9, 3.7) |
|  | 9.7.1.3 Cervix, adenocarcinoma | 10/3 | 3.2 (1.5, 5.9) | 4.8 (1.2, 10.7) | NA | NA | NA | 10/3 | 3.7 (1.8, 6.8) | 5.1 (1.4, 10.9) | NA | NA | NA | NA | NA | NA |

Abbreviation: NEC=Neuroendocrine carcinoma.

**Supplementary Table S15A.** Standardised incidence ratios (SIRs) and absolute excess risks (AERs) of second primary skin carcinomas after each distinct first primary cancer type among six-month adolescent and young adult (AYA, aged 15-39 years) cancer survivors compared to the general population in the Netherlands. Second primary cancer types are grouped according to the AYA-specific classification scheme developed by Barr and colleagues (2020). First and second cancer combinations with less than n=10 observed second cancers were excluded from the analyses. This Table presents outcomes from the main analysis.

|  | **Second primary cancers** | **9.5 Carcinoma of skin (if collected)** | | |
| --- | --- | --- | --- | --- |
|  | **First primary cancers** | **Obs/exp** | **SIR (95%CI)** | **AER per 10,000 person-years (95%CI)** |
| **Males** | **2. Lymphomas** | **22/5** | **4.3 (2.7, 6.5)** | **2.4 (1.2, 4.0)** |
|  | 2.1 Non-Hodgkin lymphomas | 13/2 | 5.8 (3.1, 9.9) | 3.9 (1.7, 7.3) |
|  | **8. Melanoma, malignant** | **22/6** | **3.5 (2.2, 5.3)** | **2.4 (1.1, 4.1)** |
|  | 8.1 Superficial spreading/low cumulative sun damage melanoma | 16/4 | 4.3 (2.5, 7.0) | 3.0 (1.3, 5.5) |
|  | **9. Carcinomas** | **33/7** | **4.7 (3.3, 6.7)** | **4.1 (2.5, 6.1)** |
|  | 9.2 Other carcinoma of head and neck | 12/1 | 9.9 (5.1, 17.3) | 9.8 (4.5, 18.0) |
|  | 9.2.2 Oral cavity, lip, and pharynx | 10/1 | 14.7 (7.1, 27.1) | 16.4 (7.2, 31.2) |
|  | 9.2.2.1 Oral cavity, lip, and pharynx, squamous | 10/1 | 18.3 (8.8, 33.6) | 21.1 (9.5, 39.9) |
|  | 9.3 Carcinoma of gastrointestinal tract | 15/3 | 6.0 (3.3, 9.9) | 5.4 (2.5, 9.6) |
|  | 9.3.4 Carcinoma of colon | 10/1 | 7.5 (3.6, 13.8) | 7.0 (2.8, 13.7) |
| **Females** | **8. Melanoma, malignant** | **40/14** | **2.9 (2.1, 3.9)** | **1.9 (1.1, 3.0)** |
|  | 8.1 Superficial spreading/low cumulative sun damage melanoma | 28/9 | 3.2 (2.2, 4.7) | 2.2 (1.1, 3.6) |
|  | **9. Carcinomas** | **60/48** | **1.3 (1.0, 1.6)** | **0.3 (0.0, 0.8)** |
|  | 9.6 Carcinoma of breast | 25/27 | 0.9 (0.6, 1.4) | -0.1 (-0.5, 0.5) |
|  | 9.6.1 Breast, infiltrating duct | 17/20 | 0.8 (0.5, 1.3) | -0.2 (-0.7, 0.4) |
|  | 9.7 Carcinoma of genital sites excluding ovary and testis | 19/12 | 1.6 (1.0, 2.5) | 0.9 (0.0, 2.2) |
|  | 9.7.1 Carcinoma of uterine cervix | 12/10 | 1.1 (0.6, 2.0) | 0.2 (-0.6, 1.4) |

**Supplementary Table S15B.** Standardised incidence ratios (SIRs) and absolute excess risks (AERs) of second primary skin carcinomas after each distinct first primary cancer type among six-month adolescent and young adult (AYA, aged 15-39 years) cancer survivors compared to the general population in the Netherlands. Second primary cancer types are grouped according to the AYA-specific classification scheme developed by Barr and colleagues (2020). First and second cancer combinations with less than n=10 observed second cancers were excluded from the analyses. This Table presents outcomes from the sensitivity analysis.

|  | **Second primary cancers** | **9.5 Carcinoma of skin (if collected)** | | |
| --- | --- | --- | --- | --- |
|  | **First primary cancers** | **Obs/exp** | **SIR (95%CI)** | **AER per 10,000 person-years (95%CI)** |
| **Males** | **2. Lymphomas** | **22/5** | **4.3 (2.7, 6.5)** | **2.4 (1.2, 4.0)** |
|  | 2.1 Non-Hodgkin lymphomas | 13/2 | 5.8 (3.1, 9.9) | 3.9 (1.7, 7.3) |
|  | **8. Melanoma, malignant** | **19/6** | **3.2 (1.9, 4.9)** | **2.0 (0.8, 3.7)** |
|  | 8.1 Superficial spreading/low cumulative sun damage melanoma | 13/4 | 3.7 (1.9, 6.3) | 2.4 (0.8, 4.7) |
|  | **9. Carcinomas** | **31/7** | **4.6 (3.1, 6.5)** | **3.8 (2.3, 5.9)** |
|  | 9.2 Other carcinoma of head and neck | 11/1 | 9.3 (4.7, 16.7) | 9.0 (4.0, 17.0) |
|  | 9.3 Carcinoma of gastrointestinal tract | 14/2 | 5.7 (3.1, 9.6) | 5.0 (2.3, 9.2) |
| **Females** | **8. Melanoma, malignant** | **38/13** | **2.9 (2.0, 4.0)** | **1.9 (1.1, 3.0)** |
|  | 8.1 Superficial spreading/low cumulative sun damage melanoma | 27/8 | 3.3 (2.2, 4.7) | 2.2 (1.1, 3.6) |
|  | **9. Carcinomas** | **59/45** | **1.3 (1.0, 1.7)** | **0.4 (0.0, 0.9)** |
|  | 9.6 Carcinoma of breast | 24/24 | 1.0 (0.6, 1.5) | 0.0 (-0.5, 0.6) |
|  | 9.6.1 Breast, infiltrating duct | 16/18 | 0.9 (0.5, 1.4) | -0.2 (-0.6, 0.5) |
|  | 9.7 Carcinoma of genital sites excluding ovary and testis | 19/12 | 1.6 (1.0, 2.5) | 0.9 (0.0, 2.2) |
|  | 9.7.1 Carcinoma of uterine cervix | 12/10 | 1.1 (0.6, 2.0) | 0.2 (-0.6, 1.4) |

**Supplementary Table S16A.** Standardised incidence ratios (SIRs) and absolute excess risks (AERs) of second primary breast carcinomas after each distinct first primary cancer type among six-month adolescent and young adult (AYA, aged 15-39 years) cancer survivors compared to the general population in the Netherlands. Second primary cancer types are grouped according to the AYA-specific classification scheme developed by Barr and colleagues (2020). First and second cancer combinations with less than n=10 observed second cancers were excluded from the analyses. This Table presents outcomes from the main analysis.

|  | **Second primary cancers** | **9.6 Carcinoma of breast** | | | **9.6.1 Breast, infiltrating duct** | | | **9.6.2 Breast, adenocarcinoma** | | | **9.6.3 Breast, lobular** | | |
| --- | --- | --- | --- | --- | --- | --- | --- | --- | --- | --- | --- | --- | --- |
|  | **First primary cancers** | **Obs/exp** | **SIR (95%CI)** | **AER per 10,000 person-years (95%CI)** | **Obs/exp** | **SIR (95%CI)** | **AER per 10,000 person-years (95%CI)** | **Obs/exp** | **SIR (95%CI)** | **AER per 10,000 person-years (95%CI)** | **Obs/exp** | **SIR (95%CI)** | **AER per 10,000 person-years (95%CI)** |
| **Females** | **1. Leukaemia’s and related disorders** | **15/17** | **0.9 (0.5, 1.4)** | **-1.4 (-5.6, 4.7)** | **13/15** | **0.9 (0.5, 1.5)** | **-1.1 (-4.9, 4.7)** | **NA** | **NA** | **NA** | **NA** | **NA** | **NA** |
|  | **2. Lymphomas** | **131/55** | **2.4 (2.0, 2.8)** | **14.5 (10.4, 19.1)** | **112/47** | **2.4 (2.0, 2.9)** | **12.4 (8.6, 16.7)** | **13/2** | **6.9 (3.7, 11.8)** | **2.1 (1.0, 3.9)** | **NA** | **NA** | **NA** |
|  | 2.1 Non-Hodgkin lymphomas | 35/23 | 1.5 (1.1, 2.1) | 7.1 (1.0, 15.0) | 32/19 | 1.7 (1.1, 2.4) | 7.4 (1.6, 15.0) | **NA** | **NA** | **NA** | **NA** | **NA** | **NA** |
|  | 2.2 Hodgkin lymphoma | 92/29 | 3.2 (2.6, 3.9) | 19.1 (13.7, 25.4) | 76/25 | 3.1 (2.4, 3.8) | 15.5 (10.6, 21.3) | 12/1 | 12.4 (6.4, 21.6) | 3.3 (1.6, 6.0) | **NA** | **NA** | **NA** |
|  | 2.2.2 Hodgkin classic, other | 91/28 | 3.2 (2.6, 4.0) | 19.4 (13.9, 25.8) | 75/24 | 3.1 (2.4, 3.9) | 15.7 (10.8, 21.6) | 12/1 | 12.6 (6.5, 22.1) | 3.4 (1.6, 6.2) | **NA** | **NA** | **NA** |
|  | **3. CNS and other intracranial and intraspinal neoplasms** | **13/13** | **1.0 (0.5, 1.7)** | **0.1 (-4.5, 6.9)** | **11/11** | **1.0 (0.5, 1.8)** | **0.0 (-4.1, 6.5)** | **NA** | **NA** | **NA** | **NA** | **NA** | **NA** |
|  | 3.1 Astroglial and related neoplasms | 12/11 | 1.1 (0.6, 1.9) | 0.6 (-4.4, 8.4) | 10/10 | 1.0 (0.5, 1.9) | 0.4 (-4.1, 7.6) | **NA** | **NA** | **NA** | **NA** | **NA** | **NA** |
|  | **4. Sarcomas** | **41/29** | **1.4 (1.0, 1.9)** | **5.0 (0.2, 11)** | **33/24** | **1.3 (0.9, 1.9)** | **3.5 (-0.7, 9.0)** | **NA** | **NA** | **NA** | **NA** | **NA** | **NA** |
|  | **7. Gonadal and related tumours** | **37/42** | **0.9 (0.6, 1.2)** | **-1.8 (-5.6, 3.1)** | **29/35** | **0.8 (0.5, 1.2)** | **-2.2 (-5.6, 2.1)** | **NA** | **NA** | **NA** | **NA** | **NA** | **NA** |
|  | 7.2 Ovary | 32/39 | 0.8 (0.6, 1.2) | -2.5 (-6.4, 2.5) | 24/32 | 0.7 (0.5, 1.1) | -3.2 (-6.5, 1.2) | **NA** | **NA** | **NA** | **NA** | **NA** | **NA** |
|  | 7.2.2 Non-germ cell | 27/35 | 0.8 (0.5, 1.1) | -3.6 (-7.8, 2.0) | 19/29 | 0.6 (0.4, 1.0) | -4.7 (-8.1, 0.2) | **NA** | **NA** | **NA** | **NA** | **NA** | **NA** |
|  | 7.2.2.1 Carcinoma | 25/33 | 0.7 (0.5, 1.1) | -4.0 (-8.2, 1.7) | 17/28 | 0.6 (0.4, 1.0) | -5.2 (-8.6, -0.4) | **NA** | **NA** | **NA** | **NA** | **NA** | **NA** |
|  | 7.2.2.1.1 Adenocarcinoma | 25/33 | 0.8 (0.5, 1.1) | -3.8 (-8.1, 2.0) | 17/28 | 0.6 (0.4, 1.0) | -5.1 (-8.6, -0.2) | **NA** | **NA** | **NA** | **NA** | **NA** | **NA** |
|  | 7.2.2.1.1.2 Cystadenocarcinoma | 22/26 | 0.8 (0.5, 1.3) | -2.5 (-7.5, 4.3) | 14/22 | 0.6 (0.3, 1.1) | -4.8 (-8.6, 0.9) | **NA** | **NA** | **NA** | **NA** | **NA** | **NA** |
|  | **8. Melanoma, malignant** | **218/186** | **1.2 (1.0, 1.3)** | **2.4 (0.3, 4.7)** | **179/158** | **1.1 (1.0, 1.3)** | **1.6 (-0.3, 3.7)** | **13/6** | **2.1 (1.1, 3.6)** | **0.5 (0.1, 1.2)** | **18/17** | **1.0 (0.6, 1.6)** | **0.0 (-0.5, 0.8)** |
|  | 8.1 Superficial spreading/low cumulative sun damage melanoma | 148/120 | 1.2 (1.0, 1.5) | 3.2 (0.6, 6.1) | 121/102 | 1.2 (1.0, 1.4) | 2.2 (-0.2, 4.8) | 12/4 | 3.1 (1.6, 5.4) | 0.9 (0.3, 1.9) | 12/11 | 1.1 (0.6, 1.9) | 0.1 (-0.6, 1.1) |
|  | 8.2 Nodular melanoma | 16/15 | 1.1 (0.6, 1.7) | 0.9 (-5.1, 9.7) | 13/13 | 1.0 (0.5, 1.8) | 0.3 (-5.0, 8.4) | **NA** | **NA** | **NA** | **NA** | **NA** | **NA** |
|  | 8.3 Other malignant | 54/51 | 1.1 (0.8, 1.4) | 0.8 (-3.1, 5.6) | 45/43 | 1.0 (0.8, 1.4) | 0.5 (-3.0, 4.9) | **NA** | **NA** | **NA** | **NA** | **NA** | **NA** |
|  | **9. Carcinomas** | **297/593** | **0.5 (0.4, 0.6)** | **-8.1 (-9.0, -7.2)** | **252/498** | **0.5 (0.4, 0.6)** | **-6.8 (-7.6, -5.9)** | **14/21** | **0.7 (0.4, 1.1)** | **-0.2 (-0.4, 0.1)** | **22/59** | **0.4 (0.2, 0.6)** | **-1.0 (-1.2, -0.7)** |
|  | 9.1 Thyroid carcinoma | 40/42 | 0.9 (0.7, 1.3) | -0.8 (-4.3, 3.7) | 34/36 | 0.9 (0.7, 1.3) | -0.6 (-3.9, 3.6) | **NA** | **NA** | **NA** | **NA** | **NA** | **NA** |
|  | 9.1.3 Papillary | 18/21 | 0.8 (0.5, 1.3) | -2.0 (-6.3, 4.2) | 16/18 | 0.9 (0.5, 1.4) | -1.3 (-5.3, 4.7) | **NA** | **NA** | **NA** | **NA** | **NA** | **NA** |
|  | 9.1.5 Papillary with follicular variant | 11/11 | 1.0 (0.5, 1.7) | -0.5 (-7.5, 10.5) | **NA** | **NA** | **NA** | **NA** | **NA** | **NA** | **NA** | **NA** | **NA** |
|  | 9.2 Other carcinoma of head and neck | 11/12 | 0.9 (0.5, 1.6) | -1.3 (-7.7, 8.9) | 10/10 | 1.0 (0.5, 1.8) | -0.2 (-6.3, 9.6) | **NA** | **NA** | **NA** | **NA** | **NA** | **NA** |
|  | 9.3 Carcinoma of gastrointestinal tract | 46/36 | 1.3 (0.9, 1.7) | 4.0 (-0.9, 10.0) | 42/30 | 1.4 (1.0, 1.9) | 4.6 (0.0, 10.4) | **NA** | **NA** | **NA** | **NA** | **NA** | **NA** |
|  | 9.3.4 Carcinoma of colon | 29/21 | 1.4 (0.9, 2.0) | 5.5 (-0.8, 13.8) | 27/17 | 1.5 (1.0, 2.2) | 6.3 (0.2, 14.3) | **NA** | **NA** | **NA** | **NA** | **NA** | **NA** |
|  | 9.3.4.2 Colon excluding appendix | 22/13 | 1.6 (1.0, 2.5) | 10.4 (0.5, 24.2) | 21/11 | 1.9 (1.2, 2.8) | 11.8 (2.1, 25.3) | **NA** | **NA** | **NA** | **NA** | **NA** | **NA** |
|  | 9.3.4.2.2 Colon excluding appendix, adenocarcinoma | 22/13 | 1.7 (1.0, 2.5) | 11.1 (0.8, 25.2) | 21/11 | 1.9 (1.2, 2.9) | 12.4 (2.4, 26.3) | **NA** | **NA** | **NA** | **NA** | **NA** | **NA** |
|  | 9.4 Carcinoma of lung, bronchus, and trachea | 12/8 | 1.5 (0.7, 2.5) | 6.9 (-3.9, 23.6) | **NA** | **NA** | **NA** | **NA** | **NA** | **NA** | **NA** | **NA** | **NA** |
|  | 9.4.2 Non-small cell carcinoma | 12/8 | 1.5 (0.8, 2.6) | 7.5 (-3.6, 24.6) | **NA** | **NA** | **NA** | **NA** | **NA** | **NA** | **NA** | **NA** | **NA** |
|  | 9.5 Carcinoma of skin (if collected) | 15/10 | 1.5 (0.8, 2.4) | 7.9 (-2.8, 23.6) | 13/9 | 1.5 (0.8, 2.6) | 7.2 (-2.6, 22.1) | **NA** | **NA** | **NA** | **NA** | **NA** | **NA** |
|  | 9.6 Carcinoma of breast | 11/332 | 0.0 (0.0, 0.1) | -16.5 (-16.8, -16.0) | **NA** | **NA** | **NA** | **NA** | **NA** | **NA** | **NA** | **NA** | **NA** |
|  | 9.7 Carcinoma of genital sites excluding ovary and testis | 144/140 | 1.0 (0.9, 1.2) | 0.5 (-2.2, 3.6) | 121/118 | 1.0 (0.9, 1.2) | 0.4 (-2.1, 3.2) | **NA** | **NA** | **NA** | 11/14 | 0.8 (0.4, 1.4) | -0.4 (-1.0, 0.7) |
|  | 9.7.1 Carcinoma of uterine cervix | 132/127 | 1.0 (0.9, 1.2) | 0.7 (-2.1, 4.0) | 113/106 | 1.1 (0.9, 1.3) | 0.9 (-1.8, 3.9) | **NA** | **NA** | **NA** | **NA** | **NA** | **NA** |
|  | 9.7.1.1 Cervix, squamous | 89/90 | 1.0 (0.8, 1.2) | -0.1 (-3.4, 3.7) | 79/75 | 1.0 (0.8, 1.3) | 0.7 (-2.4, 4.3) | **NA** | **NA** | **NA** | **NA** | **NA** | **NA** |
|  | 9.7.1.3 Cervix, adenocarcinoma | 26/23 | 1.1 (0.7, 1.7) | 2.1 (-4.2, 10.5) | 21/19 | 1.1 (0.7, 1.7) | 1.1 (-4.5, 8.9) | **NA** | **NA** | **NA** | **NA** | **NA** | **NA** |
|  | 9.7.1.4 Cervix, other | 11/10 | 1.1 (0.6, 2.0) | 2.2 (-8.2, 18.7) | **NA** | **NA** | **NA** | **NA** | **NA** | **NA** | **NA** | **NA** | **NA** |
|  | 9.8 Carcinoma of urinary tract | 11/9 | 1.3 (0.6, 2.3) | 4.2 (-5.8, 20.1) | 10/7 | 1.4 (0.7, 2.5) | 4.9 (-4.6, 20.2) | **NA** | **NA** | **NA** | **NA** | **NA** | **NA** |

Abbreviation: CNS=Central Nervous System, NA=Not Applicable.

**Supplementary Table S16B.** Standardised incidence ratios (SIRs) and absolute excess risks (AERs) of second primary breast carcinomas after each distinct first primary cancer type among six-month adolescent and young adult (AYA, aged 15-39 years) cancer survivors compared to the general population in the Netherlands. Second primary cancer types are grouped according to the AYA-specific classification scheme developed by Barr and colleagues (2020). First and second cancer combinations with less than n=10 observed second cancers were excluded from the analyses. This Table presents outcomes from the sensitivity analysis.

|  | **Second primary cancers** | **9.6 Carcinoma of breast** | | | **9.6.1 Breast, infiltrating duct** | | | **9.6.2 Breast, adenocarcinoma** | | | **9.6.3 Breast, lobular** | | | **9.6.5 Breast, medullary** | | | **9.6.8 Breast, metaplastic** | | | **9.6.10 Breast, other** | | |
| --- | --- | --- | --- | --- | --- | --- | --- | --- | --- | --- | --- | --- | --- | --- | --- | --- | --- | --- | --- | --- | --- | --- |
|  | **First primary cancers** | **Obs/exp** | **SIR (95%CI)** | **AER per 10,000 person-years (95%CI)** | **Obs/exp** | **SIR (95%CI)** | **AER per 10,000 person-years (95%CI)** | **Obs/exp** | **SIR (95%CI)** | **AER per 10,000 person-years (95%CI)** | **Obs/exp** | **SIR (95%CI)** | **AER per 10,000 person-years (95%CI)** | **Obs/exp** | **SIR (95%CI)** | **AER per 10,000 person-years (95%CI)** | **Obs/exp** | **SIR (95%CI)** | **AER per 10,000 person-years (95%CI)** | **Obs/exp** | **SIR (95%CI)** | **AER per 10,000 person-years (95%CI)** |
| **Females** | **1. Leukaemia’s and related disorders** | **15/17** | **0.9 (0.5, 1.4)** | **-1.5 (-5.6, 4.7)** | **13/15** | **0.9 (0.5, 1.5)** | **-1.1 (-4.9, 4.7)** | **NA** | **NA** | **NA** | **NA** | **NA** | **NA** | **NA** | **NA** | **NA** | **NA** | **NA** | **NA** | **NA** | **NA** | **NA** |
|  | **2. Lymphomas** | **131/55** | **2.4 (2.0, 2.8)** | **14.5 (10.4, 19.1)** | **112/47** | **2.4 (2.0, 2.9)** | **12.4 (8.7, 16.8)** | **13/2** | **6.9 (3.7, 11.9)** | **2.1 (1.0, 3.9)** | **NA** | **NA** | **NA** | **NA** | **NA** | **NA** | **NA** | **NA** | **NA** | **NA** | **NA** | **NA** |
|  | 2.1 Non-Hodgkin lymphomas | 35/23 | 1.6 (1.1, 2.2) | 7.2 (1.0, 15.0) | 32/19 | 1.7 (1.1, 2.4) | 7.4 (1.6, 15.0) | NA | NA | NA | NA | NA | NA | NA | NA | NA | NA | NA | NA | NA | NA | NA |
|  | 2.2 Hodgkin lymphoma | 92/29 | 3.2 (2.6, 3.9) | 19.1 (13.7, 25.4) | 76/25 | 3.1 (2.4, 3.8) | 15.5 (10.6, 21.3) | 12/1 | 12.4 (6.4, 21.6) | 3.3 (1.6, 6.0) | NA | NA | NA | NA | NA | NA | NA | NA | NA | NA | NA | NA |
|  | 2.2.2 Hodgkin classic, other | 91/28 | 3.2 (2.6, 4.0) | 19.4 (13.9, 25.8) | 75/24 | 3.1 (2.4, 3.9) | 15.7 (10.8, 21.6) | 12/1 | 12.6 (6.5, 22.1) | 3.4 (1.6, 6.2) | NA | NA | NA | NA | NA | NA | NA | NA | NA | NA | NA | NA |
|  | **3. CNS and other intracranial and intraspinal neoplasms** | **13/13** | **1.0 (0.5, 1.7)** | **0.1 (-4.4, 7.0)** | **11/11** | **1.0 (0.5, 1.8)** | **0.1 (-4.1, 6.6)** | **NA** | **NA** | **NA** | **NA** | **NA** | **NA** | **NA** | **NA** | **NA** | **NA** | **NA** | **NA** | **NA** | **NA** | **NA** |
|  | 3.1 Astroglial and related neoplasms | 12/11 | 1.1 (0.6, 1.9) | 0.7 (-4.3, 8.5) | 10/9 | 1.1 (0.5, 1.9) | 0.4 (-4.1, 7.7) | NA | NA | NA | NA | NA | NA | NA | NA | NA | NA | NA | NA | NA | NA | NA |
|  | **4. Sarcomas** | **41/29** | **1.4 (1.0, 1.9)** | **5.0 (0.3, 11.1)** | **33/24** | **1.4 (0.9, 1.9)** | **3.5 (-0.7, 9.0)** | **NA** | **NA** | **NA** | **NA** | **NA** | **NA** | **NA** | **NA** | **NA** | **NA** | **NA** | **NA** | **NA** | **NA** | **NA** |
|  | **7. Gonadal and related tumours** | **36/42** | **0.9 (0.6, 1.2)** | **-2.0 (-5.8, 2.8)** | **28/35** | **0.8 (0.5, 1.1)** | **-2.5 (-5.8, 1.8)** | **NA** | **NA** | **NA** | **NA** | **NA** | **NA** | **NA** | **NA** | **NA** | **NA** | **NA** | **NA** | **NA** | **NA** | **NA** |
|  | 7.2 Ovary | 31/38 | 0.8 (0.5, 1.1) | -2.8 (-6.7, 2.2) | 23/32 | 0.7 (0.5, 1.1) | -3.6 (-6.8, 0.9) | NA | NA | NA | NA | NA | NA | NA | NA | NA | NA | NA | NA | NA | NA | NA |
|  | 7.2.2 Non-germ cell | 26/35 | 0.8 (0.5, 1.1) | -3.9 (-8.1, 1.6) | 18/29 | 0.6 (0.4, 1.0) | -5.1 (-8.4, -0.3) | NA | NA | NA | NA | NA | NA | NA | NA | NA | NA | NA | NA | NA | NA | NA |
|  | 7.2.2.1 Carcinoma | 24/33 | 0.7 (0.5, 1.1) | -4.4 (-8.5, 1.2) | 16/28 | 0.6 (0.3, 0.9) | -5.7 (-8.9, -0.9) | NA | NA | NA | NA | NA | NA | NA | NA | NA | NA | NA | NA | NA | NA | NA |
|  | 7.2.2.1.1 Adenocarcinoma | 24/33 | 0.7 (0.5, 1.1) | -4.2 (-8.4, 1.5) | 16/27 | 0.6 (0.3, 1.0) | -5.5 (-8.9, -0.7) | NA | NA | NA | NA | NA | NA | NA | NA | NA | NA | NA | NA | NA | NA | NA |
|  | 7.2.2.1.1.2 Cystadenocarcinoma | 21/26 | 0.8 (0.5, 1.2) | -3.0 (-7.9, 3.7) | 13/22 | 0.6 (0.3, 1.0) | -5.3 (-9.0, 0.2) | NA | NA | NA | NA | NA | NA | NA | NA | NA | NA | NA | NA | NA | NA | NA |
|  | **8. Melanoma, malignant** | **207/179** | **1.2 (1.0, 1.3)** | **2.1 (0.0, 4.4)** | **170/152** | **1.1 (1.0, 1.3)** | **1.4 (-0.5, 3.5)** | **12/6** | **2.0 (1.0, 3.5)** | **0.5 (0.0, 1.1)** | **18/17** | **1.1 (0.6, 1.7)** | **0.1 (-0.5, 0.9)** | NA | NA | NA | NA | NA | NA | NA | NA | NA |
|  | 8.1 Superficial spreading/low cumulative sun damage melanoma | 140/116 | 1.2 (1.0, 1.4) | 2.8 (0.2, 5.8) | 114/98 | 1.2 (1.0, 1.4) | 1.8 (-0.5, 4.5) | 11/4 | 2.9 (1.4, 5.2) | 0.8 (0.2, 1.9) | 12/11 | 1.1 (0.6, 2.0) | 0.2 (-0.5, 1.2) | NA | NA | NA | NA | NA | NA | NA | NA | NA |
|  | 8.2 Nodular melanoma | 15/14 | 1.1 (0.6, 1.7) | 0.8 (-5.3, 9.7) | 13/12 | 1.1 (0.6, 1.9) | 0.9 (-4.6, 9.4) | NA | NA | NA | NA | NA | NA | NA | NA | NA | NA | NA | NA | NA | NA | NA |
|  | 8.3 Other malignant | 52/49 | 1.1 (0.8, 1.4) | 0.8 (-3.1, 5.6) | 43/42 | 1.0 (0.7, 1.4) | 0.4 (-3.1, 4.8) | NA | NA | NA | NA | NA | NA | NA | NA | NA | NA | NA | NA | NA | NA | NA |
|  | **9. Carcinomas** | **1,599/571** | **2.8 (2.7, 2.9)** | **29.1 (26.9, 31.3)** | **1,318/480** | **2.7 (2.6, 2.9)** | **23.7 (21.7, 25.8)** | **114/21** | **5.5 (4.6, 6.7)** | **2.6 (2.1, 3.3)** | **119/57** | **2.1 (1.7, 2.5)** | **1.8 (1.2, 2.4)** | **22/5** | **4.6 (2.9, 7.0)** | **0.5 (0.3, 0.8)** | **10/3** | **3.1 (1.5, 5.6)** | **0.2 (0.0, 0.4)** | **12/1** | **11.7 (6.0, 20.4)** | **0.3 (0.1, 0.6)** |
|  | 9.1 Thyroid carcinoma | 40/42 | 0.9 (0.7, 1.3) | -0.8 (-4.3, 3.7) | 34/36 | 0.9 (0.7, 1.3) | -0.6 (-3.9, 3.6) | NA | NA | NA | NA | NA | NA | NA | NA | NA | NA | NA | NA | NA | NA | NA |
|  | 9.1.3 Papillary | 18/21 | 0.8 (0.5, 1.3) | -2.0 (-6.3, 4.2) | 16/18 | 0.9 (0.5, 1.4) | -1.3 (-5.3, 4.7) | NA | NA | NA | NA | NA | NA | NA | NA | NA | NA | NA | NA | NA | NA | NA |
|  | 9.1.5 Papillary with follicular variant | 11/11 | 1.0 (0.5, 1.7) | -0.5 (-7.5, 10.5) | NA | NA | NA | NA | NA | NA | NA | NA | NA | NA | NA | NA | NA | NA | NA | NA | NA | NA |
|  | 9.2 Other carcinoma of head and neck | 11/12 | 0.9 (0.5, 1.7) | -1.1 (-7.5, 9.2) | 10/10 | 1.0 (0.5, 1.8) | 0.0 (-6.2, 9.8) | NA | NA | NA | NA | NA | NA | NA | NA | NA | NA | NA | NA | NA | NA | NA |
|  | 9.3 Carcinoma of gastrointestinal tract | 45/36 | 1.3 (0.9, 1.7) | 3.7 (-1.1, 9.7) | 41/30 | 1.4 (1.0, 1.8) | 4.3 (-0.3, 10.1) | NA | NA | NA | NA | NA | NA | NA | NA | NA | NA | NA | NA | NA | NA | NA |
|  | 9.3.4 Carcinoma of colon | 28/21 | 1.4 (0.9, 2.0) | 5.0 (-1.3, 13.2) | 26/17 | 1.5 (1.0, 2.2) | 5.7 (-0.2, 13.7) | NA | NA | NA | NA | NA | NA | NA | NA | NA | NA | NA | NA | NA | NA | NA |
|  | 9.3.4.2 Colon excluding appendix | 21/13 | 1.6 (1.0, 2.4) | 9.5 (-0.3, 23.2) | 20/11 | 1.8 (1.1, 2.8) | 10.9 (1.3, 24.3) | NA | NA | NA | NA | NA | NA | NA | NA | NA | NA | NA | NA | NA | NA | NA |
|  | 9.3.4.2.2 Colon excluding appendix, adenocarcinoma | 21/13 | 1.6 (1.0, 2.5) | 10.1 (0.0, 24.2) | 20/11 | 1.8 (1.1, 2.8) | 11.5 (1.6, 25.3) | NA | NA | NA | NA | NA | NA | NA | NA | NA | NA | NA | NA | NA | NA | NA |
|  | 9.4 Carcinoma of lung, bronchus, and trachea | 12/8 | 1.5 (0.8, 2.6) | 7.3 (-3.6, 24.2) | 13/9 | 1.5 (0.8, 2.6) | 7.2 (-2.6, 22.1) | NA | NA | NA | NA | NA | NA | NA | NA | NA | NA | NA | NA | NA | NA | NA |
|  | 9.4.2 Non-small cell carcinoma | 12/8 | 1.5 (0.8, 2.7) | 7.9 (-3.3, 25.2) | NA | NA | NA | NA | NA | NA | NA | NA | NA | NA | NA | NA | NA | NA | NA | NA | NA | NA |
|  | 9.5 Carcinoma of skin (if collected) | 15/10 | 1.5 (0.8, 2.4) | 7.9 (-2.8, 23.6) | 13/9 | 1.5 (0.8, 2.6) | 7.2 (-2.6, 22.1) | NA | NA | NA | NA | NA | NA | NA | NA | NA | NA | NA | NA | NA | NA | NA |
|  | 9.6 Carcinoma of breast | 1,315/312 | 4.2 (4.0, 4.5) | 54.0 (50.2, 57.9) | 1,075/262 | 4.1 (3.9, 4.4) | 43.8 (40.4, 47.3) | 100/12 | 8.7 (7.1, 10.5) | 4.8 (3.8, 5.9) | 98/31 | 3.2 (2.6, 3.8) | 3.6 (2.6, 4.8) | 18/3 | 7.0 (4.1, 11.1) | 0.8 (0.4, 1.4) | NA | NA | NA | 12/1 | 18.3 (9.5, 32.0) | 0.6 (0.3, 1.1) |
|  | 9.6.1 Breast, infiltrating duct | 992/243 | 4.1 (3.8, 4.3) | 50.8 (46.7, 55.2) | 838/204 | 4.1 (3.8, 4.4) | 43.0 (39.2, 47.0) | 71/9 | 8.0 (6.2, 10.1) | 4.2 (3.2, 5.5) | 57/24 | 2.4 (1.8, 3.1) | 2.2 (1.3, 3.4) | NA | NA | NA | NA | NA | NA | NA | NA | NA |
|  | 9.6.2 Breast, adenocarcinoma | 121/30 | 4.0 (3.3, 4.8) | 54.5 (42.2, 68.7) | 85/25 | 3.4 (2.7, 4.2) | 35.9 (25.7, 48.0) | 21/1 | 18.2 (11.3, 27.9) | 11.9 (7.1, 18.5) | NA | NA | NA | NA | NA | NA | NA | NA | NA | NA | NA | NA |
|  | 9.6.3 Breast, lobular | 81/16 | 5.0 (3.9, 6.2) | 72.4 (53.7, 94.4) | 49/14 | 3.6 (2.7, 4.8) | 39.6 (25.3, 57.2) | NA | NA | NA | 29/2 | 17.2 (11.5, 24.7) | 30.6 (19.8, 44.7) | NA | NA | NA | NA | NA | NA | NA | NA | NA |
|  | 9.6.5 Breast, medullary | 62/10 | 6.2 (4.8, 8.0) | 93.1 (67.3, 124.4) | 55/8 | 6.6 (5.0, 8.6) | 83.5 (59.2, 113.2) | NA | NA | NA | NA | NA | NA | 11/2 | 5.4 (2.7, 9.7) | 0.6 (0.2, 1.2) | NA | NA | NA | NA | NA | NA |
|  | 9.6.10 Breast, other | 46/8 | 5.4 (4.0, 7.2) | 80.2 (53.9, 113.1) | 36/7 | 5.1 (3.6, 7.1) | 61.9 (38.9, 91.5) | NA | NA | NA | NA | NA | NA | NA | NA | NA | NA | NA | NA | NA | NA | NA |
|  | 9.7 Carcinoma of genital sites excluding ovary and testis | 144/140 | 1.0 (0.9, 1.2) | 0.5 (-2.2, 3.6) | 121/118 | 1.0 (0.9, 1.2) | 0.4 (-2.1, 3.3) | NA | NA | NA | 11/14 | 0.8 (0.4, 1.4) | -0.4 (-1.0, 0.7) | NA | NA | NA | NA | NA | NA | NA | NA | NA |
|  | 9.7.1 Carcinoma of uterine cervix | 132/127 | 1.0 (0.9, 1.2) | 0.7 (-2.1, 4.0) | 113/106 | 1.1 (0.9, 1.3) | 0.9 (-1.8, 3.9) | NA | NA | NA | NA | NA | NA | NA | NA | NA | NA | NA | NA | NA | NA | NA |
|  | 9.7.1.1 Cervix, squamous | 89/90 | 1.0 (0.8, 1.2) | -0.1 (-3.4, 3.7) | 79/75 | 1.0 (0.8, 1.3) | 0.7 (-2.4, 4.3) | NA | NA | NA | NA | NA | NA | NA | NA | NA | NA | NA | NA | NA | NA | NA |
|  | 9.7.1.3 Cervix, adenocarcinoma | 26/23 | 1.1 (0.7, 1.7) | 2.1 (-4.2, 10.6) | 21/19 | 1.1 (0.7, 1.7) | 1.1 (-4.5, 8.9) | NA | NA | NA | NA | NA | NA | NA | NA | NA | NA | NA | NA | NA | NA | NA |
|  | 9.7.1.4 Cervix, other | 11/10 | 1.1 (0.6, 2.0) | 2.2 (-8.2, 18.7) | NA | NA | NA | NA | NA | NA | NA | NA | NA | NA | NA | NA | NA | NA | NA | NA | NA | NA |
|  | 9.8 Carcinoma of urinary tract | 10/8 | 1.2 (0.6, 2.2) | 3.1 (-6.7, 18.9) | NA | NA | NA | NA | NA | NA | NA | NA | NA | NA | NA | NA | NA | NA | NA | NA | NA | NA |

Abbreviation: CNS=Central Nervous System, NA=Not Applicable.

**Supplementary Table S17A.** Standardised incidence ratios (SIRs) and absolute excess risks (AERs) of second primary carcinomas of the genital sites (excluding ovary and testis) after each distinct first primary cancer type among six-month adolescent and young adult (AYA, aged 15-39 years) cancer survivors compared to the general population in the Netherlands. Second primary cancer types are grouped according to the AYA-specific classification scheme developed by Barr and colleagues (2020). First and second cancer combinations with less than n=10 observed second cancers were excluded from the analyses. This Table presents outcomes from the main analysis.

|  | **Second primary cancers** | **9.7 Carcinoma of genital sites excluding ovary and testis** | | | **9.7.1 Carcinoma of uterine cervix** | | | **9.7.1.1 Cervix, squamous** | | | **9.7.1.3 Cervix, adenocarcinoma** | | | **9.7.2 Corpus uteri** | | | **9.7.2.1 Corpus uteri, adenocarcinoma** | | | **9.7.2.1.1 Corpus uteri, endometrioid** | | | **9.7.2.1.2 Corpus uteri, other adenocarcinoma** | | | **9.7.3 Carcinoma of vulva and vagina** | | | **9.7.5 Carcinoma of prostate** | | |
| --- | --- | --- | --- | --- | --- | --- | --- | --- | --- | --- | --- | --- | --- | --- | --- | --- | --- | --- | --- | --- | --- | --- | --- | --- | --- | --- | --- | --- | --- | --- | --- |
|  | **First primary cancers** | **Obs/exp** | **SIR (95%CI)** | **AER per 10,000 person-years (95%CI)** | **Obs/exp** | **SIR (95%CI)** | **AER per 10,000 person-years (95%CI)** | **Obs/exp** | **SIR (95%CI)** | **AER per 10,000 person-years (95%CI)** | **Obs/exp** | **SIR (95%CI)** | **AER per 10,000 person-years (95%CI)** | **Obs/exp** | **SIR (95%CI)** | **AER per 10,000 person-years (95%CI)** | **Obs/exp** | **SIR (95%CI)** | **AER per 10,000 person-years (95%CI)** | **Obs/exp** | **SIR (95%CI)** | **AER per 10,000 person-years (95%CI)** | **Obs/exp** | **SIR (95%CI)** | **AER per 10,000 person-years (95%CI)** | **Obs/exp** | **SIR (95%CI)** | **AER per 10,000 person-years (95%CI)** | **Obs/exp** | **SIR (95%CI)** | **AER per 10,000 person-years (95%CI)** |
| **Males** | **2. Lymphomas** | **15/7** | **2.1  (1.2, 3.5)** | **1.1  (0.2, 2.5)** | **NA** | **NA** | **NA** | **NA** | **NA** | **NA** | **NA** | **NA** | **NA** | **NA** | **NA** | **NA** | **NA** | **NA** | **NA** | **NA** | **NA** | **NA** | **NA** | **NA** | **NA** | **NA** | **NA** | **NA** | **14/7** | **2.1  (1.2, 3.5)** | **1.0  (0.1, 2.4)** |
|  | 2.1 Non-Hodgkin lymphomas | 11/3 | 3.5  (1.7, 6.2) | 2.8  (0.8, 6.0) | **NA** | **NA** | **NA** | **NA** | **NA** | **NA** | **NA** | **NA** | **NA** | **NA** | **NA** | **NA** | **NA** | **NA** | **NA** | **NA** | **NA** | **NA** | **NA** | **NA** | **NA** | **NA** | **NA** | **NA** | 10/3 | 3.3  (1.6, 6.1) | 2.5  (0.7, 5.6) |
|  | **7. Gonadal and related tumours** | **19/13** | **1.4  (0.9, 2.3)** | **0.4  (-0.1, 1.2)** | **NA** | **NA** | **NA** | **NA** | **NA** | **NA** | **NA** | **NA** | **NA** | **NA** | **NA** | **NA** | **NA** | **NA** | **NA** | **NA** | **NA** | **NA** | **NA** | **NA** | **NA** | **NA** | **NA** | **NA** | **17/12** | **1.4  (0.8, 2.2)** | **0.3  (-0.2, 1.1)** |
|  | 7.1 Testis | 19/13 | 1.5  (0.9, 2.3) | 0.4  (-0.1, 1.2) | **NA** | **NA** | **NA** | **NA** | **NA** | **NA** | **NA** | **NA** | **NA** | **NA** | **NA** | **NA** | **NA** | **NA** | **NA** | **NA** | **NA** | **NA** | **NA** | **NA** | **NA** | **NA** | **NA** | **NA** | 17/12 | 1.4  (0.8, 2.2) | 0.4  (-0.2, 1.1) |
|  | 7.1.1 Germ cell and trophoblastic | 19/13 | 1.5  (0.9, 2.3) | 0.4  (-0.1, 1.2) | **NA** | **NA** | **NA** | **NA** | **NA** | **NA** | **NA** | **NA** | **NA** | **NA** | **NA** | **NA** | **NA** | **NA** | **NA** | **NA** | **NA** | **NA** | **NA** | **NA** | **NA** | **NA** | **NA** | **NA** | 17/12 | 1.4  (0.8, 2.3) | 0.4  (-0.2, 1.1) |
|  | 7.1.1.1 Seminoma | 11/8 | 1.4  (0.7, 2.4) | 0.5  (-0.4, 1.8) | **NA** | **NA** | **NA** | **NA** | **NA** | **NA** | **NA** | **NA** | **NA** | **NA** | **NA** | **NA** | **NA** | **NA** | **NA** | **NA** | **NA** | **NA** | **NA** | **NA** | **NA** | **NA** | **NA** | **NA** | 11/8 | 1.4  (0.7, 2.6) | 0.5  (-0.3, 1.9) |
|  | **8. Melanoma, malignant** | **10/9** | **1.1  (0.5, 2.1)** | **0.2  (-0.6, 1.4)** | **NA** | **NA** | **NA** | **NA** | **NA** | **NA** | **NA** | **NA** | **NA** | **NA** | **NA** | **NA** | **NA** | **NA** | **NA** | **NA** | **NA** | **NA** | **NA** | **NA** | **NA** | **NA** | **NA** | **NA** | **10/8** | **1.2  (0.6, 2.2)** | **0.2  (-0.5, 1.5)** |
|  | **9. Carcinomas** | **15/11** | **1.3  (0.7, 2.2)** | **0.6  (-0.4, 2.1)** | **NA** | **NA** | **NA** | **NA** | **NA** | **NA** | **NA** | **NA** | **NA** | **NA** | **NA** | **NA** | **NA** | **NA** | **NA** | **NA** | **NA** | **NA** | **NA** | **NA** | **NA** | **NA** | **NA** | **NA** | **13/11** | **1.2  (0.6, 2.1)** | **0.4  (-0.6, 1.8)** |
| **Females** | **2. Lymphomas** | **14/11** | **1.3  (0.7, 2.2)** | **0.6  (-0.6, 2.4)** | **NA** | **NA** | **NA** | **NA** | **NA** | **NA** | **NA** | **NA** | **NA** | **NA** | **NA** | **NA** | **NA** | **NA** | **NA** | **NA** | **NA** | **NA** | **NA** | **NA** | **NA** | **NA** | **NA** | **NA** | **NA** | **NA** | **NA** |
|  | 2.2 Hodgkin lymphoma | 10/6 | 1.6  (0.8, 3.0) | 1.2  (-0.4, 3.7) | **NA** | **NA** | **NA** | **NA** | **NA** | **NA** | **NA** | **NA** | **NA** | **NA** | **NA** | **NA** | **NA** | **NA** | **NA** | **NA** | **NA** | **NA** | **NA** | **NA** | **NA** | **NA** | **NA** | **NA** | **NA** | **NA** | **NA** |
|  | 2.2.2 Hodgkin classic, other | 10/6 | 1.7  (0.8, 3.0) | 1.2  (-0.4, 3.8) | **NA** | **NA** | **NA** | **NA** | **NA** | **NA** | **NA** | **NA** | **NA** | **NA** | **NA** | **NA** | **NA** | **NA** | **NA** | **NA** | **NA** | **NA** | **NA** | **NA** | **NA** | **NA** | **NA** | **NA** | **NA** | **NA** | **NA** |
|  | **8. Melanoma, malignant** | **29/33** | **0.9  (0.6, 1.3)** | **-0.3  (-1.0, 0.6)** | **20/22** | **0.9  (0.5, 1.4)** | **-0.2  (-0.8, 0.6)** | **12/16** | **0.7  (0.4, 1.3)** | **-0.3  (-0.8, 0.3)** | **NA** | **NA** | **NA** | **NA** | **NA** | **NA** | **NA** | **NA** | **NA** | **NA** | **NA** | **NA** | **NA** | **NA** | **NA** | **NA** | **NA** | **NA** | **NA** | **NA** | **NA** |
|  | 8.1 Superficial spreading/low cumulative sun damage melanoma | 16/22 | 0.7  (0.4, 1.2) | -0.6  (-1.4, 0.5) | 13/15 | 0.9  (0.5, 1.5) | -0.2  (-0.9, 0.8) | **NA** | **NA** | **NA** | **NA** | **NA** | **NA** | **NA** | **NA** | **NA** | **NA** | **NA** | **NA** | **NA** | **NA** | **NA** | **NA** | **NA** | **NA** | **NA** | **NA** | **NA** | **NA** | **NA** | **NA** |
|  | 8.3 Other malignant | 10/9 | 1.1  (0.5, 2.1) | 0.3  (-1.2, 2.7) | **NA** | **NA** | **NA** | **NA** | **NA** | **NA** | **NA** | **NA** | **NA** | **NA** | **NA** | **NA** | **NA** | **NA** | **NA** | **NA** | **NA** | **NA** | **NA** | **NA** | **NA** | **NA** | **NA** | **NA** | **NA** | **NA** | **NA** |
|  | **9. Carcinomas** | **160/98** | **1.6 (1.4, 1.9)** | **1.7  (1.1, 2.4)** | **48/61** | **0.8  (0.6, 1.0)** | **-0.3  (-0.7, 0.1)** | **34/44** | **0.8  (0.5, 1.1)** | **-0.3  (-0.6, 0.1)** | **11/13** | **0.8  (0.4, 1.5)** | **-0.1  (-0.2, 0.2)** | **76/27** | **2.8  (2.2, 3.5)** | **1.4  (0.9, 1.9)** | **73/25** | **2.9  (2.3, 3.6)** | **1.3  (0.9, 1.8)** | **61/22** | **2.8  (2.1, 3.6)** | **1.1  (0.7, 1.6)** | **12/4** | **3.4  (1.8, 6.0)** | **0.2  (0.1, 0.5)** | **27/9** | **2.9  (1.9, 4.2)** | **0.5  (0.2, 0.8)** | **NA** | **NA** | **NA** |
|  | 9.1 Thyroid carcinoma | 15/8 | 1.9  (1.1, 3.2) | 2.3  (0.2, 5.3) | **NA** | **NA** | **NA** | **NA** | **NA** | **NA** | **NA** | **NA** | **NA** | **NA** | **NA** | **NA** | **NA** | **NA** | **NA** | **NA** | **NA** | **NA** | **NA** | **NA** | **NA** | **NA** | **NA** | **NA** | **NA** | **NA** | **NA** |
|  | 9.3 Carcinoma of gastrointestinal tract | 24/6 | 3.8  (2.5, 5.7) | 7.0  (3.6, 11.6) | **NA** | **NA** | **NA** | **NA** | **NA** | **NA** | **NA** | **NA** | **NA** | 21/2 | 13.6  (8.4, 20.7) | 7.6  (4.5, 12.0) | 21/1 | 14.4  (8.9, 22.0) | 7.7 (4.5, 12.0) | 15/1 | 11.9  (6.7, 19.6) | 5.4 (2.8, 9.2) | **NA** | **NA** | **NA** | **NA** | **NA** | **NA** | **NA** | **NA** | **NA** |
|  | 9.3.4 Carcinoma of colon | 15/4 | 4.1  (2.3, 6.8) | 7.4  (3.1, 13.8) | **NA** | **NA** | **NA** | **NA** | **NA** | **NA** | **NA** | **NA** | **NA** | 13/1 | 14.6  (7.8, 25.0) | 7.9  (4.0, 14.0) | 13/1 | 15.5  (8.3, 26.5) | 8 (4.0, 14) | **NA** | **NA** | **NA** | **NA** | **NA** | **NA** | **NA** | **NA** | **NA** | **NA** | **NA** | **NA** |
|  | 9.3.4.2 Colon excluding appendix | 13/2 | 5.8  (3.1, 10) | 13.1  (5.7, 24.3) | **NA** | **NA** | **NA** | **NA** | **NA** | **NA** | **NA** | **NA** | **NA** | 12/1 | 19.8  (10.2, 34.5) | 13.8  (6.8, 24.7) | 12/1 | 20.9  (10.8, 36.5) | 13.9 (6.8, 24.7) | **NA** | **NA** | **NA** | **NA** | **NA** | **NA** | **NA** | **NA** | **NA** | **NA** | **NA** | **NA** |
|  | 9.3.4.2.2 Colon excluding appendix, adenocarcinoma | 13/2 | 6.0  (3.2, 10.2) | 13.5  (5.9, 25.1) | **NA** | **NA** | **NA** | **NA** | **NA** | **NA** | **NA** | **NA** | **NA** | 12/1 | 20.0  (10.4, 35.0) | 14.2  (7.0, 25.4) | 12/1 | 21.2  (10.9, 37.0) | 14.3 (7.0, 25.5) | **NA** | **NA** | **NA** | **NA** | **NA** | **NA** | **NA** | **NA** | **NA** | **NA** | **NA** | **NA** |
|  | 9.6 Carcinoma of breast | 73/54 | 1.4  (1.1, 1.7) | 1.0  (0.2, 2.0) | 26/33 | 0.8  (0.5, 1.2) | -0.3  (-0.8, 0.3) | 18/24 | 0.8  (0.4, 1.2) | -0.3  (-0.7, 0.2) | **NA** | **NA** | **NA** | 35/15 | 2.3  (1.6, 3.2) | 1.0  (0.5, 1.7) | 34/14 | 2.4  (1.6, 3.3) | 1.0 (0.5, 1.7) | 32/12 | 2.6  (1.8, 3.7) | 1.0  (0.5, 1.7) | **NA** | **NA** | **NA** | **NA** | **NA** | **NA** | **NA** | **NA** | **NA** |
|  | 9.6.1 Breast, infiltrating duct | 57/42 | 1.4  (1.0, 1.8) | 1.0  (0.1, 2.1) | 20/26 | 0.8  (0.5, 1.2) | -0.4  (-0.9, 0.3) | 14/19 | 0.7  (0.4, 1.2) | -0.3  (-0.7, 0.3) | **NA** | **NA** | **NA** | 27/11 | 2.4  (1.5, 3.4) | 1.0  (0.4, 1.8) | 26/11 | 2.4  (1.6, 3.5) | 1 (0.4, 1.8) | 25/9 | 2.7  (1.7, 4.0) | 1.0  (0.4, 1.8) | **NA** | **NA** | **NA** | **NA** | **NA** | **NA** | **NA** | **NA** | **NA** |
|  | 9.6.2 Breast, adenocarcinoma | 10/5 | 2.0  (1.0, 3.7) | 2.9  (-0.1, 7.7) | **NA** | **NA** | **NA** | **NA** | **NA** | **NA** | **NA** | **NA** | **NA** | **NA** | **NA** | **NA** | **NA** | **NA** | **NA** | **NA** | **NA** | **NA** | **NA** | **NA** | **NA** | **NA** | **NA** | **NA** | **NA** | **NA** | **NA** |
|  | 9.7 Carcinoma of genital sites excluding ovary and testis | 28/23 | 1.2  (0.8, 1.7) | 0.6  (-0.6, 2.1) | **NA** | **NA** | **NA** | **NA** | **NA** | **NA** | **NA** | **NA** | **NA** | **NA** | **NA** | **NA** | **NA** | **NA** | **NA** | **NA** | **NA** | **NA** | **NA** | **NA** | **NA** | 16/2 | 7.2  (4.1, 11.7) | 1.7  (0.8, 2.9) | **NA** | **NA** | **NA** |
|  | 9.7.1 Carcinoma of uterine cervix | 22/21 | 1.0  (0.7, 1.6) | 0.1  (-1.0, 1.6) | **NA** | **NA** | **NA** | **NA** | **NA** | **NA** | **NA** | **NA** | **NA** | **NA** | **NA** | **NA** | **NA** | **NA** | **NA** | **NA** | **NA** | **NA** | **NA** | **NA** | **NA** | 13/2 | 6.5  (3.5, 11.1) | 1.5  (0.7, 2.7) | **NA** | **NA** | **NA** |
|  | 9.7.1.1 Cervix, squamous | 12/15 | 0.8  (0.4, 1.4) | -0.5  (-1.6, 1.1) | **NA** | **NA** | **NA** | **NA** | **NA** | **NA** | **NA** | **NA** | **NA** | **NA** | **NA** | **NA** | **NA** | **NA** | **NA** | **NA** | **NA** | **NA** | **NA** | **NA** | **NA** | **NA** | **NA** | **NA** | **NA** | **NA** | **NA** |

Abbreviation: NA=Not Applicable.

**Supplementary Table S17B.** Standardised incidence ratios (SIRs) and absolute excess risks (AERs) of primary carcinomas of the genital sites (excluding ovary and testis) after each distinct first primary cancer type among six-month adolescent and young adult (AYA, aged 15-39 years) cancer survivors compared to the general population in the Netherlands. Second primary cancer types are grouped according to the AYA-specific classification scheme developed by Barr and colleagues (2020). First and second cancer combinations with less than n=10 observed second cancers were excluded from the analyses. This Table presents outcomes from the sensitivity analysis.

|  | **Second primary cancers** | **9.7 Carcinoma of genital sites excluding ovary and testis** | | | **9.7.1 Carcinoma of uterine cervix** | | | **9.7.1.1 Cervix, squamous** | | | **9.7.1.3 Cervix, adenocarcinoma** | | | **9.7.2 Corpus uteri** | | | **9.7.2.1 Corpus uteri, adenocarcinoma** | | | **9.7.2.1.1 Corpus uteri, endometrioid** | | | **9.7.2.1.2 Corpus uteri, other adenocarcinoma** | | | **9.7.3 Carcinoma of vulva and vagina** | | | **9.7.5 Carcinoma of prostate** | | |
| --- | --- | --- | --- | --- | --- | --- | --- | --- | --- | --- | --- | --- | --- | --- | --- | --- | --- | --- | --- | --- | --- | --- | --- | --- | --- | --- | --- | --- | --- | --- | --- |
|  | **First primary cancers** | **Obs/exp** | **SIR (95%CI)** | **AER per 10,000 person-years (95%CI)** | **Obs/exp** | **SIR (95%CI)** | **AER per 10,000 person-years (95%CI)** | **Obs/exp** | **SIR (95%CI)** | **AER per 10,000 person-years (95%CI)** | **Obs/exp** | **SIR (95%CI)** | **AER per 10,000 person-years (95%CI)** | **Obs/exp** | **SIR (95%CI)** | **AER per 10,000 person-years (95%CI)** | **Obs/exp** | **SIR (95%CI)** | **AER per 10,000 person-years (95%CI)** | **Obs/exp** | **SIR (95%CI)** | **AER per 10,000 person-years (95%CI)** | **Obs/exp** | **SIR (95%CI)** | **AER per 10,000 person-years (95%CI)** | **Obs/exp** | **SIR (95%CI)** | **AER per 10,000 person-years (95%CI)** | **Obs/exp** | **SIR (95%CI)** | **AER per 10,000 person-years (95%CI)** |
| **Males** | **2. Lymphomas** | **15/7** | **2.1  (1.2, 3.5)** | **1.1  (0.2, 2.5)** | **NA** | **NA** | **NA** | **NA** | **NA** | **NA** | **NA** | **NA** | **NA** | **NA** | **NA** | **NA** | **NA** | **NA** | **NA** | **NA** | **NA** | **NA** | **NA** | **NA** | **NA** | **NA** | **NA** | **NA** | **14/7** | **2.1  (1.2, 3.6)** | **1.1  (0.1, 2.4)** |
|  | 2.1 Non-Hodgkin lymphomas | 11/3 | 3.5  (1.7, 6.2) | 2.9  (0.9, 6.0) | NA | NA | NA | NA | NA | NA | NA | NA | NA | NA | NA | NA | NA | NA | NA | NA | NA | NA | NA | NA | NA | NA | NA | NA | 10/3 | 3.4  (1.6, 6.2) | 2.6  (0.7, 5.6) |
|  | **7. Gonadal and related tumours** | **19/13** | **1.5  (0.9, 2.3)** | **0.4  (-0.1, 1.2)** | **NA** | **NA** | **NA** | **NA** | **NA** | **NA** | **NA** | **NA** | **NA** | **NA** | **NA** | **NA** | **NA** | **NA** | **NA** | **NA** | **NA** | **NA** | **NA** | **NA** | **NA** | **NA** | **NA** | **NA** | **17/12** | **1.4  (0.8, 2.3)** | **0.4  (-0.2, 1.1)** |
|  | 7.1 Testis | 19/13 | 1.5  (0.9, 2.3) | 0.5  (-0.1, 1.3) | NA | NA | NA | NA | NA | NA | NA | NA | NA | NA | NA | NA | NA | NA | NA | NA | NA | NA | NA | NA | NA | NA | NA | NA | 17/12 | 1.4  (0.8, 2.3) | 0.4  (-0.1, 1.1) |
|  | 7.1.1 Germ cell and trophoblastic | 19/13 | 1.5  (0.9, 2.3) | 0.5  (-0.1, 1.3) | NA | NA | NA | NA | NA | NA | NA | NA | NA | NA | NA | NA | NA | NA | NA | NA | NA | NA | NA | NA | NA | NA | NA | NA | 17/12 | 1.4  (0.8, 2.3) | 0.4  (-0.1, 1.1) |
|  | 7.1.1.1 Seminoma | 11/8 | 1.4  (0.7, 2.5) | 0.5  (-0.4, 1.9) | NA | NA | NA | NA | NA | NA | NA | NA | NA | NA | NA | NA | NA | NA | NA | NA | NA | NA | NA | NA | NA | NA | NA | NA | 11/7 | 1.5  (0.7, 2.6) | 0.6  (-0.3, 2) |
|  | **8. Melanoma, malignant** | **10/9** | **1.2  (0.6, 2.2)** | **0.2  (-0.6, 1.5)** | **NA** | **NA** | **NA** | **NA** | **NA** | **NA** | **NA** | **NA** | **NA** | **NA** | **NA** | **NA** | **NA** | **NA** | **NA** | **NA** | **NA** | **NA** | **NA** | **NA** | **NA** | **NA** | **NA** | **NA** | **10/8** | **1.2  (0.6, 2.3)** | **0.3 (-0.5, 1.6)** |
|  | **9. Carcinomas** | **14/11** | **1.3  (0.7, 2.1)** | **0.5  (-0.5, 2.0)** | **NA** | **NA** | **NA** | **NA** | **NA** | **NA** | **NA** | **NA** | **NA** | **NA** | **NA** | **NA** | **NA** | **NA** | **NA** | **NA** | **NA** | **NA** | **NA** | **NA** | **NA** | **NA** | **NA** | **NA** | **12/10** | **1.1  (0.6, 2.0)** | **0.2  (-0.7, 1.7)** |
| **Females** | **2. Lymphomas** | **14/11** | **1.3  (0.7, 2.2)** | **0.6  (-0.6, 2.4)** | **NA** | **NA** | **NA** | **NA** | **NA** | **NA** | **NA** | **NA** | **NA** | **NA** | **NA** | **NA** | **NA** | **NA** | **NA** | **NA** | **NA** | **NA** | **NA** | **NA** | **NA** | **NA** | **NA** | **NA** | **NA** | **NA** | **NA** |
|  | 2.2 Hodgkin lymphoma | 10/6 | 1.6  (0.8, 3.0) | 1.2  (-0.4, 3.7) | NA | NA | NA | NA | NA | NA | NA | NA | NA | NA | NA | NA | NA | NA | NA | NA | NA | NA | NA | NA | NA | NA | NA | NA | NA | NA | NA |
|  | 2.2.2 Hodgkin classic, other | 10/6 | 1.7  (0.8, 3.0) | 1.2  (-0.4, 3.8) | NA | NA | NA | NA | NA | NA | NA | NA | NA | NA | NA | NA | NA | NA | NA | NA | NA | NA | NA | NA | NA | NA | NA | NA | NA | NA | NA |
|  | **8. Melanoma, malignant** | **28/32** | **0.9  (0.6, 1.3)** | **-0.3  (-1.0, 0.6)** | **19/22** | **0.9  (0.5, 1.4)** | **-0.2  (-0.8, 0.6)** | **12/16** | **0.8  (0.4, 1.3)** | **-0.3  (-0.7, 0.4)** | **NA** | **NA** | **NA** | **NA** | **NA** | **NA** | **NA** | **NA** | **NA** | **NA** | **NA** | **NA** | **NA** | **NA** | **NA** | **NA** | **NA** | **NA** | **NA** | **NA** | **NA** |
|  | 8.1 Superficial spreading/low cumulative sun damage melanoma | 15/21 | 0.7  (0.4, 1.2) | -0.7  (-1.5, 0.4) | 12/14 | 0.8  (0.4, 1.5) | -0.3  (-1.0, 0.8) | NA | NA | NA | NA | NA | NA | NA | NA | NA | NA | NA | NA | NA | NA | NA | NA | NA | NA | NA | NA | NA | NA | NA | NA |
|  | 8.3 Other malignant | 10/9 | 1.2  (0.6, 2.2) | 0.4  (-1.1, 2.9) | NA | NA | NA | NA | NA | NA | NA | NA | NA | NA | NA | NA | NA | NA | NA | NA | NA | NA | NA | NA | NA | NA | NA | NA | NA | NA | NA |
|  | **9. Carcinomas** | **161/95** | **1.7  (1.4, 2.0)** | **1.9  (1.2, 2.6)** | **50/59** | **0.8  (0.6, 1.1)** | **-0.3  (-0.6, 0.2)** | **35/43** | **0.8  (0.6, 1.1)** | **-0.2  (-0.5, 0.2)** | **12/13** | **0.9  (0.5, 1.6)** | **0.0  (-0.2, 0.2)** | **72/26** | **2.8  (2.2, 3.5)** | **1.3  (0.9, 1.8)** | **69/24** | **2.8  (2.2, 3.6)** | **1.3  (0.8, 1.8)** | **58/21** | **2.8  (2.1, 3.6)** | **1.1  (0.7, 1.5)** | **11/3** | **3.3  (1.6, 5.9)** | **0.2  (0.1, 0.5)** | **31/9** | **3.5  (2.3, 4.9)** | **0.6  (0.3, 1.0)** | **NA** | **NA** | **NA** |
|  | 9.1 Thyroid carcinoma | 15/8 | 1.9  (1.1, 3.2) | 2.3  (0.2, 5.3) | NA | NA | NA | NA | NA | NA | NA | NA | NA | NA | NA | NA | NA | NA | NA | NA | NA | NA | NA | NA | NA | NA | NA | NA | NA | NA | NA |
|  | 9.3 Carcinoma of gastrointestinal tract | 23/6 | 3.7  (2.3, 5.6) | 6.6  (3.3, 11.2) | NA | NA | NA | NA | NA | NA | NA | NA | NA | 20/2 | 13.0  (7.9, 20.1) | 7.3  (4.2, 11.6) | 20/1 | 13.8  (8.4, 21.3) | 7.3  (4.2, 11.6) | 15/1 | 12.0  (6.7, 19.7) | 5.4  (2.8, 9.3) | NA | NA | NA | NA | NA | NA | NA | NA | NA |
|  | 9.3.4.2 Colon excluding appendix | 12/2 | 5.5  (2.8, 9.5) | 12  (4.9, 23.1) | NA | NA | NA | NA | NA | NA | NA | NA | NA | 11/1 | 18.3  (9.1, 32.8) | 12.8  (6.0, 23.5) | 11/1 | 19.4  (9.7, 34.7) | 12.8  (6.1, 23.5) | NA | NA | NA | NA | NA | NA | NA | NA | NA | NA | NA | NA |
|  | 9.3.4.2.2 Colon excluding appendix, adenocarcinoma | 12/2 | 5.6  (2.9, 9.8) | 12.5  (5.1, 23.8) | NA | NA | NA | NA | NA | NA | NA | NA | NA | 11/1 | 18.6  (9.3, 33.2) | 13.2 (6.2, 24.2) | 11/1 | 19.6  (9.8, 35.1) | 13.2  (6.2, 24.2) | NA | NA | NA | NA | NA | NA | NA | NA | NA | NA | NA | NA |
|  | 9.3.4 Carcinoma of colon | 14/4 | 3.9  (2.1, 6.5) | 6.9  (2.7, 13.1) | NA | NA | NA | NA | NA | NA | NA | NA | NA | 12/1 | 13.6  (7.0, 23.8) | 7.3  (3.5, 13.3) | 12/1 | 14.4  (7.5, 25.2) | 7.4  (3.5, 13.3) | NA | NA | NA | NA | NA | NA | NA | NA | NA | NA | NA | NA |
|  | 9.6 Carcinoma of breast | 70/50 | 1.4  (1.1, 1.8) | 1.1  (0.2, 2.0) | 26/31 | 0.8  (0.5, 1.2) | -0.3  (-0.8, 0.4) | 18/23 | 0.8  (0.5, 1.3) | -0.3  (-0.6, 0.3) | NA | NA | NA | 32/14 | 2.3  (1.6, 3.2) | 1.0  (0.4, 1.7) | 31/13 | 2.4  (1.6, 3.3) | 1.0  (0.4, 1.7) | 29/11 | 2.6  (1.7, 3.7) | 1.0  (0.4, 1.6) | NA | NA | NA | NA | NA | NA | NA | NA | NA |
|  | 9.6.1 Breast, infiltrating duct | 56/40 | 1.4  (1.1, 1.8) | 1.1  (0.2, 2.2) | 20/25 | 0.8  (0.5, 1.2) | -0.3  (-0.9, 0.4) | 14/18 | 0.8  (0.4, 1.3) | -0.3  (-0.7, 0.4) | NA | NA | NA | 25/11 | 2.4  (1.5, 3.5) | 1.0  (0.4, 1.8) | 24/10 | 2.4  (1.5, 3.6) | 1.0  (0.4, 1.7) | 23/9 | 2.7  (1.7, 4.0) | 1.0  (0.4, 1.8) | NA | NA | NA | NA | NA | NA | NA | NA | NA |
|  | 9.7 Carcinoma of genital sites excluding ovary and testis | 33/23 | 1.4  (1.0, 2.0) | 1.2  (-0.1, 2.8) | NA | NA | NA | NA | NA | NA | NA | NA | NA | NA | NA | NA | NA | NA | NA | NA | NA | NA | NA | NA | NA | 19/2 | 8.6  (5.1, 13.4) | 2.0  (1.1, 3.3) | NA | NA | NA |
|  | 9.7.1 Carcinoma of uterine cervix | 24/21 | 1.1  (0.7, 1.7) | 0.4  (-0.7, 1.9) | NA | NA | NA | NA | NA | NA | NA | NA | NA | NA | NA | NA | NA | NA | NA | NA | NA | NA | NA | NA | NA | 13/2 | 6.5  (3.5, 11.1) | 1.5  (0.7, 2.7) | NA | NA | NA |
|  | 9.7.1.1 Cervix, squamous | 13/15 | 0.9  (0.5, 1.5) | -0.4  (-1.5, 1.4) | NA | NA | NA | NA | NA | NA | NA | NA | NA | NA | NA | NA | NA | NA | NA | NA | NA | NA | NA | NA | NA | NA | NA | NA | NA | NA | NA |

Abbreviation: NA=Not Applicable.

**Supplementary Table S18A.** Standardised incidence ratios (SIRs) and absolute excess risks (AERs) of second primary urinary tract carcinomas after each distinct first primary cancer type among six-month adolescent and young adult (AYA, aged 15-39 years) cancer survivors compared to the general population in the Netherlands. Second primary cancer types are grouped according to the AYA-specific classification scheme developed by Barr and colleagues (2020). First and second cancer combinations with less than n=10 observed second cancers were excluded from the analyses. This Table presents outcomes from the main analysis.

|  | **Second primary cancers** | **9.8 Carcinoma of urinary tract** | | | **9.8.1 Carcinoma of kidney** | | | **9.8.1.1 Kidney, adenocarcinoma** | | | **9.8.1.1.1 Kidney, renal cell** | | | **9.8.2 Carcinoma of bladder** | | | **9.8.2.1 Urinary bladder, transitional cell carcinoma** | | |
| --- | --- | --- | --- | --- | --- | --- | --- | --- | --- | --- | --- | --- | --- | --- | --- | --- | --- | --- | --- |
|  | **First primary cancers** | **Obs/exp** | **SIR (95%CI)** | **AER per 10,000 person-years (95%CI)** | **Obs/exp** | **SIR (95%CI)** | **AER per 10,000 person-years (95%CI)** | **Obs/exp** | **SIR (95%CI)** | **AER per 10,000 person-years (95%CI)** | **Obs/exp** | **SIR (95%CI)** | **AER per 10,000 person-years (95%CI)** | **Obs/exp** | **SIR (95%CI)** | **AER per 10,000 person-years (95%CI)** | **Obs/exp** | **SIR (95%CI)** | **AER per 10,000 person-years (95%CI)** |
| **Males** | **2. Lymphomas** | **15/7** | **2.1 (1.2, 3.5)** | **1.1 (0.2, 2.5)** | **12/5** | **2.6 (1.4, 4.6)** | **1.1 (0.2, 2.3)** | **12/5** | **2.6 (1.4, 4.6)** | **1.1 (0.2, 2.3)** | **NA** | **NA** | **NA** | **NA** | **NA** | **NA** | **NA** | **NA** | **NA** |
|  | **7. Gonadal and related tumours** | **32/14** | **2.3 (1.6, 3.2)** | **1.3 (0.6, 2.2)** | **15/9** | **1.6 (0.9, 2.7)** | **0.4 (-0.1, 1.1)** | **15/9** | **1.6 (0.9, 2.7)** | **0.4 (-0.1, 1.1)** | **13/8** | **1.7 (0.9, 2.9)** | **0.4 (0.0, 1.0)** | **13/4** | **3.1 (1.7, 5.4)** | **0.6 (0.2, 1.3)** | **11/4** | **2.9 (1.5, 5.2)** | **0.5 (0.1, 1.1)** |
|  | 7.1 Testis | 32/14 | 2.3 (1.6, 3.3) | 1.3 (0.6, 2.3) | 15/9 | 1.7 (0.9, 2.8) | 0.4 (0.0, 1.1) | 15/9 | 1.7 (0.9, 2.8) | 0.4 (0.0, 1.2) | 13/7 | 1.8 (0.9, 3.0) | 0.4 (0.0, 1.1) | 13/4 | 3.2 (1.7, 5.5) | 0.7 (0.2, 1.3) | 11/4 | 3.0 (1.5, 5.3) | 0.5 (0.1, 1.2) |
|  | 7.1.1 Germ cell and trophoblastic | 32/14 | 2.3 (1.6, 3.3) | 1.3 (0.6, 2.3) | 15/9 | 1.7 (0.9, 2.8) | 0.4 (0.0, 1.2) | 15/9 | 1.7 (0.9, 2.8) | 0.4 (0.0, 1.2) | 13/7 | 1.8 (0.9, 3.0) | 0.4 (0.0, 1.1) | 13/4 | 3.2 (1.7, 5.5) | 0.7 (0.2, 1.3) | 11/4 | 3.0 (1.5, 5.3) | 0.5 (0.1, 1.2) |
|  | 7.1.1.1 Seminoma | 18/8 | 2.3 (1.3, 3.6) | 1.6 (0.4, 3.2) | NA | NA | NA | NA | NA | NA | NA | NA | NA | NA | NA | NA | NA | NA | NA |
|  | **8. Melanoma, malignant** | **12/8** | **1.4 (0.7, 2.5)** | **0.5 (-0.3, 1.9)** | **NA** | **NA** | **NA** | **NA** | **NA** | **NA** | **NA** | **NA** | **NA** | **NA** | **NA** | **NA** | **NA** | **NA** | **NA** |
|  | **9. Carcinomas** | **19/9** | **2.1 (1.2, 3.2)** | **1.5 (0.3, 3.2)** | **10/6** | **1.7 (0.8, 3.2)** | **0.7 (-0.2, 2.0)** | **10/6** | **1.7 (0.8, 3.2)** | **0.7 (-0.2, 2.0)** | **NA** | **NA** | **NA** | **NA** | **NA** | **NA** | **NA** | **NA** | **NA** |
| **Females** | **9. Carcinomas** | **56/26** | **2.2 (1.6, 2.8)** | **0.8 (0.5, 1.3)** | **28/17** | **1.7 (1.1, 2.4)** | **0.3 (0.1, 0.7)** | **27/17** | **1.6 (1.1, 2.4)** | **0.3 (0.0, 0.6)** | **25/14** | **1.8 (1.1, 2.6)** | **0.3 (0.1, 0.6)** | **23/8** | **2.9 (1.8, 4.4)** | **0.4 (0.2, 0.7)** | **18/7** | **2.7 (1.6, 4.2)** | **0.3 (0.1, 0.6)** |
|  | 9.6 Carcinoma of breast | 20/14 | 1.4 (0.8, 2.1) | 0.3 (-0.1, 0.8) | 14/9 | 1.5 (0.8, 2.5) | 0.2 (-0.1, 0.7) | 14/9 | 1.5 (0.8, 2.6) | 0.2 (-0.1, 0.7) | 13/8 | 1.6 (0.9, 2.8) | 0.3 (-0.1, 0.7) | NA | NA | NA | NA | NA | NA |
|  | 9.6.1 Breast, infiltrating duct | 14/11 | 1.3 (0.7, 2.1) | 0.2 (-0.2, 0.8) | NA | NA | NA | NA | NA | NA | NA | NA | NA | NA | NA | NA | NA | NA | NA |
|  | 9.7 Carcinoma of genital sites excluding ovary and testis | 22/6 | 3.5 (2.2, 5.4) | 1.9 (0.9, 3.3) | NA | NA | NA | NA | NA | NA | NA | NA | NA | 16/2 | 8.3 (4.8, 13.5) | 1.7 (0.9, 2.9) | 12/2 | 7.3 (3.7, 12.7) | 1.2 (0.5, 2.3) |
|  | 9.7.1 Carcinoma of uterine cervix | 20/6 | 3.6 (2.2, 5.5) | 1.9 (0.9, 3.3) | NA | NA | NA | NA | NA | NA | NA | NA | NA | 14/2 | 8.1 (4.4, 13.6) | 1.6 (0.8, 2.9) | 10/1 | 6.7 (3.2, 12.4) | 1.1 (0.4, 2.2) |
|  | 9.7.1.1 Cervix, squamous | 15/4 | 3.8 (2.1, 6.3) | 2.1 (0.8, 3.9) | NA | NA | NA | NA | NA | NA | NA | NA | NA | 11/1 | 9.0 (4.5, 16.2) | 1.8 (0.8, 3.5) | NA | NA | NA |

Abbreviation: NA=Not Applicable.

**Supplementary Table S18B.** Standardised incidence ratios (SIRs) and absolute excess risks (AERs) of second primary urinary tract carcinomas after each distinct first primary cancer type among six-month adolescent and young adult (AYA, aged 15-39 years) cancer survivors compared to the general population in the Netherlands. Second primary cancer types are grouped according to the AYA-specific classification scheme developed by Barr and colleagues (2020). First and second cancer combinations with less than n=10 observed second cancers were excluded from the analyses. This Table presents outcomes from the sensitivity analysis.

|  | **Second primary cancers** | **9.8 Carcinoma of urinary tract** | | | **9.8.1 Carcinoma of kidney** | | | **9.8.1.1 Kidney, adenocarcinoma** | | | **9.8.1.1.1 Kidney, renal cell** | | | **9.8.2 Carcinoma of bladder** | | | **9.8.2.1 Urinary bladder, transitional cell carcinoma** | | |  |
| --- | --- | --- | --- | --- | --- | --- | --- | --- | --- | --- | --- | --- | --- | --- | --- | --- | --- | --- | --- | --- |
|  | | **First primary cancers** | **Obs/exp** | **SIR (95%CI)** | **AER per 10,000 person-years (95%CI)** | **Obs/exp** | **SIR (95%CI)** | **AER per 10,000 person-years (95%CI)** | **Obs/exp** | **SIR (95%CI)** | **AER per 10,000 person-years (95%CI)** | **Obs/exp** | **SIR (95%CI)** | **AER per 10,000 person-years (95%CI)** | **Obs/exp** | **SIR (95%CI)** | **AER per 10,000 person-years (95%CI)** | **Obs/exp** | **SIR (95%CI)** | **AER per 10,000 person-years (95%CI)** |
| **Males** | | **2. Lymphomas** | **15/7** | **2.1 (1.2, 3.5)** | **1.1 (0.2, 2.5)** | **12/5** | **2.6 (1.4, 4.6)** | **1.1 (0.2, 2.3)** | **12/5** | **2.7 (1.4, 4.6)** | **1.1 (0.2, 2.3)** | **NA** | **NA** | **NA** | **NA** | **NA** | **NA** | **NA** | **NA** | **NA** |
|  | | **7. Gonadal and related tumours** | **32/14** | **2.3 (1.6, 3.3)** | **1.3 (0.6, 2.3)** | **15/9** | **1.7 (0.9, 2.8)** | **0.4 (0.0, 1.1)** | **15/9** | **1.7 (0.9, 2.8)** | **0.4 (0.0, 1.1)** | **13/7** | **1.8 (0.9, 3.0)** | **0.4 (0.0, 1.1)** | **13/4** | **3.2 (1.7, 5.5)** | **0.6 (0.2, 1.3)** | **11/4** | **3.0 (1.5, 5.3)** | **0.5 (0.1, 1.2)** |
|  | | 7.1 Testis | 32/13 | 2.4 (1.6, 3.4) | 1.4 (0.6, 2.4) | 15/9 | 1.7 (1.0, 2.8) | 0.5 (0.0, 1.2) | 15/9 | 1.7 (1.0, 2.8) | 0.5 (0.0, 1.2) | 13/7 | 1.8 (1.0, 3.1) | 0.4 (0.0, 1.1) | 13/4 | 3.3 (1.7, 5.6) | 0.7 (0.2, 1.4) | 11/4 | 3.0 (1.5, 5.4) | 0.6 (0.1, 1.2) |
|  | | 7.1.1 Germ cell and trophoblastic | 32/13 | 2.4 (1.6, 3.4) | 1.4 (0.6, 2.4) | 15/9 | 1.7 (1.0, 2.8) | 0.5 (0.0, 1.2) | 15/9 | 1.7 (1.0, 2.8) | 0.5 (0.0, 1.2) | 13/7 | 1.8 (1.0, 3.1) | 0.4 (0.0, 1.1) | 13/4 | 3.3 (1.7, 5.6) | 0.7 (0.2, 1.4) | 11/4 | 3.0 (1.5, 5.4) | 0.6 (0.1, 1.2) |
|  | | 7.1.1.1 Seminoma | 18/8 | 2.3 (1.4, 3.7) | 1.7 (0.5, 3.4) | NA | NA | NA | NA | NA | NA | NA | NA | NA | NA | NA | NA | NA | NA | NA |
|  | | **8. Melanoma, malignant** | **11/8** | **1.4 (0.7, 2.4)** | **0.4 (-0.4, 1.8)** | **NA** | **NA** | **NA** | **NA** | **NA** | **NA** | **NA** | **NA** | **NA** | **NA** | **NA** | **NA** | **NA** | **NA** | **NA** |
|  | | **9. Carcinomas** | **44/9** | **4.9 (3.5, 6.5)** | **5.5 (3.6, 7.9)** | **31/6** | **5.5 (3.7, 7.8)** | **4.0 (2.4, 6.0)** | **31/6** | **5.5 (3.7, 7.8)** | **4.0 (2.4, 6.1)** | **27/5** | **5.7 (3.8, 8.3)** | **3.5 (2.1, 5.5)** | **NA** | **NA** | **NA** | **NA** | **NA** | **NA** |
|  | | 9.8 Carcinoma of urinary tract | 28/1 | 20.4 (13.6, 29.5) | 32.4 (20.9, 47.5) | 23/1 | 27.1 (17.1, 40.6) | 26.9 (16.7, 40.9) | 23/1 | 27.2 (17.2, 40.7) | 26.9 (16.7, 40.9) | 21/1 | 29.6 (18.4, 45.3) | 24.7 (14.9, 38.2) | NA | NA | NA | NA | NA | NA |
|  | | 9.8.1 Carcinoma of kidney | 21/1 | 24.8 (15.3, 37.9) | 38.1 (23.0, 59.1) | 21/1 | 39.7 (24.6, 60.7) | 38.7 (23.6, 59.7) | 21/1 | 39.9 (24.7, 61.0) | 38.7 (23.6, 59.7) | 19/0 | 43.2 (26.0, 67.4) | 35.1 (20.8, 55.2) | NA | NA | NA | NA | NA | NA |
|  | | 9.8.1.1 Kidney, adenocarcinoma | 21/1 | 25.0 (15.5, 38.2) | 38.4 (23.2, 59.6) | 21/1 | 40.1 (24.8, 61.3) | 39.0 (23.8, 60.2) | 21/1 | 40.3 (24.9, 61.5) | 39.0 (23.8, 60.2) | 19/0 | 43.5 (26.2, 68.0) | 35.4 (21.0, 55.7) | NA | NA | NA | NA | NA | NA |
|  | | 9.8.1.1.1 Kidney, renal cell | 18/1 | 22.7 (13.4, 35.8) | 35.0 (20.1, 56.3) | 18/0 | 36.4 (21.6, 57.5) | 35.7 (20.7, 56.9) | 18/0 | 36.5 (21.6, 57.7) | 35.7 (20.7, 56.9) | 18/0 | 43.6 (25.9, 69.0) | 35.8 (20.9, 57.1) | NA | NA | NA | NA | NA | NA |
| **Females** | | **9. Carcinomas** | **64/25** | **2.6 (2.0, 3.3)** | **1.1 (0.7, 1.6)** | **34/16** | **2.1 (1.5, 3.0)** | **0.5 (0.2, 0.9)** | **33/16** | **2.1 (1.4, 2.9)** | **0.5 (0.2, 0.9)** | **31/14** | **2.3 (1.5, 3.2)** | **0.5 (0.2, 0.9)** | **25/8** | **3.3 (2.1, 4.9)** | **0.5 (0.2, 0.8)** | **20/6** | **3.1 (1.9, 4.8)** | **0.4 (0.2, 0.7)** |
|  | | 9.6 Carcinoma of breast | 18/13 | 1.3 (0.8, 2.1) | 0.3 (-0.1, 0.8) | 12/9 | 1.4 (0.7, 2.4) | 0.2 (-0.1, 0.7) | 12/9 | 1.4 (0.7, 2.5) | 0.2 (-0.1, 0.7) | 11/7 | 1.5 (0.7, 2.7) | 0.2 (-0.1, 0.7) | NA | NA | NA | NA | NA | NA |
|  | 9.6.1 Breast, infiltrating duct | 13/10 | 1.3 (0.7, 2.2) | 0.2 (-0.2, 0.8) | NA | NA | NA | NA | NA | NA | NA | NA | NA | NA | NA | NA | NA | NA | NA |  |
|  | 9.7 Carcinoma of genital sites excluding ovary and testis | 22/6 | 3.5 (2.2, 5.4) | 1.9 (0.9, 3.3) | NA | NA | NA | NA | NA | NA | NA | NA | NA | 16/2 | 8.3 (4.8, 13.5) | 1.7 (0.9, 2.9) | 12/2 | 7.3 (3.7, 12.7) | 1.2 (0.5, 2.3) |  |
|  | 9.7.1 Carcinoma of uterine cervix | 20/6 | 3.6 (2.2, 5.5) | 1.9 (0.9, 3.3) | NA | NA | NA | NA | NA | NA | NA | NA | NA | 14/2 | 8.1 (4.4, 13.6) | 1.6 (0.8, 2.9) | 10/1 | 6.7 (3.2, 12.4) | 1.1 (0.4, 2.2) |  |
|  | 9.7.1.1 Cervix, squamous | 15/4 | 3.8 (2.1, 6.3) | 2.1 (0.8, 3.9) | NA | NA | NA | NA | NA | NA | NA | NA | NA | 11/1 | 9.0 (4.5, 16.2) | 1.8 (0.8, 3.5) | NA | NA | NA |  |
|  | 9.8 Carcinoma of urinary tract | 11/0 | 30.6 (15.3, 54.7) | 20 (9.7, 36.4) | NA | NA | NA | NA | NA | NA | NA | NA | NA | NA | NA | NA | NA | NA | NA |  |

Abbreviation: NA=Not Applicable.

**Supplementary Table S19A.** Standardised incidence ratios (SIRs) and absolute excess risks (AERs) of second primary other invasive carcinomas after each distinct first primary cancer type among six-month adolescent and young adult (AYA, aged 15-39 years) cancer survivors compared to the general population in the Netherlands. Second primary cancer types are grouped according to the AYA-specific classification scheme developed by Barr and colleagues (2020). First and second cancer combinations with less than n=10 observed second cancers were excluded from the analyses. This Table presents outcomes from the main analysis.

|  | **Second primary cancers** | **9.9 Other invasive carcinomas** | | | **9.9.2 Unknown primary** | | |
| --- | --- | --- | --- | --- | --- | --- | --- |
|  | **First primary cancers** | **Obs/exp** | **SIR (95%CI)** | **AER per 10,000 person-years (95%CI)** | **Obs/exp** | **SIR (95%CI)** | **AER per 10,000 person-years (95%CI)** |
| **Females** | **9. Carcinomas** | **41/9** | **4.5 (3.2, 6.1)** | **0.9 (0.6, 1.3)** | **32/6** | **5.5 (3.7, 7.7)** | **0.7 (0.4, 1.1)** |
|  | 9.6 Carcinoma of breast | 18/5 | 3.4 (2.0, 5.4) | 0.7 (0.3, 1.2) | 13/3 | 3.8 (2.0, 6.6) | 0.5 (0.2, 1.0) |
|  | 9.6.1 Breast, infiltrating duct | 15/4 | 3.7 (2.1, 6.1) | 0.7 (0.3, 1.3) | 12/3 | 4.6 (2.4, 8.1) | 0.6 (0.2, 1.2) |
|  | 9.7 Carcinoma of genital sites excluding ovary and testis | 15/2 | 7.1 (3.9, 11.6) | 1.6 (0.8, 2.7) | 12/1 | 9.0 (4.6, 15.6) | 1.3 (0.6, 2.4) |
|  | 9.7.1 Carcinoma of uterine cervix | 12/2 | 6.3 (3.2, 10.9) | 1.3 (0.6, 2.5) | 10/1 | 8.3 (4.0, 15.2) | 1.2 (0.5, 2.3) |

**Supplementary Table S19B.** Standardised incidence ratios (SIRs) and absolute excess risks (AERs) of second primary other invasive carcinomas after each distinct first primary cancer type among six-month adolescent and young adult (AYA, aged 15-39 years) cancer survivors compared to the general population in the Netherlands. Second primary cancer types are grouped according to the AYA-specific classification scheme developed by Barr and colleagues (2020). First and second cancer combinations with less than n=10 observed second cancers were excluded from the analyses. This Table presents outcomes from the sensitivity analysis.

|  | **Second primary cancers** | **9.9 Other invasive carcinomas** | | | **9.9.2 Unknown primary** | | |
| --- | --- | --- | --- | --- | --- | --- | --- |
|  | **First primary cancers** | **Obs/exp** | **SIR (95%CI)** | **AER per 10,000 person-years (95%CI)** | **Obs/exp** | **SIR (95%CI)** | **AER per 10,000 person-years (95%CI)** |
| **Females** | **9. Carcinomas** | **41/9** | **4.6 (3.3, 6.3)** | **0.9 (0.6, 1.3)** | **32/6** | **5.6 (3.9, 7.9)** | **0.7 (0.5, 1.1)** |
|  | 9.6 Carcinoma of breast | 18/5 | 3.6 (2.1, 5.7) | 0.7 (0.3, 1.3) | 13/3 | 4.0 (2.1, 6.9) | 0.5 (0.2, 1.0) |
|  | 9.6.1 Breast, infiltrating duct | 16/4 | 4.2 (2.4, 6.8) | 0.8 (0.4, 1.5) | 12/2 | 4.8 (2.5, 8.4) | 0.6 (0.3, 1.3) |
|  | 9.7 Carcinoma of genital sites excluding ovary and testis | 15/2 | 7.1 (3.9, 11.6) | 1.6 (0.8, 2.7) | 12/1 | 9.0 (4.6, 15.6) | 1.3 (0.6, 2.4) |
|  | 9.7.1 Carcinoma of uterine cervix | 12/2 | 6.3 (3.2, 10.9) | 1.3 (0.6, 2.5) | 10/1 | 8.3 (4.0, 15.3) | 1.2 (0.5, 2.3) |

**Supplementary Table S20A.** Standardised incidence ratios (SIRs) and absolute excess risks (AERs) of second primary unspecified malignant neoplasms (except central nervous system) after each distinct first primary cancer type among six-month adolescent and young adult (AYA, aged 15-39 years) cancer survivors compared to the general population in the Netherlands. Second primary cancer types are grouped according to the AYA-specific classification scheme developed by Barr and colleagues (2020). First and second cancer combinations with less than n=10 observed second cancers were excluded from the analyses. This Table presents outcomes from the main analysis.

|  | **Second primary cancers** | **11. Unspecified malignant neoplasms except CNS** | | |
| --- | --- | --- | --- | --- |
|  | **First primary cancers** | **Obs/exp** | **SIR (95%CI)** | **AER per 10,000 person-years (95%CI)** |
| **Females** | **9. Carcinomas** | 12/12 | 1.0 (0.5, 1.8) | 0.0 (-0.1, 0.3) |

Abbreviation: CNS=Central Nervous System.

**Supplementary Table S21.** Cumulative incidence of any second primary cancer up-to 25 years after first primary cancer among six-month adolescent and young adult (AYA, aged 15-39 years) cancer survivors in the Netherlands between 1989-2018. Cancer types were grouped according to the AYA-specific classification scheme developed by Barr and colleagues (2020). Death of any cause was included as competing risk event. This Table presents outcomes from the sensitivity analysis.

|  | **Cumulative Incidence (95%CI)** | | | | | | | |
| --- | --- | --- | --- | --- | --- | --- | --- | --- |
|  | **Males** | | | | **Females** | | | |
|  | **5** | **10** | **20** | **25** | **5** | **10** | **20** | **25** |
| **Total** | 1.6 (1.5, 1.8) | 3.4 (3.2, 3.6) | 7.9 (7.5, 8.3) | 11.4 (10.8, 12.0) | 2.5 (2.3, 2.6) | 5.0 (4.8, 5.2) | 11.5 (11.1, 11.9) | 15.6 (15.1, 16.1) |
| **Age at diagnosis (years)^a^** |  |  |  |  |  |  |  |  |
| 15−19 | 0.9 (0.6, 1.3) | 2.1 (1.6, 2.8) | 4.8 (3.8, 5.9) | 5.1 (4.0, 6.3) | 0.9 (0.6, 1.3) | 2.1 (1.5, 2.8) | 5.6 (4.4, 7.0) | 8.5 (6.6, 10.6) |
| 20−24 | 1.6 (1.3, 2.0) | 3.0 (2.5, 3.6) | 6.0 (5.2, 7.0) | 8.7 (7.4, 10.2) | 1.3 (1.0, 1.7) | 2.7 (2.2, 3.3) | 7.0 (5.9, 8.1) | 11.1 (9.6, 12.9) |
| 25−29 | 1.5 (1.3, 1.8) | 3.1 (2.7, 3.6) | 6.5 (5.8, 7.3) | 9.4 (8.3, 10.6) | 2.1 (1.8, 2.4) | 4.2 (3.7, 4.7) | 10.2 (9.3, 11.1) | 14.3 (13.0, 15.6) |
| 30−34 | 1.7 (1.4, 2.0) | 3.3 (2.9, 3.7) | 7.8 (7.1, 8.6) | 11.8 (10.7, 13.1) | 2.5 (2.3, 2.8) | 4.8 (4.4, 5.2) | 11.0 (10.3, 11.7) | 14.4 (13.5, 15.4) |
| 35−39 | 1.9 (1.6, 2.1) | 4.1 (3.7, 4.5) | 10.5 (9.7, 11.3) | 15.1 (13.9, 16.3) | 2.9 (2.7, 3.1) | 6.0 (5.7, 6.3) | 13.5 (12.9, 14.1) | 18.2 (17.4, 19.0) |
| **Tumour stage (TNM, Figo and Ann arbor)** |  |  |  |  |  |  |  |  |
| stage I | 1.9 (1.7, 2.1) | 4.0 (3.7, 4.4) | 9.0 (8.4, 9.6) | 12.8 (11.9, 13.8) | 2.4 (2.2, 2.6) | 5.3 (5.0, 5.6) | 12.4 (11.8, 13.0) | 16.7 (16.0, 17.5) |
| stage II | 1.6 (1.3, 2.0) | 3.4 (2.9, 3.9) | 8.4 (7.4, 9.4) | 12.5 (11.1, 14.1) | 3.0 (2.7, 3.3) | 6.0 (5.6, 6.4) | 13.3 (12.6, 14.1) | 18.1 (17.1, 19.1) |
| stage III | 1.6 (1.3, 2.0) | 3.1 (2.6, 3.7) | 8.7 (7.5, 10.1) | 12.2 (10.3, 14.3) | 3.1 (2.7, 3.6) | 4.6 (4.0, 5.2) | 11.2 (9.9, 12.5) | 14.5 (12.9, 16.3) |
| stage IV | 1.4 (1.0, 1.9) | 3.0 (2.4, 3.8) | 6.2 (5.1, 7.5) | 8.1 (6.7, 9.6) | 1.5 (1.1, 1.9) | 2.6 (2.0, 3.2) | 5.5 (4.4, 6.7) | 8.9 (7.0, 10.9) |
| Other/unknown | 1.2 (1.0, 1.5) | 2.6 (2.2, 3.0) | 5.8 (5.1, 6.5) | 8.5 (7.5, 9.6) | 1.6 (1.3, 1.9) | 3.2 (2.7, 3.6) | 7.7 (6.9, 8.5) | 10.9 (9.8, 12.1) |
| **Cancer types** |  |  |  |  |  |  |  |  |
| **1. Leukaemia’s and related disorders** | **0.9 (0.6, 1.4)** | **2.4 (1.7, 3.2)** | **6.0 (4.6, 7.7)** | **9.6 (7.0, 12.6)** | **1.6 (1.1, 2.2)** | **2.6 (1.9, 3.5)** | **5.6 (4.1, 7.5)** | **9.8 (6.9, 13.2)** |
| 1.1 Acute lymphoblastic leukaemia | 1.3 (0.6, 2.5) | 2.0 (1.0, 3.4) | 4.9 (2.8, 7.7) | 4.9 (2.8, 7.7) | 0.8 (0.2, 2.2) | 1.2 (0.4, 3.1) | 4.3 (1.9, 8.4) | 6.8 (3.2, 12.2) |
| 1.2 Acute myeloid leukaemia | 0.5 (0.2, 1.5) | 2.3 (1.2, 4.0) | 5.4 (3.2, 8.5) | 7.5 (4.3, 11.9) | 2.0 (1.1, 3.4) | 2.9 (1.7, 4.4) | 5.2 (3.2, 7.9) | 9.7 (5.8, 14.7) |
| 1.2.1 Acute promyelocytic leukaemia | NA | NA | NA | NA | 3.2 (0.9, 8.4) | 5.9 (2.2, 12.4) | 11.5 (4.4, 22.4) | 32.4 (14.7, 51.5) |
| 1.2.2 Other acute myeloid leukaemia | 0.4 (0.1, 1.4) | 1.9 (0.9, 3.6) | 4.3 (2.3, 7.3) | 5.5 (2.8, 9.6) | 1.8 (0.9, 3.2) | 2.3 (1.3, 3.9) | 4.1 (2.3, 6.6) | 4.8 (2.7, 7.9) |
| 1.3 Chronic myeloid leukaemia | 1.6 (0.7, 3.3) | 2.5 (1.2, 4.6) | 6.9 (3.4, 12.2) | 12.2 (4.1, 24.8) | 0.8 (0.2, 2.8) | 3.1 (1.3, 6.3) | 4.8 (2.2, 9.0) | 10.5 (4.0, 20.7) |
| 1.4 Chronic lymphocytic leukaemia | 1.8 (0.4, 5.8) | 6.7 (2.7, 13.1) | 12.0 (5.7, 20.9) | 25.3 (11.7, 41.5) | NA | NA | NA | NA |
| **2. Lymphomas** | **1.1 (0.8, 1.4)** | **2.6 (2.2, 3.1)** | **8.9 (7.8, 10.0)** | **13.6 (12.0, 15.2)** | **0.9 (0.7, 1.3)** | **2.9 (2.4, 3.5)** | **12.1 (10.7, 13.5)** | **19.4 (17.2, 21.6)** |
| 2.1 Non-Hodgkin lymphomas | 1.0 (0.7, 1.5) | 2.5 (1.9, 3.2) | 9.2 (7.6, 11.1) | 12.4 (10.2, 14.9) | 1.2 (0.7, 1.8) | 3.5 (2.6, 4.7) | 9.7 (7.7, 11.9) | 17.2 (13.6, 21.2) |
| 2.1.3 Diffuse large B-cell (DLBCL) | 1.3 (0.7, 2.2) | 2.8 (1.8, 4.0) | 9.9 (7.3, 12.8) | 14.2 (10.4, 18.6) | 0.8 (0.3, 1.8) | 3.2 (1.9, 5.0) | 9.4 (6.3, 13.3) | 14.3 (9.2, 20.5) |
| 2.1.5 Anaplastic T-cell and null-cell excluding NK/T-cell | 0.2 (0.0, 1.3) | 1.3 (0.4, 3.1) | 9.3 (5.4, 14.6) | 10.8 (6.2, 16.9) | 1.2 (0.3, 3.2) | 2.2 (0.8, 4.9) | 8.1 (4.0, 14.0) | 19.3 (10.3, 30.3) |
| 2.1.6 Follicular | 1.0 (0.3, 2.4) | 3.4 (1.9, 5.7) | 11.3 (7.4, 16.0) | 15.6 (9.9, 22.5) | 1.5 (0.5, 3.5) | 3.5 (1.7, 6.3) | 10.2 (6.3, 15.1) | 19.1 (11.4, 28.4) |
| 2.1.9 Other non-Hodgkin lymphoma NOS | 0.6 (0.1, 3.1) | 3.5 (1.3, 7.4) | 9.2 (4.8, 15.2) | 10.5 (5.6, 17.0) | NA | NA | NA | NA |
| 2.2 Hodgkin lymphoma | 1.0 (0.7, 1.5) | 2.7 (2.1, 3.3) | 8.7 (7.4, 10.3) | 15.1 (12.8, 17.5) | 0.8 (0.5, 1.2) | 2.7 (2.1, 3.5) | 13.8 (11.8, 15.9) | 21.2 (18.3, 24.2) |
| 2.2.1 Hodgkin NLP | NA | 1.3 (0.2, 4.2) | 10.3 (4.9, 18.1) | 16.8 (6.0, 32.5) | NA | NA | NA | NA |
| 2.2.2 Hodgkin classic, other | 1.1 (0.8, 1.6) | 2.8 (2.2, 3.5) | 8.6 (7.2, 10.2) | 15.0 (12.7, 17.5) | 0.8 (0.5, 1.2) | 2.7 (2.1, 3.5) | 13.8 (11.9, 15.9) | 21.0 (18.2, 24.1) |
| **3. CNS and other intracranial and intraspinal neoplasms** | **0.7 (0.4, 1.2)** | **1.7 (1.2, 2.3)** | **3.0 (2.2, 3.9)** | **4.2 (3.1, 5.6)** | **1.0 (0.6, 1.6)** | **1.8 (1.2, 2.6)** | **5.1 (3.8, 6.7)** | **5.8 (4.3, 7.5)** |
| 3.1 Astroglial and related neoplasms | 0.8 (0.5, 1.2) | 1.8 (1.2, 2.4) | 3.1 (2.3, 4.1) | 4.0 (2.9, 5.4) | 1.1 (0.6, 1.7) | 1.9 (1.3, 2.8) | 5.0 (3.7, 6.7) | 5.6 (4.0, 7.4) |
| 3.1.1 Oligodendriogliomas | 1.3 (0.5, 2.6) | 2.2 (1.1, 3.9) | 3.3 (1.8, 5.5) | 4.9 (2.6, 8.3) | 1.0 (0.3, 2.6) | 2.4 (1.1, 4.7) | 4.3 (2.1, 7.8) | 4.3 (2.1, 7.8) |
| 3.1.1.2 Oligodendrioglioma, invasive | 1.3 (0.5, 2.6) | 2.2 (1.1, 3.9) | 3.3 (1.8, 5.5) | 4.9 (2.6, 8.3) | 1.0 (0.3, 2.6) | 2.4 (1.1, 4.7) | 4.3 (2.1, 7.8) | 4.3 (2.1, 7.8) |
| 3.1.4 Other astrocytoma/astroglial neoplasms | 0.6 (0.3, 1.2) | 1.8 (1.1, 2.8) | 2.5 (1.6, 3.6) | 3.3 (2.1, 5.0) | 1.1 (0.6, 2.0) | 1.7 (1.0, 2.8) | 5.0 (3.3, 7.2) | 5.5 (3.6, 8.0) |
| 3.1.4.3 Other astrocytoma/astroglial, invasive | 0.7 (0.3, 1.3) | 1.8 (1.1, 2.9) | 2.6 (1.7, 3.9) | 3.3 (2.0, 4.9) | 1.0 (0.5, 2.0) | 1.7 (0.9, 2.9) | 4.7 (3.0, 7.0) | 5.3 (3.3, 7.9) |
| **4. Sarcomas** | **1.2 (0.8, 1.8)** | **2.1 (1.5, 2.7)** | **4.5 (3.5, 5.7)** | **7.3 (5.7, 9.3)** | **1.1 (0.7, 1.7)** | **3.3 (2.5, 4.2)** | **7.7 (6.3, 9.4)** | **10.1 (8.2, 12.3)** |
| 4.1 Osteosarcoma | 0.7 (0.1, 2.3) | 0.7 (0.1, 2.3) | 1.7 (0.4, 5.0) | 3.0 (0.8, 7.7) | 1.0 (0.2, 3.2) | 4.0 (1.8, 7.7) | 8.1 (4.1, 13.9) | 11.1 (5.1, 19.9) |
| 4.2 Chondrosarcoma | 1.8 (0.8, 3.8) | 3.8 (1.9, 6.7) | 6.8 (3.4, 11.8) | 6.8 (3.4, 11.8) | 2.5 (1.2, 4.7) | 6.2 (3.7, 9.7) | 11.0 (6.3, 17.3) | 11.0 (6.3, 17.3) |
| 4.4 Fibromatous neoplasms | 1.6 (0.8, 3.1) | 3.0 (1.7, 4.9) | 6.0 (3.9, 8.8) | 11.6 (7.6, 16.5) | 0.6 (0.2, 1.6) | 2.4 (1.3, 4.1) | 6.7 (4.2, 10.0) | 8.4 (5.4, 12.3) |
| 4.4.3 Other fibromatous neoplasms | 1.3 (0.5, 2.8) | 3.0 (1.6, 5.2) | 5.2 (3.0, 8.2) | 11.6 (7.0, 17.5) | 0.2 (0.0, 1.2) | 2.4 (1.2, 4.4) | 7.0 (4.1, 10.7) | 8.4 (5.1, 12.8) |
| 4.5 Liposarcoma | 1.1 (0.2, 3.6) | 1.8 (0.5, 4.7) | 7.0 (3.2, 12.9) | 11.3 (5.2, 19.9) | 1.7 (0.5, 4.5) | 6.6 (3.3, 11.3) | 13.6 (8.3, 20.2) | 16.4 (9.4, 25.0) |
| **5. Blood and lymphatic vessel tumours** | **3.2 (2.1, 4.9)** | **5.1 (3.5, 7.2)** | **8.9 (6.4, 11.9)** | **11.2 (7.3, 15.9)** | **2.7 (0.7, 6.9)** | **2.7 (0.7, 6.9)** | **4.5 (1.3, 10.6)** | **7.7 (2.3, 17.3)** |
| 5.2 Malignant blood and lymphatic vessel tumours, all sites | 3.2 (2.1, 4.9) | 5.1 (3.5, 7.2) | 8.9 (6.4, 11.9) | 11.2 (7.3, 15.9) | 2.7 (0.7, 6.9) | 2.7 (0.7, 6.9) | 4.5 (1.3, 10.6) | 7.7 (2.3, 17.3) |
| 5.2.1 Kaposi sarcoma | 3.4 (2.1, 5.1) | 5.3 (3.6, 7.5) | 9.3 (6.7, 12.5) | 11.9 (7.6, 17.1) | 2.4 (0.2, 10.8) | 2.4 (0.2, 10.8) | 8.0 (1.2, 23.8) | 8.0 (1.2, 23.8) |
| **7. Gonadal and related tumours** | **1.8 (1.5, 2.0)** | **3.9 (3.5, 4.3)** | **8.0 (7.3, 8.8)** | **11.4 (10.3, 12.5)** | **1.4 (1.0, 2.0)** | **2.8 (2.1, 3.6)** | **7.7 (6.4, 9.1)** | **10.8 (9.0, 12.7)** |
| 7.1 Testis | 1.7 (1.5, 2.0) | 3.8 (3.4, 4.2) | 7.9 (7.2, 8.7) | 11.2 (10.1, 12.4) | NA | NA | NA | NA |
| 7.1.1 Germ cell and trophoblastic | 1.7 (1.5, 2.0) | 3.8 (3.4, 4.2) | 7.9 (7.2, 8.6) | 11.2 (10.1, 12.4) | NA | NA | NA | NA |
| 7.1.1.1 Seminoma | 2.1 (1.8, 2.6) | 4.4 (3.8, 5.1) | 8.8 (7.7, 10.0) | 12.5 (10.8, 14.4) | NA | NA | NA | NA |
| 7.1.1.2 Embryonal carcinoma | 1.0 (0.6, 1.7) | 2.7 (1.9, 3.8) | 6.0 (4.4, 7.9) | 9.4 (6.8, 12.5) | NA | NA | NA | NA |
| 7.1.1.4 Teratoma | 1.4 (0.9, 2.1) | 3.5 (2.6, 4.6) | 6.8 (5.4, 8.5) | 10.3 (8.2, 12.7) | NA | NA | NA | NA |
| 7.1.1.5 Mixed germ cell | 1.9 (1.3, 2.6) | 3.3 (2.5, 4.3) | 7.8 (5.9, 10.2) | 10.1 (6.8, 14.3) | NA | NA | NA | NA |
| 7.1.1.6 Choriocarcinoma and other trophoblastic | 0.7 (0.2, 1.9) | 3.1 (1.7, 5.2) | 7.9 (4.9, 11.9) | 8.7 (5.4, 13.0) | NA | NA | NA | NA |
| 7.1.1.7 Other | 0.8 (0.1, 4.2) | 4.5 (1.4, 10.4) | 8.2 (3.2, 16.3) | 8.2 (3.2, 16.3) | NA | NA | NA | NA |
| 7.2 Ovary | NA | NA | NA | NA | 1.5 (1.0, 2.1) | 2.9 (2.2, 3.7) | 7.6 (6.3, 9.0) | 10.9 (9.0, 13.0) |
| 7.2.1 Germ cell and trophoblastic | NA | NA | NA | NA | 1.9 (0.8, 3.8) | 2.7 (1.3, 5.1) | 7.0 (3.8, 11.6) | 9.6 (5.2, 15.6) |
| 7.2.2 Non-germ cell | NA | NA | NA | NA | 1.4 (0.9, 2.1) | 2.9 (2.1, 3.8) | 7.5 (6.2, 9.1) | 10.9 (8.9, 13.1) |
| 7.2.2.1 Carcinoma | NA | NA | NA | NA | 1.5 (1.0, 2.2) | 3.0 (2.2, 3.9) | 7.6 (6.2, 9.2) | 10.8 (8.8, 13.0) |
| 7.2.2.1.1 Adenocarcinoma | NA | NA | NA | NA | 1.5 (1.0, 2.3) | 3.0 (2.2, 4.0) | 7.7 (6.3, 9.3) | 10.9 (8.9, 13.2) |
| 7.2.2.1.2 Other carcinoma | NA | NA | NA | NA | NA | NA | 4.6 (0.3, 18.9) | 4.6 (0.3, 18.9) |
| 7.2.2.2 Sex cord and other specialized gonadal | NA | NA | NA | NA | NA | NA | 5.0 (0.9, 14.7) | 13.3 (3.9, 28.5) |
| 7.4 Germ cell and trophoblastic excluding CNS, ovary, testis | 1.8 (0.6, 4.2) | 6.5 (3.6, 10.5) | 11.6 (7.1, 17.4) | 16.4 (10.0, 24.1) | 0.5 (0.1, 2.8) | 2.5 (0.8, 5.9) | 11.0 (5.6, 18.4) | 11.0 (5.6, 18.4) |
| 7.4.1 Germ cell tumours including non-gestational Trophoblastic tumours | 1.4 (0.3, 4.5) | 5.3 (2.4, 10.1) | 9.5 (4.6, 16.5) | 16.5 (8.5, 26.9) | NA | NA | 26.7 (1.0, 68.6) | 26.7 (1.0, 68.6) |
| 7.4.2 Gestational Trophoblastic tumours | 2.6 (0.5, 8.1) | 8.3 (3.4, 16.1) | 14.9 (7.1, 25.5) | 14.9 (7.1, 25.5) | 0.6 (0.1, 2.8) | 2.6 (0.9, 6.1) | 10.2 (5.0, 17.6) | 10.2 (5.0, 17.6) |
| **8. Melanoma, malignant** | **2.1 (1.7, 2.5)** | **4.0 (3.5, 4.6)** | **8.8 (7.8, 9.9)** | **11.9 (10.5, 13.4)** | **2.7 (2.4, 3.0)** | **5.4 (5.0, 5.9)** | **12.2 (11.3, 13.0)** | **15.6 (14.5, 16.8)** |
| 8.1 Superficial spreading/low cumulative sun damage melanoma | 2.3 (1.8, 2.8) | 4.5 (3.8, 5.3) | 10.7 (9.2, 12.3) | 13.3 (11.4, 15.5) | 2.7 (2.3, 3.1) | 5.5 (5.0, 6.1) | 13.2 (12.0, 14.4) | 16.3 (14.7, 17.9) |
| 8.2 Nodular melanoma | 2.3 (1.3, 3.7) | 3.3 (2.0, 5.0) | 7.3 (5.0, 10.1) | 7.3 (5.0, 10.1) | 3.7 (2.6, 5.1) | 6.9 (5.2, 8.8) | 11.6 (9.2, 14.4) | 14.9 (11.4, 18.8) |
| 8.3 Other malignant | 1.5 (0.9, 2.2) | 3.3 (2.4, 4.3) | 5.9 (4.5, 7.6) | 10.4 (8.0, 13.0) | 2.2 (1.6, 2.8) | 4.6 (3.8, 5.6) | 10.0 (8.6, 11.5) | 14.0 (12.1, 16.0) |
| **9. Carcinomas** | **2.2 (1.9, 2.6)** | **4.3 (3.8, 4.8)** | **9.7 (8.7, 10.7)** | **13.5 (12.1, 14.9)** | **2.9 (2.7, 3.1)** | **5.7 (5.4, 5.9)** | **12.4 (11.9, 12.9)** | **16.7 (16.0, 17.3)** |
| 9.1 Thyroid carcinoma | 0.9 (0.4, 1.8) | 2.2 (1.3, 3.6) | 6.9 (4.5, 9.9) | 10.5 (7.1, 14.8) | 0.8 (0.5, 1.2) | 2.3 (1.7, 3.1) | 7.5 (6.0, 9.1) | 12.4 (10.0, 15.0) |
| 9.1.1 Medullary | 2.2 (0.4, 7.0) | 4.8 (1.5, 10.8) | 10.3 (4.4, 19.3) | 13.0 (5.8, 23.3) | 1.0 (0.1, 4.8) | 2.2 (0.4, 7.1) | 10.5 (4.5, 19.5) | 19.5 (9.4, 32.4) |
| 9.1.3 Papillary | 1.0 (0.3, 2.3) | 1.6 (0.7, 3.4) | 5.6 (2.8, 9.6) | 10.7 (5.8, 17.4) | 0.5 (0.2, 1.0) | 2.0 (1.3, 3.0) | 6.3 (4.4, 8.7) | 9.6 (6.6, 13.3) |
| 9.1.4 Follicular | NA | 1.7 (0.1, 8.0) | 5.8 (1.5, 14.5) | 5.8 (1.5, 14.5) | 1.5 (0.6, 3.3) | 3.6 (1.9, 6.2) | 8.9 (5.6, 13.2) | 13.6 (8.4, 20.0) |
| 9.1.5 Papillary with follicular variant | 0.8 (0.1, 3.8) | 2.7 (0.7, 7.2) | 9.8 (3.5, 19.8) | 13.3 (5.1, 25.4) | 0.9 (0.4, 2.1) | 2.6 (1.4, 4.3) | 8.7 (5.9, 12.3) | 13.6 (9.4, 18.6) |
| 9.2 Other carcinoma of head and neck | 2.8 (1.9, 4.0) | 5.5 (4.1, 7.1) | 15.5 (12.5, 18.8) | 19.8 (16.0, 24.0) | 3.9 (2.7, 5.5) | 6.5 (4.8, 8.5) | 16.3 (13.0, 20.0) | 21.7 (17.1, 26.5) |
| 9.2.1 Nasopharyngeal carcinoma | 1.3 (0.3, 4.4) | 3.8 (1.4, 8.2) | 15.2 (6.6, 27.0) | 15.2 (6.6, 27.0) | NA | 2.9 (0.5, 9.2) | 8.8 (2.6, 20.0) | 16.1 (6.2, 30.2) |
| 9.2.2 Oral cavity, lip, and pharynx | 4.1 (2.7, 6.0) | 7.1 (5.1, 9.7) | 17.2 (13.1, 21.7) | 22.2 (16.8, 28.0) | 5.2 (3.2, 8.0) | 8.6 (5.8, 12.2) | 23.0 (17.2, 29.3) | 28.3 (21.0, 36.0) |
| 9.2.2.1 Oral cavity, lip, and pharynx, squamous | 4.7 (3.0, 6.9) | 8.3 (5.9, 11.3) | 19.3 (14.6, 24.5) | 25.4 (19.1, 32.1) | 6.6 (3.9, 10.4) | 11.3 (7.5, 16.1) | 29.5 (22.0, 37.4) | 36.2 (27.0, 45.4) |
| 9.2.3 Salivary gland | NA | NA | 4.6 (0.8, 13.9) | 8.8 (2.1, 21.9) | 2.7 (1.1, 5.6) | 3.9 (1.8, 7.3) | 8.4 (4.5, 13.8) | 13.3 (6.6, 22.3) |
| 9.2.3.2 Salivary gland, other malignant | NA | NA | NA | NA | 3.4 (1.3, 7.3) | 5.1 (2.2, 9.7) | 10.1 (5.1, 17.0) | 12.9 (6.3, 21.9) |
| 9.2.4 Other carcinoma of head and neck | 2.4 (0.8, 5.7) | 6.6 (3.3, 11.2) | 20.2 (13.1, 28.4) | 24.4 (15.9, 33.9) | 5.5 (2.0, 11.4) | 8.0 (3.5, 15.0) | 19.3 (10.6, 30.0) | 23.1 (12.6, 35.5) |
| 9.3 Carcinoma of gastrointestinal tract | 2.1 (1.6, 2.7) | 4.0 (3.2, 4.9) | 7.8 (6.5, 9.3) | 11.1 (9.2, 13.2) | 1.9 (1.5, 2.5) | 3.0 (2.4, 3.7) | 7.7 (6.5, 9.2) | 10.8 (8.9, 12.8) |
| 9.3.2 Carcinoma of stomach | 1.1 (0.4, 2.8) | 1.9 (0.8, 4.0) | 3.3 (1.4, 6.4) | 5.4 (2.5, 10.0) | 1.6 (0.6, 3.6) | 2.1 (0.9, 4.4) | 4.3 (2.0, 8.0) | 4.3 (2.0, 8.0) |
| 9.3.4 Carcinoma of colon | 3.1 (2.2, 4.2) | 5.6 (4.2, 7.2) | 10.0 (7.9, 12.4) | 15.6 (12.3, 19.3) | 1.8 (1.2, 2.6) | 3.1 (2.2, 4.2) | 8.5 (6.7, 10.6) | 12.6 (9.9, 15.7) |
| 9.3.4.1 Appendix | 1.2 (0.3, 3.1) | 2.4 (0.9, 5.3) | 4.6 (2.1, 8.6) | 5.5 (2.6, 10.0) | NA | 0.3 (0.0, 1.7) | 2.2 (0.9, 4.5) | 7.8 (4.1, 13.0) |
| 9.3.4.1.1 NET | 0.4 (0.0, 2.2) | 1.8 (0.5, 4.8) | 4.2 (1.7, 8.5) | 5.1 (2.2, 9.9) | NA | 0.4 (0.0, 1.9) | 2.0 (0.7, 4.4) | 8.1 (4.1, 13.7) |
| 9.3.4.2 Colon excluding appendix | 3.6 (2.5, 5.1) | 6.4 (4.8, 8.4) | 11.7 (9.1, 14.7) | 19.7 (15.2, 24.6) | 2.7 (1.8, 3.9) | 4.4 (3.2, 6.0) | 12.5 (9.7, 15.7) | 15.9 (12.2, 20.0) |
| 9.3.4.2.2 Colon excluding appendix, adenocarcinoma | 3.6 (2.5, 5.0) | 6.5 (4.8, 8.5) | 11.9 (9.2, 15.0) | 19.6 (15.1, 24.6) | 2.7 (1.7, 3.9) | 4.4 (3.1, 6.0) | 12.4 (9.6, 15.6) | 15.9 (12.2, 20.0) |
| 9.3.5 Carcinoma of rectum | 1.4 (0.7, 2.5) | 2.8 (1.7, 4.5) | 7.0 (4.5, 10.2) | 7.8 (5.0, 11.4) | 1.8 (1.0, 3.2) | 3.0 (1.7, 4.7) | 7.3 (4.7, 10.5) | 10.7 (6.6, 16.0) |
| 9.3.5.2 Rectum, adenocarcinoma | 1.5 (0.8, 2.8) | 3.1 (1.8, 4.9) | 6.7 (4.3, 9.9) | 7.5 (4.8, 11.1) | 1.9 (1.0, 3.3) | 2.9 (1.7, 4.7) | 6.2 (3.9, 9.3) | 9.7 (5.7, 15.0) |
| 9.4 Carcinoma of lung, bronchus, and trachea | 1.6 (0.8, 2.9) | 2.5 (1.4, 4.1) | 6.2 (4.0, 9.2) | 10.1 (6.2, 15.0) | 1.9 (1.1, 3.0) | 3.4 (2.3, 4.8) | 5.7 (4.0, 7.9) | 7.5 (5.2, 10.4) |
| 9.4.2 Non-small cell carcinoma | 1.5 (0.7, 2.8) | 2.5 (1.3, 4.2) | 5.7 (3.4, 8.6) | 10.2 (5.9, 15.9) | 1.9 (1.1, 3.1) | 3.6 (2.4, 5.2) | 6.2 (4.3, 8.6) | 8.2 (5.7, 11.4) |
| 9.4.2.1 Non-small cell, adenocarcinoma | NA | 0.7 (0.1, 3.6) | 3.4 (0.9, 8.8) | 6.2 (1.9, 14.4) | 2.6 (1.3, 4.7) | 3.7 (2.0, 6.3) | 5.8 (3.3, 9.4) | 7.1 (3.9, 11.6) |
| 9.4.2.2 Non-small cell, neuroendocrine | 2.9 (0.9, 6.7) | 2.9 (0.9, 6.7) | 8.4 (3.7, 15.6) | 17.5 (6.4, 33.1) | 0.4 (0.0, 2.1) | 2.6 (1.0, 5.7) | 6.4 (3.0, 11.7) | 8.2 (3.8, 14.7) |
| 9.4.2.3 Non-small cell, other | 2.1 (0.7, 5.0) | 4.2 (1.8, 8.0) | 6.2 (3.0, 11.1) | 8.2 (3.9, 14.5) | 2.6 (1.0, 5.6) | 4.3 (2.0, 7.9) | 6.3 (3.2, 11.0) | 9.6 (5.0, 16.0) |
| 9.5 Carcinoma of skin (if collected) | 4.0 (2.4, 6.2) | 7.4 (5.0, 10.4) | 15.6 (11.1, 20.8) | 19.1 (13.7, 25.3) | 3.0 (1.7, 4.9) | 7.1 (4.9, 10.0) | 13.4 (9.5, 18.0) | 19.4 (13.4, 26.3) |
| 9.6 Carcinoma of breast | NA | NA | 11.6 (0.7, 39.0) | 11.6 (0.7, 39.0) | 3.8 (3.5, 4.0) | 7.4 (7.0, 7.8) | 15.2 (14.5, 16.0) | 19.9 (18.9, 20.9) |
| 9.6.1 Breast, infiltrating duct | NA | NA | NA | NA | 3.7 (3.4, 4.0) | 7.2 (6.8, 7.7) | 15.2 (14.4, 16.0) | 19.5 (18.4, 20.6) |
| 9.6.2 Breast, adenocarcinoma | NA | NA | 22.2 (1.0, 61.5) | 22.2 (1.0, 61.5) | 3.4 (2.5, 4.4) | 6.4 (5.2, 7.8) | 14.7 (12.6, 16.9) | 18.8 (16.1, 21.6) |
| 9.6.3 Breast, lobular | NA | NA | NA | NA | 4.8 (3.5, 6.4) | 8.2 (6.4, 10.3) | 16.4 (13.5, 19.6) | 21.6 (17.6, 25.9) |
| 9.6.5 Breast, medullary | NA | NA | NA | NA | 4.7 (2.9, 7.0) | 12.3 (9.2, 15.9) | 21.5 (17.0, 26.4) | 30.6 (24.1, 37.3) |
| 9.6.10 Breast, other | NA | NA | NA | NA | 4.8 (3.0, 7.3) | 9.6 (6.8, 12.9) | 15.4 (11.8, 19.5) | 20.4 (15.8, 25.4) |
| 9.7 Carcinoma of genital sites excluding ovary and testis | 2.4 (0.5, 7.6) | 4.1 (1.1, 10.7) | 7.9 (2.1, 19.1) | 7.9 (2.1, 19.1) | 1.6 (1.3, 1.9) | 3.4 (2.9, 3.9) | 9.2 (8.3, 10.2) | 13.5 (12.2, 14.8) |
| 9.7.1 Carcinoma of uterine cervix | NA | NA | NA | NA | 1.5 (1.2, 1.8) | 3.1 (2.6, 3.6) | 8.8 (7.9, 9.8) | 13.2 (11.8, 14.6) |
| 9.7.1.1 Cervix, squamous | NA | NA | NA | NA | 1.4 (1.1, 1.8) | 3.2 (2.7, 3.8) | 8.5 (7.4, 9.6) | 12.6 (11.0, 14.3) |
| 9.7.1.2 Cervix, adenosquamous | NA | NA | NA | NA | NA | 3.7 (1.5, 7.5) | 8.3 (4.3, 13.9) | 13.4 (7.1, 21.9) |
| 9.7.1.3 Cervix, adenocarcinoma | NA | NA | NA | NA | 2.1 (1.4, 3.1) | 3.1 (2.2, 4.4) | 11.5 (9.1, 14.3) | 16.7 (13.2, 20.4) |
| 9.7.1.4 Cervix, other | NA | NA | NA | NA | 1.4 (0.5, 3.3) | 1.4 (0.5, 3.3) | 5.5 (3.2, 8.9) | 10.2 (6.4, 15.1) |
| 9.7.2 Corpus uteri | NA | NA | NA | NA | 1.1 (0.3, 2.9) | 3.8 (1.8, 6.8) | 8.6 (5.0, 13.4) | 14.0 (7.8, 21.9) |
| 9.7.2.1 Corpus uteri, adenocarcinoma | NA | NA | NA | NA | 1.4 (0.4, 3.7) | 5.0 (2.4, 8.9) | 10.0 (5.5, 16.0) | 15.7 (7.9, 25.9) |
| 9.7.2.1.2 Corpus uteri, other adenocarcinoma | NA | NA | NA | NA | 1.8 (0.4, 5.8) | 7.6 (3.5, 13.6) | 13.4 (7.5, 21.0) | 18.7 (10.2, 29.2) |
| 9.7.3 Carcinoma of vulva and vagina | NA | NA | NA | NA | 3.4 (1.7, 5.9) | 9.0 (5.9, 13.0) | 18.4 (13.1, 24.4) | 19.4 (13.9, 25.7) |
| 9.8 Carcinoma of urinary tract | 3.0 (2.0, 4.4) | 6.0 (4.4, 8.0) | 12.5 (9.5, 15.8) | 17.3 (13.1, 21.9) | 2.8 (1.6, 4.5) | 5.0 (3.2, 7.3) | 11.9 (8.3, 16.2) | 16.0 (11.0, 21.9) |
| 9.8.1 Carcinoma of kidney | 3.6 (2.3, 5.5) | 7.1 (4.9, 9.7) | 14.6 (10.4, 19.4) | 18.1 (12.7, 24.3) | 2.7 (1.4, 5.0) | 5.3 (3.1, 8.4) | 9.8 (5.9, 14.8) | 13.0 (7.7, 19.8) |
| 9.8.1.1 Kidney, adenocarcinoma | 3.7 (2.3, 5.5) | 7.1 (4.9, 9.8) | 14.7 (10.5, 19.6) | 18.3 (12.8, 24.5) | 2.7 (1.4, 5.0) | 5.3 (3.1, 8.4) | 9.8 (5.9, 14.8) | 13.0 (7.7, 19.8) |
| 9.8.1.1.1 Kidney, renal cell | 3.4 (2.0, 5.3) | 6.2 (4.1, 8.8) | 14.2 (9.9, 19.3) | 17.0 (11.6, 23.2) | 3.1 (1.5, 5.6) | 5.4 (3.1, 8.8) | 10.3 (6.2, 15.7) | 13.8 (8.1, 21.0) |
| 9.8.2 Carcinoma of bladder | 1.3 (0.4, 3.5) | 3.8 (1.8, 7.0) | 8.7 (5.0, 13.6) | 16.0 (9.4, 24.0) | 2.0 (0.6, 5.3) | 3.6 (1.4, 7.8) | 12.6 (6.7, 20.3) | 18.9 (9.6, 30.6) |
| 9.8.2.1 Urinary bladder, transitional cell carcinoma | 1.0 (0.2, 3.3) | 3.9 (1.7, 7.6) | 8.1 (4.3, 13.4) | 15.0 (8.2, 23.5) | 2.7 (0.7, 7.1) | 4.9 (1.8, 10.2) | 15.4 (7.9, 25.2) | 18.4 (9.6, 29.4) |
| 9.9 Other invasive carcinomas | 0.8 (0.2, 2.5) | 3.3 (1.4, 6.3) | 4.0 (1.9, 7.5) | 10.7 (4.8, 19.4) | 2.8 (1.4, 5.0) | 4.3 (2.4, 7.0) | 6.3 (3.6, 9.9) | 6.3 (3.6, 9.9) |
| **10. Miscellaneous specified neoplasms** | **1.0 (0.1, 4.9)** | **1.0 (0.1, 4.9)** | **1.0 (0.1, 4.9)** | **1.0 (0.1, 4.9)** | **2.3 (0.6, 6.0)** | **3.1 (1.0, 7.2)** | **8.7 (3.9, 15.9)** | **12.4 (6.1, 21.1)** |

Abbreviation: TNM=Tumour, Node, Metastasis, Figo=Fédération Internationale de Gynécologie et d'Obstétrique, NOS=Not otherwise specified, CNS=Central Nervous System, NA=Not Applicable.

^a^ Age at diagnosis of first primary cancer.

**Supplementary Table S22.** Standardised incidence ratios (SIRs) and absolute excess risks (AERs) of any second primary malignant cancer diagnosis after first primary malignant cancer among six-month adolescent and young adult (AYA, aged 15-39 years) cancer survivors compared to the general population in the Netherlands. Cancer types are grouped according to the AYA-specific classification scheme developed by Barr and colleagues (2020). Cancer combinations with less than n=10 observed second cancers were excluded from the analyses. This Table presents outcomes from the sensitivity analysis.

| **First primary cancers** | **Second primary cancer risk** | | | | | | | | | |
| --- | --- | --- | --- | --- | --- | --- | --- | --- | --- | --- |
|  | **Males** | | | | | **Females** | | | | |
|  | **Person-years** | **Obs/exp** | **SIR (95%CI)** | **AER per 10,000 person-years (95%CI)** | **P-value** | **Person-years** | **Obs/exp** | **SIR (95%CI)** | **AER per 10,000 person-years (95%CI)** | **P-value** |
| **Total** | 404,409.7 | 2,018/702 | 2.9 (2.7, 3.0) | 32.5 (30.4, 34.8) | 0.000 | 622,548.1 | 4,549/2,093 | 2.2 (2.1, 2.2) | 39.5 (37.3, 41.6) | 0.000 |
| **Age at diagnosis (years)^a^** |  |  |  |  |  |  |  |  |  |  |
| 15-19 | 33,554.0 | 92/20 | 4.7 (3.8, 5.7) | 21.6 (16.2, 27.8) | 0.000 | 28,490.7 | 96/22 | 4.3 (3.5, 5.3) | 25.9 (19.5, 33.4) | 0.000 |
| 20-24 | 55,950.3 | 215/47 | 4.5 (3.9, 5.2) | 30.0 (25.0, 35.4) | 0.000 | 49,823.7 | 221/67 | 3.3 (2.9, 3.8) | 30.9 (25.3, 37.2) | 0.000 |
| 25-29 | 83,600.9 | 341/101 | 3.4 (3.0, 3.8) | 28.8 (24.5, 33.3) | 0.000 | 93,256.5 | 574/200 | 2.9 (2.6, 3.1) | 40.1 (35.2, 45.4) | 0.000 |
| 30-34 | 105,874.1 | 527/189 | 2.8 (2.6, 3.0) | 31.9 (27.8, 36.4) | 0.000 | 175,670.2 | 1,212/559 | 2.2 (2.0, 2.3) | 37.2 (33.3, 41.2) | 0.000 |
| 35-39 | 125,430.5 | 843/345 | 2.4 (2.3, 2.6) | 39.7 (35.2, 44.4) | 0.000 | 275,307.0 | 2,446/1,245 | 2.0 (1.9, 2.0) | 43.6 (40.1, 47.2) | 0.000 |
| **Tumour stage (TNM, Figo and Ann arbor)** |  |  |  |  |  |  |  |  |  |  |
| stage I | 195,589.6 | 950/353 | 2.7 (2.5, 2.9) | 30.5 (27.5, 33.7) | 0.000 | 320,247.0 | 2,177/1,118 | 1.9 (1.9, 2.0) | 33.1 (30.3, 36.0) | 0.000 |
| stage II | 71,277.6 | 355/125 | 2.8 (2.6, 3.2) | 32.3 (27.3, 37.8) | 0.000 | 165,101.5 | 1,441/571 | 2.5 (2.4, 2.7) | 52.7 (48.2, 57.3) | 0.000 |
| stage III | 41,756.4 | 227/66 | 3.5 (3.0, 3.9) | 38.6 (31.8, 46.2) | 0.000 | 44,098.0 | 386/133 | 2.9 (2.6, 3.2) | 57.3 (48.8, 66.5) | 0.000 |
| stage IV | 21,830.5 | 138/38 | 3.6 (3.1, 4.3) | 45.8 (35.7, 57.3) | 0.000 | 15,588.5 | 117/42 | 2.8 (2.3, 3.4) | 48.4 (35.4, 63.3) | 0.000 |
| Other/unknown | 73,955.6 | 348/121 | 2.9 (2.6, 3.2) | 30.8 (25.9, 36.0) | 0.000 | 77,513.0 | 428/229 | 1.9 (1.7, 2.1) | 25.7 (20.6, 31.2) | 0.000 |
| **Cancer types** |  |  |  |  |  |  |  |  |  |  |
| **1. Leukaemia’s and related disorders** | **18,367.8** | **80/28** | **2.8 (2.2, 3.5)** | **28.0 (19.0, 38.7)** | **0.000** | **15,918.7** | **68/40** | **1.7 (1.3, 2.2)** | **17.6 (8.0, 29.0)** | **0.000** |
| 1.1 Acute lymphoblastic leukaemia | 4,869.5 | 18/5 | 3.3 (2.0, 5.2) | 25.8 (10.8, 47.3) | 0.000 | 3,091.0 | 10/5 | 2.0 (1.0, 3.7) | 16.2 (-0.6, 43.4) | 0.063 |
| 1.2 Acute myeloid leukaemia | 4,546.0 | 21/7 | 3.1 (1.9, 4.8) | 31.4 (13.8, 55.8) | 0.000 | 5,337.6 | 29/15 | 2.0 (1.3, 2.8) | 26.6 (8.6, 50.3) | 0.001 |
| 1.2.1 Acute promyelocytic leukaemia | NA | NA | NA | NA | NA | 1,173.8 | 11/4 | 3.1 (1.5, 5.5) | 63.2 (16.2, 137.1) | 0.002 |
| 1.2.2 Other acute myeloid leukaemia | 3,775.9 | 15/6 | 2.7 (1.5, 4.4) | 24.7 (7.2, 50.5) | 0.002 | 4,163.7 | 18/11 | 1.6 (0.9, 2.5) | 16.2 (-1.4, 41.3) | 0.076 |
| 1.3 Chronic myeloid leukaemia | 3,592.4 | 15/5 | 2.8 (1.6, 4.6) | 26.7 (8.3, 53.8) | 0.001 | 2,583.1 | 10/7 | 1.4 (0.7, 2.5) | 10.6 (-9.5, 43.1) | 0.391 |
| 1.4 Chronic lymphocytic leukaemia | 1,197.7 | 13/3 | 4.4 (2.3, 7.4) | 83.6 (32.9, 160.7) | 0.000 | NA | NA | NA | NA | NA |
| **2. Lymphomas** | **70,172.9** | **350/114** | **3.1 (2.8, 3.4)** | **33.7 (28.6, 39.2)** | **0.000** | **52,583.1** | **336/129** | **2.6 (2.3, 2.9)** | **39.4 (32.7, 46.6)** | **0.000** |
| 2.1 Non-Hodgkin lymphomas | 27,510.4 | 135/48 | 2.8 (2.4, 3.3) | 31.6 (23.7, 40.6) | 0.000 | 17,365.4 | 106/52 | 2.0 (1.7, 2.5) | 31.0 (19.9, 43.8) | 0.000 |
| 2.1.3 Diffuse large B-cell (DLBCL) | 11,061.9 | 61/20 | 3.0 (2.3, 3.9) | 37.0 (24.0, 52.7) | 0.000 | 6,550.9 | 35/19 | 1.8 (1.3, 2.6) | 24.5 (8.3, 45.4) | 0.001 |
| 2.1.5 Anaplastic T-cell and null-cell excluding NK/T-cell | 4,262.4 | 18/7 | 2.4 (1.4, 3.8) | 24.9 (7.7, 49.4) | 0.001 | 2,809.3 | 16/8 | 2.1 (1.2, 3.4) | 29.8 (5.4, 65.4) | 0.011 |
| 2.1.6 Follicular | 4,797.0 | 30/10 | 3.1 (2.1, 4.4) | 42.4 (22.0, 69.1) | 0.000 | 3,690.0 | 26/14 | 1.9 (1.2, 2.7) | 32.6 (8.2, 65.4) | 0.005 |
| 2.1.9 Other non-Hodgkin lymphoma NOS | 2,025.5 | 12/4 | 2.9 (1.5, 5.1) | 39.0 (10.4, 83.3) | 0.002 | NA | NA | NA | NA | NA |
| 2.2 Hodgkin lymphoma | 38,944.7 | 194/58 | 3.4 (2.9, 3.9) | 35.0 (28.2, 42.5) | 0.000 | 33,073.5 | 217/69 | 3.1 (2.7, 3.6) | 44.7 (36.3, 54.0) | 0.000 |
| 2.2.1 Hodgkin NLP | 2,749.4 | 11/4 | 3.0 (1.5, 5.4) | 26.7 (6.7, 58.3) | 0.003 | NA | NA | NA | NA | NA |
| 2.2.2 Hodgkin classic, other | 36,195.2 | 183/54 | 3.4 (2.9, 3.9) | 35.6 (28.5, 43.5) | 0.000 | 32,315.4 | 214/68 | 3.2 (2.8, 3.6) | 45.3 (36.7, 54.8) | 0.000 |
| **3. CNS and other intracranial and intraspinal neoplasms** | **17,864.1** | **59/23** | **2.5 (1.9, 3.3)** | **19.9 (12.1, 29.5)** | **0.000** | **13,347.6** | **55/30** | **1.8 (1.4, 2.4)** | **18.8 (8.6, 31.2)** | **0.000** |
| 3.1 Astroglial and related neoplasms | 15,539.9 | 53/21 | 2.6 (1.9, 3.3) | 20.8 (12.2, 31.3) | 0.000 | 11,528.0 | 48/26 | 1.8 (1.4, 2.4) | 19.1 (8.1, 32.6) | 0.000 |
| 3.1.1 Oligodendriogliomas | 4,147.3 | 15/6 | 2.5 (1.4, 4.0) | 21.4 (5.5, 44.9) | 0.003 | 2,703.4 | 10/7 | 1.5 (0.7, 2.7) | 11.6 (-7.6, 42.7) | 0.311 |
| 3.1.1.2 Oligodendrioglioma, invasive | 4,147.3 | 15/6 | 2.5 (1.4, 4.0) | 21.4 (5.5, 44.9) | 0.003 | 2,703.4 | 10/7 | 1.5 (0.7, 2.7) | 11.6 (-7.6, 42.7) | 0.311 |
| 3.1.4 Other astrocytoma/astroglial neoplasms | 8,515.2 | 26/10 | 2.5 (1.7, 3.7) | 18.5 (7.9, 32.7) | 0.000 | 6,921.2 | 28/15 | 1.9 (1.2, 2.7) | 18.8 (5.2, 36.8) | 0.003 |
| 3.1.4.3 Other astrocytoma/astroglial, invasive | 7,232.1 | 24/9 | 2.7 (1.7, 4.0) | 21.0 (9.0, 37.2) | 0.000 | 6,128.9 | 24/14 | 1.8 (1.1, 2.6) | 17.1 (3.0, 36.2) | 0.013 |
| **4. Sarcomas** | **25,207.5** | **86/41** | **2.1 (1.7, 2.6)** | **17.7 (10.9, 25.7)** | **0.000** | **24,284.5** | **114/67** | **1.7 (1.4, 2.0)** | **19.4 (11.2, 28.9)** | **0.000** |
| 4.1 Osteosarcoma | NA | NA | NA | NA | NA | 2,112.1 | 12/3 | 3.7 (1.9, 6.5) | 41.6 (14.1, 84.0) | 0.000 |
| 4.2 Chondrosarcoma | 3,652.9 | 13/6 | 2.3 (1.2, 3.9) | 19.8 (3.1, 45.1) | 0.013 | 3,661.4 | 20/10 | 2.0 (1.2, 3.1) | 27.5 (6.3, 57.3) | 0.000 |
| 4.4 Fibromatous neoplasms | 7,685.1 | 31/15 | 2.1 (1.4, 3.0) | 21.4 (8.4, 38.3) | 0.000 | 7,942.9 | 25/25 | 1.0 (0.7, 1.5) | 0.6 (-10.5, 15.6) | 1.000 |
| 4.4.3 Other fibromatous neoplasms | 6,312.3 | 23/12 | 1.9 (1.2, 2.8) | 17.2 (3.9, 35.5) | 0.007 | 6,620.1 | 20/20 | 1.0 (0.6, 1.5) | -0.6 (-12.3, 15.9) | 1.000 |
| 4.5 Liposarcoma | 2,310.6 | 10/5 | 2.0 (0.9, 3.6) | 21.4 (-1.1, 57.7) | 0.068 | 2,286.5 | 19/8 | 2.5 (1.5, 3.9) | 49.6 (16.6, 96.3) | 0.001 |
| **5. Blood and lymphatic vessel tumours** | **4,756.8** | **43/9** | **4.8 (3.4, 6.4)** | **71.4 (46.5, 102.8)** | **0.000** | **NA** | **NA** | **NA** | **NA** | **NA** |
| 5.2 Malignant blood and lymphatic vessel tumours, all sites | 4,756.8 | 43/9 | 4.8 (3.4, 6.4) | 71.4 (46.5, 102.8) | 0.000 | NA | NA | NA | NA | NA |
| 5.2.1 Kaposi sarcoma | 4,352.7 | 41/9 | 4.8 (3.4, 6.5) | 74.6 (48.0, 108.1) | 0.000 | NA | NA | NA | NA | NA |
| **7. Gonadal and related tumours** | **137,882.9** | **595/224** | **2.7 (2.5, 2.9)** | **26.9 (23.5, 30.5)** | **0.000** | **28,660.8** | **149/97** | **1.5 (1.3, 1.8)** | **18.1 (10.1, 27.2)** | **0.000** |
| 7.1 Testis | 134,061.2 | 567/219 | 2.6 (2.4, 2.8) | 26.0 (22.6, 29.6) | 0.000 | NA | NA | NA | NA | NA |
| 7.1.1 Germ cell and trophoblastic | 133,789.0 | 565/218 | 2.6 (2.4, 2.8) | 25.9 (22.5, 29.5) | 0.000 | NA | NA | NA | NA | NA |
| 7.1.1.1 Seminoma | 61,723.4 | 295/118 | 2.5 (2.2, 2.8) | 28.7 (23.4, 34.4) | 0.000 | NA | NA | NA | NA | NA |
| 7.1.1.2 Embryonal carcinoma | 20,145.6 | 62/29 | 2.2 (1.7, 2.8) | 16.6 (9.4, 25.3) | 0.000 | NA | NA | NA | NA | NA |
| 7.1.1.4 Teratoma | 2,793.2 | 11/4 | 2.8 (1.4, 5.0) | 25.3 (5.6, 56.4) | 0.000 | NA | NA | NA | NA | NA |
| 7.1.1.5 Mixed germ cell | 22,474.0 | 91/35 | 2.6 (2.1, 3.2) | 25.1 (17.2, 34.3) | 0.000 | NA | NA | NA | NA | NA |
| 7.1.1.6 Choriocarcinoma and other trophoblastic | 19,477.4 | 77/24 | 3.2 (2.5, 4.0) | 27.2 (18.8, 37.1) | 0.000 | NA | NA | NA | NA | NA |
| 7.1.1.7 Other | 6,091.3 | 23/8 | 2.9 (1.8, 4.3) | 24.7 (10.9, 43.6) | 0.000 | NA | NA | NA | NA | NA |
| 7.2 Ovary | NA | NA | NA | NA | NA | 25,895.7 | 138/89 | 1.6 (1.3, 1.8) | 18.9 (10.4, 28.6) | 0.000 |
| 7.2.1 Germ cell and trophoblastic | NA | NA | NA | NA | NA | 4,147.3 | 18/9 | 2.0 (1.2, 3.1) | 21.6 (3.9, 46.8) | 0.011 |
| 7.2.2 Non-germ cell | NA | NA | NA | NA | NA | 21,748.3 | 120/80 | 1.5 (1.2, 1.8) | 18.4 (9.0, 29.2) | 0.000 |
| 7.2.2.1 Carcinoma | NA | NA | NA | NA | NA | 20,915.2 | 116/77 | 1.5 (1.3, 1.8) | 18.8 (9.2, 29.9) | 0.000 |
| 7.2.2.1.1 Adenocarcinoma | NA | NA | NA | NA | NA | 20,494.9 | 115/75 | 1.5 (1.3, 1.8) | 19.4 (9.6, 30.6) | 0.000 |
| 7.2.2.1.1.2 Cystadenocarcinoma | NA | NA | NA | NA | NA | 16,514.1 | 94/60 | 1.6 (1.3, 1.9) | 20.7 (9.8, 33.4) | 0.000 |
| 7.2.2.2 Sex cord and other specialized gonadal | 2,660.0 | 22/4 | 5.7 (3.6, 8.7) | 68.2 (37.4, 110.8) | 0.000 | 2,445.3 | 11/8 | 1.5 (0.7, 2.6) | 14.0 (-8.5, 49.5) | 0.288 |
| 7.4 Germ cell and trophoblastic excluding CNS, ovary, testis | 1,839.9 | 13/3 | 4.7 (2.5, 8.0) | 55.6 (22.6, 105.8) | 0.000 | NA | NA | NA | NA | NA |
| 7.4.1 Germ cell tumours including non-gestational Trophoblastic tumours | NA | NA | NA | NA | NA | 2,374.7 | 10/7 | 1.4 (0.6, 2.5) | 11.0 (-10.9, 46.3) | 0.423 |
| 7.6 Fibroepithelial including Brenner, excluding breast phyllodes | NA | NA | NA | NA | NA | 25,895.7 | 138/89 | 1.6 (1.3, 1.8) | 18.9 (10.4, 28.6) | 0.000 |
| **8. Melanoma, malignant** | **64,422.8** | **341/124** | **2.8 (2.5, 3.1)** | **33.7 (28.3, 39.7)** | **0.000** | **130,450.4** | **866/410** | **2.1 (2.0, 2.3)** | **34.9 (30.6, 39.5)** | **0.000** |
| 8.1 Superficial spreading/low cumulative sun damage melanoma | 39,629.5 | 225/74 | 3.0 (2.7, 3.5) | 38.1 (31.0, 46.1) | 0.000 | 85,720.6 | 574/264 | 2.2 (2.0, 2.4) | 36.2 (30.8, 41.9) | 0.000 |
| 8.2 Nodular melanoma | 6,875.2 | 32/13 | 2.4 (1.7, 3.5) | 27.5 (12.8, 46.7) | 0.000 | 10,931.1 | 84/33 | 2.6 (2.1, 3.2) | 47.0 (31.5, 65.3) | 0.000 |
| 8.3 Other malignant | 17,918.2 | 84/37 | 2.3 (1.8, 2.8) | 26.4 (16.9, 37.6) | 0.000 | 33,798.7 | 208/114 | 1.8 (1.6, 2.1) | 27.9 (19.8, 36.8) | 0.000 |
| **9. Carcinomas** | **63,349.5** | **458/135** | **3.4 (3.1, 3.7)** | **51.0 (44.5, 57.9)** | **0.000** | **353,582.0** | **2,932/1,308** | **2.2 (2.2, 2.3)** | **45.9 (43.0, 49.0)** | **0.000** |
| 9.1 Thyroid carcinoma | 10,009.1 | 34/19 | 1.8 (1.2, 2.5) | 14.8 (4.4, 28.3) | 0.003 | 32,240.4 | 122/98 | 1.2 (1.0, 1.5) | 7.4 (1.0, 14.8) | 0.022 |
| 9.1.1 Medullary | NA | NA | NA | NA | NA | 1,573.5 | 10/4 | 2.3 (1.1, 4.3) | 36.2 (3.1, 89.5) | 0.026 |
| 9.1.3 Papillary | 5,611.3 | 16/11 | 1.5 (0.8, 2.4) | 9.3 (-2.9, 27.1) | 0.161 | 16,857.0 | 48/49 | 1.0 (0.7, 1.3) | -0.6 (-8.1, 8.6) | 0.953 |
| 9.1.4 Follicular | NA | NA | NA | NA | NA | 5,270.5 | 25/16 | 1.5 (1.0, 2.3) | 16.4 (-0.3, 39.0) | 0.055 |
| 9.1.5 Papillary with follicular variant | NA | NA | NA | NA | NA | 7,884.3 | 37/26 | 1.4 (1.0, 1.9) | 13.5 (-0.4, 31.2) | 0.058 |
| 9.2 Other carcinoma of head and neck | 10,898.4 | 104/24 | 4.4 (3.6, 5.4) | 73.9 (56.4, 94.1) | 0.000 | 8,499.0 | 91/27 | 3.3 (2.7, 4.1) | 74.9 (54.1, 99.3) | 0.000 |
| 9.2.1 Nasopharyngeal carcinoma | 1,452.8 | 10/2 | 4.7 (2.2, 8.6) | 54.1 (18.2, 111.8) | 0.000 | NA | NA | NA | NA | NA |
| 9.2.2 Oral cavity, lip, and pharynx | 5,650.8 | 66/13 | 5.1 (3.9, 6.5) | 93.8 (67.3, 125.6) | 0.000 | 3,638.0 | 54/12 | 4.4 (3.3, 5.7) | 114.7 (77.7, 159.9) | 0.000 |
| 9.2.2.1 Oral cavity, lip, and pharynx, squamous | 4,440.9 | 62/10 | 5.9 (4.6, 7.6) | 116.1 (83.6, 155.5) | 0.000 | 2,370.7 | 50/8 | 5.9 (4.4, 7.8) | 175.2 (120.8, 242.4) | 0.000 |
| 9.2.3 Salivary gland | NA | NA | NA | NA | NA | 2,933.7 | 16/8 | 1.9 (1.1, 3.1) | 25.9 (2.5, 59.9) | 0.025 |
| 9.2.3.2 Salivary gland, other malignant | NA | NA | NA | NA | NA | 1,993.6 | 13/6 | 2.1 (1.1, 3.6) | 34.4 (3.9, 80.7) | 0.021 |
| 9.2.4 Other carcinoma of head and neck | 1,886.0 | 25/5 | 5.0 (3.2, 7.4) | 106.0 (59.2, 169.1) | 0.000 | 1,011.5 | 15/4 | 3.9 (2.2, 6.5) | 110.5 (45.2, 206.8) | 0.000 |
| 9.3 Carcinoma of gastrointestinal tract | 22,900.4 | 157/49 | 3.2 (2.7, 3.8) | 47.4 (37.1, 59.0) | 0.000 | 25,332.2 | 155/82 | 1.9 (1.6, 2.2) | 28.7 (19.4, 39.1) | 0.000 |
| 9.3.2 Carcinoma of stomach | 2,117.7 | 11/5 | 2.2 (1.1, 3.9) | 28.1 (2.1, 69.1) | 0.000 | 1,635.3 | 10/5 | 1.9 (0.9, 3.5) | 28.6 (-3.2, 79.9) | 0.090 |
| 9.3.4 Carcinoma of colon | 12,200.0 | 95/25 | 3.7 (3.0, 4.6) | 57.1 (42.2, 74.4) | 0.000 | 15,132.2 | 91/47 | 1.9 (1.5, 2.4) | 28.8 (17.0, 42.5) | 0.000 |
| 9.3.4.1 Appendix | NA | NA | NA | NA | NA | 6,997.4 | 15/17 | 0.9 (0.5, 1.5) | -2.9 (-12.4, 11.0) | 0.734 |
| 9.3.4.1.1 NET | NA | NA | NA | NA | NA | 6,476.1 | 14/15 | 0.9 (0.5, 1.5) | -1.9 (-11.7, 12.7) | 0.879 |
| 9.3.4.2 Colon excluding appendix | 8,564.3 | 86/20 | 4.3 (3.4, 5.3) | 76.8 (56.7, 100.4) | 0.000 | 8,134.8 | 76/30 | 2.5 (2.0, 3.1) | 56.0 (36.2, 79.5) | 0.000 |
| 9.3.4.2.2 Colon excluding appendix, adenocarcinoma | 8,348.0 | 84/20 | 4.2 (3.4, 5.3) | 76.9 (56.5, 100.9) | 0.000 | 7,898.3 | 74/30 | 2.5 (1.9, 3.1) | 55.9 (35.8, 79.9) | 0.000 |
| 9.3.5 Carcinoma of rectum | 5,744.9 | 29/13 | 2.3 (1.6, 3.3) | 28.7 (12.0, 50.7) | 0.000 | 5,614.0 | 30/20 | 1.5 (1.0, 2.1) | 17.3 (-0.1, 40.1) | 0.052 |
| 9.3.5.2 Rectum, adenocarcinoma | 5,092.6 | 27/11 | 2.4 (1.6, 3.5) | 30.8 (12.7, 54.9) | 0.000 | 4,782.1 | 25/18 | 1.4 (0.9, 2.1) | 14.9 (-3.5, 39.8) | 0.128 |
| 9.4 Carcinoma of lung, bronchus, and trachea | 3,327.4 | 28/6 | 4.3 (2.9, 6.2) | 64.7 (36.4, 102.1) | 0.000 | 5,307.3 | 40/19 | 2.1 (1.5, 2.9) | 40.2 (18.7, 67.5) | 0.000 |
| 9.4.2 Non-small cell carcinoma | 3,148.3 | 24/6 | 3.9 (2.5, 5.8) | 56.8 (29.4, 94.0) | 0.000 | 5,172.5 | 39/18 | 2.1 (1.5, 2.9) | 40.3 (18.5, 68.0) | 0.000 |
| 9.4.2.1 Non-small cell, adenocarcinoma | NA | NA | NA | NA | NA | 1,252.2 | 17/4 | 3.9 (2.3, 6.3) | 101.2 (44.5, 182.8) | 0.000 |
| 9.4.2.2 Non-small cell, neuroendocrine | 1,752.2 | 10/3 | 3.1 (1.5, 5.7) | 38.7 (9.0, 86.6) | 0.004 | 3,239.7 | 10/11 | 0.9 (0.4, 1.6) | -3.6 (-19.7, 22.3) | 0.878 |
| 9.4.2.3 Non-small cell, other | 605.1 | 10/1 | 6.7 (3.2, 12.4) | 140.7 (54.7, 279.4) | 0.000 | 680.6 | 12/3 | 4.5 (2.3, 7.9) | 137.3 (52.1, 269.0) | 0.000 |
| 9.5 Carcinoma of skin (if collected) | 5,288.7 | 47/12 | 4.0 (2.9, 5.3) | 66.7 (43.1, 96.0) | 0.000 | 6,179.0 | 48/23 | 2.1 (1.5, 2.8) | 40.2 (19.8, 65.5) | 0.000 |
| 9.6 Carcinoma of breast | NA | NA | NA | NA | NA | 185,785.3 | 1,964/709 | 2.8 (2.6, 2.9) | 67.5 (62.9, 72.3) | 0.000 |
| 9.6.1 Breast, infiltrating duct | NA | NA | NA | NA | NA | 147,325.8 | 1,502/551 | 2.7 (2.6, 2.9) | 64.6 (59.5, 69.9) | 0.000 |
| 9.6.2 Breast, adenocarcinoma | NA | NA | NA | NA | NA | 16,691.0 | 184/69 | 2.7 (2.3, 3.1) | 68.7 (53.3, 85.8) | 0.000 |
| 9.6.3 Breast, lobular | NA | NA | NA | NA | NA | 8,939.7 | 112/37 | 3.0 (2.5, 3.6) | 83.8 (61.6, 109.2) | 0.000 |
| 9.6.5 Breast, medullary | NA | NA | NA | NA | NA | 5,587.8 | 81/23 | 3.5 (2.8, 4.4) | 103.8 (74.0, 139.1) | 0.000 |
| 9.6.10 Breast, other | NA | NA | NA | NA | NA | 4,678.2 | 62/20 | 3.1 (2.4, 4.0) | 90.1 (59.2, 127.5) | 0.000 |
| 9.7 Carcinoma of genital sites excluding ovary and testis | NA | NA | NA | NA | NA | 82,961.6 | 455/323 | 1.4 (1.3, 1.5) | 15.9 (11.0, 21.2) | 0.000 |
| 9.7.1 Carcinoma of uterine cervix | NA | NA | NA | NA | NA | 75,522.7 | 398/292 | 1.4 (1.2, 1.5) | 14.0 (9.0, 19.5) | 0.000 |
| 9.7.1.1 Cervix, squamous | NA | NA | NA | NA | NA | 53,420.7 | 270/207 | 1.3 (1.2, 1.5) | 11.9 (6.0, 18.3) | 0.000 |
| 9.7.1.2 Cervix, adenosquamous | NA | NA | NA | NA | NA | 2,521.0 | 14/10 | 1.5 (0.8, 2.5) | 17.8 (-7.4, 55.4) | 0.206 |
| 9.7.1.3 Cervix, adenocarcinoma | NA | NA | NA | NA | NA | 14,321.5 | 91/53 | 1.7 (1.4, 2.1) | 26.8 (14.5, 41.3) | 0.000 |
| 9.7.1.4 Cervix, other | NA | NA | NA | NA | NA | 5,259.4 | 23/23 | 1.0 (0.6, 1.5) | -0.5 (-16.6, 21.3) | 1.000 |
| 9.7.2 Corpus uteri | NA | NA | NA | NA | NA | 3,719.4 | 19/16 | 1.2 (0.7, 1.8) | 7.0 (-13.4, 35.7) | 0.585 |
| 9.7.2.1 Corpus uteri, adenocarcinoma | NA | NA | NA | NA | NA | 2,834.2 | 16/13 | 1.3 (0.7, 2.1) | 12.0 (-12.2, 47.2) | 0.405 |
| 9.7.2.1.2 Corpus uteri, other adenocarcinoma | NA | NA | NA | NA | NA | 1,842.3 | 15/9 | 1.6 (0.9, 2.6) | 30.3 (-5.5, 83.2) | 0.113 |
| 9.7.3 Carcinoma of vulva and vagina | NA | NA | NA | NA | NA | 3,539.5 | 38/14 | 2.7 (1.9, 3.7) | 67.7 (36.3, 107.7) | 0.000 |
| 9.8 Carcinoma of urinary tract | 8,226.1 | 72/20 | 3.6 (2.8, 4.6) | 63.4 (44.3, 86.1) | 0.000 | 5,308.9 | 41/19 | 2.1 (1.5, 2.9) | 41.1 (19.2, 68.6) | 0.000 |
| 9.8.1 Carcinoma of kidney | 5,290.7 | 49/12 | 4.0 (2.9, 5.3) | 69.4 (45.3, 99.2) | 0.000 | 3,829.0 | 23/14 | 1.7 (1.1, 2.5) | 24.5 (2.5, 54.6) | 0.025 |
| 9.8.1.1 Kidney, adenocarcinoma | 5,244.6 | 49/12 | 4.0 (3.0, 5.3) | 70.2 (45.8, 100.2) | 0.000 | 3,829.0 | 23/14 | 1.7 (1.1, 2.5) | 24.5 (2.5, 54.6) | 0.025 |
| 9.8.1.1.1 Kidney, renal cell | 4,909.7 | 42/12 | 3.6 (2.6, 4.9) | 62.1 (38.2, 92.2) | 0.000 | 3,504.4 | 22/13 | 1.7 (1.1, 2.6) | 26.8 (3.3, 59.0) | 0.021 |
| 9.8.2 Carcinoma of bladder | 2,550.9 | 20/6 | 3.1 (1.9, 4.8) | 53.1 (22.6, 95.8) | 0.000 | 1,246.5 | 14/5 | 3.0 (1.7, 5.1) | 75.4 (24.5, 151.5) | 0.001 |
| 9.8.2.1 Urinary bladder, transitional cell carcinoma | 2,334.0 | 16/6 | 2.6 (1.5, 4.3) | 42.6 (13.3, 85.4) | 0.001 | 973.8 | 12/3 | 3.4 (1.8, 6.0) | 87.3 (27.7, 179.3) | 0.001 |
| 9.9 Other invasive carcinomas | 1,434.8 | 11/3 | 4.0 (2.0, 7.1) | 57.4 (19.0, 117.9) | 0.000 | 1,968.2 | 16/7 | 2.4 (1.3, 3.8) | 46.8 (12.0, 97.5) | 0.004 |
| **10. Miscellaneous specified neoplasms** | **NA** | **NA** | **NA** | **NA** | **NA** | **1,487.3** | **10/5** | **2.0 (1.0, 3.7)** | **33.8 (-1.2, 90.3)** | **0.061** |

Abbreviation: TNM=Tumour, Node, Metastasis, Figo=Fédération Internationale de Gynécologie et d'Obstétrique, NOS=Not otherwise specified, CNS=Central Nervous System, NA=Not Applicable.

^a^ Age at diagnosis of first primary cancer.
